# The N6-methyladenosine-mediated cLMNB1 degrades FGFR4 to overcome osimertinib resistance in non-small cell lung cancer

**DOI:** 10.1038/s41419-025-08124-8

**Published:** 2025-11-10

**Authors:** Yuxian Qian, Hui Wang, Yipeng Feng, Yijian Zhang, Qianfan Hu, Zehao Pan, Xiaodong Zhang, Lin Xu, Li Yin, Gaochao Dong, Xing Zhang, Feng Jiang

**Affiliations:** 1 Department of Thoracic Surgery, Affiliated Cancer Hospital of Nanjing Medical University and Jiangsu Cancer Hospital and Jiangsu Institute of Cancer Research, Nanjing, China; 2 Jiangsu Key Laboratory of Molecular and Translational Cancer Research, Nanjing, China; 3 The Fourth Clinical College of Nanjing Medical University, Nanjing, P. R. China; 4 Department of Medical Oncology, The Affiliated Tumor Hospital of Nantong University, Nantong, China; 5 Collaborative Innovation Center for Cancer Personalized Medicine, Nanjing Medical University, Nanjing, China

**Keywords:** Cancer therapeutic resistance, RNA, Non-small-cell lung cancer, Drug delivery

## Abstract

Osimertinib resistance is the main challenge in treating EGFR-mutant lung adenocarcinoma (LUAD). The role of N6-methyladenosine (m6A) modification of circular RNAs (circRNAs) in osimertinib-resistant LUAD remains largely unknown. We used MeRIP-seq and circRNA-seq to screen for potential circRNA candidates that influence osimertinib resistance. It was observed that circRNA LMNB1 (cLMNB1) increased the sensitivity of LUAD to osimertinib in vitro and in vivo. Mechanistically, cLMNB1 acts as a scaffold between fibroblast growth factor receptor 4 (FGFR4) and E3 ubiquitin-protein ligase CBL (c-Cbl), enhancing the ubiquitin-dependent degradation of FGFR4. Furthermore, METTL3 and YTHDF2 are responsible for increased m6A modification levels and decreased cLMNB1 expression in osimertinib-resistant LUAD without affecting its functions. Our findings demonstrate that cLMNB1, mediated by m6A modification, overcomes osimertinib resistance by destabilizing the FGFR4 protein in LUAD. cLMNB1 with an m6A modification site mutation (cLMNB1-mut) could be a promising nucleic acid drug, as it has shown excellent efficacy in osimertinib-resistant preclinical models of LUAD.

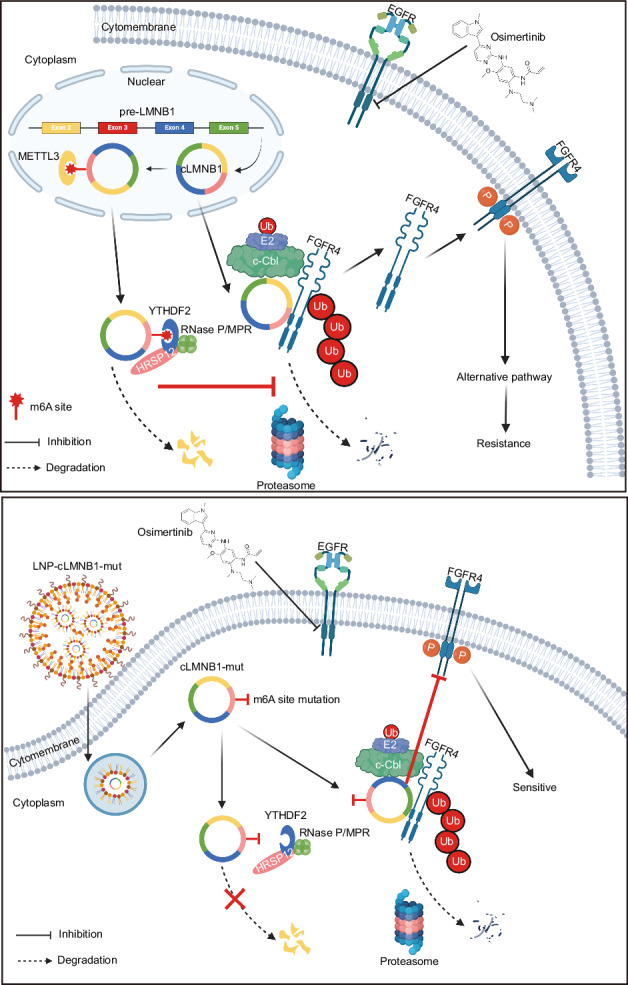

## Introduction

Lung cancer is the most common cancer type worldwide and has the highest incidence and mortality rates [1, 2]. Approximately 85% of lung cancers are non-small cell lung cancers (NSCLC), with lung adenocarcinoma (LUAD) being the main histological type [1]. The recommended first-line treatment for patients with advanced LUAD who carry epidermal growth factor receptor (EGFR) mutations is EGFR tyrosine kinase inhibitors (EGFR-TKIs) such as osimertinib [3]. However, most patients receiving osimertinib as the first-line treatment develop acquired resistance within an average of two years [4]. Currently, the main studies on the mechanism of osimertinib resistance have reported self-mutations of EGFR and EGFR-independent mechanisms, including compensatory activation of signaling pathways and histological transformation [5, 6]. However, efficient strategies to address this resistance remain lacking.

Circular RNAs (circRNAs) are closed-loop noncoding RNAs endowed with a longer half-life than linear RNA molecules. CircRNAs can perform various biological functions through a range of mechanisms, including functioning as microRNA (miRNA) sponges, interacting with proteins, and translating into proteins [7]. The N6-methyladenosine (m6A) modification is an epigenetic modification in eukaryotic cells that regulates gene expression at the post-transcriptional level, which has also been found in circRNAs [8]. Previous studies [9–11] have identified a significant association between m6A methylation and osimertinib resistance in LUAD. For instance, METTL7B has been shown to increase the m6A methylation levels of mRNAs encoding antioxidant enzymes, facilitating the development of osimertinib resistance by maintaining the balance of reactive oxygen species [11]. Nevertheless, the biological functions of m6A-modified circRNAs in osimertinib-resistant LUAD are largely unknown.

Fibroblast growth factor receptors (FGFRs) are a gene family of transmembrane tyrosine kinase receptors that activate important downstream signaling pathways [12]. Among them, FGFR4 has been reported to promote cancer cell proliferation, invasion, survival, and chemoresistance in multiple cancer types [13]. *FGFR1* gene amplification is relatively common in NSCLC, resulting in drug resistance and poor prognosis [14]. In hepatocellular carcinoma (HCC), FGFR inhibition by lenvatinib leads to feedback activation of the EGFR-PAK2-ERK5 signaling axis, which is blocked by gefitinib [15]. Indeed, dual inhibition of EGFR and FGFR is effective in NSCLC [16], but the role of FGFR4 in osimertinib-resistant LUAD has rarely been reported.

Lipid nanoparticle (LNP)-encapsulated mRNA vaccines produced by Moderna and Pfizer-BioNTech have protected millions of people against COVID-19 [17]. Recently, LNP-encapsulated circRNAs have emerged as a prospective strategy for treating cancer due to their long half-life and low immunogenicity [18].

This study found that the circRNA LMNB1 (cLMNB1) enhanced the interaction between FGFR4 and the E3 ubiquitin-protein ligase CBL (c-Cbl), overcoming osimertinib resistance in LUAD. In addition, we found that cLMNB1 is degraded by the reader protein YTHDF2 after undergoing METTL3-mediated m6A modification. Furthermore, LNP-encapsulated cLMNB1 with an m6A modification site mutation (cLMNB1-mut) exhibited superior efficacy in osimertinib-resistant LUAD.

## Results

### cLMNB1 is poorly expressed and highly methylated in osimertinib-resistant LUAD

We generated osimertinib-resistant cells by continuously exposing osimertinib-sensitive LUAD cell lines PC9 and HCC827 with EGFR mutations to osimertinib for three months in vitro. The resulting osimertinib-resistant cell lines, PC9OR (OR means osimertinib resistant) and HCC827OR, showed higher IC50 values for osimertinib than their respective parental cell lines (Supplementary Fig. 1A). Bright-field micrographs of cultured parental and resistant cells are shown in Supplementary Fig. 1B. Xenograft experiments confirmed that the resistant cells had a significant tolerance to osimertinib (Supplementary Fig. 1C, D).

For a subsequent study, we collected 20 osimertinib-resistant samples of puncturing lung biopsy and 20 naïve samples as controls. The m6A dot blot assays detected higher m6A modification levels in resistant cell lines and osimertinib-resistant LUAD (LUAD-OR) tissues (Supplementary Fig. 2A, B). The immunohistochemical (IHC) staining suggested that m6A modification levels in LUAD-OR tissues were higher than in osimertinib-sensitive LUAD tissues (Supplementary Fig. 2C). Moreover, the increased m6A modification levels were validated by quantitative assays in the PC9OR and HCC827OR cell lines, and LUAD-OR tissues (Supplementary Fig. 2D–F). These results above strongly point to the involvement of m6A modifications in therapy resistance.

**Fig. 1:**
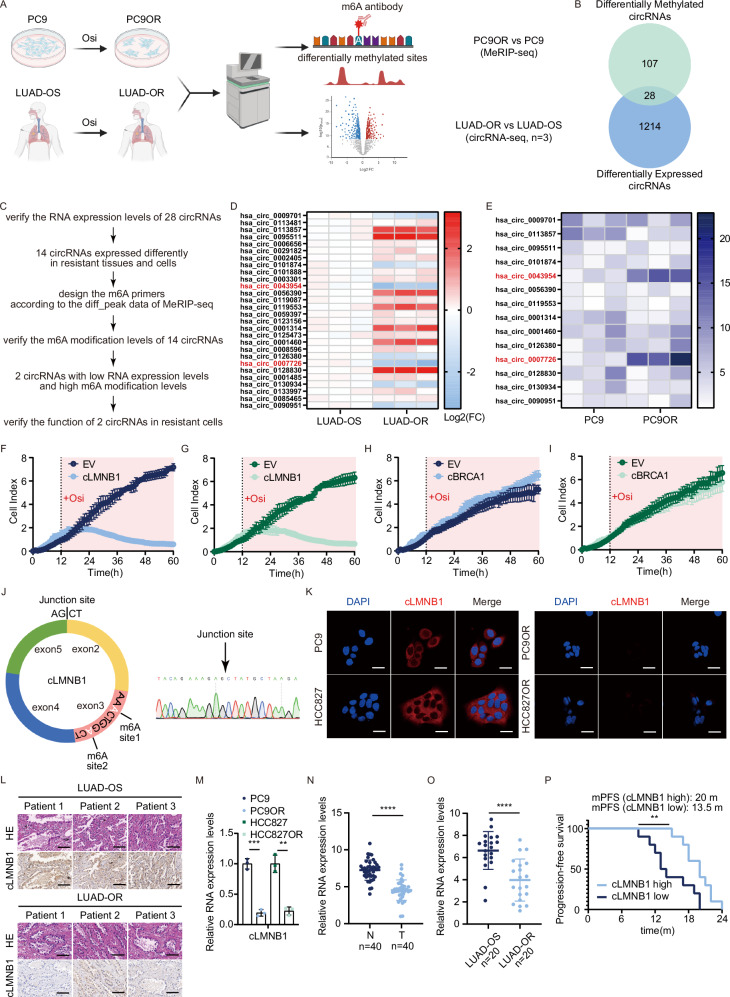
cLMNB1 is poorly expressed and highly methylated in osimertinib-resistant LUAD. **A** PC9 and PC9OR cells were collected, followed by MeRIP-seq (*n* = 3). Lung tissues of patients (LUAD-OS and LUAD-OR) were collected for circRNA-seq (*n* = 3). **B** Venn diagram showing the overlap of circRNAs from two sequencings. 107 differentially methylated peaks were detected. The 1214 circRNAs were expressed differentially. **C** The flow chart of screening procedures. **D** The expression levels of 28 circRNAs in LUAD tissues were detected by qPCR (*n* = 3). Each grid represented the mean value of triplicate data from one lung tissue. The fold change took the log function. Red represented up and blue meant down. **E** The m6A modification levels of 14 circRNAs in PC9 and PC9OR cells were detected by MeRIP-qPCR (*n* = 3). The darker blue represented the larger fold change. **F**, **G** RTCA of PC9OR and HCC827OR cells stably transfected with EV or cLMNB1 (*n* = 3). **H**, **I** RTCA of PC9OR and HCC827OR cells stably transfected with EV or cBRCA1 (*n* = 3). **J** Schematic diagram of cLMNB1 (left). The cDNA of PC9OR was amplified by qPCR using divergent cLMNB1 primers, and the product was subsequently sequenced using Sanger sequencing to verify the junction sequence. (right). **K** Left, FISH for cLMNB1 cellular localization in PC9 and HCC827 cells (*n* = 3), scale bar, 20 μm. Right, FISH for cLMNB1 cellular localization in PC9OR and HCC827OR cells (*n* = 3), scale bar, 20 μm. **L** ISH of cLMNB1 in sensitive and resistant LUAD tissues. Scale bars, 50 μm. **M** Expression of cLMNB1 in PC9, HCC827, PC9OR and HCC827OR cells (*n* = 3). **N** Expression of cLMNB1 in LUAD tissues and paired normal lung tissues (*n* = 40). **O** Expression of cLMNB1 in sensitive and resistant LUAD tissues (*n* = 20). **P** Kaplan–Meier survival analysis of patients with low and high expression of cLMNB1. Three independent experiments were conducted for each result. **p < 0.01, ***p < 0.001, ****p < 0.0001 compared with the controls.

Aberrant m6A status was associated with abnormal circRNA expression and molecular dysfunction [19]. However, the relationship between m6A-modified circRNAs and osimertinib resistance has rarely been reported. We conducted MeRIP-seq analysis on PC9 and PC9OR cells and circRNA-seq on three LUAD-OR and three LUAD-OS tissues, seeking circRNAs with different expression and m6A modification levels (Fig. 1A). We identified 1214 differentially expressed and 107 differentially methylated circRNAs. By intersecting distinct expression and m6A modification profiles, we obtained 55 circRNAs. From these, we eliminated circRNAs not cataloged in the circRNA Database, resulting in a final candidate set of 28 circRNAs (Fig. 1B). The circRNA screening workflow is presented in Fig. 1C. We validated the expression of these 28 circRNAs through quantitative real-time polymerase chain reaction (RT-qPCR) in LUAD tissues and cell lines. Only fourteen circRNAs exhibited differential expression levels in resistant tissues and cells (Fig. 1D and Supplementary Fig. 2G, H). The putative methylation peak of each circRNA was deduced from the MeRIP-seq data, and primers for methylated RNA immunoprecipitation followed by qPCR (MeRIP-qPCR) were custom-designed based on their respective sequences. Subsequently, we used MeRIP-qPCR to investigate whether these 14 circRNAs displayed disparities in m6A modification levels in both resistant cell lines (Fig. 1E and Supplementary Fig. 2I). Following these screening procedures, we detected two downregulated circRNAs with high m6A modification levels, has_circ_0007726 (cLMNB1) and has_circ_0043954 (cBRCA1), in osimertinib-resistant cells and tissues. Subsequently, we transfected PC9OR and HCC827OR cells with cLMNB1 and cBRCA1 plasmids, respectively, and monitored cell proliferation using a real-time cell analysis (RTCA) device at hourly intervals. Our findings revealed comparable cell growth rates in the experimental and control groups in the absence of osimertinib. However, the growth of osimertinib-resistant cells transfected with cLMNB1, but not cBRCA1, was impeded when osimertinib was introduced (Fig. 1F–I). We opted to prioritize cLMNB1 for subsequent investigation due to its notable m6A modification and potential pivotal roles in suppressing osimertinib resistance in LUAD.

cLMNB1 (has_circ_0007726) is formed by the circularization of exons 2–5 of the *LMNB1* gene (Fig. 1J left). The back-splicing site was confirmed by Sanger sequencing in LUAD cells (Fig. 1J, right). As shown in Supplementary Fig. 3A, cLMNB1 could be amplified from cDNA but not gDNA, indicating that cLMNB1 was a back-splicing product of pre-mRNA. Additionally, qPCR and fluorescence in situ hybridization (FISH) assays showed the predominant cytoplasmic distribution of cLMNB1 (Supplementary Fig. 3B, D and Fig. 1K). Notably, under identical imaging conditions, the fluorescence intensity of cLMNB1 was significantly lower in resistant cell lines compared to parental cells, further aligning with sequencing and qPCR validation data (Fig. 1K). Moreover, cLMNB1 was more resistant to RNase R degradation than the linear form of *LMNB1* mRNA (Supplementary Fig. 3C). The actinomycin D assay demonstrated that the half-life of cLMNB1 was much longer than that of the linear *LMNB1* transcript (Supplementary Fig. 3E, F).

In situ hybridization (ISH) assay also verified the low expression of cLMNB1 in LUAD-OR tissues (Fig. 1L). The expression of cLMNB1 was decreased in both resistant cell lines (Fig. 1M). Its expression levels were lower in tumor tissues than in normal tissues (Fig. 1N). We also observed the downregulation of cLMNB1 in LUAD-OR tissues (Fig. 1O). Kaplan–Meier plots demonstrated that high cLMNB1 expression in our cohort of 20 LUAD-OR patients was associated with a delayed onset of resistance and longer progression-free survival (Fig. 1P). Based on these results, we speculated that cLMNB1 suppresses osimertinib resistance in LUAD.

### cLMNB1 overcomes osimertinib resistance in vitro and in vivo independently of m6A modifications

**Fig. 2:**
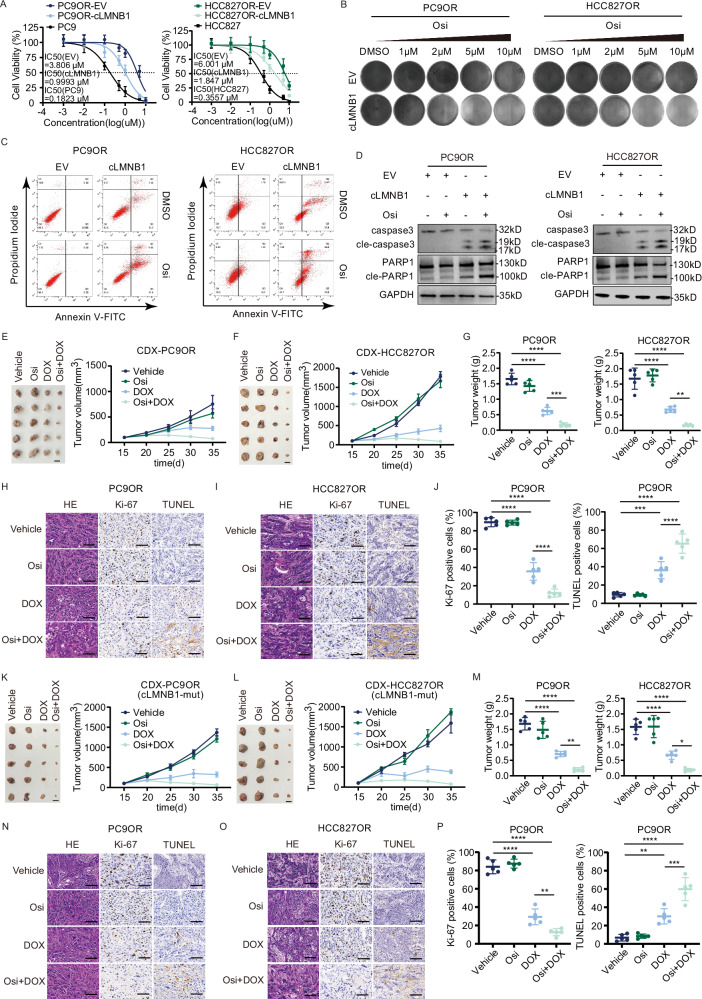
cLMNB1 overcomes osimertinib resistance in vitro and in vivo independently of m6A modifications. **A** PC9, HCC827, PC9OR, and HCC827OR cells were treated with osimertinib (Osi) at the indicated concentrations. IC50 was assessed after 3 days. **B** PC9OR, and HCC827OR cells with cLMNB1 overexpression were treated with osimertinib (Osi) at the indicated concentrations, followed by crystal violet staining. **C** Apoptosis levels of PC9OR (left) and HCC827OR (right) cells treated with osimertinib were analyzed by FACS. **D** The protein levels of caspase3, PARP1, and their cleavage forms of PC9OR (left) and HCC827OR (right) cells treated with osimertinib were analyzed by Western blot. **E**, **F** PC9OR, and HCC827OR cells were stably transfected with DOX-ON-cLMNB1. Mice bearing PC9OR and HCC827OR cell xenograft tumors were treated with vehicle control, Osi for 20 days, followed by treatment cessation and follow-up (*n* = 5). Scale bar, 10 mm. **G** Tumor weight of Vehicle, Osi, DOX, and Osi + DOX group at day 35 (*n* = 5). **H**, **I** Immunohistochemistry (H&E, Ki-67, and TUNEL stain, 40×) analysis of xenograft tumors from (**E**, **F**). Scale bar, 50 μm. **J** Per cent Ki-67 positive cells and per cent necrosis area of PC9OR cell xenograft tumors from (**E**) are plotted. **K**, **L** PC9OR, and HCC827OR cells were stably transfected with DOX-ON-cLMNB1-mut. Mice bearing PC9OR and HCC827OR cell xenograft tumors were treated with vehicle control, Osi for 20 days, followed by treatment cessation and follow-up (*n* = 5). Scale bar, 10 mm. **M** Tumor weight of Vehicle, Osi, DOX, and Osi + DOX group at day 35 (*n* = 5). **N**, **O** Immunohistochemistry (H&E, Ki-67, and TUNEL stain, 40×) analysis of xenograft tumors from (**K**, **L**). Scale bar, 50 μm. **P** Per cent Ki-67 positive cells and per cent necrosis area of PC9OR cell xenograft tumors from (**K**) are plotted. Three independent experiments were conducted for each result. *p < 0.05, **p < 0.01, ***p < 0.001, ****p < 0.0001 compared with the controls.

To elucidate the role of cLMNB1 in regulating osimertinib resistance in LUAD, we constructed PC9OR and HCC827OR cells with stable overexpression of cLMNB1. As shown in Supplementary Fig. 4A, the overexpression efficiency of cLMNB1 was verified. Using the Cell Counting Kit-8 assay, we observed lower IC50 values in PC9OR and HCC827OR cells with cLMNB1 overexpression than in those without, indicating that cLMNB1 enhanced their sensitivity to osimertinib (Fig. 2A). Treating PC9OR and HCC827OR cells for three days with various osimertinib concentrations determined by the IC50 values in Fig. 2A and staining them with crystal violet (Fig. 2B), we found that cLMNB1 disrupted the tolerance of PC9OR and HCC827OR cells to osimertinib. Flow cytometry assays showed that cLMNB1 overexpression induced slight apoptosis in PC9OR and HCC827OR cells, while cLMNB1 overexpression with osimertinib had a fatal impact on these cells (Fig. 2C and Supplementary Fig. 4B). PC9OR and HCC827OR cells with cLMNB1 overexpression exhibited the highest level of apoptosis when exposed to osimertinib at a concentration equivalent to the IC50 value for three days, as evidenced by the upregulation of caspase-3 and PARP1 cleavage observed by western blot (Fig. 2D). In conclusion, cLMNB1 enhanced the sensitivity of LUAD-OR to osimertinib in vitro.

We then designed a TET-ON-cLMNB1 overexpression vector to test the therapeutic effect of cLMNB1 after tumor formation. PC9OR and HCC827OR cells stably transfected with TET-ON-cLMNB1 were only expressed when mice were fed with DOX (Supplementary Fig. 4C). PC9OR and HCC827OR cells stably transfected with TET-ON-cLMNB1 were transplanted subcutaneously into BALB/c-nude mice. After developing palpable tumors, the mice were randomly assigned to four groups. The time for tumors to reach 0.5 cm^3^ in the DOX and Osi + DOX groups was significantly longer than in the Vehicle and Osi groups, indicating that the tumor growth in vivo was significantly attenuated by cLMNB1 overexpression alone or with osimertinib. Moreover, the Osi + DOX group achieved better tumor remission than the DOX group (Fig. 2E–G). IHC staining of the tumors showed lower Ki-67 expression and a higher TUNEL positivity rate in tumors under the DOX and Osi + DOX treatments, indicating decreased proliferation and increased apoptosis (Fig. 2H–J and Supplementary Fig. 4D). These findings confirmed that cLMNB1 could overcome osimertinib resistance in vivo.

The SRAMP database suggested that cLMNB1 had six potential m6A modification sites (Supplementary Data 1). As the schematic drawing of cLMNB1 shows (Fig. 1J left), MeRIP-seq detected one differentially modified m6A peak that contained two potential m6A modification sites suggested in the SRAMP database. The peak was visualized using IGV software (Supplementary Fig. 4E). To clarify which was indeed the m6A modification site in cLMNB1, wild-type and mutant plasmids were constructed as shown in Supplementary Fig. 4F. The same amount of both was used to transfect PC9OR cells in which cLMNB1 was stably silenced. RT-qPCR analysis indicated no difference in the expression levels of cLMNB1 among the experimental groups (Supplementary Fig. 4 G). The intracellular levels of m6A-modified cLMNB1 were markedly reduced when site 2 and both sites were mutated, whereas no change in m6A modification of cLMNB1 was observed when site 1 was mutated (Supplementary Fig. 4H).

We then studied the effect of cLMNB1 m6A modification levels on its function by constructing the m6A-modified cLMNB1 with site 2 mutation (cLMNB1-mut). In vitro, cLMNB1-mut induced apoptosis of PC9OR and HCC827OR cells, especially in the presence of osimertinib (Supplementary Fig. 4I–K). Furthermore, cLMNB1-mut inhibited the growth of osimertinib-resistant tumors in mice (Fig. 2K–M). Lower Ki-67 expression and a higher TUNEL positivity rate were found in tumors treated by DOX (cLMNB1-mut) and Osi + DOX (cLMNB1-mut), indicating decreased proliferation and increased apoptosis (Fig. 2N–P and Supplementary Fig. 4L). These results were validated, showing that cells stably transfected with TET-ON-cLMNB1-mut were only expressed when the mice were fed with DOX (Supplementary Fig. 4M). In summary, cLMNB1-mut participated in inhibiting osimertinib resistance in vitro and in vivo.

### METTL3 and YTHDF2 mediate cLMNB1 degradation

**Fig. 3:**
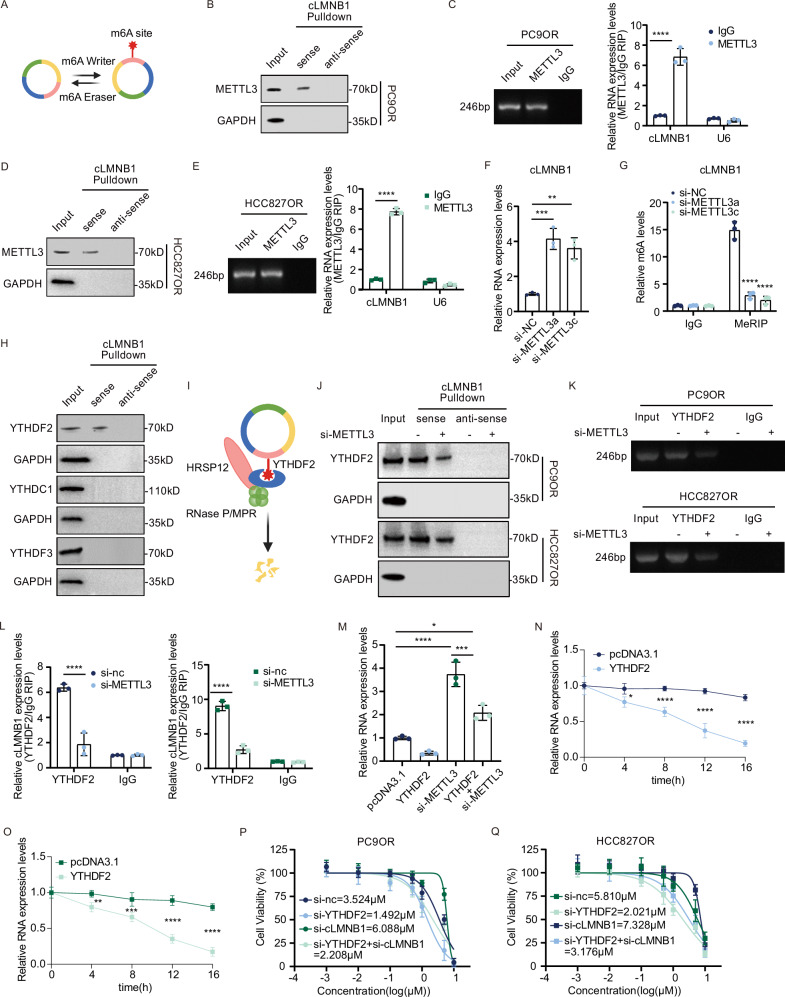
METTL3 and YTHDF2 mediate cLMNB1 degradation. **A** Schematic diagram showing the function of the m6A writer and m6A eraser. **B**–**E** Western blot of independent RNA pulldown assays verified the specific association of METTL3 protein with cLMNB1 using PC9OR (**B**) and HCC827OR (**D**) cells. RIP assay verifying the binding between METTL3 and cLMNB1 in PC9OR (**C**) and HCC827OR (**E**) cells. **F** The expression levels of cLMNB1 under METTL3 depression. **G** MeRIP analysis of m6A modification levels of cLMNB1 after METTL3 knockdown. **H** Western blot of the cLMNB1 pulldown assay indicated the potential and functional m6A reader, YTHDF2. **I** Schematic diagram showing the function of the YTHDF2 complex. **J** Western blot of independent RNA pulldown assays verified the specific association of YTHDF2 protein with cLMNB1 using PC9OR and HCC827OR cells with or without METTL3 knockdown. **K**, **L** RIP assay verifying the binding between YTHDF2 and cLMNB1, which was mediated by METTL3. **M** The expression levels of cLMNB1 under YTHDF2 overexpression with or without METTL3 knockdown. **N**, **O** Relative RNA levels of cLMNB1 after YTHDF2 overexpression detected by qPCR. **P**, **Q** PC9OR and HCC827OR cells were treated with osimertinib (Osi) at the indicated concentrations, when YTHDF2 and cLMNB1 were inhibited. IC50 was assessed after 3 days. Three independent experiments were conducted for each result. n.s. no significance, *p < 0.05, **p < 0.01, ***p < 0.001, ****p < 0.0001 compared with the controls.

The expression of the linear form of *LMNB1* mRNA in resistant cells was higher than in control ones (Supplementary Fig. 5A). The opposite trends of cLMNB1 and *LMNB1* expression aroused our interest. The main roles of m6A modification in circRNAs include biogenesis, cytoplasmic export, degradation, and translation [20–23]. Since the resistant and parental cells did not differ in nuclear-cytoplasmic distribution of cLMNB1 (Supplementary Fig. 5B), we hypothesized that m6A modifications could influence its expression. To further characterize the roles of m6A modifications in mediating cLMNB1, we identified the specific m6A writer and eraser responsible for the m6A modification, the function of which is briefly illustrated in Fig. 3A. We found that the mRNA expression of *METTL3*, the most common m6A writer, was significantly upregulated in PC9OR and HCC827OR cells (Supplementary Fig. 5C, D). Therefore, we speculated that METTL3 added the m6A modification to cLMNB1. The binding between cLMNB1 and METTL3 was verified by RNA pulldown following immunoblot and RNA immunoprecipitation (RIP) assays (Fig. 3B–E). We used siRNAs to downregulate *METTL3*, validating the efficacy of *METTL3* knockdown by western blot (Supplementary Fig. 5E). We selected si-METTL3a and si-METTL3c for subsequent investigations, revealing a significant impact of METTL3 reduction on cLMNB1 expression and m6A modification levels (Fig. 3F, G).

The m6A writers catalyze the addition of m6A modifications, while the reader proteins mediate the biological functions of the modified RNA [24]. YTHDF2, YTHDC1, and YTHDF3 are the usual m6A reader proteins mediating circRNAs [20–23]. Using a specific cLMNB1 probe, we found that YTHDF2 might be the reader modulating its biological function (Fig. 3H). YTHDF2 can facilitate the degradation of circRNAs by recruiting decay machinery or altering their interaction with other RNA-binding proteins and ribonucleases (Fig. 3I). The binding between cLMNB1 and YTHDF2 was verified by RNA pulldown following immunoblot and RIP assays. Their binding could be weakened by *METTL3* knockdown (Fig. 3J–L). YTHDF2 overexpression accelerated the degradation of cLMNB1. Nevertheless, coinstantaneous *METTL3* knockdown attenuated the degradation (Fig. 3M). Moreover, the overexpression of YTHDF2 markedly reduced the half-life of cLMNB1 (Fig. 3N, O). We then concurrently inhibited YTHDF2 and cLMNB1 to investigate the impact of m6A modification on cLMNB1-mediated osimertinib resistance. Our findings indicated that inhibition of YTHDF2 increased the susceptibility of resistant cells to osimertinib, a response that was negated by cLMNB1 suppression (Fig. 3P, Q). YTHDF2 overexpression and downregulation were validated by western blot (Supplementary Fig. 5F, G). These results demonstrated that the METTL3/YTHDF2 axis cooperatively promoted the degradation of cLMNB1 in LUAD-OR.

### FGFR4 is a potential downstream target of cLMNB1

**Fig. 4:**
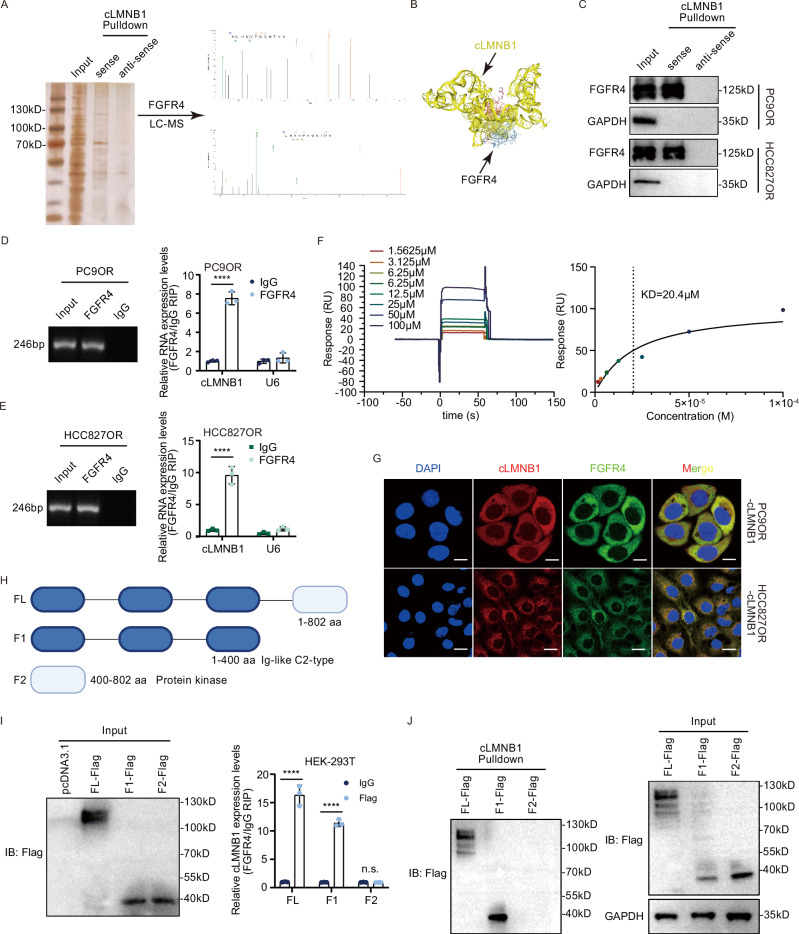
FGFR4 is a potential downstream target of cLMNB1. **A** cLMNB1 probe and control probes were biotinylated and incubated with PC9OR cell lysates for RNA pull-down assays. Photograph presenting silver staining for the proteins precipitated in the RNA pull-down assays (left). Two segments of FGFR4 proteins identified by mass spectrometry (right). **B** Graphical representation of the molecular docking between cLMNB1 and the FGFR4 protein using HDock. **C** Western blot of independent RNA pulldown assays verified the specific association of FGFR4 protein with cLMNB1 using PC9OR and HCC827OR cells. **D**, **E** RIP assays verifying the binding between FGFR4 and cLMNB1 in PC9OR and HCC827OR cells. **F** SPR analysis of binding of FGFR4 and cLMMB1. Shown are the binding curves measured at different concentrations of cLMNB1 and the steady-state affinity, indicated by the value of KD. **G** FISH and IF assays showing the colocalization of FGFR4 and cLMNB1. Scale bar, 20 μm. **H** Diagrams of full-length (FL) FGFR4 proteins and truncates with domain depletion. **I** Western blot analysis of FGFR4 with full-length and truncated forms in the lysates of the HEK-293T cells (left). RIP assay for cLMNB1 enrichment in cells transfected with flag-tagged FGFR4 (FL) overexpression vectors and truncated FGFR4 expression vectors. **J** Western blot of cLMNB1 pulldown assay in cells transfected with flag-tagged FGFR4 (FL) overexpression vectors and truncated FGFR4 expression vectors. Three independent experiments were conducted for each result. n.s. no significance, ****p < 0.0001 compared with the controls.

We performed RNA pulldown assays followed by electrophoresis, silver staining, and mass spectrometry (MS) to clarify the molecular mechanisms by which cLMNB1 influences osimertinib resistance. FGFR4, a protein recognized by MS for its crucial involvement in enhancing the malignant phenotype of cancer cells and affecting the tumor microenvironment, captured our attention and was pinpointed as a potential protein interacting with cLMNB1 (Fig. 4A). HDock predicted a potential physical interaction between cLMNB1 and FGFR4 protein (Fig. 4B). The binding between them was further validated by independent RNA pulldown and immunoblot assays (Fig. 4C). Additionally, RIP assays revealed marked enrichment of cLMNB1 when using FGFR4 antibodies (Fig. 4D, E). To further demonstrate that FGFR4 could specifically bind to cLMNB1 in vitro, we designed a surface plasmon resonance (SPR) assay, and the result indicated that FGFR4 could bind to cLMNB1 with a KD value of 20.4 μM (Fig. 4F). FISH combined with immunofluorescent (IF) assays demonstrated the colocalization of exogenous cLMNB1 and FGFR4 in the cytoplasm, confirming the direct interaction between them (Fig. 4G). We constructed truncated FGFR4 mutants to identify the domain on FGFR4 responsible for its association with cLMNB1 (Fig. 4H). RIP and RNA pulldown assays indicated that deleting the Ig-like C2-type domain at the N-terminus significantly abolished the interaction between cLMNB1 and FGFR4 (Fig. 4I, J). In conclusion, cLMNB1 interacted with FGFR4, a potential mediator of osimertinib resistance.

### cLMNB1 destabilizes FGFR4 via K48-linked polyubiquitination

**Fig. 5:**
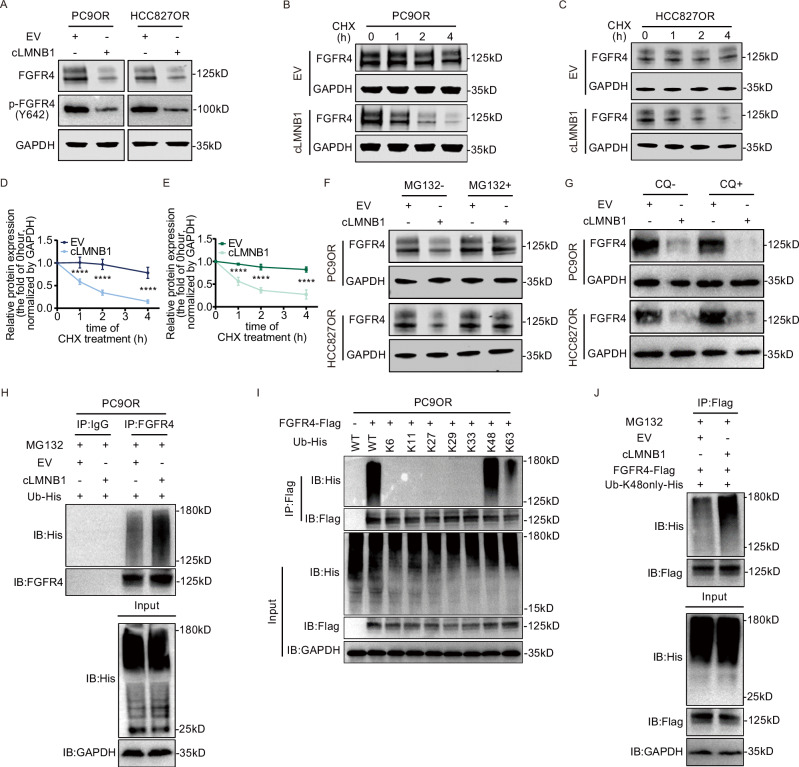
cLMNB1 destabilizes FGFR4 via K48-linked polyubiquitination. **A** Western blot for protein levels of FGFR4 and its phosphorylated form after cLMNB1 overexpression. **B**, **C** PC9OR and HCC827OR cells with cLMNB1 overexpression were treated with cycloheximide (CHX) for the indicated times. Western blot analysis of FGFR4 protein levels upon CHX treatment are presented, with the level at 0 h as a control. **D**, **E** The statistical analysis of (**B**, **C**) are shown. **F** Western blot analysis of FGFR4 protein levels regulated by cLMNB1 with or without MG132 treatment. **G** Western blot analysis of FGFR4 protein levels regulated by cLMNB1 with or without chloroquine (CQ) treatment. **H** Effects of cLMNB1 overexpression on the ubiquitination of FGFR4 proteins. PC9OR cells were transfected with cLMNB1 overexpression plasmids and His-tagged ubiquitin expression plasmids or the corresponding empty vectors. The cell lysates were incubated with FGFR4 or IgG antibodies and protein A/G magnetic beads. The proteins precipitated in the co-IP assay were analyzed by Western blot. IB immunoblot. **I** The ubiquitin linkage types of FGFR4 proteins were detected by Western blot. PC9OR cells were transfected with Flag-tagged FGFR4 overexpression plasmids and His-tagged ubiquitin expression plasmids retaining a unique lysine at a specific ubiquitin linkage site, respectively. **J** Western blot assay showing the upregulation of K48-linked ubiquitination with cLMNB1 overexpression in PC9OR cells. Ub-K48only-His: the cells were transfected with plasmids expressing His-tagged ubiquitin with all lysines mutated except K48. Three independent experiments were conducted for each result. ****p < 0.0001 compared with the controls.

We next investigated the impact of cLMNB1 on FGFR4 expression. FGFR4 and phosphorylated FGFR4 protein was highly expressed in PC9OR and HCC827OR cells (Supplementary Fig. 6A). The results showed that cLMNB1 overexpression did not change the mRNA level of *FGFR4* (Supplementary Fig. 6B) but significantly downregulated FGFR4 protein and its active form, which is phosphorylated at tyrosine 642 (Fig. 5A and Supplementary Fig. 6C). Cycloheximide treatment revealed that cLMNB1 overexpression shortened the half-life of FGFR4 protein in PC9OR and HCC827OR cells (Fig. 5B–E). Therefore, we hypothesized that cLMNB1 played an important role in FGFR4 degradation. In mammalian cells, the ubiquitin-proteasome system and autophagy-lysosome pathway participate in the degradation of most proteins [25]. To determine whether any of them mediated the influence of cLMNB1 on FGFR4 degradation, we treated cells with the proteasome inhibitor MG132 or the autophagic degradation pathway inhibitor chloroquine. We found that MG132, but not chloroquine, abolished the effect of cLMNB1 overexpression on FGFR4 protein levels (Fig. 5F, G and Supplementary Fig. 6D, E). Co-immunoprecipitation (co-IP) assays showed that the ubiquitination of endogenous FGFR4 was markedly upregulated by cLMNB1 overexpression (Fig. 5H). E3 ubiquitin ligases regulate protein polyubiquitination and, consequently, its function through various ubiquitin linkage types [26]. There are seven common types of ubiquitin linkages [27]. We constructed plasmids retaining a unique lysine at specific ubiquitin linkage sites and then transfected PC9OR cells with them. As shown in Fig. 5I, K48- and K63-linked polyubiquitin were the main types. K48-linked polyubiquitin is usually associated with substrate protein degradation, while K63-linked polyubiquitin is correlated with protein stabilization or activation [28]. We observed that cLMNB1 overexpression enhanced the K48-linked but not K63-linked polyubiquitination of FGFR4 (Fig. 5J and Supplementary Fig. 6F). These findings confirmed that cLMNB1 mediated FGFR4 degradation through the K48-linked ubiquitin-proteasome pathway. Furthermore, phosphoproteomic profiling of FGFR4 downstream effectors following cLMNB1 overexpression revealed significantly attenuated phosphorylation of STAT3 (Y705), AKT (S473), and ERK1/2 (T202/Y204) (Supplementary Fig. 6G).

Since the m6A modification could not affect the suppressive function of cLMNB1-mut, we hypothesized that the mutant could also mediate the ubiquitination of FGFR4. As shown in Supplementary Fig. 6H, I, cLMNB1-mut overexpression markedly degraded FGFR4 and phosphorylated FGFR4 and increased the ubiquitination of exogenous FGFR4. Moreover, YTHDF2 inhibition induced FGFR4 degradation, which was attenuated by cLMNB1 downregulation (Supplementary Fig. 6J). These results indicated that m6A modification might protect the FGFR4 protein from degradation by inhibiting cLMNB1.

### cLMNB1 promotes ubiquitination degradation of FGFR4 through c-Cbl

**Fig. 6:**
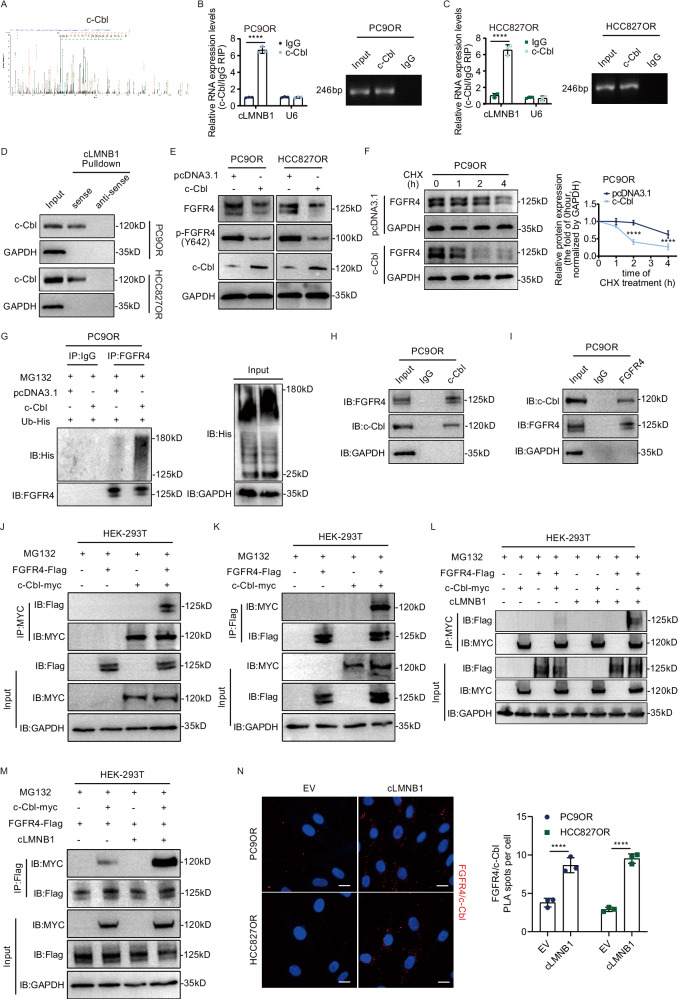
cLMNB1 promotes ubiquitination degradation of FGFR4 through c-Cbl. **A** The segment of c-Cbl proteins identified by mass spectrometry. **B**, **C** RIP assays verifying the binding between c-Cbl and cLMNB1 in PC9OR and HCC827OR cells. **D** Western blot of independent RNA pulldown assays verified the specific association of c-Cbl protein with cLMNB1 using PC9OR and HCC827OR cells. **E** Western blot for protein levels of FGFR4 and its phosphorylated form after c-Cbl overexpression. **F** PC9OR cells with c-Cbl overexpression were treated with cycloheximide (CHX) for the indicated times. Western blot analysis of FGFR4 protein levels upon CHX treatment are presented, with the level at 0 h as a control (left). The statistical analysis is shown on the right. **G** Effects of c-Cbl overexpression on the ubiquitination of FGFR4 proteins. **H**, **I** PC9OR cells were lysed, immunoprecipitated with anti-c-cBL, and then subjected to Western blotting assays using FGFR4 antibody (**H**), and vice versa (**I**). **J**, **K** HEK-293T cells transfected with FGFR4-Flag and c-Cbl-myc were lysed, immunoprecipitated with MYC-tag antibody or Flag-tag antibody, and subjected to Western blotting analysis. **L**, **M** HEK-293T cells were transfected with FGFR4-Flag, c-Cbl-myc, and cLMNB1 overexpression vectors. Co-IP assays validated that cLMNB1 increased the FGFR4-Flag level precipitated by c-Cbl-myc (**L**), and vice versa (**M**). MG132 was used to prevent the degradation of FGFR4. **N** Representative images of results obtained to investigate FGFR4 and c-Cbl interaction by Duolink in situ proximity ligation assay (PLA) assay in the indicated cells. Scale bars, 20 μm (left). Statistical analysis of average PLA spots per cell (right). Three independent experiments were conducted for each result. Three independent experiments were conducted for each result. ****p < 0.0001 compared with the controls.

We next sought to determine which E3 ligases were involved in the regulatory effect of cLMNB1 on FGFR4 degradation. The MS results detected several E3 ligases in the precipitation pulled down by cLMNB1 probes (Supplementary Table 5). E3 ubiquitin-protein ligase CBL (c-Cbl) was selected as the candidate E3 ligase of FGFR4 through bioinformatics analysis on the Ubibrowser platform and the above MS results. A secondary spectrogram of c-Cbl is shown in Fig. 6A. RIP assays and RNA pulldown confirmed that cLMNB1 binds to c-Cbl (Fig. 6B–D). CircRNAs can modulate the degradation process of downstream proteins by acting as a scaffold for that protein and its E3 ligase. Therefore, we hypothesized that cLMNB1 might function in this way. As expected, c-Cbl overexpression significantly downregulated FGFR4 and its phosphorylated form (Fig. 6E and Supplementary Fig. 7A), and c-Cbl knockdown upregulated FGFR4 expression (Supplementary Fig. 7B). c-Cbl overexpression efficiently shortened the half-life of FGFR4 (Fig. 6F and Supplementary Fig. 7C, D). Co-IP assays showed that cells with c-Cbl overexpression exhibited increased endogenous and exogenous FGFR4 ubiquitylation (Fig. 6G and Supplementary Fig. 7E), while c-Cbl inhibition induced the opposite result (Supplementary Fig. 7F). These results provided essential evidence that c-Cbl acted as an E3 ligase of FGFR4. We used co-IP assays to assess the interaction between c-Cbl and FGFR4, showing that endogenous FGFR4 was immunoprecipitated by c-Cbl and endogenous c-Cbl was immunoprecipitated by FGFR4 (Fig. 6H, I). Moreover, exogenous FGFR4 co-IP assays demonstrated that FGFR4-Flag could be easily detected in the anti-myc-c-Cbl immunoprecipitants and vice versa (Fig. 6J, K). The interaction between FGFR4 and c-Cbl could be markedly enhanced by cLMNB1 (Fig. 6L, M). Moreover, Duolink in situ proximity ligation assay (PLA) also confirmed the enhanced assembly of FGFR4 with c-Cbl after cLMNB1 overexpression (Fig. 6N). FISH combined with IF assays demonstrated the colocalization of exogenous cLMNB1 and endogenous c-Cbl and FGFR4 in the cytoplasm, further proving the involvement of cLMNB1 in the binding between c-Cbl and FGFR4 (Supplementary Fig. 7G). Therefore, cLMNB1 might act as a scaffold for the binding between FGFR4 and c-Cbl, leading to increased degradation of FGFR4.

### cLMNB1 increases the sensitivity of LUAD to osimertinib through FGFR4 inhibition

**Fig. 7:**
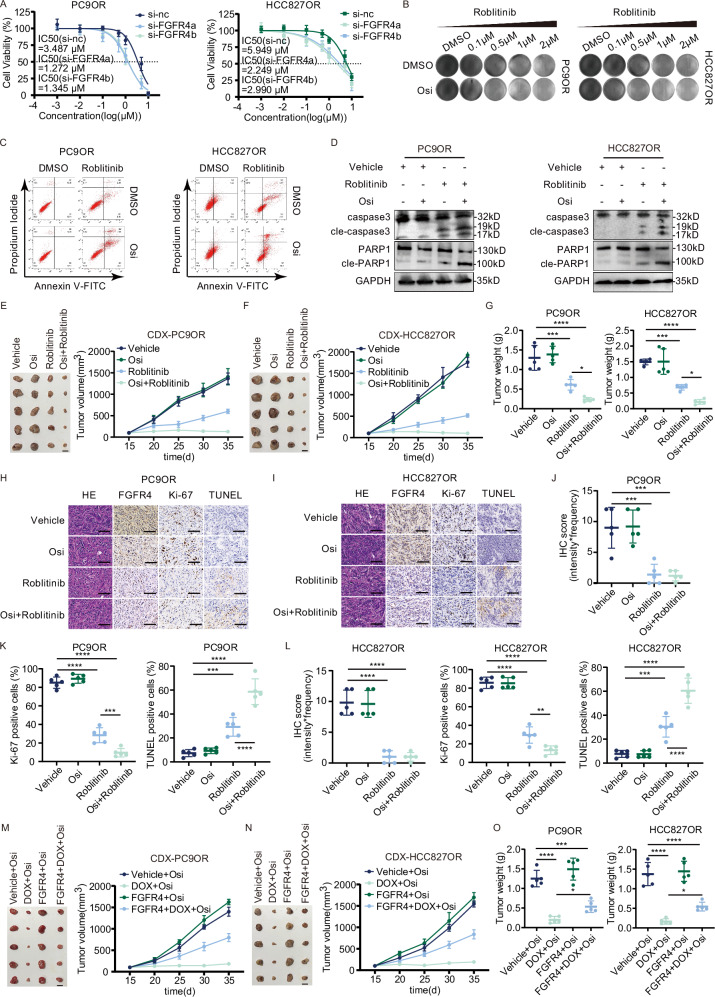
cLMNB1 increases the sensitivity of LUAD to osimertinib through FGFR4 inhibition. **A** PC9OR and HCC827OR cells with FGFR4 suppression were treated with osimertinib (Osi) at the indicated concentrations. IC50 was assessed after 3 days. **B** PC9OR and HCC827OR cells were treated with Roblitinib at the indicated concentrations, followed by crystal violet staining. **C** Apoptosis levels of PC9OR and HCC827OR cells treated with Osi or/and Roblitinib were analyzed by FACS. **D** The protein levels of caspase3, PARP1, and their cleavage forms of PC9OR and HCC827OR cells treated with Osi or/and Roblitinib were analyzed by Western blot. **E**, **F** Mice bearing PC9OR and HCC827OR cell xenograft tumors were treated with vehicle control, Osi, Roblitinb, and Osi+Roblitinb for 20 days, followed by treatment cessation and follow-up (*n* = 5). Scale bar, 10 mm. **G** Tumor weight of Vehicle, Osi, Roblitinib, and Osi + Roblitinib group at day 35 (*n* = 5). **H**, **I** Immunohistochemistry (H&E, FGFR4, Ki-67, and TUNEL stain, 40×) analysis of xenograft tumors from (**E**, **F**). Scale bar, 50 μm. **J**–**L** IHC score of FGFR4, cent Ki-67 positive cells, and per cent necrosis area of PC9OR and HCC827OR cell xenograft tumors from (**E**, **F**) are plotted. **M**, **N** PC9OR, and HCC827OR cells were stably transfected with DOX-ON-cLMNB1 and FGFR4 overexpression vectors. Mice bearing PC9OR and HCC827OR cell xenograft tumors were treated with Osi for 20 days, followed by treatment cessation and follow-up (*n* = 5). Scale bar, 10 mm. **O** Tumor weight of Vehicle + Osi, DOX + Osi, FGFR4 + Osi, FGFR4 + DOX+Osi group at day 35 (*n* = 5). Three independent experiments were conducted for each result. *p < 0.05, **p < 0.01, ***p < 0.001, ****p < 0.0001 compared with the controls.

We then sought to clarify the role of FGFR4 in mediating osimertinib resistance. It was observed that the sensitivity of PC9OR and HCC827OR cells to osimertinib was enhanced following the inhibition of FGFR4 using siRNAs (Fig. 7A). The efficiency of *FGFR4* knockdown and overexpression was confirmed by western blot (Supplementary Fig. 8A, B). Roblitinib, a specific tyrosine kinase inhibitor of FGFR4, showed promising results in preclinical studies and early-phase clinical trials. The roblitinib experimental concentrations were determined by the IC50 values (Supplementary Fig. 8C). The inhibition of FGFR4 by treatment of roblitinib for three days induced apoptosis of PC9OR and HCC827OR cells (Fig. 7B). Flow cytometry assays showed that apoptosis of PC9OR and HCC827OR cells treated with roblitinib or roblitinib with osimertinib for three days increased slightly in the first and extensively in the latter treatment (Fig. 7C and Supplementary Fig. 8D). The protein levels of cleaved caspase-3 and PARP1 detected by western blot further substantiated that apoptosis was induced by FGFR4 inhibition (Fig. 7D). In conclusion, FGFR4 inhibition could partially alleviate the resistance of LUAD to osimertinib in vitro.

We then tested the therapeutic effect of FGFR4 after tumor formation in vivo. PC9OR and HCC827OR cells were transplanted subcutaneously into BALB/c-nude mice. After developing palpable tumors, the mice were randomly assigned to four groups. The time for tumors to reach 0.5 cm^3^ in the Roblitinib and Osi + Roblitinib groups was significantly longer than in the Vehicle and Osi groups. It indicated that tumor proliferation in vivo was significantly attenuated by roblitinib alone or with osimertinib (Fig. 7E–G). IHC staining of the tumors showed lower FGFR4 and Ki-67 expression and a higher TUNEL positivity rate in the Roblitinib and Osi + Roblitinib groups than the Vehicle and Osi groups, indicating decreased proliferation and increased apoptosis (Fig. 7H–L). These results proved that FGFR4 inhibition could overcome osimertinib resistance in vivo.

We then explored whether the osimertinib resistance suppressed by cLMNB1 depended on FGFR4. We demonstrated that FGFR4 overexpression attenuated the apoptosis of resistant cells induced by cLMNB1 and cLMNB1-mut (Supplementary Fig. 8E–H). PC9OR and HCC827OR cells stably expressing TET-ON-cLMNB1 were transfected with control or FGFR4 overexpression lentiviruses. Cells with TET-ON-cLMNB1 or TET-ON-cLMNB1 plus FGFR4 were transplanted subcutaneously into ten BALB/c-nude mice. After developing palpable tumors, the mice were randomly assigned to two groups, respectively. All mice were treated with osimertinib. The DOX + Osi group showed the largest tumor regression extent, while high FGFR4 expression counteracted this effect (Fig. 7M–O). Based on these results, cLMNB1 increased the sensitivity of LUAD to osimertinib by inhibiting the FGFR4 protein.

### The cLMNB1-mut encapsulated in LNPs is promising to overcome osimertinib resistance

**Fig. 8:**
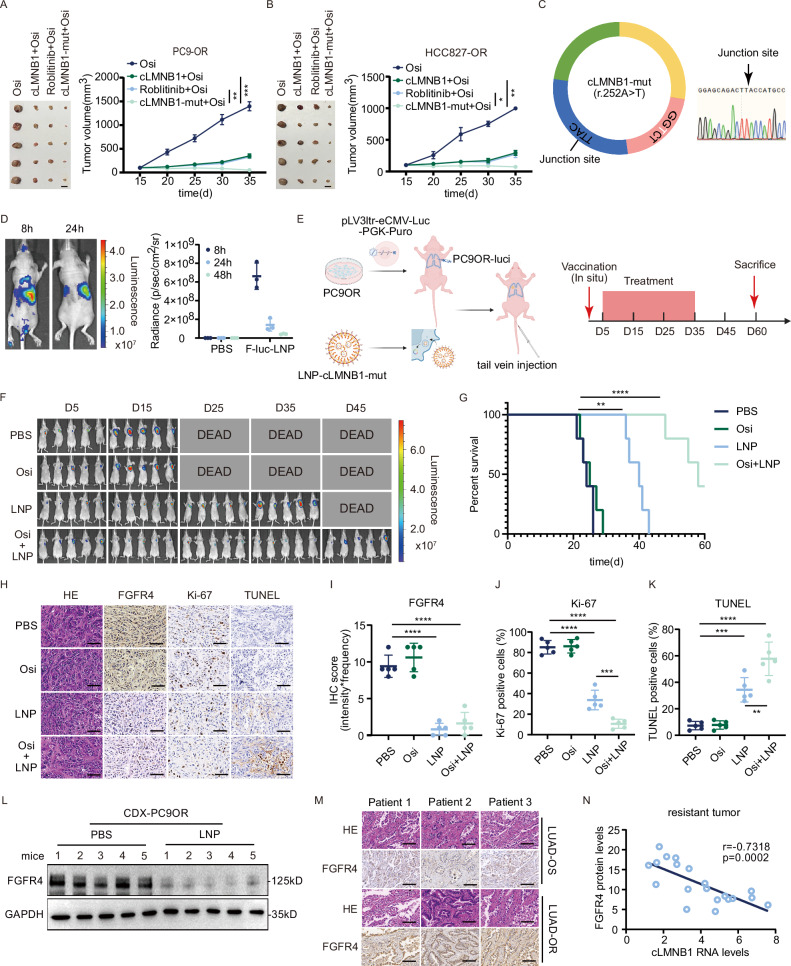
The cLMNB1-mut encapsulated in LNPs is promising to overcome osimertinib resistance. **A**, **B** PC9OR and HCC827OR cells were stably transfected with DOX-ON-cLMNB1, DOX-ON-cLMNB1-mut, or the corresponding empty vectors. Mice bearing PC9OR and HCC827OR cell xenograft tumors were treated with Osi for 20 days (*n* = 5). **C** Schematic diagram of cLMNB1-mut (left). The junction sequence was verified by Sanger sequencing (right). **D** Bioluminescence imaging assay of mice at 8 h and 24 h after injection of F-luc-LNP through the tail vein. **E** Mouse lungs were orthotopically transplanted with PC9OR-luci cells, and then mice were given PBS, Osi, LNP, Osi + LNP, followed by treatment cessation and follow-up (*n* = 5). **F** Bioluminescence imaging of nude mice every 10 days. **G** Kaplan–Meier survival analysis of nude mice. **H** Hematoxylin-eosin (H&E, 40×) and Immunohistochemistry (FGFR4, Ki-67, and TUNEL stain, 40×) analysis of mouse lungs. Scale bar, 50 μm. **I**–**K** IHC score of FGFR4, per cent Ki-67 positive cells, and per cent area necrosis were plotted. **L** Western blot for protein levels of FGFR4 in the mouse tumors treated with PBS and LNP (*n* = 5). **M** H&E (40×) and Immunohistochemistry (FGFR4 stain, 40×) analysis of sensitive and resistant patient lungs. Scale bar, 50 μm. **N** Pearson’s correlation between FGFR4 protein and cLMNB1 RNA expression in our osimertinib resistance LUAD sample cohort (*n* = 20). Three independent experiments were conducted for each result. *p < 0.05, **p < 0.01, ***p < 0.001, ****p < 0.0001 compared with the controls.

Since cLMNB1 could be degraded in an m6A-dependent manner, we hypothesized that cLMB1-mut could enhance the therapeutic effect of osimertinib in LUAD-OR. Moreover, the results above suggested that cLMNB1-mut induced a high apoptosis rate in resistant cells in vitro and in vivo (Fig. 2K–M and Supplementary Fig. 4H–J). Therefore, we transfected cells with empty vector, TET-ON-cLMNB1, or TET-ON-cLMNB1-mut and transplanted them subcutaneously into BALB/c-nude mice. The four transplanted groups are depicted in Fig. 8A, B. All mice were treated with osimertinib. As shown in Fig. 8A, B, tumor growth was significantly inhibited in the cLMNB1-mut overexpression group compared to the cLMNB1 overexpression and roblitinib groups.

mRNA vaccines are a key technology in combating existing and emerging infectious diseases. Recently, highly stable circRNA encapsulated in LNPs showed therapeutic potential in various diseases. We developed exogenous cLMNB1-mut, the structure of which is shown in Fig. 8C. It was validated by Sanger-seq and shown to have high integrity and purity and fragment size in line with expectations (Supplementary Fig. 9A). The cLMNB1-mut was more resistant to RNase R degradation than the linear form of *LMNB1* mRNA (Supplementary Fig. 9B). Consequently, we produced LNPs-cLMNB1-mut suspended in PBS whose average particle size based on dynamic light scattering was 87.35 nm. A narrow polydispersity index of 0.1555 indicated a homogeneous LNP distribution. We transfected PC9OR and HCC827OR cells with LNPs-GFP, ensuring they could enter the cells by fluorescence microscopy (Supplementary Fig. 9C). Western blot assays demonstrated that cells transfected with LNPs-cLMNB1-mut had lower FGFR4 protein expression than cells transfected with LNPs-EV (Supplementary Fig. 9D). We injected F-luc-LNPs into BALB/c-nude mice through the tail vein. Bioluminescence imaging assays indicated that LNPs accumulated in several organs and were gradually metabolized, mainly in the liver (Fig. 8D).

We then implanted luciferase-labeled PC9OR cells in situ into the lungs of nude mice and confirmed the inoculation by bioluminescence imaging after surgery. These mice were randomly assigned into four groups and treated as indicated, measuring their tumor size by bioluminescence imaging every ten days (Fig. 8E). The results revealed that mice treated with Osi + LNP had the lowest tumor burden and the longest overall survival (Fig. 8F, G). IHC assays of the lungs of these mice showed low FGFR4 and Ki-67 expression and a high TUNEL positivity rate, indicating decreased proliferation and increased apoptosis (Fig. 8H–K). Moreover, we extracted proteins from the mouse lung tumors and confirmed that LNPs-cLMNB1-mut degraded FGFR4 protein expression in vivo (Fig. 8L). Hematoxylin-eosin staining of vital organs of mice treated with LNPs-cLMNB1-mut showed no apparent organ toxicity (Supplementary Fig. 9E). There were no significant differences in body weight among the four groups, indicating that the LNPs were safe and reliable (Supplementary Fig. 9F). Overall, LNPs-cLMNB1-mut overcame osimertinib resistance by degrading the FGFR4 protein in vivo.

We further verified the clinical association between cLMNB1 and FGFR4. In our patient cohort, high FGFR4 protein expression levels were confirmed by IHC assays in LUAD-OR patients (Fig. 8M). A negative correlation was found between the expression of FGFR4 protein and cLMNB1 in 20 LUAD-OR patients (Fig. 8N).

## Discussion

This study found a circRNA, cLMNB1, with low expression and high m6A modification levels in LUAD-OR. METTL3 catalyzed the m6A modifications of cLMNB1, while YTHDF2 identified the m6A modification and induced RNA degradation. cLMNB1 acted as a scaffold, increasing the interaction between FGFR4 and c-Cbl and leading to FGFR4 degradation in a ubiquitin-dependent way. This process was inhibited in LUAD-OR due to the low cLMNB1 expression. Given that cLMNB1 degraded in an m6A-dependent manner, we constructed the cLMNB1-mut, which was stable against such degradation. Encapsulated in LNPs, cLMNB1-mut overcame osimertinib resistance when delivered in resistant cells.

With the advent of COVID-19 mRNA vaccines, LNP drug delivery systems started being used to treat various diseases. Benefiting from its excellent stability and low immunogenicity, circRNA is expected to become a nucleic acid drug superior to mRNA. Various LNPs have been developed, offering drug delivery systems with reduced toxicity, enhanced cellular affinity, and improved organ targeting [29]. Given the biological process wherein cLMNB1 is degraded through m6A modifications, we constructed cLMNB1-mut to delay degradation and achieve improved therapeutic efficacy. This suggests that we can utilize RNA modifications to design synthetic circRNA and improve efficacy. Developing innovative LNPs, including lung-targeted LNPs and those designed for administration via airway inhalation, could potentially help treat lung cancer [30].

In recent years, great breakthroughs have been made in lung cancer treatment [31]. However, many treatment strategies are less effective than expected. The main reason for these unsatisfactory results is the emergence of therapeutic resistance [32]. m6A modifications can mediate several biological processes of circRNAs. Since both circRNAs and m6A modifications are key modulators in cancer therapeutic resistance, m6A-modified circRNAs are gaining increasing attention in cancer treatment resistance. Exosomal transmission of circRNA-SORE led to upregulation in sorafenib-resistant HCC cells. This upregulation attenuated PRP19-induced YBX1 degradation by binding to YBX1, thereby decreasing the therapeutic effectiveness of sorafenib [33]. It was demonstrated that hsa_circ_0005576 could increase the mRNA expression of insulin-like growth factor 1 receptor (*IGF1R*) by acting as a sponge for miR-512-5p in LUAD-OR cells [34]. Our research further enriches the study of m6A-modified circRNAs in LUAD-OR.

Osimertinib has been recommended as a first-line treatment in patients with the T790M EGFR mutation because of its efficacy in treating brain metastases and resistant tumors due to the mutation. However, osimertinib resistance is a non-negligible problem in LUAD therapy. Current research summarizes the development of osimertinib resistance through four routes: EGFR-dependent, EGFR-independent, epithelial-to-mesenchymal transition, and small cell lung cancer (SCLC) transformation [5]. This study found FGFR4 protein upregulation in LUAD-OR, a possible alternative pathway to maintain the resistance of treated LUAD. FGFR4 is widely known as a star target in HCC and gastric carcinoma. It was demonstrated that FGFR inhibition by lenvatinib leads to feedback activation of the EGFR-PAK2-ERK5 signaling axis, which is blocked by EGFR inhibition [15]. The treatment of 12 patients with advanced HCC who were unresponsive to lenvatinib with a combination of lenvatinib and gefitinib (trial identifier NCT04642547) resulted in meaningful clinical responses. The NCT02325739 trial led by Novartis Pharmaceuticals was an open-label study of oral FGF401 (roblitinib) in adult patients with HCC or solid malignancies characterized by positive FGFR4 and KLB expression [35]. The efficacy and safety of erdafitinib, a pan-FGFR inhibitor, are now being evaluated in patients with advanced NSCLC after relapse of standard therapy [36]. The FGFR and EGFR families always function as complementary signaling pathways in tumors, suggesting that the FGFR family could be a promising therapeutic target following EGFR-TKI resistance.

The main limitation of this study is the lack of explanation of the FGFR4 downstream events. It is well known that FGFR4 activates the mitogen-activated protein kinase and SRC homology 2 domain-containing phosphatase 2 signaling pathways, thereby maintaining the malignant phenotype in cancer cells [37, 38]. Furthermore, the fibroblast growth factor 19-FGFR4-dependent signaling has been reported as a critical pathway in the development of HCC [39]. However, the autocrine or paracrine ligands that FGFR4 binds to in LUAD remain to be elucidated. The crosstalk between the tumor microenvironment and the FGFR4-positive tumor cells merits further investigation.

## Materials and methods

### Tissue samples

Human tissues were collected from the Nantong Cancer Hospital respiratory Department. The LUAD-OS tissues were obtained from treatment-naïve patients with EGFR mutations. The LUAD-OR tissues were collected from osimertinib-treated patients who were diagnosed with recurrence by computerized tomography. The clinical characteristics of the patients are detailed in Supplementary Table 1. The study protocol was reviewed and approved by the Nantong Cancer Hospital research ethics committee (Ethics review approval number: 2023-038) and conformed to the ethical standards for medical research involving human subjects, as laid out in the 1964 Declaration of Helsinki and its later amendments. Participants provided written informed consent prior to taking part in the study.

### Cell lines

The HEK-293T cell line and human NSCLC cell lines PC9 and HCC827 cells were purchased from the China Center for Type Culture Collection. PC9 and HCC827 cells were cultured in 1640 with 10% FBS (Corning). HEK-293T cells were cultured in DMEM with 10% FBS (Corning). Before the experiment, cells were tested for mycoplasma, cross-contamination between species, and authenticity. Cell lines in the experiments were used within 20 passages.

### Animal models

The Animal Care and Use Committee (IACUC) of Nanjing Medical University authorized all mouse research (IACUC-2304023-1). The IACUC allows a maximum tumor size of 2 cm; none of the trials exceeded this restriction. For animal research, four-week-old female BALB/c nude mice were purchased from GemPharmatech. All animals were housed at Nanjing Medical University in a pathogen-free environment. All animals were subjected to a 12-h light-dark cycle. The room temperature was kept at 22 °C, and the humidity level was kept between 55% and 70%.

For the subcutaneous xenograft model in Supplementary Fig. 1C, subcutaneous injections of 5 × 10^6^ PC9, HCC827, PC9OR, or HCC827OR cells with 50% Matrigel (Corning) were performed on each nude mouse. For efficacy studies, when the xenografts reached approximately 100 mm^3^, the mice were treated with osimertinib (10 mg/kg once daily) (*n* = 5 mice per group). All the mice were sacrificed after the treatment, and then the tumors were removed for further studies.

For the subcutaneous xenograft model in Fig. 2, subcutaneous injections of 5 × 10^6^ PC9OR or HCC827OR cells (stably transfected with TET-ON-cLMNB1 or TET-ON-cLMNB1-mut) with 50% Matrigel (Corning) were performed on each nude mouse. For efficacy studies, when the xenografts reached approximately 100 mm^3^, the mice were randomized into 4 groups (*n* = 5 mice per group): (1) Vehicle control, (2) Osi (10 mg/kg once daily), (3) Dox (100 mg/kg once daily), and (4) Osi (10 mg/kg once daily) + Dox (100 mg/kg once daily). All the mice were treated as described and sacrificed after the treatment, and then the tumors were removed for further studies.

For the subcutaneous xenograft model in Figs. 7H, I, subcutaneous injections of 5 × 10^6^ PC9OR or HCC827OR cells with 50% Matrigel (Corning) were performed on each nude mouse. For efficacy studies, when the xenografts reached approximately 100 mm^3^, the mice were randomized into 4 groups (*n* = 5 mice per group): (1) Vehicle, (2) Osi (10 mg/kg once daily), (3) Roblitinib (10 mg/kg once daily), and (4) Osi (10 mg/kg once daily) + Roblitinib (10 mg/kg once daily). All the mice were treated as described and sacrificed after the treatment, and then the tumors were removed for further studies.

For the subcutaneous xenograft model in Fig. 7P, Q, subcutaneous injections of 5 × 10^6^ PC9OR or HCC827OR cells (stably transfected with TET-ON-cLMNB1 or/and FGFR4) with 50% Matrigel (Corning) were performed on each nude mouse. For efficacy studies, when the xenografts reached approximately 100 mm^3^, the mice stably with TET-ON-cLMNB1 were randomized into 2 groups (*n* = 5 mice per group): (1) Vehicle, (2) Dox (100 mg/kg once daily). The mice stably with TET-ON-cLMNB1 and FGFR4 were randomized into another 2 groups (*n* = 5 mice per group): (1) FGFR4, (2) FGFR4 + Dox (100 mg/kg once daily). All the mice were treated with osimertinib (10 mg/kg once daily) and sacrificed after the treatment, and then the tumors were removed for further studies.

For the orthotopic xenograft mouse model in Fig. 8, PC9OR-luci cells were intrapulmonary injected into the mice. After injection for 5 days, lung orthotopic xenografts were assessed by bioluminescence imaging (PerkinElmer). Mice were randomly divided into 4 groups (*n* = 5 mice per group) according to the intensity of the region of interest (ROI): (1) PBS control, (2) Osi (10 mg/kg once daily), (3) LNP (0.3 mg/kg once daily), (4) Osi (10 mg/kg once daily) + LNP (0.3 mg/kg once daily). Thirty days later, all treatment was withdrawn. Lung tumors were recorded every 10 days by bioluminescence imaging. After 55 days, the mice were sacrificed, and their lungs were extracted, weighed, and photographed. For HE and IHC investigations, the lung tissue was fixed in 4% paraformaldehyde.

Detailed methods are provided in Supplementary Data 2.

### Statistics and reproducibility

All data are based on independent experiments with independent biological replicates. No statistical methods were used to predetermine the sample size. Most results were qualitatively replicated in two different cell lines and provided consistent results using independent techniques. Investigators were not blinded to the assignment. No samples or experiments were excluded during the experiment and outcome assessment. Experimental data are expressed as mean and individual points, mean and standard deviation, or mean and range, as shown in the figure legend. Immunofluorescence experiments were performed in at least three independent biological replicates, and for each replicate, several hundred cells were scored in each condition. Cytostatic assays were performed on at least three independent biological replicates, and statistical significance was determined using a two-way ANOVA (Prism v.8.0). It was tested for significance using an unpaired two-tailed Student’s *t*-test for multiple comparisons with a single control. The tests are performed under the assumption that the values follow a normal distribution and have similar variances. Statistical analysis was performed using Prism v.8.0.

## Supplementary information

Supplementary information

Uncropped blots and gels

## Data Availability

MeRIP-seq data have been deposited in the Gene Expression Omnibus (ncbi.nlm.nih.gov/geo) under accession number GSE272182. The raw data of circRNA-seq reported in this paper have been deposited in the Genome Sequence Archive in the National Genomics Data Center, China National Center for Bioinformation / Beijing Institute of Genomics, Chinese Academy of Sciences (GSA-Human: HRA007900), which are publicly accessible at https://ngdc.cncb.ac.cn/gsa-human. This document includes source data. All further data supporting the conclusions of this study are accessible upon reasonable request from the corresponding author.
