# Supplementary material for: The N6-methyladenosine-mediated cLMNB1 degrades FGFR4 to overcome osimertinib resistance in non-small cell lung cancer
Source: Cell Death Dis. 2025 Nov 10;16(1):818. doi: 10.1038/s41419-025-08124-8 (PMC12603240; doi:10.1038/s41419-025-08124-8)
Supplement: Supplementary file 1 — Supplementary information [file 41419_2025_8124_MOESM1_ESM.pdf]

>hsa_circ_0007726|NM_005573|LMNB1
CTATGCTAAGAAGGAATCTGATCTTAATGGCGCCCAGATCAAGCTTCGAGAATATGAAG
CAGCACTGAATTCGAAAGATGCAGCTCTTGCTACTGCACTTGGTGACAAAAAAAGTTT
AGAGGGAGATTTGGAGGATCTGAAGGATCAGATTGCCCAGTTGGAAGCCTCCTTAGCT
GCAGCCAAAAAACAGTTAGCAGATGAAACTTTACTTAAAGTAGATTTGGAGAATCGTT
GTCAGAGCCTTACTGAGGACTTGGAGTTTCGCAAAAGCATGTATGAAGAGGAGATTAA
CGAGACCAGAAGGAAGCATGAAACGCGCTTGGTAGAGGTGGATTCTGGGCGTCAAAT
TGAGTATGAGTACAAGCTGGCGCAAGCCCTTCATGAGATGAGAGAGCAACATGATGCC
CAAGTGAGGCTGTATAAGGAGGAGCTGGAGCAGACTTACCATGCCAAACTTGAGAATG
CCAGACTGTCATCAGAGATGAATACTTCTACTGTCAACAGTGCCAGGGAAGAACTGAT
GGAAAGCCGCATGAGAATTGAGAGCCTTTCATCCCAGCTTTCTAATCTACAGAAAGAG

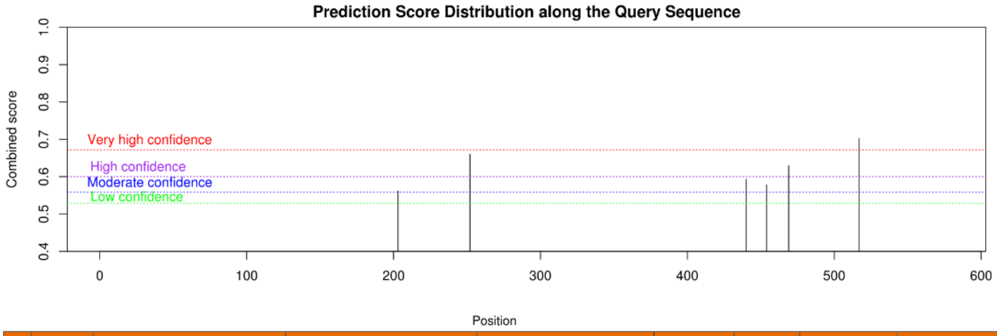

#	Position	Sequence context	Structural context	Local structure visualization	Score(binary)	Score(knn)	Score(spectrum)	Score(combined)	Decision
1	203	CAAAA AACAG UUAGC<br>AGAUG AAACU UUACU<br>UAAAG UAGAU UUGGA	N/A	N/A	0.481	0.499	0.682	0.562	m6A site (**Moderate** confidence)
2	252	CGUUG UCAGA GCCUU<br>ACUGA GGACU UGGAG<br>UUUCG CAAAA GCAUG	N/A	N/A	0.716	0.681	0.581	0.660	m6A site (**High** confidence)
3	440	GUAUA AGGAG GAGCU<br>GGAGC AGACU UACCA<br>UGCCA AACUU GAGAA	N/A	N/A	0.574	0.611	0.617	0.593	m6A site (**Moderate** confidence)
4	454	UGGAG CAGAC UUACC<br>AUGCC AAACU UGAGA<br>AUGCC AGACU GUCAU	N/A	N/A	0.511	0.488	0.682	0.578	m6A site (**Moderate** confidence)
5	469	AUGCC AAACU UGAGA<br>AUGCC AGACU GUCAU<br>CAGAG AUGAA UACUU	N/A	N/A	0.599	0.529	0.684	0.629	m6A site (**High** confidence)
6	517	CUGUC AACAG UGCCA<br>GGGAA GAACU GAUGG<br>AAAGC CGCAU GAGAA	N/A	N/A	0.710	0.559	0.709	0.702	m6A site (**Very high** confidence)

diffreps_peak_593341 of MeRIP-seq

XXXXX predicted position of SRAMP

Detailed methods

Compounds. All compounds used in this study were listed in Supplementary Table 2.

Lentivirus, plasmids and siRNAs. Lentivirus vectors expressing cLMNB1 or FGFR4 were purchased from Nanjing Corues Biotechnology. PC9OR and HCC827OR cells were infected with lentiviruses in the presence of 2 μg/ml polybrene (MedChemExpress). TET-ON-cLMNB1 or TET-ON-cLMNB1-mut expression plasmid was created by cloning the cLMNB1 or cLMNB1-mut sequence using pHBLV-TetOn-SV40-Puro-TRE3GS-MCS. Subsequently, PC9OR and HCC827OR cells stably overexpressing TET-ON-cLMNB1 or TET-ON-cLMNB1-mut were constructed using the same method as above. Plasmids overexpressing cLMNB1, cLMNB1-mut1, cLMNB1-mut2, cLMNB1-mut3, cBRCA1, YTHDF2, Flag-FGFR4, myc-c-Cbl, His-Ub, His-Ub-K6, His-Ub-K11, His-Ub-K27, His-Ub-K29, His-Ub-K33, His-Ub-K48, His-Ub-K63 were purchased from Nanjing Corues Biotechnology. All plasmids were verified by DNA sequencing. Lipofectamine 3000 was used to transfect plasmids according to the manufacturer's instructions. Nanjing Corues Biotechnology provided the siRNAs targeting cLMNB1, METTL3, YTHDF2, FGFR4 and c-Cbl. Lipofectamine RNAiMAX (Thermo) was used for siRNA transfections.

Cell Counting Kit-8 (CCK-8) and colony formation assay. Drug sensitivity was assessed by CCK-8 assay. Briefly, resistant LUAD cells were seeded into 96-well cell culture plates at a density of 5,000 cells/well for IC50 (half-maximal inhibitory concentration) assessment. For IC50 assay, cells were treated with indicated drugs or DMSO as control after cell attachment and incubated for indicated time. Then, 10 μl of CCK-8 was added to the cells. After incubation for another 2 hours at 37°C. The absorbance values were measured by a microplate reader (Bio-Rad, CA, USA) at 450 nm.

Colony formation assay was also performed for assessment of IC50. 5 × 10^4 resistant LUAD cells were inoculated into 12-well cell culture plates. After drug incubation for 3 days, the cells were washed with PBS, fixed by 4% formaldehyde for 5 min and stained with 1% crystal violet for 15 min.

Cell growth and viability assays. The real-time cell analysis (RTCA) system (ACEA Biosciences) was used to monitor cell growth using cell proliferation plates. Following the addition of RPMI-1640 containing 10% FBS to the chamber, cells (10,000) were plated onto each well of the E-plate. The drug was administered after the cells being completely adherent to the wall. Readings were taken every hour until the experiment ended (up to 60 hours).

PI/Annexin V apoptosis assay. All cells, both floating and adherent, were trypsinized and rinsed with PBS after the indicated treatments. Annexin V-FITC Apoptosis Detection Kit I (BD Biosciences) was used to detect apoptotic cells by staining with Annexin V-FITC and PI according to the manufacturer's instructions. A flow cytometer (BD Biosciences) was used to analyse the apoptosis of the cells.

RNA extraction and qRT-PCR. Patient tissue and cell RNA were extracted using the TRIzol reagent (Thermo) followed by reverse transcription using the RNA-to-cDNA kit (Takara). To quantitatively express the contents of RNA, qRT-PCR analysis was performed using SYBR Green Premix (Vazyme) in the QuantStudio 6 Flex system (ABI) or ViiA 7 Dx system (ABI). β-actin, GAPDH or U6 served as the internal control for circRNA and mRNA, and qRT-PCR primer sequences are listed in Supplementary Table 3.

MeRIP-qPCR. m6A modifications were determined using MeRIP m6A Kit (Millipore) according to the manufacturer’s instructions. Briefly, 100 µg of total RNA was sheared to an approximate

length of 100 nt using metal-ion-induced fragmentation. Before immunoprecipitation, one-tenth of fragmented RNA was saved as input control. The RNA fragments were then incubated with anti-m6A antibody-conjugated (202003, Synaptic Systems) or rabbit IgG-conjugated (Millipore) protein A/G magnetic beads in 1×immunoprecipitation buffer, supplemented with RNase inhibitors, at 4°C for 2 h on a rotating wheel. After three washes, the bound RNA was eluted through competition with free N6-methyladenosine, and then purified using the RNeasy mini kit (QIAGEN). Input and immunoprecipitated m6A RNAs were reverse transcribed using a PrimeScript RT reagent kit (Takara), and the RNAs were further analyzed by qRT-PCR with the primers listed in Supplementary Table 3.

m6A dot blot. The m6A dot blot assay was performed as previously described.[40] The total RNA samples were loaded to Hybond-N + membrane (GE Healthcare, UK) and UV crossed with the nylon membrane. The membrane was then blocked with 5% nonfat milk for 1 hour and incubated with m6A antibody (AF7407, Beyotime, China) at 4°C, overnight. After incubating with horseradish peroxidase-conjugated anti-mouse IgG, the membrane was visualized with the ECL detection system. The same amount of total RNA samples was spotted on the membrane and stained with 0.02% methylene blue (MB) in 0.3 M sodium acetate (pH = 5.2). The results of m6A level were shown in the form of relative density normalized to methylene blue staining density of the m6A dot blot.

m6A Quantification. The change of global m6A levels in total RNA was measured by EpiQuik m6A RNA Methylation Quantification Kit (Colorimetric) (Epigentek) following the manufacturer’s protocol.

MeRIP-Seq and Data Analysis. Total RNA of PC9OR and PC9 cells was prepared as

previously described.[41] The m6A Methylated RNA Immunoprecipitation sequencing (MeRIP-seq) was performed by Cloudseq Biotech, Inc. (Shanghai, China), according to published procedures.[41] Differentially methylated peaks of circRNAs are listed in Supplementary Table 4.

CircRNA-seq. Osimertinib-resistant samples of puncturing lung biopsy and treatment-naïve samples were collected. The total RNA was extracted using TRIzol reagent. Subsequently, the concentration and integrity of the extracted total RNA were determined using the Qubit 3.0 Fluorometer (Invitrogen) and the Agilent 2100 Bioanalyzer (ABI). Total RNA was treated with 10 units of RNase R (Genesee) for 30 minutes at 37 °C. The quality and concentration of the library were determined using a DNA 1000 chip on an Agilent 2100 Bioanalyzer. The KAPA Biosystems Library Quantification kit (Kapa Biosystems) was used to assess the accuracy of quantification for sequencing applications. Before clustering, each library was diluted to a final concentration of 10 nM and pooled equimolarly. All samples were subjected to Paired-End (Nova-seq 6000) sequencing. The reads were first mapped to the most recent using Bowtie2 version 2.1.0[47]. UCSC transcript collection STAR[48] and DCC[49] STAR were used to map the data to the mapped genome for circRNA expression investigation. The edgeR tool was used to identify differentially expressed genes. The figure was created using R. Differentially expressed circRNAs are listed in Supplementary Table 4.

RNase R treatment. Total RNA was isolated from PC9OR and HCC827OR cells and separated into two groups. After pre-treatment with RNase R (Genesee) at 37 °C for 30 minutes according to the manufacturer's recommendations, qRT-PCR was performed to determine the expression of cLMNB1, LMNB1, and GAPDH with or without RNase R. GelRed was used to visualize the products after they were separated on a 1.5% agarose gel.

Nuclear and cytoplasmic extraction assay. According to the manufacturer's procedure, a nucleocytoplasmic separation kit (Thermo) was used to extract nucleocytoplasmic protein or RNA. The nucleus and cytoplasmic RNAs were used to detect the expression of target genes by qRT-PCR. β-actin as the cell plasma localization reference and U6 as the nuclear localization reference was applied to normalise the target gene expression and calculate the ratio of the nucleus to the cytoplasm.

RNA fluorescence in situ hybridization (FISH). A cy3-labelled oligonucleotide probe complementary to the cLMNB1 junction region was designed using the Clone Manager Kit analysis tool. PC9, HCC827, PC9OR and HCC827OR cells were seeded in covered glass-bottom confocal dishes and cultured overnight. In situ staining was performed using the RNA FISH kit (GenePharma) following the manufacturer's instructions. Nuclei were stained using DAPI. Images were acquired on an LSM 880 laser confocal microscope (Carl Zeiss). The sequence of the cLMNB1 probe is CAGATTCCTTCTTAGCATAGCTC.

CircRNA pull-down. RNA pulldown experiments were carried out according to the manufacturer's instructions using a Pierce Magnetic RNA-Protein Pull-Down kit (Thermo). A biotinylated cLMNB1 probe (0.2 nmol, Riobio) was pre-treated with magnetic beads before being incubated with PC9OR and HCC827OR cell lysates overnight at 4 °C. IB and MS were used to examine the isolated proteins. The biotinylated cLMNB1 probe sequence is TTAGCATAGCTCTTTCTGTAGATG, and the proteins detected by MS are listed in Supplementary Table 5.

The Proximity Ligation Assay (PLA). PLA is a technique employed to detect specific interactions between proteins using secondary antibodies that are linked to oligonucleotides.

When two distinct proteins are in close proximity, typically less than 40 nanometers apart, the oligonucleotides labeled with fluorescence can trigger the amplification of a Texas red signal under appropriate conditions. The PLA procedure was carried out utilizing the Duolink® In Situ Red Starter Kit Mouse/Rabbit, following the instructions provided by the manufacturer (Sigma-Aldrich, USA). The process began with cells being fixed using a 4% paraformaldehyde solution and then made permeable with 0.2% Triton X-100. After this, the cells were treated with a blocking solution for one hour, followed by an incubation with primary antibodies at 4°C for an entire night. Subsequently, the cells were incubated with secondary antibodies that had oligonucleotides attached (anti-rabbit PLUS probe and anti-mouse MINUS probe) at 37°C for one hour. This was succeeded by incubations with a ligation-ligase solution at 37°C for one hour and an amplification-polymerase solution at 37°C for one hour and forty minutes. The resulting fluorescent signals were observed under an OBSERVER D1/AX10 cam HRC microscope from Zeiss, located in Oberkochen, Germany. Each individual fluorescent spot indicated the close association of two interacting proteins within the cell.

RIP. The RIP assay was carried out according to the manufacturer's guidelines using a Magna RIP RNA-Binding Protein Immunoprecipitation kit (Millipore). In brief, 5 μg of control IgG antibody (1:20; Thermo), METTL3 antibody (86132S, Cell Signaling Technology), YTHDF2 antibody (71283S, Cell Signaling Technology), FGFR4 antibody (8562S, Cell Signaling Technology), c-Cbl antibody (sc-1651, Santa Cruz) was coupled to magnetic beads and treated overnight at 4 °C with PC9OR and HCC827OR cell lysates, respectively. The protein was then digested with proteinase K, and the RNA was extracted using phenol-chloroform. Finally, reverse transcription and qRT-PCR were used to measure cLMNB1 expression.

Co-IP. Cells were lysed in immunoprecipitation lysis buffer (Thermo) by spinning the cell lysate on a rotor wheel for 10 minutes at 4 °C. All supernatants were pooled after centrifugation of the samples at 15,000 g. The immunoprecipitation input was removed, and the samples were incubated with appropriate magnetic beads (Thermo) for 3 hours at 4 °C with rotation. Afterwards, the beads were washed three times with immunoprecipitation wash buffer and eluted with Laemmli buffer. The purified proteins were analysed by IB.

Immunoblotting and antibodies. After washing the cells 3 times with ice-cold PBS, cells were harvested by centrifugation at 1000 rpm for 5 minutes and then lysed using RIPA buffer (Thermo) containing phosphatase and protease inhibitors (Thermo) for 30 minutes at 4°C. Collect the supernatant by centrifugation at 15,000 g for 15 minutes at 4 °C. Protein concentration was determined by BCA Protein Assay (Beyotime). Denatured lysates were separated on 4 - 20% SDS-PAGE gels and transferred to polyvinylidene fluoride (PVDF) membranes (Millipore). Membranes were blocked in 5% non-fat dry milk for 2 hours and then immunoblotted with primary antibody overnight. After incubation with the secondary antibodies, the signal of relevant proteins was detected with the ECL chemiluminescence detection kit (Vazyme, Nanjing, China). Some membranes were incubated with Alexa 680 and 800-conjugated species-specific secondary antibodies (Rockland). Western blots were visualized with an infrared scanner (LI-COR). The following primary antibodies were used: from Cell Signaling Technology (all at 1:1000 dilution), METTL3 antibody (86132S), YTHDF2 antibody (71283S), FGFR4 antibody (8562S), Ubiquitin P4D1 (3936S), DYKDDDDK Tag (14793S); from Abcam, p-FGFR4 (Y642) (ab192589); from Santa Cruz, c-Cbl antibody (sc-1651); from Proteintech, Flag-Tag (66008-4-Ig), MYC-Tag (16286-1-AP, 60003-2-Ig), 6×His-Tag (10001-0-AP, 66005-1-Ig), GAPDH (60004-

1-Ig). The antibodies were employed at the manufacturer's recommended dilutions.

IF. Immunofluorescence was performed using a specific antibody to FGFR4 antibody (8562S, Cell Signaling Technology) in Fig. 4F, FGFR4 antibody (8562S, Cell Signaling Technology) and c-Cbl antibody (sc-1651, Santa Cruz) in Fig. 6N. PC9OR and HCC827OR cells were fixed with 4% formaldehyde for 15 minutes before being blocked for 60 minutes at room temperature with 5% normal goat serum. Alexa Fluor 488-labeled, or Alexa Fluor 555-labeled secondary antibodies (Thermo) were used for immunostaining. Nuclei were counterstained with DAPI.

IHC. IHC was performed on the formalin-fixed, paraffin-embedded mouse or human tumour tissue sections using FGFR4 antibody (8562S, Cell Signaling Technology), Ki-67 antibody (27309-1-AP, Proteintech), and m6A antibody (68055-1-Ig, Proteintech). IHC was carried out using an automated protocol developed for the BenchMark XT automated slide-staining system (Ventana Medical Systems) and was detected using an ultraView Universal DAB detection kit (Ventana Medical Systems). Hematoxylin II (Ventana-Roche) was used as a counterstain. The intensity of staining was graded as 0 (negative), 1 (weak), 2 (moderate), and 3 (strong). The frequency was graded from 0 to 4 by the percentage of positive cells as follows: grade 0, <3%; grade 1, 3% to 25%; grade 2, 25% to 50%; grade 3, 50% to 75%; and grade 4, >75%. Product score = intensity * frequency score.

Surface plasmon resonance (SPR). This analysis was conducted using a BIAcore X100 system equipped with a sensor chip that featured a dextran matrix and had streptavidin pre-immobilized (supplied by GE Healthcare). The FGFR4 recombinant protein was sourced from MCE (catalog number HY-P76929), and cLMNB1 was custom-synthesized by Geneseed (Guangzhou, China). Initially, the FGFR4 recombinant protein was affixed to the sensor chip

according to the supplier's protocol. A Tris-HCl buffer at a concentration of 50 mM and a pH level of 7.4 served as the flow buffer. A range of cLMNB1 concentrations were formulated, and their interactions with FGFR4 were quantified through SPR analysis. Given the rapid association (Kon) and dissociation (Koff) rates, the dissociation constant (KD) values were derived using a one-site binding model in a steady-state scenario. The model was represented by the formula Y = Bmax * X / (KD + X) + background, where X denotes the varying concentrations of the analyte, and Y represents the maximum response signal in resonance units.

Production and purification of circRNA. A base at position 252 of the sequence of cLMNB1 was mutated to T base. The cLMNB1-mut was produced and purified by Geneseed (Guangzhou, China). The fully processed RNA was purified via lithium chloride precipitation and resuspended in RNase-free water. For RNase digestion assay. To confirm the generation of circRNA via splicing reaction, RNA samples were digested by RNase R (Genesee) at 37 °C for 15 min, followed by agarose gel electrophoresis. RNA samples were also reverse transcribed to cDNA and then amplified via PCR by junction-spanning primers (F: CGCTTGGTAGAGGTGGATTC, R: CTGTAGATTAGAAAGCTGGG). After that the sample was sent for Sanger sequencing.

Encapsulation of circRNA by LNP. For the synthesis of multi-armed ionizable lipid, PAMAM dendrimer G0 was mixed with 1,2-epoxytetradecane at a molar ratio of 1:7. The mixture was reacted under vigorous stirring at 90 °C for 3 days. The crude reaction mixture was separated by chromatography on silica with gradient elution from CH2Cl2 to CH2Cl2/MeOH/NH4OH (75/22/3, v/v/v). LNP were prepared by combining an aqueous phase containing circRNA with

an ethanol phase containing the lipid and cholesterol components via microfluidic mixing devices (Micro&Nano Technologies). The devices utilized chaotic mixing features to induce fluid folding in a state of laminar flow to reproducibly form homogeneous LNP. The aqueous phase was composed of 100 mM citrate buffer and circRNA. The ethanol phase contained the ionizable lipid, 1,2-distearoyl-sn-glycerol-3-phosphocholine (Sinopeg), cholesterol (Sinopeg), and lipid anchored polyethylene glycol (Sinopeg) at the molar ratio of 50:10:38:2. The aqueous and ethanol phases were then mixed in the microfluidic device at a 3:1 ratio.After synthesis, the LNP were dialyzed against PBS for 12 hours (MWCO = 3.5 kDa). To measure size, the LNPs were suspended in PBS and analyzed using dynamic light scattering (DLS) performed on a Zetasizer Nano (Malvern Instruments, Malvern, UK). The diameter and polydispersity index of the LNP were measured in triplicate.

Intracellular distribution of LNP. PC9OR and HCC827OR cells were cultured in glass-bottomed confocal dishes. GFP-encapsulated LNPs were added to cells and cells were imaged via LSM 880 laser confocal microscope (Carl Zeiss).

***In vivo* LNP-circRNA vaccine injection assay.** To verify the distribution and metabolism *in vivo*, tail vein injection of LNPs-circRNA (0.3 mg/kg) or LNPs-PBS were performed. Mice were injected intraperitoneally with D-Luciferin potassium salt (15 mg/ml, 10 μl/g, Beyotime) at the indicated times post-injection. Mice were assessed by bioluminescence imaging (PerkinElmer) 10 minutes later.

Supplementary figures

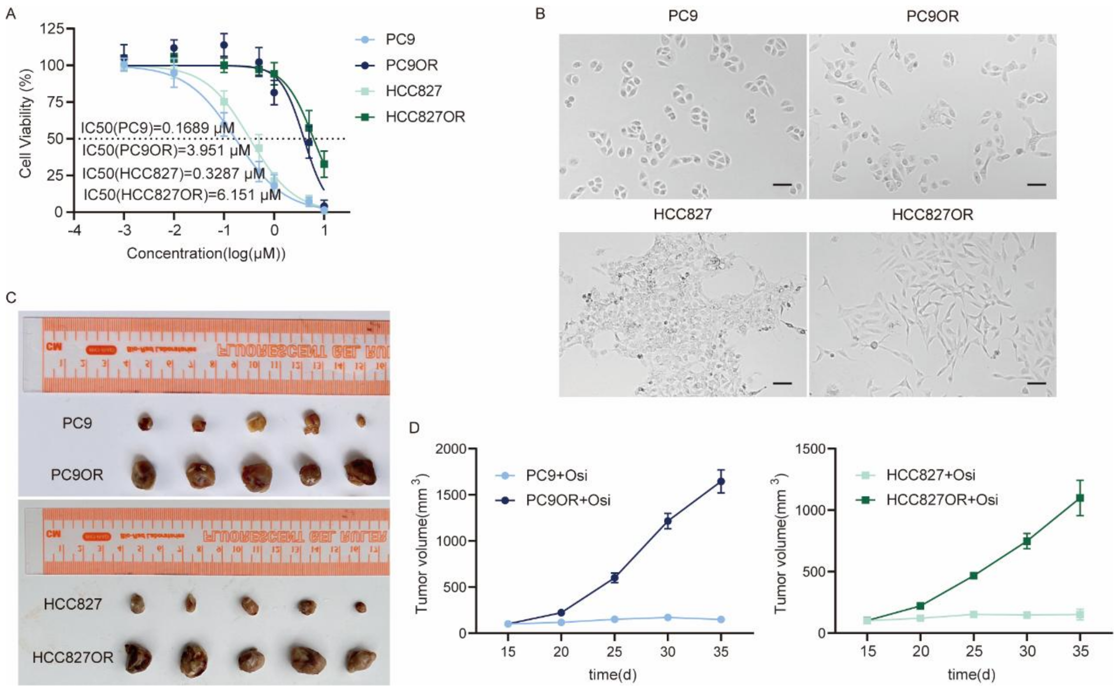

Supplementary Fig. 1 cLMNB1 is poorly expressed and highly methylated in osimertinib-resistant LUAD. A, PC9OR, HCC827OR, and their parental cells were treated with osimertinib (Osi) at the indicated concentrations. IC50 was assessed after 3 days. B, Bright-field micrographs of cultured parental cells and resistant cells treated with vehicle or Osi. Scale bar, 200 μm. C, Upper, mice bearing PC9OR and parental cell xenograft tumors were treated with Osi for 20 days followed by treatment cessation and follow-up (n=5). Lower, the results of HCC827OR and HCC827 cell xenograft tumors were shown. D, The volume of tumors from C were plotted. Three independent experiments were conducted for each result. $^{**}p < 0.01$, $^{****}p < 0.0001$ compared with the controls.

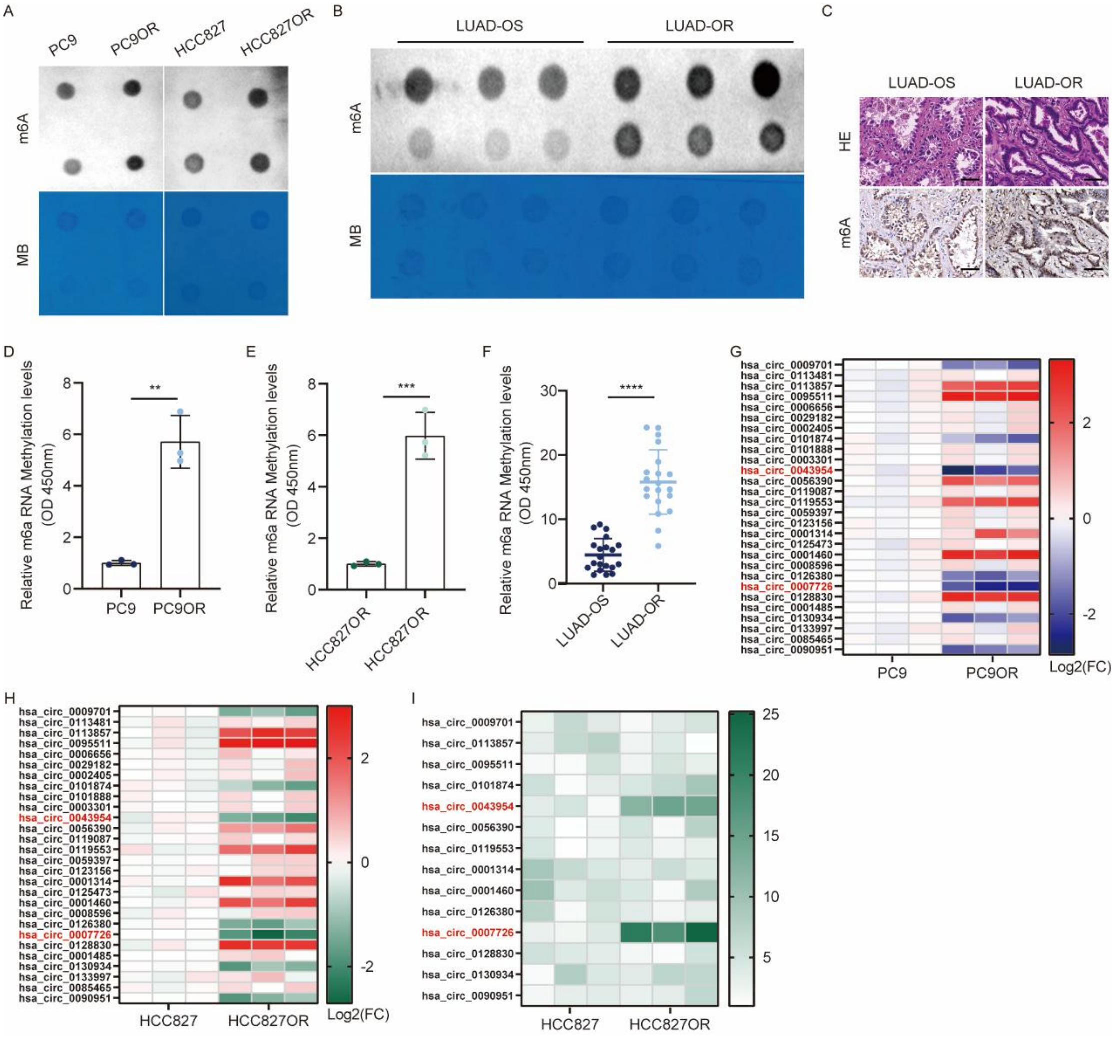

Supplementary Fig. 2 cLMNB1 is poorly expressed and highly methylated in osimertinib-resistant LUAD. A, RNA dot blot analysis (upper) of m6A levels in PC9OR, HCC827OR and their parental cells. Methylene blue staining served as a loading control (lower). B, RNA dot blot analysis of m6A levels in LUAD-OR and LUAD-OS tissues (upper) and its loading control (lower). C, IHC of m6A levels in sensitive and resistant LUAD tissues. Scale bars, 50 μm. D,E, Relative m6A levels of circRNAs in PC9OR, HCC827OR and their parental cells (n=3), as assessed by the m6A RNA Methylation Quantification Kit. F, Relative m6A levels of circRNAs in LUAD-OR and LUAD-OS tissues (n=20). G,H, The expression levels of 28 circRNAs in PC9OR (G) and HCC827OR (H) cells were detected by qPCR (n=3). The fold change took the log function. Red represented up and blue or green meant down. I, The m6A modification levels

of 14 circRNAs in HCC827 and HCC827OR cells were detected by MeRIP-qPCR (n=3). The darker green represented the larger fold change. Three independent experiments were conducted for each result. **p < 0.01, ***p < 0.001, ****p < 0.0001 compared with the controls.

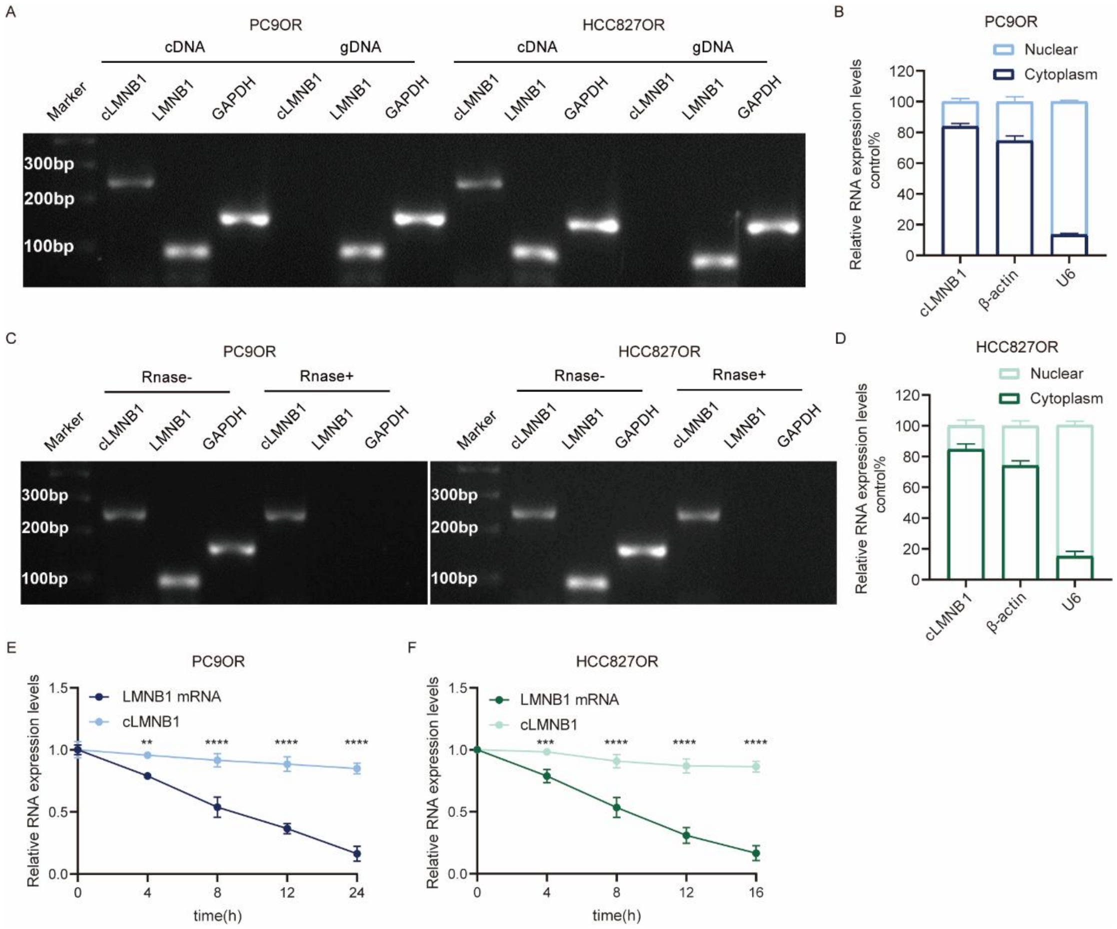

Supplementary Fig. 3 cLMNB1 is poorly expressed and highly methylated in osimertinib-resistant LUAD. A, Convergent and divergent primers were used to validate the loop structure of cLMNB1. B, Detection of cLMNB1 expression in cytoplasmic and nuclear fractions of RNAs extracted from PC9OR cells. C, The expression of cLMNB1, linear LMNB1 and GAPDH in cells with or without RNase R treatment. D, Detection of cLMNB1 expression in cytoplasmic and nuclear fractions of RNAs extracted from HCC827OR cells. E,F, Relative RNA levels of cLMNB1 and linear LMNB1 after actinomycin D treatment detected by qPCR. Three independent experiments were conducted for each result. **p < 0.01, ****p < 0.0001 compared

with the controls.

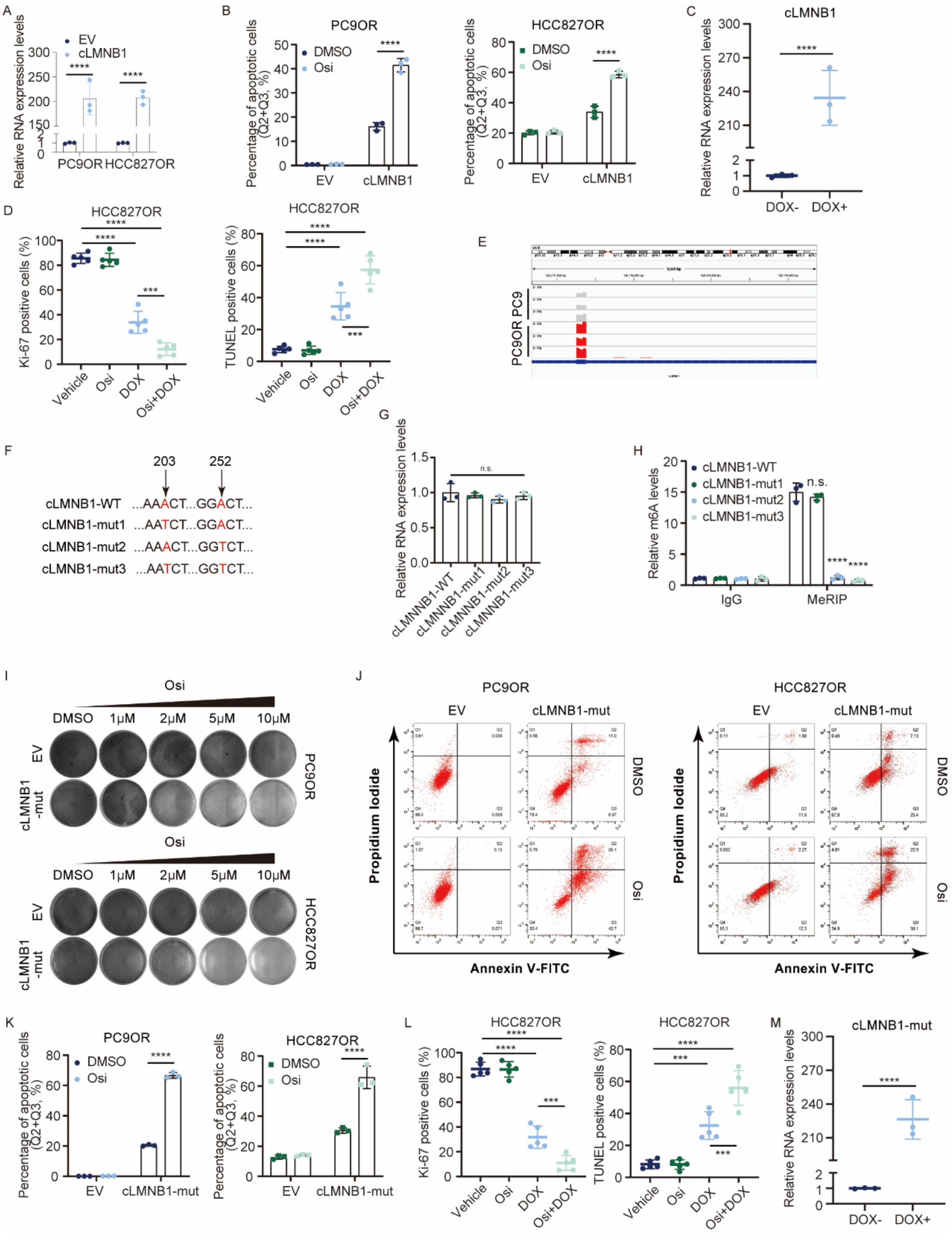

Supplementary Fig. 4 cLMNB1 overcomes osimertinib resistance *in vitro and in vivo* independently of m6A modifications. A, The expression levels of cLMNB1 were measured by qPCR with empty vehicle or cLMNB1 overexpression. B, Percentage of apoptotic PC9OR (left) and HCC827OR (right) cells from Fig.2C treated with osimertinib (Osi). C, The expression

levels of cLMNB1 were measured by qPCR in mice xenograft tumors feeding with or without Doxycycline (DOX) (n=3). D, Per cent Ki-67 positive cells and per cent necrosis area of HCC827OR cell xenograft tumors from Fig. 2F are plotted. E, Visualization of the predicted m6A modification peak of cLMNB1 from MeRIP-seq using IGV program. F, Schematic diagram showing the specific mutation site of cLMNB1. G, The expression of cLMNB1 was detected by qPCR in the cells transfected with cLMNB1 wild type and mutant plasmids. WT: wild type, mut: mutant. H, MeRIP-qPCR analysis of m6A modification levels of cLMNB1. I, PC9OR (upper) and HCC827OR (lower) cells with cLMNB1-mut overexpression were treated with Osi at the indicated concentrations, following by crystal violet staining. J, Apoptosis levels of PC9OR (left) and HCC827OR (right) cells treated with Osi were analysed by FACS. K, Percentage of apoptotic PC9OR (left) and HCC827OR (right) cells of E treated with osimertinib (Osi). L, Per cent Ki-67 positive cells and per cent necrosis area of HCC827OR cell xenograft tumors from Fig. 2L are plotted. M, The expression levels of cLMNB1-mut were measured by qPCR in mice xenograft tumors feeding with or without Doxycycline (DOX) (n=3). Three independent experiments were conducted for each result. ***$p < 0.01$, ****$p < 0.0001$ compared with the controls.

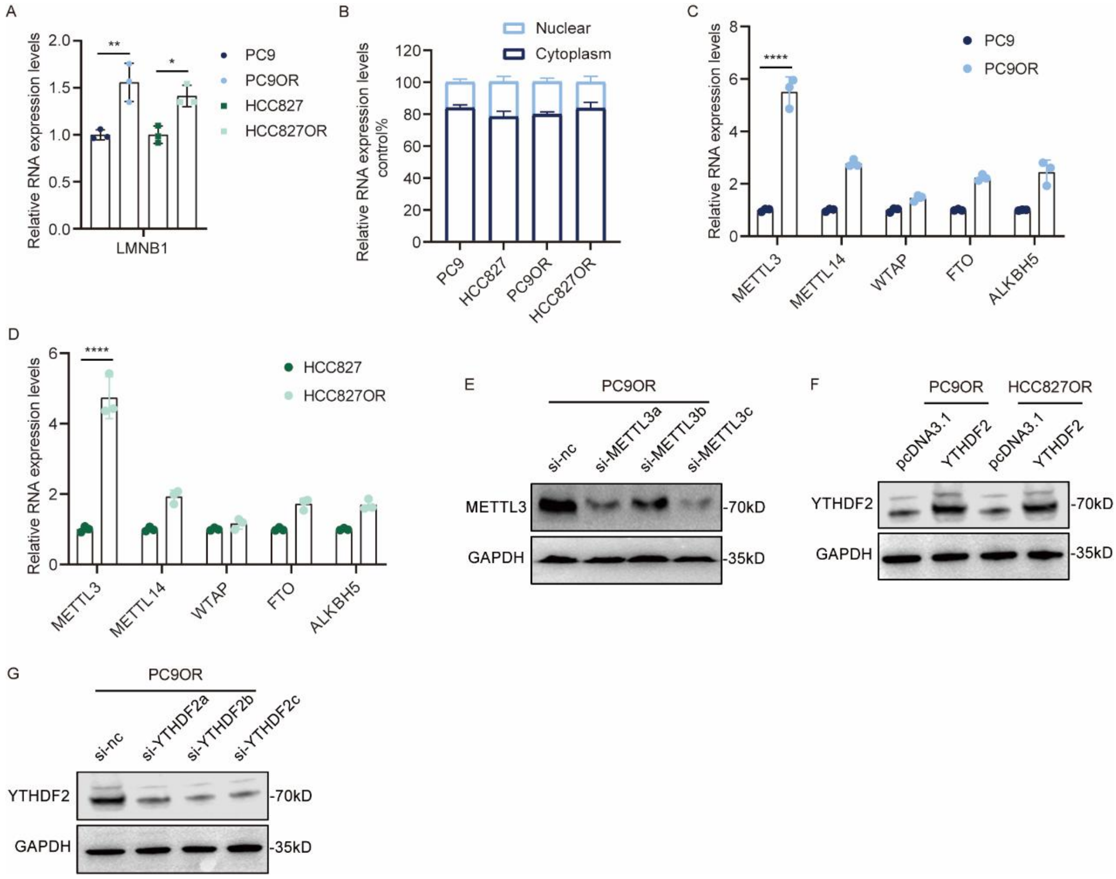

Supplementary Fig. 5 METTL3 and YTHDF2 mediate cLMNB1 degradation. A, The expression of linear LMNB1 mRNA was detected by qPCR in resistant and parental cells. B, Detection of cLMNB1 expression in cytoplasmic and nuclear fractions of RNAs extracted from resistant and parental cells. C,D, The mRNA expression of m6A writers (METTL3, METTL14, WTAP) and erasers (FTO, ALKBH5) were plotted in PC9 (C) and HCC827 (D) cell lines. E, The protein expression of METTL3 suppression validated by Western blot. F, The protein expression of YTHDF2 overexpression validated by Western blot. G, The protein expression of YTHDF2 suppression validated by Western blot. Three independent experiments were conducted for each result. n.s. no significance, *p < 0.01, **p < 0.01, ****p < 0.0001 compared with the controls.

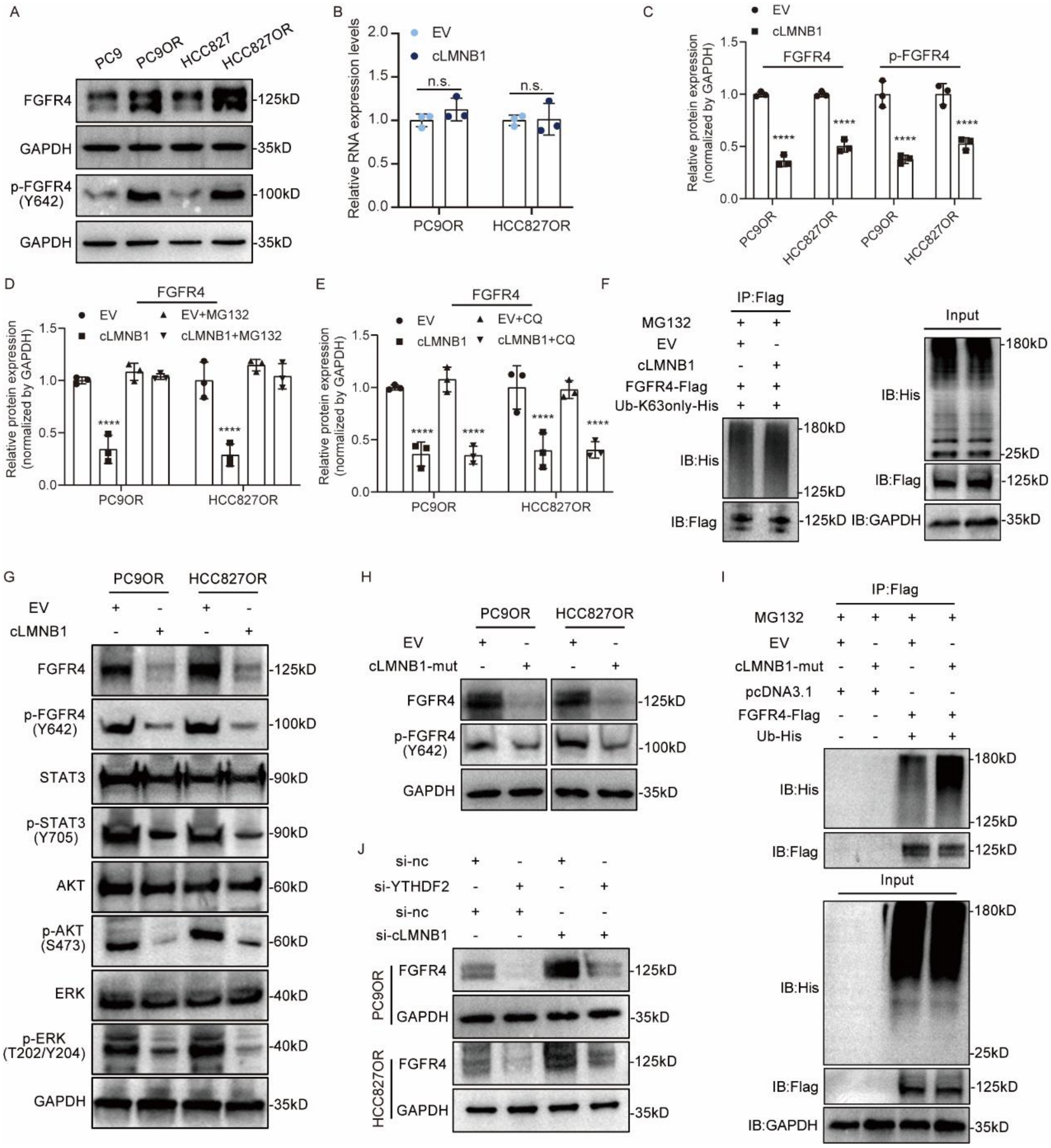

Supplementary Fig. 6 cLMNB1 destabilizes FGFR4 via K48-linked polyubiquitination. A, The protein levels of FGFR4 and its phosphorylated form in resistant and parental cells were detected by Western blot. B, The FGFR4 mRNA expression detected by qPCR in resistant cells transfected with cLMNB1 plasmids. C, The statistical analysis of protein levels of FGFR4 and its phosphorylated form after cLMNB1 overexpression (n=3). D, The statistical analysis of protein levels of FGFR4 protein levels regulated by cLMNB1 with or without MG132 treatment (n=3). E, The statistical analysis of protein levels of FGFR4 protein levels regulated by cLMNB1

with or without CQ treatment (n=3). F, Western blot assay showing the upregulation of K63-linked ubiquitination with cLMNB1 overexpressing in PC9OR cells. Ub-K63only-His: the cells were transfected with plasmids expressing His-tagged ubiquitin with all lysines mutated except K63. G, Western blot for protein levels of key downstream signaling pathways of FGFR4 and corresponding phosphorylated form after cLMNB1 overexpression. H, Western blot for protein levels of FGFR4 and its phosphorylated form after cLMNB1-mut overexpression. I, Effects of cLMNB1-mut overexpression on the ubiquitination of FGFR4 proteins. J, The protein levels of FGFR4 were detected after YTHDF2 or/and cLMNB1 suppression. Three independent experiments were conducted for each result. n.s. no significance compared with the controls, ****$p < 0.0001$ compared with the controls.

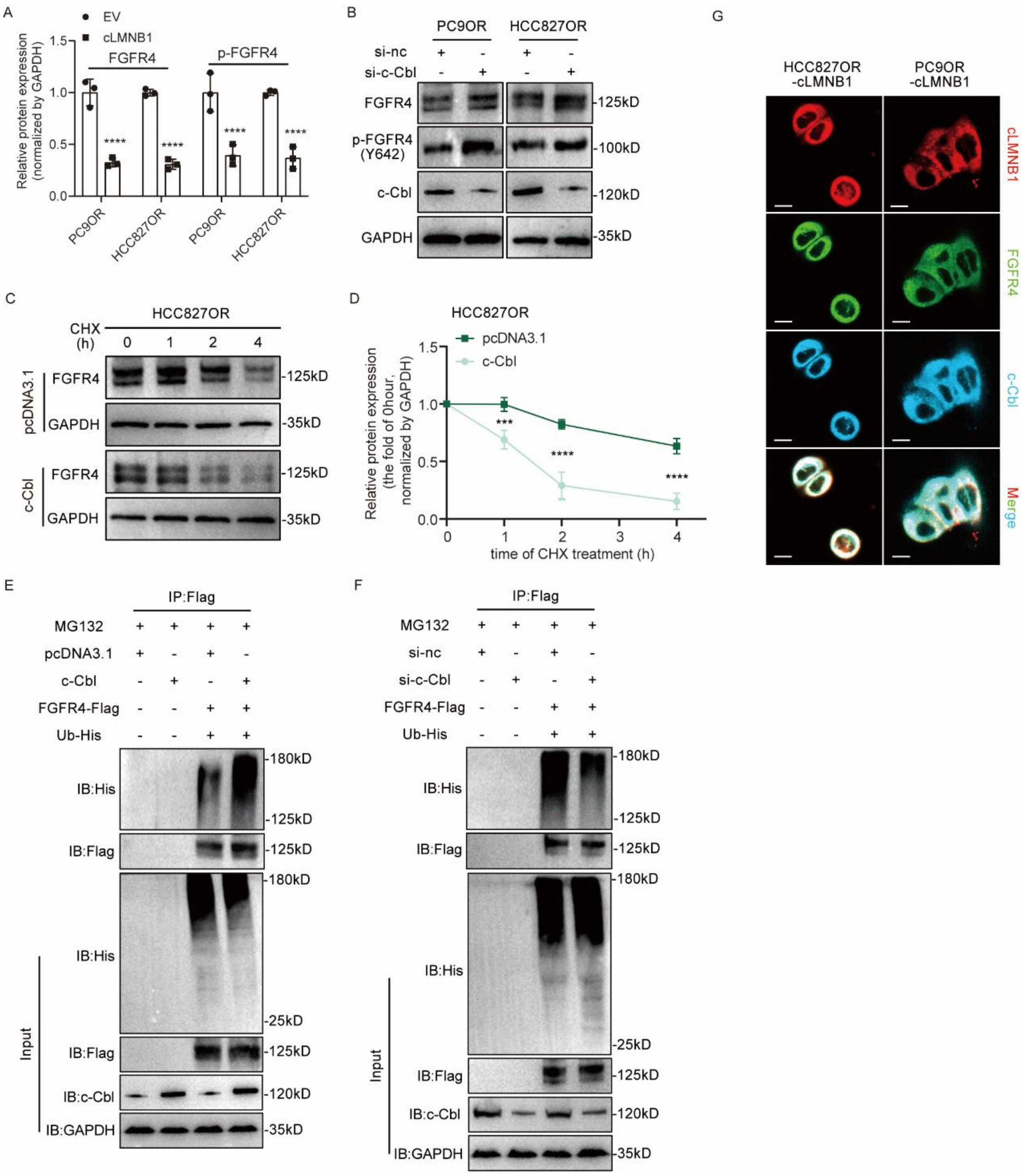

Supplementary Fig. 7 cLMNB1 promotes ubiquitination degradation of FGFR4 through c-Cbl. A, The statistical analysis of protein levels of FGFR4 and its phosphorylated form after c-Cbl overexpression (n=3). B, Western blot for protein levels of FGFR4 and its phosphorylated form after c-Cbl suppression. C,D, HCC827OR cells with c-Cbl overexpression were treated with cycloheximide (CHX) for the indicated times. Western blot analysis of FGFR4 protein levels upon CHX treatment are presented, with the level at 0 h as a control (C). The statistical analysis is shown in D. E,F, Effects of c-Cbl overexpression (E) and suppression (F) on the ubiquitination

of FGFR4 proteins. G, FISH and IF assays showing the colocalization of FGFR4, c-Cbl and cLMNB1. Scale bar, 20 μm. Three independent experiments were conducted for each result. ***p < 0.01, ****p < 0.0001 compared with the controls.

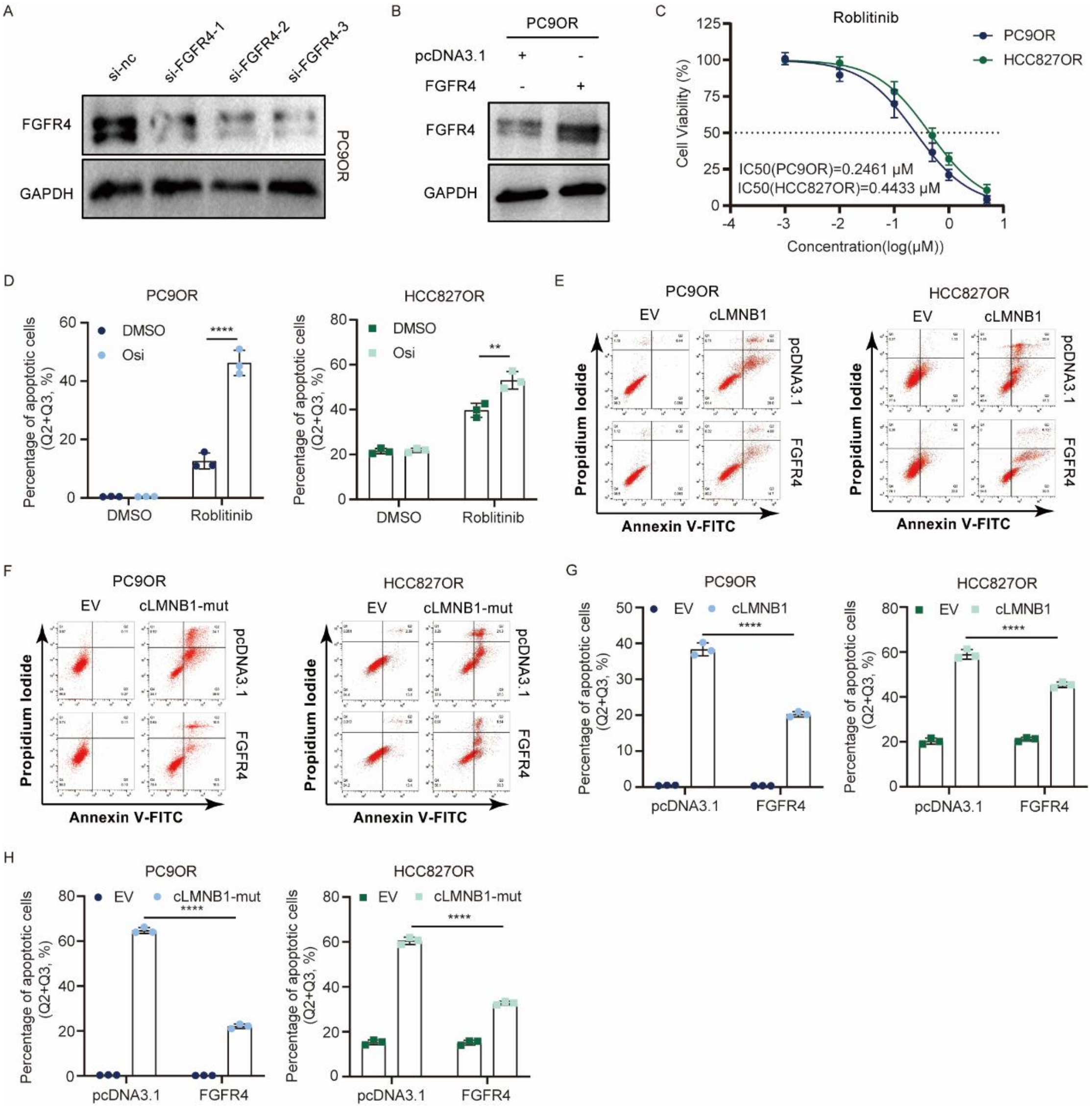

Supplementary Fig. 8 cLMNB1 increases the sensitivity of LUAD to osimertinib through FGFR4 inhibition. A, The protein expression of FGFR4 suppression was validated by Western blot. B, The protein expression of FGFR4 overexpression was validated by Western blot. C, PC9OR and HCC827OR cells were treated with Roblitinib at the indicated concentrations. IC50 was assessed after 3 days. D, Percentage of apoptotic PC9OR (left) and HCC827OR (right)

cells from Fig.7C. E, PC9OR and HCC827OR cells were transfected with cLMNB1 or/and FGFR4 overexpression plasmids. Apoptosis levels of PC9OR (left) and HCC827OR (right) cells treated with osimertinib (Osi) were analysed by FACS. F, PC9OR and HCC827OR cells were transfected with cLMNB1-mut or/and FGFR4 overexpression plasmids. Apoptosis levels of PC9OR (left) and HCC827OR (right) cells treated with osimertinib (Osi) were analysed by FACS. G, Percentage of apoptotic PC9OR (left) and HCC827OR (right) cells of E. H, Percentage of apoptotic PC9OR (left) and HCC827OR (right) cells of F. Three independent experiments were conducted for each result. **p < 0.01, ****p < 0.0001 compared with the controls.

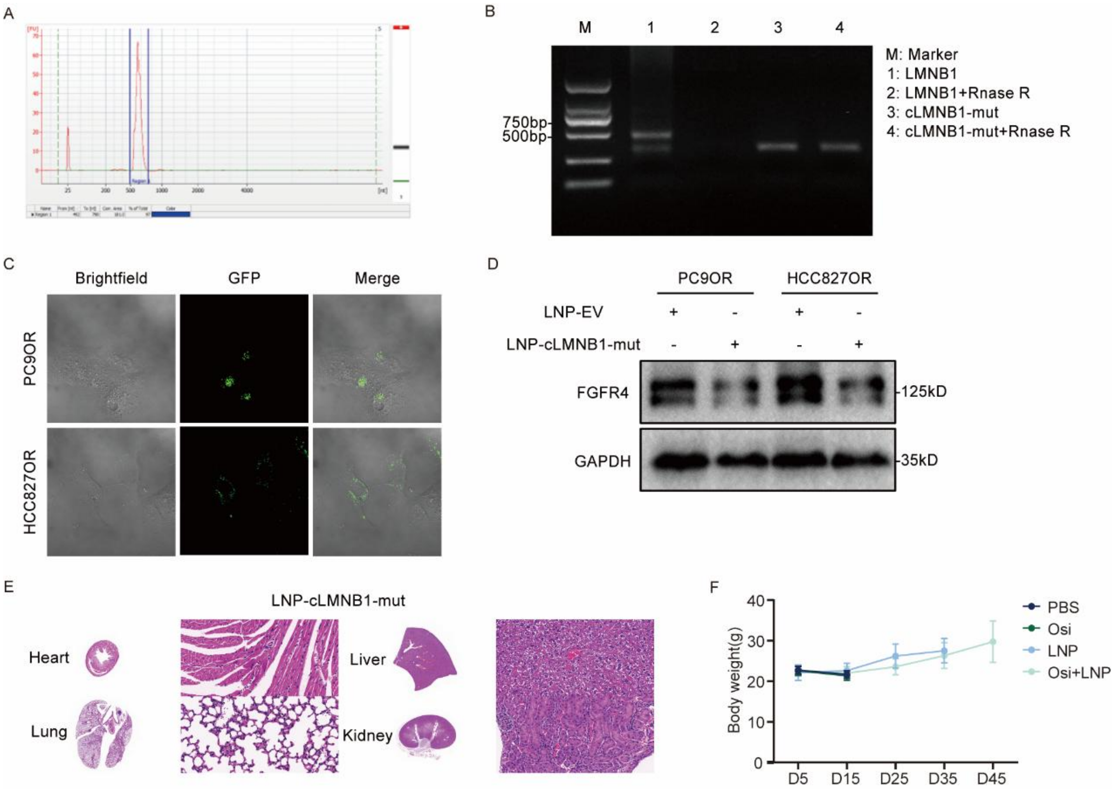

Supplementary Fig. 9 The cLMNB1-mut encapsulated in lipid nanoparticles is promising to overcome osimertinib resistance. A, The integrity and purity of cLMNB1-mut was verified by Agilent 2100 instrument. B, The expression of cLMNB1-mut and linear LMNB1 in cells with or without RNase R treatment. C, PC9OR and HCC827OR cells were given LNPs-GFP. Representative images of LNPs-GFP that were taken up were recorded by laser confocal

microscope. Scale bar, 20 μm. D, Western blot for protein levels of FGFR4 in the resistant cells treated with LNPs-cLMNB1-mut. E, Hematoxylin-eosin (H&E, 1×, 40×) analysis of mice treated with LNPs-cLMNB1-mut. Scale bar, 1000 μm (left), 50 μm (right). F, Body weight of PBS, Osi, LNP, Osi + LNP group every 10 days (n=5). Three independent experiments were conducted for each result.

Supplementary Table 1

Number	Sample type	Gender	Age	Smoking	Surgery	Tumor size (cm	Tumor location	Tumor differentiate	Pleura-	Lymph node	TNM stage
LUAD-S-01	Frozen sample	Female	67	No	2022/2/14	2.3*2*1.7	Left upper lobe	Moderate differentiation	No	No	T1cN0M0
LUAD-S-02	Frozen sample	Male	70	No	2022/3/15	3*3*2	Right middle	High differentiation	No	No	T1cN0M0
LUAD-S-03	Frozen sample	Female	55	No	2022/4/20	1.5*1.5*1.5	Left lower lobe	Poor differentiation	No	10,11	T1bN1M0
LUAD-S-04	Frozen sample	Male	60	No	2022/5/22	4.5*3.5*2.8	Right upper lobe	Moderate differentiation	No	No	T2bN0M0
LUAD-S-05	Frozen sample	Female	72	No	2022/6/18	2*2*1.8	Left lower lobe	High-moderate differentiation	No	No	T1bN0M0
LUAD-S-06	Frozen sample	Female	65	Yes	2022/7/21	3*3*2.5	Right lower lobe	Poor differentiation	No	No	T1cN0M0
LUAD-S-07	Frozen sample	Female	68	No	2022/8/30	2.2*2.2*2	Left upper lobe	Moderate differentiation	No	No	T1cN0M0
LUAD-S-08	Frozen sample	Male	59	Yes	2022/9/11	3.5*3.1*2.4	Right upper lobe	Moderate to low differentiation	No	10,11	T2aN1M0
LUAD-S-09	Frozen sample	Female	66	No	2022/10/14	2*2*1.5	Left lower lobe	Poor differentiation	No	No	T1bN0M0
LUAD-S-10	Frozen sample	Male	63	Yes	2022/11/19	2.7*2.7*2.2	Right middle	Moderate differentiation	No	No	T1cN0M0
LUAD-S-11	Frozen sample	Male	71	No	2023/1/4	5.6*4.2*3.9	Left upper lobe	High-moderate differentiation	No	No	T3N0M0
LUAD-S-12	Frozen sample	Male	62	Yes	2023/2/13	2.8*2.8*2.5	Right lower lobe	Poor differentiation	No	No	T1cN0M0
LUAD-S-13	Frozen sample	Female	56	No	2023/3/11	1.8*1.8*1.3	Left upper lobe	Moderate differentiation	No	No	T1bN0M0
LUAD-S-14	Frozen sample	Male	64	Yes	2023/4/17	3.1*3*2.7	Right upper lobe	Moderate to low differentiation	No	No	T2aN0M0
LUAD-S-15	Frozen sample	Female	61	No	2023/5/16	2.7*2.5*2	Left lower lobe	Poor differentiation	No	No	T1cN0M0
LUAD-S-16	Frozen sample	Male	58	No	2023/6/30	2.6*2.2*1.8	Right middle	Moderate differentiation	No	No	T1cN0M0
LUAD-S-17	Frozen sample	Male	65	No	2023/7/21	2.7*2.1*2	Left upper lobe	Well differentiation	No	No	T1cN0M0
LUAD-S-18	Frozen sample	Male	61	Yes	2023/8/24	4.8*3.8*3.5	Right lower lobe	Poor differentiation	No	10	T2bN1M0
LUAD-S-19	Frozen sample	Female	67	No	2023/9/27	2.6*2.5*2	Left upper lobe	Moderate differentiation	No	No	T1cN0M0
LUAD-S-20	Frozen sample	Male	66	No	2023/10/30	3*3*2.7	Right upper lobe	High differentiation	No	No	T1cN0M0

Number	Sample type	Gender	Age	Smoking	Lung Biopsy Date	Tumor size (cm^3)	Tumor location	Tumor differentiate	Lymph node metastasis	Lymph node group	Distant metastasis	Metastasis organ	TNM stage
LUAD-R-01	Frozen sample	Female	57	No	2022/2/26	2.6*2.2*1.6	Left upper lobe	Moderate differentiation	Yes	2R, 4R	No	None	T1cN2M0
LUAD-R-02	Frozen sample	Male	70	No	2022/3/10	3.5*2.1*2	Right lower lobe	Poor differentiation	Yes	10, 11	No	None	T2aN1M0
LUAD-R-03	Frozen sample	Male	65	No	2022/6/18	2.8*2.3*1.9	Left lower lobe	High differentiation	Yes	10	No	None	T1cN1M0
LUAD-R-04	Frozen sample	Male	73	Yes	2022/7/12	4.1*3.2*2.5	Right upper lobe	Moderate differentiation	No	None	No	None	T2bN0M0
LUAD-R-05	Frozen sample	Female	69	No	2022/8/5	3.2*2.7*2.1	Left upper lobe	Poor differentiation	Yes	2R, 4R	No	None	T2aN2M0
LUAD-R-06	Frozen sample	Male	68	No	2022/9/22	2.9*2.1*1.8	Right middle lobe	Moderate differentiation	No	None	Yes	Liver	T1cN0M1
LUAD-R-07	Frozen sample	Male	72	No	2022/10/14	3.8*3*2.4	Left lower lobe	High differentiation	No	None	No	None	T2aN0M0
LUAD-R-08	Frozen sample	Male	66	Yes	2022/11/30	4.3*3.1*2.9	Right upper lobe	Poor differentiation	Yes	7, 8	No	None	T2bN2M0
LUAD-R-09	Frozen sample	Female	71	No	2022/12/19	3*2.4*2.2	Left upper lobe	Moderate differentiation	Yes	10, 11	No	None	T1cN1M0
LUAD-R-10	Frozen sample	Male	69	Yes	2023/1/6	3.6*2.9*2	Right lower lobe	High differentiation	Yes	10	Yes	Liver	T2aN1M1
LUAD-R-11	Frozen sample	Female	68	No	2023/1/11	3.7*3.1*2.6	Left lower lobe	Poor differentiation	No	None	Yes	Brain	T2aN0M1
LUAD-R-12	Frozen sample	Male	70	No	2023/2/25	4*3.3*2.8	Right upper lobe	Moderate differentiation	Yes	4R, 7	Yes	Bone	T2bN2M1
LUAD-R-13	Frozen sample	Female	49	No	2023/3/26	2.5*2*1.5	Left upper lobe	High differentiation	No	None	No	None	T1bN0M0
LUAD-R-14	Frozen sample	Male	57	Yes	2023/4/5	3.9*3*2.5	Right lower lobe	Poor differentiation	Yes	2R, 10	Yes	Brain	T2aN1M1
LUAD-R-15	Frozen sample	Female	65	No	2023/4/21	2.7*2.3*2	Left lower lobe	Moderate differentiation	Yes	5, 6	No	None	T1cN2M0
LUAD-R-16	Frozen sample	Female	67	No	2023/5/4	4.2*3.2*2.8	Right middle lobe	High differentiation	Yes	4L, 9	No	None	T2bN2M0
LUAD-R-17	Frozen sample	Female	62	No	2023/5/13	3.3*2.8*2.4	Left upper lobe	Poor differentiation	Yes	5, 6	Yes	Adrenal Gland	T2aN2M1
LUAD-R-18	Frozen sample	Male	71	Yes	2023/8/14	3.4*2.9*2.1	Right lower lobe	Moderate differentiation	Yes	10	No	None	T1cN1M0
LUAD-R-19	Frozen sample	Female	69	No	2023/9/13	4*3.5*3	Left lower lobe	High differentiation	Yes	4L, 9	No	None	T2bN2M0
LUAD-R-20	Frozen sample	Male	68	No	2023/9/24	3.1*2.5*2	Right upper lobe	Poor differentiation	Yes	7, 8	Yes	Liver	T1cN2M1

Supplementary Table 2

**Compounds used in this study.**			
**Reagent or resource**	**Source**	**Identifier**	**Concentration**
DMSO	Solarbio	Cat# D9371	\
Doxycycline	MedChemExpress	Cat# HY-N0565	100 mg/kg
Osimertinib	MedChemExpress	Cat# HY-15772	1 μM / 10 mg/kg
Roblitinib	MedChemExpress	Cat# HY-101568	200 nM / 10 mg/kg
MG132	MedChemExpress	Cat# HY-13259	100 μM
Cycloheximide	MedChemExpress	Cat# HY-12320	100 μg/ml
Actinomycin D	MedChemExpress	Cat# HY-17559	1 μM
Hydroxychloroquine	MedChemExpress	Cat# HY-B1370A	10 μM
D-luciferin potassium salt	Invitrogen	Cat# L2916	150 mg/kg

Supplementary Table 3

The sequences of primers used in this study.		
**Primers for PCR (5'-3')**		**concentration**
cLMNB1-F	CAACAGTGCCAGGGAAGAAC	1μmol
cLMNB1-R	CCAACTGGGCAATCTGATCC	1μmol
LMNB1-F	ACATGGAAATCAGTGCTTACAGG	1μmol
LMNB1-R	GGGATACTGTCACACGGGA	1μmol
GAPDH-F	GGAGCGAGATCCCTCCAAAAT	1μmol
GAPDH-R	GGCTGTTGTCATACTTCTCATGG	1μmol
FGFR4-F	GAGGGGCCGCCTAGAGATT	1μmol
FGFR4-R	CAGGACGATCATGGAGCCT	1μmol
U6-F	CTCGCTTCGGCAGCACA	1μmol
U6-R	AACGCTTCACGAATTTGCGT	1μmol
METTL3-F	CTACTCGACCCACAGGTGCT	1μmol
METTL3-R	ATTACCCTTGACTGCGCTGA	1μmol
METTL14-F	ACTACGGCCGATGGTGTCTA	1μmol
METTL14-R	GGGAGGCTTCGTTACAATCA	1μmol
WTAP-F	TCTCGAGTTTGCGAATTCCT	1μmol
WTAP-R	GCGCAGTACTTCCTCTGTCC	1μmol
ALKBH5-F	CCTAAACGTTCTTGCAGGTGA	1μmol
ALKBH5-R	TTAAGTTCCAGCGGTTGTGA	1μmol
FTO-F	TTTGGCAGTTTCAGCATACG	1μmol
FTO-R	AGTCCAATGGTGAATTGCAG	1μmol
Predicted peak 01-F	GCTGGTCTCGAACTCCTGAC	1μmol
Predicted peak 01-R	ATTAGAAAAGGAGGCCGGGC	1μmol
Predicted peak 02-F	CCAGTGACAAGTTGGGAGAT	1μmol
Predicted peak 02-R	TCTCTTGGTTTCTGTGGAGCT	1μmol
Predicted peak 03-F	AAACCCAGGAGTGTCAGGAG	1μmol
Predicted peak 03-R	CGTAAAATCCCAGCCACAGG	1μmol
Predicted peak 04-F	TCCAAAACCCATACACTGATCA	1μmol
Predicted peak 04-R	GGCCCTGTCTACAATTCCCT	1μmol
Predicted peak 05-F	GCAGTGTTTGGTTTTCTGTTCT	1μmol
Predicted peak 05-R	ACCAACACAAATGCCCATCA	1μmol
Predicted peak 06-F	CCCACCTCACCTGCACAG	1μmol
Predicted peak 06-R	ACATCAAAGCCCTCACTAGTC	1μmol
Predicted peak 07-F	TGGCCTACTTGATGGAGAACT	1μmol
Predicted peak 07-R	GGGCAGTGACTCTATTTCCCT	1μmol
Predicted peak 08-F	TCTTTGTTGGGAAGAGCTTTGA	1μmol
Predicted peak 08-R	TGGAAGCAAGAGATTTTAGACCT	1μmol
Predicted peak 09-F	ACGAAGCAAAGGAAACCAAGT	1μmol
Predicted peak 09-R	ACAACTTGAAGAATGCTAGGGAC	1μmol
Predicted peak 10-F	AGATTATGCTGGCCTCCGTA	1μmol
Predicted peak 10-R	TCTCCTACCATCAGAGTCCCT	1μmol
Predicted peak 11-F	AGTTCTGAAGACACATGGACAG	1μmol
Predicted peak 11-R	ACGGCAGTAAAAGGGTAGAC	1μmol
Predicted peak 12-F	AAGGAGACAGGGAAGTGCAA	1μmol
Predicted peak 12-R	CCCACTCCCACTGTTTCACT	1μmol
Predicted peak 13-F	CCAGATGAAAACCCAACCCG	1μmol

Predicted peak 13-R	CTGGGGACTGTAGCCTAGC	1μmol
Predicted peak 14-F	ACATTGGGTTGTTTTCACCTACT	1μmol
Predicted peak 14-R	TGGCTTCCACCCTCAAAAGA	1μmol

Supplementary Table 4

chrom	txStart	txEnd	PeakID	score	Peak leng	circRNA	Foldchang	P_value	FDR	Regulatio	circBaseID	source	best trans	GeneName	Catalog	predicted_sequence_length
chr12	123707584	123707674	diffreps p	8.330463	90	chr12:123	2.750193	4.67237E-09	1.76E-06	up	hsa circ 0029182	circBase	NM 0227	MPHOSPH9	exonic	2771
chr7	134164993	134165000	diffreps p	6.886443	7	chr7:1341	3.11787	1.29885E-07	2.1E-05	up		novel	NM 0202	AKR1B10	sense overlapping	52840
chr11	29402788	29403120	diffreps p	10.41328	332	chr11:294	3.721698	3.86116E-11	5.3E-08	up		PMID:250	T056445	G013057	sense overlapping	2871
chr1	47725960	47726211	diffreps p	7.108231	251	chr1:4772	2.278484	7.79415E-08	1.45E-05	up	hsa circ 0113481	circBase	NM 0030	STIL	exonic	860
chr14	103106001	103106320	diffreps p	6.367622	319	chr14:103	11.0708	4.28922E-07	5.22E-05	up		novel	NM 0151	RCOR1	intronic	26238
chr20	5935747	5935897	diffreps p	8.753885	150	chr20:593	5.262911	1.76244E-09	8.23E-07	up	hsa circ 0059397	circBase	NM 0324	MCM8	exonic	1032
chr4	1924441	1925680	diffreps p	5.167179	1239	chr4:1893	2.467499	6.80489E-06	0.000463	up		novel	NM 0073	WHSC1	sense overlapping	43097
chr11	29403141	29403960	diffreps p	12.3429	819	chr11:294	4.353894	4.54044E-13	7.17E-09	up		PMID:250	T056445	G013057	sense overlapping	2871
chr12	57136770	57136880	diffreps p	5.173286	110	chr12:571	7.627907	6.70986E-06	0.000458	up		novel	NM 0009	PRIM1	exonic	610
chr4	178274461	178274882	diffreps p	6.762308	421	chr4:1782	2.125128	1.72859E-07	2.61E-05	up	hsa circ 0001459	circBase	NM 0182	NEIL3	exonic	421
chr4	189407001	189407019	diffreps p	7.260629	18	chr4:1893	2.420241	5.48745E-08	1.11E-05	up		PMID:250	NR 0338	LINC01060	exonic	146
chr7	134259021	134259320	diffreps p	7.355139	299	chr7:1342	107	4.41429E-08	9.48E-06	up		novel	NM 0202	AKR1B10	sense overlapping	38237
chr20	5932656	5932809	diffreps p	11.02304	153	chr20:593	2.935554	9.48339E-12	2.62E-08	up	hsa circ 0059397	circBase	NM 0324	MCM8	exonic	1032
chr7	134241821	134242020	diffreps p	6.418848	199	chr7:1342	6.809129	3.81199E-07	4.76E-05	up		novel	NM 0202	AKR1B10	sense overlapping	35032
chr20	5948081	5948233	diffreps p	7.117778	152	chr20:593	2.373953	7.62469E-08	1.43E-05	up	hsa circ 0059397	circBase	NM 0324	MCM8	exonic	1032
chr17	26904767	26905120	diffreps p	7.626714	353	chr17:269	4.436522	2.36203E-08	6.06E-06	up		novel	ENST000	RP11-192H23.4	intronic	543
chr1	63269389	63269533	diffreps p	6.041888	144	chr1:6326	2.333333	9.08054E-07	9.28E-05	up	hsa circ 0113857	circBase	NM 0328	ATG4C	exonic	1157
chr17	26905141	26905310	diffreps p	8.570177	169	chr17:269	3.830853	2.69044E-09	1.15E-06	up		novel	ENST000	RP11-192H23.4	intronic	543
chr12	78415526	78415642	diffreps p	11.3289	116	chr12:784	2.481933	4.6892E-12	1.74E-08	up	hsa circ 0002405	circBase	NM 0149	NAV3	exonic	1143
chr7	24719941	24720139	diffreps p	8.206007	198	chr7:2466	2.823661	6.2229E-09	2.21E-06	up	hsa circ 0133997	circBase	NM 0164	MPP6	sense overlapping	56858
chr19	53885564	53886064	diffreps p	6.055429	500	chr19:538	2.191909	8.8018E-07	9.05E-05	up		novel	uc010ydx.	ZNF765	intronic	500
chr1	47737747	47737913	diffreps p	10.68658	166	chr1:4772	2.326084	2.05787E-11	3.9E-08	up	hsa circ 0113481	circBase	NM 0030	STIL	exonic	860
chrX	69572449	69572506	diffreps p	6.671751	57	chrX:6956	2.24412	2.12936E-07	3.05E-05	up	hsa circ 0090951	circBase	NM 0123	KIF4A	exonic	901
chr2	215638301	215638940	diffreps p	8.369881	639	chr2:2155	6	4.26696E-09	1.65E-06	up	hsa circ 0119087	circBase	NM 0004	BARD1	sense overlapping	68200
chr14	37761321	37761620	diffreps p	5.430026	299	chr14:377	77.6	3.71513E-06	0.000283	up		novel	ENST000	MIPOL1	intronic	26243
chr7	134206941	134207320	diffreps p	8.295739	379	chr7:1341	313.4	5.06129E-09	1.88E-06	up		novel	NM 0202	AKR1B10	sense overlapping	52840
chr11	64846824	64847259	diffreps p	6.286607	435	chr11:648	2.254264	5.16884E-07	6.01E-05	up		PMID:252	NM 0806	CDCA5	exonic	435
chr1	200577957	200578056	diffreps p	7.090093	99	chr1:2005	3.818391	8.12656E-08	1.5E-05	up		novel	NM 0148	KIF14	exonic	1358
chr12	104972141	104972660	diffreps p	7.433444	519	chr12:104	8.787234	3.68601E-08	8.32E-06	up		novel	ENST000	CHST11	intronic	35244
chr2	215661501	215661841	diffreps p	5.835283	340	chr2:2155	2.633839	1.46123E-06	0.000135	up	hsa circ 0119087	circBase	NM 0004	BARD1	sense overlapping	68200
chr20	5935253	5935336	diffreps p	7.776086	83	chr20:593	5.176101	1.67461E-08	4.67E-06	up	hsa circ 0059397	circBase	NM 0324	MCM8	exonic	1032
chr1	1627041	1627500	diffreps p	5.552373	459	chr1:1599	5.854785	2.80303E-06	0.000226	up		novel	NM 0017	CDK11B	sense overlapping	66359
chr11	74331061	74331134	diffreps p	6.435876	73	chr11:743	2.079698	3.66542E-07	4.61E-05	up	hsa circ 0006656	circBase	NM 0065	POLD3	exonic	617
chr11	8740421	8740569	diffreps p	6.116785	148	chr11:874	2.405024	7.64215E-07	8.1E-05	up		novel	NM 0054	ST5	antisense	163
chr5	126141262	126141388	diffreps p	11.27869	126	chr5:1261	2.421283	5.26391E-12	1.86E-08	up	hsa circ 0007726	circBase	NM 0055	LMNB1	exonic	580
chr10	91512323	91512448	diffreps p	7.084687	125	chr10:915	2.059407	8.22836E-08	1.51E-05	up	hsa circ 0019079	circBase	NM 0161	KIF20B	exonic	893
chr4	178281656	178281831	diffreps p	5.368029	175	chr4:1782	2.799842	4.2852E-06	0.000318	up	hsa circ 0001460	circBase	NM 0182	NEIL3	exonic	596
chr1	52091801	52092060	diffreps p	5.60378	259	chr1:5203	78.6	2.49012E-06	0.000206	up		novel	NM 0245	OSBPL9	sense overlapping	78049
chr1	121484051	121484060	diffreps p	5.276319	9	chr1:1214	2.298538	5.29275E-06	0.000378	up		novel	T018636	G004213	sense overlapping	1019
chr7	134190181	134190740	diffreps p	7.434996	559	chr7:1341	179.0902	3.67286E-08	8.29E-06	up		novel	NM 0202	AKR1B10	sense overlapping	52840
chr14	45705016	45705147	diffreps p	6.448048	131	chr14:457	2.062343	3.56412E-07	4.51E-05	up	hsa circ 0101888	circBase	NM 0183	MIS18BP1	exonic	451
chr9	33568561	33569120	diffreps p	12.05825	559	chr9:3352	8.247573	8.74478E-13	8.85E-09	up		novel	NM 0012	ANKRD18B	sense overlapping	43643
chr7	73654267	73654425	diffreps p	10.55399	158	chr7:7365	2.264594	2.7926E-11	4.54E-08	up		novel	NM 1814	RFC2	exonic	508
chr7	24705741	24706000	diffreps p	6.613418	259	chr7:2466	3.198175	2.43546E-07	3.38E-05	up	hsa circ 0133997	circBase	NM 0164	MPP6	sense overlapping	56858
chr12	104701721	104702040	diffreps p	6.98676	319	chr12:104	100.1	1.03095E-07	1.78E-05	up		novel	NM 0033	TXNRD1	sense overlapping	48760
chr9	33547661	33549480	diffreps p	8.917751	1819	chr9:3352	2.580589	1.20851E-09	6.11E-07	up		novel	NM 0012	ANKRD18B	sense overlapping	43643
chr1	249215361	249215780	diffreps p	5.801611	419	chr1:2492	81.7	1.57903E-06	0.000143	up		novel	NM 1707	PGBD2	sense overlapping	12913
chr1	44877961	44878540	diffreps p	9.583996	579	chr1:4487	2.599533	2.60618E-10	1.87E-07	up		novel	NM 0181	RNF220	sense overlapping	5640
chr1	1633821	1634140	diffreps p	10.07662	319	chr1:1599	2.36225	8.38267E-11	8.51E-08	up		novel	NM 0017	CDK11B	sense overlapping	66359
chr3	160149430	160149440	diffreps p	9.167574	10	chr3:1601	3.23044	6.7987E-10	3.85E-07	up		novel	NM 0054	SMC4	exonic	2043
chr8	124351550	124351560	diffreps p	7.689138	10	chr8:1243	5.683453	2.04579E-08	5.44E-06	up	hsa circ 0085465	circBase	NM 0141	ATAD2	exonic	333
chr4	39301921	39302034	diffreps p	5.472228	113	chr4:3930	2.090432	3.3711E-06	0.000263	up	hsa circ 0008596	circBase	NM 0029	RFC1	exonic	698
chr2	128754061	128754065	diffreps p	5.686643	4	chr2:1287	3.366483	2.05758E-06	0.000176	up	hsa circ 0056390	circBase	NM 0245	SAP130	exonic	264
chr7	134165021	134165280	diffreps p	6.072747	259	chr7:1341	2.775472	8.45771E-07	8.77E-05	up		novel	NM 0202	AKR1B10	sense overlapping	52840
chr7	134242341	134242820	diffreps p	8.034579	479	chr7:1342	4.390244	9.23467E-09	2.98E-06	up		novel	NM 0202	AKR1B10	sense overlapping	35032
chr5	32134801	32135080	diffreps p	5.107389	279	chr5:3212	69.3	7.80928E-06	0.000516	up	hsa circ 0128830	circBase	NM 0221	GOLPH3	sense overlapping	22386
chr1	63906650	63906820	diffreps p	7.144297	170	chr1:6390	2.340801	7.17303E-08	1.36E-05	up		novel	NM 0142	ITGB3BP	sense overlapping	13989
chr17	41222944	41223140	diffreps p	6.002603	196	chr17:412	2.206621	9.94024E-07	9.93E-05	up	hsa circ 0043954	circBase	NM 0072	BRCA1	exonic	590
chr8	124346439	124346562	diffreps p	7.613595	123	chr8:1243	2.589071	2.43447E-08	6.19E-06	up	hsa circ 0085456	circBase	NM 0141	ATAD2	exonic	1148
chr1	44878721	44878960	diffreps p	5.118117	239	chr1:4487	71.9	7.61874E-06	0.000506	up		novel	NM 0181	RNF220	sense overlapping	5640
chr14	45706850	45706924	diffreps p	6.730083	74	chr14:457	2.000589	1.86173E-07	2.75E-05	up	hsa circ 0101888	circBase	NM 0183	MIS18BP1	exonic	451
chr1	47728574	47728740	diffreps p	8.163248	166	chr1:4772	2.891162	6.86676E-09	2.38E-06	up	hsa circ 0113481	circBase	NM 0030	STIL	exonic	860
chr2	215634041	215634280	diffreps p	5.553537	239	chr2:2155	3.189498	2.79552E-06	0.000226	up	hsa circ 0119087	circBase	NM 0004	BARD1	sense overlapping	68200
chr8	48259241	48260920	diffreps p	8.892461	1679	chr8:4823	3.387613	1.28097E-09	6.42E-07	up		novel	ENST000	SPIDR	intronic	81039
chr12	104970041	104970560	diffreps p	5.596549	519	chr12:104	11.12	2.53192E-06	0.000208	up		novel	ENST000	CHST11	intronic	35244
chr11	17982741	17983420	diffreps p	8.337922	679	chr11:179	167.1	4.5928E-09	1.74E-06	up	hsa circ 0095511	circBase	NM 0121	SERGEF	sense overlapping	50670
chr13	50574441	50574820	diffreps p	5.175668	379	chr13:505	63.8	6.67317E-06	0.000455	up		novel	NR 00261	DLEU2	sense overlapping	53909
chr14	45587401	45587600	diffreps p	5.267839	199	chr14:455	2.383037	5.39711E-06	0.000384	up	hsa circ 0101874	circBase	NM 0020	FKBP3	sense overlapping	12763
chr3	56661609	56661769	diffreps p	6.223876	160	chr3:5666	2.007252	5.97205E-07	6.71E-05	up	hsa circ 0001314	circBase	NM 0152	FAM208A	exonic	392
chr20	33362561	33362960	diffreps p	5.522871	399	chr20:333	12.21519	3.00005E-06	0.000239	up		novel	NM 0140	NCOA6	sense overlapping	21353
chr3	160150813	160150997	diffreps p	6.642985	184	chr3:1601	2.447376	2.27518E-07	3.21E-05	up		novel	NM 0054	SMC4	exonic	2043
chr1	47735306	47735400	diffreps p	8.768202	94	chr1:4772	2.823287	1.70529E-09	8.05E-07	up	hsa circ 0113481	circBase	NM 0030	STIL	exonic	860
chrX	153287358	153287400	diffreps p	10.06862	42	chrX:1532	2.511251	8.5385E-11	8.59E-08	up		novel	NM 0049	MECP2	sense overlapping	284
chr6	24509681	24512540	diffreps p	11.07739	2859	chr6:2450	2.324592	8.36772E-12	2.47E-08	up		novel	NM 0010	ALDH5A1	sense overlapping	31165
chr17	7515581	7515940	diffreps p	6.075918	359	chr17:750	9.744681	8.39618E-07	8.73E-05	up		novel	NM 0048	FXR2	sense overlapping	10133
chr12	78400198	78401225	diffreps p	6.548329	1027	chr12:784	2.179446	2.82925E-07	3.78E-05	up	hsa circ 0002405	circBase	NM 0149	NAV3	exonic	1143
chr12	104978261	104979200	diffreps p	10.39478	939	chr12:104	4.039578	4.0292E-11	5.47E-08	up		novel	ENST000	CHST11	intronic	35244
chr2	215616901	215617500	diffreps p	8.25834	599	chr2:2155	2.444564	5.51646E-09	2.01E-06	up	hsa circ 0119087	circBase	NM 0004	BARD1	sense overlapping	68200
chr4	22470121	22470500	diffreps p	5.126624	379	chr4:2246	19.74359	7.47096E-06	0.000498	down		novel	NM 1452	ADGRA3	sense overlapping	16497
chr14	55148652	55149060	diffreps p	6.513297	408	chr14:551	2.575422	3.06692E-07	4.03E-05	down	hsa circ 0003301	circBase	NM 0155	SAMD4A	sense overlapping	20646
chr1	241751441	241751700	diffreps p	6.760199	259	chr1:2417	2.275117	1.737E-07	2.62E-05	down		novel	ENST000	OPN3	sense overlapping	7215
chr20	732453	732500	diffreps p	8.529944	47	chr20:732	2.685125	2.95159E-09	1.24E-06	down		novel	T212270	G049020	antisense	975
chr3	194331644	194331749	diffreps p	7.311948	105	chr3:1943	2.165642	4.87586E-08	1.02E-05	down	hsa circ 0123156	circBase	ENST000	TMEM44	exonic	500
chr2	24786941	24787160	diffreps p	6.527311	219	chr2:2475	5.058161	2.96954E-07	3.92E-05	down	hsa circ 0119553	circBase	NM 0037	NCOA1	sense overlapping	47846
chr7	134244501	134244860	diffreps p	7.678435	359	chr7:1342	109.1544	2.09684E-08	5.54E-06	down		novel	NM 0202	AKR1B10	sense overlapping	35032
chr2	31587023	31587110	diffreps p	5.299661	87	chr2:3158	2.425494	5.01579E-06	0.000361	down		novel	NM 0003	XDH	exonic	2325
chr5	56160560	56160640	diffreps p	6.091365	80	chr5:5616	2.000944	8.1028E-07	8.49E-05	down	hsa circ 0001485	circBase	NM 0059	MAP3K1	exonic	467
chr1	10221321	10221344	diffreps p	6.367082	23	chr1:1020	4.98	4.29455E-07	5.23E-05	down	hsa circ 0009701	circBase	NM 0060	UBE4B	exonic	607
chr9	100787121	100787700	diffreps p	5.114022	579	chr9:1007	3.373591	7.69092E-06	0.00051	down	hsa circ 0004036	circBase	T363596	G085884	sense overlapping	8163
chr2	86707341	86707485	diffreps p	6.017757	144	chr2:8670	2.041306	9.59937E-07	9.68E-05	down	hsa circ 0008804	circBase	NM 0184	KDM3A	exonic	788
chr4	148800181	148800380	diffreps p	6.150173	199	chr4:1487	2.250142	7.07663E-07	7.64E-05	down	hsa circ 0125473	circBase	NM 0246	ARHGAP10	sense overlapping	27257
chr6	146212101	146212680	diffreps p	5.42977	579	chr6:1461	6.993243	3.71732E-06	0.000284	down	hsa circ 0130934	circBase	NM 1730	SHPRH	sense overlapping	30590
chr4	41673570	41673611	diffreps p	8.083212	41	chr4:4166	2.061736	8.25635E-09	2.73E-06	down	hsa circ 0126380	circBase	NM 0011	LIMCH1	exonic	1267
chr8	19597621	19598380	diffreps p	5.249112	759	chr8:1956	6.385135	5.63492E-06	0.000397	down		novel	ENST000	CSGALNACT1	intronic	48466
chr17	15519021	15519123	diffreps p	5.466272	102	chr17:155	2.043457	3.41766E-06	0.000266	down		novel	NM 0063	CDRT1	exonic	617
chr2	233677100	233677201	diffreps p	5.453564	101	chr2:2336	2.062766	3.51914E-06	0.000272	down		novel	NM 0155	GIGYF2	exonic	1934
chr8	19572341	19572780	diffreps p	7.484199	439	chr8:1956	2.873523	3.27945E-08	7.68E-06	down		novel	ENST000	CSGALNACT1	intronic	48466
chr19	57776759	57776780	diffreps p	5.128981	21	chr19:577	3.246964	7.43052E-06	0.000496	down		novel	T181957	G041860	intronic	203
chr8	119108521	119108740	diffreps p	5.709596	219	chr8:1190	24.02632	1.95166E-06	0.000169	down		novel	ENST000	EXT1	intronic	89028
chr6	72101641	72101960	diffreps p	6.893052	319	chr6:7209	2.491502	1.27923E-07	2.08E-05	down		novel	ENST000	LINC00472	intronic	17637
chr4	113181921	113182018	diffreps p	6.463004	97	chr4:1131	2.070195	3.44347E-07	4.4E-05	down	hsa circ 0125024	circBase	NM 0185	AP1AR	exonic	199
chr2	233680346	233680447	diffreps p	7.215836	101	chr2:2336	2.015848	6.08364E-08	1.2E-05	down		novel	NM 0155	GIGYF2	exonic	1934
chr7	134247921	134248300	diffreps p	6.931613	379	chr7:1342	16.83189	1.17054E-07	1.96E-05	down		novel	NM 0202	AKR1B10	sense overlapping	35032
chr5	167880972	167881105	diffreps p	7.342071	133	chr5:1678	2.0105	4.54914E-08	9.69E-06	down		novel	NM 0152	WWC1	exonic	2283
chr11	18001321	18001920	diffreps p	7.540966	599	chr11:179	80.4	2.87762E-08	6.98E-06	down	hsa circ 0095511	circBase	NM 0121	SERGEF	sense overlapping	50670
chr12	124907046	124907101	diffreps p	6.937678	55	chr12:124	2.064308	1.15431E-07	1.94E-05	down	hsa circ 0029309	circBase	NM 0063	NCOR2	exonic	667
chr1	234538741	234539200	diffreps p	5.7385	459	chr1:2345	67.7	1.826E-06	0.000161	down		novel	NM 0056	TARBP1	sense overlapping	71959

CircRNAI	logFC	logCPM	F	PValue	FDR	regulation	chrom	txStart	txEnd	strand	circBaseID	source	best_trans	GeneNam	Catalog	predicted_sequence_length
chr3:1361	7.689087	10.45646	3.125859	0.005944	0.330455	up	chr3	1.36E+08	1.36E+08	-	hsa_circ_0067478	circBase	NM_0058	STAG1	exonic	1520
chr13:761	7.536846	10.3247	2.90482	0.007807	0.330455	up	chr13	76134888	76143643	+	hsa_circ_0000494	circBase	NM_0060	UCHL3	exonic	420
chr17:511	7.435016	10.23834	2.750107	0.009465	0.330455	up	chr17	5117046	5117754	-		novel	NM_0012	SCIMP	intronic	708
chr2:2934	7.403011	10.21108	2.703515	0.010034	0.330455	up	chr2	29344239	29404563	+	hsa_circ_0119725	circBase	T187145	G042928	exonic	3092
chr6:7176	7.388318	10.19897	2.682868	0.010297	0.330455	up	chr6	7176887	7189555	+	hsa_circ_0001573	circBase	NM_0010	RREB1	exonic	709
chr7:1381	7.368418	10.18225	2.654433	0.010672	0.330455	up	chr7	1.38E+08	1.38E+08	+	hsa_circ_0082572	circBase	NM_0038	TRIM24	exonic	267
chr5:1456	7.345516	10.16305	2.631498	0.010984	0.330455	up	chr5	1.46E+08	1.46E+08	+	hsa_circ_0006087	circBase	NM_0189	RBM27	exonic	295
chr20:477	7.342789	10.16078	2.629664	0.01101	0.330455	up	chr20	47704545	47708664	+	hsa_circ_0060756	circBase	NM_0013	CSE1L	exonic	724
chr2:2047	7.326759	10.14695	2.618542	0.011165	0.330455	up	chr2	20478343	20527139	-	hsa_circ_0052852	circBase	NM_0153	PUM2	exonic	1975
chr13:965	7.319473	10.1413	2.613935	0.01123	0.330455	up	chr13	96577933	96638686	-		novel	NM_0201	UGGT2	exonic	1364
chr4:4093	7.319473	10.1413	2.613935	0.01123	0.330455	up	chr4	40936472	40973233	-		novel	NM_0043	APBB2	sense overl	36761
chr17:171	7.290845	10.11746	2.594641	0.011507	0.330455	up	chr17	17158172	17168295	-	hsa_circ_0042251	circBase	NM_0036	COPS3	exonic	582
chr6:5293	7.27168	10.10152	2.581719	0.011696	0.330455	up	chr6	52935854	52941341	+	hsa_circ_0003700	circBase	NM_0334	FBXO9	exonic	304
chr17:599	7.27168	10.10152	2.581719	0.011696	0.330455	up	chr17	59962261	59981946	-	hsa_circ_0045042	circBase	NM_0207	INTS2	exonic	908
chr7:1115	7.27168	10.10152	2.581719	0.011696	0.330455	up	chr7	1.12E+08	1.12E+08	-	hsa_circ_0005937	circBase	NM_0147	DOCK4	exonic	365
chr19:478	7.247176	10.08121	2.565219	0.011942	0.330455	up	chr19	47856010	47865950	+	hsa_circ_0109754	circBase	NM_0146	DHX34	exonic	1870
chr4:1289	7.196884	10.03972	2.538389	0.012354	0.330455	up	chr4	12891341	12891630	-	hsa_circ_0125284	circBase	T261428	G060119	exonic	289
chr16:877	7.147901	9.999534	2.512109	0.012773	0.330455	up	chr16	87782278	87795646	-	hsa_circ_0000724	circBase	NM_0175	KLHDC4	exonic	407
chr2:1666	7.144775	9.996991	2.510553	0.012798	0.330455	up	chr2	1.67E+08	1.67E+08	-		novel	ENST000	AC009495	intronic	11330
chr2:3902	7.13577	9.989034	2.505757	0.012876	0.330455	up	chr2	39029856	39046286	-		novel	NM_1989	DHX57	exonic	726
chr18:745	7.132616	9.98647	2.504183	0.012902	0.330455	up	chr18	74561481	74583781	+	hsa_circ_0001993	circBase	NM_0073	ZNF236	exonic	612
chr10:126	7.127094	9.981976	2.501422	0.012947	0.330455	up	chr10	1.26E+08	1.26E+08	-	hsa_circ_0000267	circBase	NM_0146	FAM53B	exonic	773
chr20:105	7.117997	9.975142	2.497142	0.013018	0.330455	up	chr20	10536878	10582467	+	hsa_circ_0059430	circBase	NM_0010	SLX4IP	exonic	378
chr12:123	7.112421	9.970617	2.494351	0.013064	0.330455	up	chr12	12339856	12356334	-	hsa_circ_0025491	circBase	NM_0023	LRP6	exonic	395
chr8:1311	7.088457	9.951363	2.482403	0.013264	0.330455	up	chr8	1.31E+08	1.31E+08	-	hsa_circ_0001824	circBase	NM_0184	ASAP1	exonic	334
chr11:867	7.080139	9.945461	2.478622	0.013328	0.330455	up	chr11	86778735	86802437	+	hsa_circ_0008359	circBase	NM_0229	TMEM13	exonic	255
chr20:135	7.071491	9.936758	2.473426	0.013416	0.330455	up	chr20	13539654	13561628	-	hsa_circ_0002001	circBase	NM_0177	TASP1	exonic	272
chr6:3583	7.06621	9.933084	2.471051	0.013457	0.330455	up	chr6	35836786	35840505	-	hsa_circ_0131657	circBase	NM_0031	SRPK1	exonic	927
chr2:5520	7.062935	9.93143	2.469883	0.013477	0.330455	up	chr2	55209650	55214834	-	hsa_circ_0001006	circBase	NM_0070	RTN4	exonic	347
chr17:155	7.043292	9.913943	2.457641	0.013688	0.330455	up	chr17	15516014	15519123	-		novel	NM_0063	CDRT1	exonic	617
chr12:692	7.043292	9.913943	2.457641	0.013688	0.330455	up	chr12	69210591	69218431	+	hsa_circ_0027491	circBase	NM_0023	MDM2	exonic	349
chr17:790	7.037919	9.910233	2.454385	0.013745	0.330455	up	chr17	79073727	79082309	+		novel	NM_0063	BAIAP2	exonic	1046
chr5:7079	7.034547	9.907529	2.45207	0.013786	0.330455	up	chr5	70797388	70808255	+	hsa_circ_0129474	circBase	NM_0184	BDP1	exonic	2291
chr22:480	7.034547	9.907529	2.45207	0.013786	0.330455	up	chr22	48081948	48082955	+	hsa_circ_0063865	circBase	NR_1220	LOC2849	exonic	436
chr3:1545	7.034547	9.907529	2.45207	0.013786	0.330455	up	chr3	15452751	15457449	-	hsa_circ_0122464	circBase	ENST000	METTL6	exonic	506
chr4:1782	7.019336	9.895227	2.441531	0.013972	0.330455	up	chr4	1.78E+08	1.78E+08	+	hsa_circ_0001460	circBase	NM_0182	NEIL3	exonic	596
chr14:316	7.011103	9.888016	2.435415	0.014081	0.330455	up	chr14	31613316	31641328	-	hsa_circ_0101612	circBase	ENST000	HECTD1	exonic	1622
chr14:103	7.005622	9.884267	2.432091	0.014141	0.330455	up	chr14	1.03E+08	1.03E+08	+	hsa_circ_0033426	circBase	NM_0033	TRAF3	exonic	1152
chr12:268	6.97818	9.861613	2.41259	0.014498	0.330455	up	chr12	26864105	26878698	-	hsa_circ_0098235	circBase	NM_0022	ITPR2	exonic	788
chr15:351	6.976105	9.860622	2.41163	0.014515	0.330455	up	chr15	35192822	35196706	-	hsa_circ_0103344	circBase	NM_0146	AQR	exonic	412
chr10:796	6.968503	9.853906	2.405887	0.014622	0.330455	up	chr10	79613111	79614128	-	hsa_circ_0006649	circBase	NM_0047	DLG5	exonic	328
chr14:361	6.954982	9.843583	2.396817	0.014793	0.330455	up	chr14	36121010	36143915	-		novel	NM_0149	RALGAP	exonic	1052
chrX:1003	6.944488	9.834717	2.389142	0.014939	0.330455	up	chrX	10031484	10066619	+	hsa_circ_0001910	circBase	NM_0156	WWC3	exonic	825
chr5:1678	6.939691	9.831604	2.386207	0.014995	0.330455	up	chr5	1.68E+08	1.68E+08	+		novel	NM_0152	WWC1	exonic	2283
chr20:356	6.939691	9.831604	2.386207	0.014995	0.330455	up	chr20	35693826	35696589	-	hsa_circ_0115145	circBase	NM_0028	RBL1	exonic	606
chr15:765	6.938022	9.831136	2.38565	0.015005	0.330455	up	chr15	76566752	76588078	-	hsa_circ_0000638	circBase	NM_0001	ETFA	exonic	777
chr17:549	6.918859	9.81502	2.37112	0.015287	0.330455	up	chr17	54926056	54934026	+	hsa_circ_0003108	circBase	NM_0036	DGKE	sense overl	7970
chr5:1679	6.915197	9.812133	2.368483	0.015339	0.330455	up	chr5	1.68E+08	1.68E+08	+	hsa_circ_0009145	circBase	NM_0028	RARS	exonic	324
chr8:1247	6.915197	9.812133	2.368483	0.015339	0.330455	up	chr8	1.25E+08	1.25E+08	+		novel	NM_1449	FAM91A1	exonic	342
chr7:2016	6.915197	9.812133	2.368483	0.015339	0.330455	up	chr7	20168013	20204022	-		novel	NM_1827	MACC1	sense overl	36009
chr17:181	6.908781	9.80707	2.363854	0.01543	0.330455	up	chr17	18177738	18178294	-		novel	NM_0046	TOP3A	sense overl	556
chrX:2482	6.886633	9.789364	2.347659	0.015754	0.330455	up	chrX	24828014	24861794	+	hsa_circ_0090162	circBase	NM_0169	POLA1	exonic	1083
chr9:2782	6.883751	9.787261	2.345698	0.015793	0.330455	up	chr9	2782755	2827151	-		novel	NM_0148	KIAA002	sense overl	44396
chr1:1511	6.883751	9.787261	2.345698	0.015793	0.330455	up	chr1	1.51E+08	1.51E+08	-	hsa_circ_0110803	circBase	NM_0059	VPS72	exonic	268
chr6:1111	6.874649	9.779367	2.338551	0.015939	0.330455	up	chr6	1.11E+08	1.11E+08	-		novel	NM_0013	CDK19	intronic	400
chr2:7040	6.874649	9.779367	2.338551	0.015939	0.330455	up	chr2	70402804	70409129	-	hsa_circ_0008220	circBase	NM_0178	C2orf42	exonic	1051
chr3:1943	6.851603	9.761951	2.322351	0.016274	0.330455	up	chr3	1.94E+08	1.94E+08	-	hsa_circ_0123156	circBase	ENST000	TMEM44	exonic	500
chr4:1838	6.851603	9.761951	2.322351	0.016274	0.330455	up	chr4	1.84E+08	1.84E+08	-	hsa_circ_0005274	circBase	NM_0019	DCTD	sense overl	24227
chr5:7738	6.83842	9.750873	2.312009	0.016492	0.330455	up	chr5	77385216	77412058	-	hsa_circ_0073092	circBase	NM_0036	AP3B1	exonic	609
chr6:1070	6.83842	9.750873	2.312009	0.016492	0.330455	up	chr6	10703622	10705077	+		novel	NM_0179	PAK1IP1	sense overl	1455
chr5:7738	6.83842	9.750873	2.312009	0.016492	0.330455	up	chr5	77385216	77461496	-	hsa_circ_0129692	circBase	NM_0036	AP3B1	exonic	1410
chr1:1467	6.822638	9.739229	2.299987	0.016749	0.330455	up	chr1	1.47E+08	1.47E+08	+	hsa_circ_0004220	circBase	NM_0042	CHD1L	exonic	449
chr8:6246	6.821831	9.739877	2.300431	0.016739	0.330455	up	chr8	62460629	62475439	-		novel	NM_0043	ASPH	exonic	464
chr3:1975	6.818724	9.736188	2.296872	0.016816	0.330455	up	chr3	1.98E+08	1.98E+08	+	hsa_circ_0123272	circBase	ENST000	LRCH3	exonic	1790
chr9:1100	6.818724	9.736188	2.296872	0.016816	0.330455	up	chr9	1.1E+08	1.1E+08	+	hsa_circ_0087859	circBase	NM_0028	RAD23B	exonic	968
chr6:1766	6.811864	9.730853	2.291404	0.016935	0.330455	up	chr6	17665469	17676001	-	hsa_circ_0075734	circBase	NM_0051	NUP153	exonic	881
chr3:1941	6.801259	9.721803	2.28226	0.017136	0.330455	up	chr3	1.94E+08	1.94E+08	-	hsa_circ_0001376	circBase	NM_0245	ATP13A3	exonic	520
chr7:1412	6.801259	9.721803	2.28226	0.017136	0.330455	up	chr7	1.41E+08	1.41E+08	+	hsa_circ_0082771	circBase	NM_0182	AGK	exonic	602
chr3:3161	6.797285	9.718722	2.279095	0.017206	0.330455	up	chr3	31617887	31621588	+	hsa_circ_0001278	circBase	NM_1788	STT3B	exonic	397
chr8:1243	6.782129	9.70804	2.267923	0.017456	0.330455	up	chr8	1.24E+08	1.24E+08	-	hsa_circ_0085473	circBase	NM_0141	ATAD2	exonic	333
chr3:1834	6.763115	9.692134	2.251745	0.017824	0.330455	up	chr3	1.83E+08	1.83E+08	+	hsa_circ_0008096	circBase	NM_0180	YEATS2	exonic	557
chr6:2450	6.763115	9.692134	2.251745	0.017824	0.330455	up	chr6	24502750	24533915	+		novel	NM_0010	ALDH5A1	sense overl	31165
chr12:110	6.763115	9.692134	2.251745	0.017824	0.330455	up	chr12	1.11E+08	1.11E+08	+	hsa_circ_0097240	circBase	NM_0140	IFT81	exonic	1062
chr14:542	6.763115	9.692134	2.251745	0.017824	0.330455	up	chr14	54216719	54235681	-		novel	T104615	G024669	exonic	262
chr8:9073	6.763115	9.692134	2.251745	0.017824	0.330455	up	chr8	90734242	90737869	-	hsa_circ_0008587	circBase	NR_1258	LOC1019	exonic	604
chr20:608	6.759035	9.688989	2.248498	0.017899	0.330455	up	chr20	60831112	60838747	+	hsa_circ_0115550	circBase	NM_0148	OSBPL2	exonic	350
chr15:521	6.754944	9.685838	2.245243	0.017974	0.330455	up	chr15	52161413	52194233	+	hsa_circ_0035292	circBase	NM_0145	TMOD3	exonic	898
chr12:104	6.751883	9.683471	2.242799	0.018031	0.330455	up	chr12	1.05E+08	1.05E+08	+		novel	NM_0033	TXNRD1	exonic	575
chr11:465	6.750628	9.683234	2.242422	0.01804	0.330455	up	chr11	46565524	46570050	-		novel	NM_0177	AMBRA1	exonic	738
chrX:6731	6.723933	9.661839	2.223924	0.018477	0.330455	up	chrX	67316711	67339174	-	hsa_circ_0090930	circBase	NM_0025	OPHN1	exonic	410
chr2:2253	6.715334	9.656006	2.220841	0.018551	0.330455	up	chr2	2.25E+08	2.25E+08	-	hsa_circ_0119239	circBase	NM_0035	CUL3	exonic	636
chr17:186	6.683643	9.630892	2.208246	0.018856	0.330455	up	chr17	18634382	18635447	+	hsa_circ_0042351	circBase	NM_0010	TRIM16L	exonic	262
chr18:453	6.679331	9.627613	2.206569	0.018897	0.330455	up	chr18	45377644	45396935	-	hsa_circ_0108532	circBase	NM_0059	SMAD2	exonic	548
chr19:194	6.679153	9.628253	2.206755	0.018893	0.330455	up	chr19	19439493	19440458	+		novel	NM_0153	MAU2	intronic	965
chr12:110	6.679153	9.628253	2.206755	0.018893	0.330455	up	chr12	1.11E+08	1.11E+08	+	hsa_circ_0097245	circBase	NM_0140	IFT81	exonic	697
chr9:1082	6.679153	9.628253	2.206755	0.018893	0.330455	up	chr9	1.08E+08	1.08E+08	+	hsa_circ_0137637	circBase	NM_0011	FSD1L	exonic	881
chr7:1341	6.679153	9.628253	2.206755	0.018893	0.330455	up	chr7	1.34E+08	1.34E+08	+		novel	NM_0202	AKR1B10	sense overl	55422
chr2:2475	6.679153	9.628253	2.206755	0.018893	0.330455	up	chr2	24759583	24807429	+	hsa_circ_0119553	circBase	NM_0037	NCOA1	sense overl	47846
chr14:103	6.658864	9.614092	2.199265	0.019077	0.330455	up	chr14	1.03E+08	1.03E+08	-	hsa_circ_0006809	circBase	NM_0060	CDC42BP	exonic	462
chr1:4619	6.646811	9.604981	2.194566	0.019194	0.330455	up	chr1	46193302	46195441	-	hsa_circ_0012275	circBase	NM_0058	IPP	exonic	324
chr11:701	6.642196	9.599265	2.192014	0.019257	0.330455	up	chr11	70118278	70118542	+		novel	NM_0036	PPFIA1	exonic	264
chr14:658	6.642042	9.599953	2.192224	0.019252	0.330455	up	chr14	65898754	65922436	+	hsa_circ_0008468	circBase	NM_1781	FUT8	intronic	23682
chr1:3327	6.634289	9.594092	2.189189	0.019328	0.330455	up	chr1	33276191	33276658	-	hsa_circ_0113072	circBase	NM_0036	YARS	exonic	323
chr3:1382	6.629977	9.590029	2.187225	0.019377	0.330455	up	chr3	1.38E+08	1.38E+08	-	hsa_circ_0002468	circBase	NM_0244	CEP70	exonic	396
chr17:174	6.608488	9.574489	2.178512	0.019598	0.330455	up	chr17	1746096	1747980	+	hsa_circ_0005946	circBase	NM_0029	RPA1	exonic	239
chr2:2914	6.599523	9.566926	2.174417	0.019702	0.330455	up	chr2	29140758	29152576	+	hsa_circ_0119719	circBase	NM_0151	WDR43	exonic	691
chr3:1495	6.599523	9.566926	2.174417	0.019702	0.330455	up	chr3	1.5E+08	1.5E+08	+	hsa_circ_0003502	circBase	NM_0072	RNF13	exonic	622
chrX:4648	6.599523	9.566926	2.174417	0.019702	0.330455	up	chrX	46480411	46513140	-	hsa_circ_0140329	circBase	ENST000	SLC9A7	intronic	32729
chr22:241	6.599523	9.566926	2.174417	0.019702	0.330455	up	chr22	24130723	24131167	+		novel	ENST000	SMARCB	intronic	444
chr4:1488	6.599523	9.566926	2.174417	0.019702	0.330455	up	chr4	1.49E+08	1.49E+08	+	hsa_circ_0007265	circBase	NM_0246	ARHGAP	exonic	488
chr3:1013	6.599523	9.566926	2.174417	0.019702	0.330455	up	chr3	1.01E+08	1.01E+08	-	hsa_circ_0003179	circBase	NM_0144	ZBTB11	exonic	468
chr20:458	6.594954	9.563499	2.172489	0.019752	0.330455	up	chr20	45891031	45927616	-		novel	NM_1830	ZMYND8	exonic	1312
chr2:2042	6.586938	9.557482	2.169102	0.019839	0.330455	up	chr2	2.04E+08	2.04E+08	+	hsa_circ_0118828	circBase	NM_0057	ABI2	exonic	706
chr9:1263	6.586107	9.559047	2.16959	0.019826	0.330455	up	chr9	1.26E+08	1.26E+08	-		novel	ENST000	DENND1	intronic	5372
chr19:907	6.564826	9.541623	2.160013	0.020075	0.330455	up	chr19	9074939	9075455	-		novel	NM_0246	MUC16	sense overl	516
chr11:111	6.564826	9.541623	2.160013	0.020075	0.330455	up	chr11	1.11E+08	1.11E+08	+		novel	NM_0151	SIK2	sense overl	13708
chr10:575	6.556648	9.535522	2.15655	0.020166	0.330455	up	chr10	5754356	5768920	+		novel	uc021pmn	C10orf18	sense overl	14564
chr10:738	6.556648	9.535522	2.15655	0.020166	0.330455	up	chr10	73887839	73921432	-		novel	NR_0455	ASCC1	exonic	468
chr16:319	6.55555	9.533843	2.15575	0.020187	0.330455	up	chr16	31926873	31927041	+		novel	NM_0034	ZNF267	sense overl	168
chr2:2392	6.55555	9.533843	2.15575	0.020187	0.330455	up	chr2	2.39E+08	2.39E+08	+		novel	NM_0156	TRAF3IP1	sense overl	51785
chr10:102	6.524611	9.511545	2.142896	0.020527	0.330455	up	chr10	1.03E+08	1.03E+08	+	hsa_circ_0006654	circBase	NM_0181	SLF2	exonic	1069
chr14:660	6.524611	9.511545	2.142896	0.020527	0.330455	up	chr14	66028054	66043389	+		novel	ENST000	FUT8	intronic	15335
chr9:9405	6.524611	9.511545	2.142896	0.020527	0.330455	up	chr9	94058302	94089985	-	hsa_circ_0139327	circBase	NM_0016	AUH	sense overl	31683
chrX:1499	6.524611	9.511545	2.142896	0.020527	0.330455	up	chrX	1.5E+08	1.5E+08	-	hsa_circ_0001948	circBase	NM_0314	CD99L2	exonic	366
chr1:9398	6.522736	9.511593	2.142667	0.020533	0.330455	up	chr1	93987638	94014983	+	hsa_circ_0013209	circBase	NM_0177	FNBP1L	exonic	1337
chr17:356	6.516203	9.505316	2.139297	0.020624	0.330455	up	chr17	35687112	35687365	-	hsa_circ_0043269	circBase	NM_1988	ACACA	exonic	253
chr9:1148	6.510195	9.499981	2.136118	0.02071	0.330455	up	chr9	1.15E+08	1.15E+08	-	hsa_circ_0137729	circBase	NM_0224	SUSD1	exonic	798
chr17:154	6.510195	9.499981	2.136118	0.02071	0.330455	up	chr17	15496650	15510997	-		novel	NM_0063	CDRT1	exonic	884

chrX:7954	6.510195	9.499981	2.136118	0.02071	0.330455	up	chrX	79544404	79565732	-	hsa_circ_0140637	circBase	NR_11064	CHMP1B2	exonic	562
chr1:1554	6.510195	9.499981	2.136118	0.02071	0.330455	up	chr1	1.55E+08	1.55E+08	-		PMID:250	NM_0184	ASH1L	sense overl	8687
chr12:800	6.510195	9.499981	2.136118	0.02071	0.330455	up	chr12	80007354	80060411	-		novel	NM_0025	PAWR	sense overl	53057
chr7:1585	6.496807	9.49009	2.129916	0.020878	0.330455	up	chr7	1.59E+08	1.59E+08	-	hsa_circ_0083220	circBase	NM_0207	ESYT2	exonic	473
chr11:781	6.489974	9.487264	2.12775	0.020937	0.330455	up	chr11	78180292	78204241	-	hsa_circ_0023812	circBase	NM_0246	NARS2	exonic	337
chr1:4487	6.483243	9.480823	2.123962	0.021041	0.330455	up	chr1	44876255	44881895	+		novel	NM_0181	RNF220	sense overl	5640
chr4:3984	6.463368	9.465301	2.11433	0.021307	0.330455	up	chr4	39843567	39851272	-	hsa_circ_0126287	circBase	NM_0011	PDS5A	exonic	571
chr12:646	6.463368	9.465301	2.11433	0.021307	0.330455	up	chr12	6463603	6471407	-		novel	NM_0010	SCNN1A	exonic	676
chr9:7868	6.463368	9.465301	2.11433	0.021307	0.330455	up	chr9	78682870	78686814	+	hsa_circ_0087232	circBase	NM_0062	PCSK5	exonic	262
chr17:418	6.463368	9.465301	2.11433	0.021307	0.330455	up	chr17	4186092	4200109	-	hsa_circ_0003239	circBase	NM_0033	UBE2G1	exonic	401
chr22:286	6.463368	9.465301	2.11433	0.021307	0.330455	up	chr22	28692185	28693840	-	hsa_circ_0003618	circBase	NM_0011	TTC28	exonic	404
chr20:388	6.463368	9.465301	2.11433	0.021307	0.330455	up	chr20	3888572	3891477	+	hsa_circ_0115199	circBase	NM_0249	PANK2	exonic	607
chr3:1304	6.463368	9.465301	2.11433	0.021307	0.330455	up	chr3	1.3E+08	1.3E+08	-		novel	NM_0146	PIK3R4	exonic	1276
chr15:757	6.463368	9.465301	2.11433	0.021307	0.330455	up	chr15	75703832	75705386	-	hsa_circ_0036353	circBase	NM_0154	SIN3A	exonic	535
chr16:705	6.458349	9.461627	2.112004	0.021372	0.330455	up	chr16	70566381	70578436	+	hsa_circ_0005330	circBase	NM_0124	SF3B3	exonic	759
chr8:1824	6.458349	9.461627	2.112004	0.021372	0.330455	up	chr8	1824736	1893821	+		novel	NM_0146	ARHGEF1	exonic	2718
chr4:1704	6.458349	9.461627	2.112004	0.021372	0.330455	up	chr4	1.7E+08	1.71E+08	-	hsa_circ_0125768	circBase	NM_0122	NEK1	exonic	708
chr18:125	6.453313	9.457943	2.10967	0.021437	0.330455	up	chr18	12506475	12535600	-	hsa_circ_0000829	circBase	NM_0201	SPIRE1	exonic	369
chr10:101	6.449539	9.455171	2.107914	0.021486	0.330455	up	chr10	1.02E+08	1.02E+08	+		novel	ENST000	ABCC2	exonic	373
chr19:494	6.440654	9.44943	2.104138	0.021592	0.330455	up	chr19	49488717	49489292	-	hsa_circ_0002296	circBase	NM_0021	GYS1	exonic	331
chr8:1313	6.42213	9.43733	2.096193	0.021818	0.330455	up	chr8	1.31E+08	1.31E+08	-	hsa_circ_0003691	circBase	NM_0184	ASAP1	exonic	213
chr2:2336	6.42213	9.43733	2.096193	0.021818	0.330455	up	chr2	2.34E+08	2.34E+08	+		novel	NM_0155	GIGYF2	exonic	1934
chr6:8622	6.42213	9.43733	2.096193	0.021818	0.330455	up	chr6	86227473	86239993	-	hsa_circ_0008093	circBase	NM_0204	SNX14	exonic	458
chr1:2193	6.42213	9.43733	2.096193	0.021818	0.330455	up	chr1	2.19E+08	2.19E+08	+	hsa_circ_0004314	circBase	NM_1387	LYPLAL1	sense overl	25631
chr1:1450	6.41497	9.429764	2.091775	0.021944	0.330455	up	chr1	1.45E+08	1.45E+08	+		novel	NM_0012	NBPF9	intronic	6689
chr3:1835	6.41497	9.429764	2.091775	0.021944	0.330455	up	chr3	1.84E+08	1.84E+08	-		novel	NM_0186	PARL	exonic	286
chr14:766	6.41497	9.429764	2.091775	0.021944	0.330455	up	chr14	76633005	76647192	+	hsa_circ_0005267	circBase	NM_0179	GPATCH2	exonic	531
chr3:8940	6.41497	9.429764	2.091775	0.021944	0.330455	up	chr3	8940577	8944204	-		novel	NM_0201	RAD18	exonic	295
chr15:638	6.409781	9.425998	2.089367	0.022014	0.330455	up	chr15	63845913	63855207	+	hsa_circ_0008153	circBase	NM_0065	USP3	exonic	477
chr1:5295	6.409781	9.425998	2.089367	0.022014	0.330455	up	chr1	52959282	52975384	-	hsa_circ_0003632	circBase	NM_0152	ZCCHC11	exonic	436
chr9:8844	6.400671	9.419382	2.084895	0.022143	0.330455	up	chr9	88444377	88457794	+		novel	NR_02941	LOC3897	exonic	1046
chr12:568	6.400671	9.419382	2.084895	0.022143	0.330455	up	chr12	56826809	56828015	-	hsa_circ_0098834	circBase	NM_0039	TIMELES	exonic	592
chr10:983	6.39677	9.417334	2.083374	0.022187	0.330455	up	chr10	98303832	98336586	-	hsa_circ_0094647	circBase	NM_0201	TM9SF3	exonic	1083
chr10:822	6.39677	9.417334	2.083374	0.022187	0.330455	up	chr10	82266983	82269227	+	hsa_circ_0008856	circBase	NM_0309	TSPAN14	exonic	318
chrM:892	6.39677	9.417334	2.083374	0.022187	0.330455	up	chrM	8927	9190	+		novel	uc011mfi.	OK/SW-cl	sense overl	263
chr5:1451	6.39677	9.417334	2.083374	0.022187	0.330455	up	chr5	1.45E+08	1.45E+08	-	hsa_circ_0008132	circBase	NM_1384	PRELID2	exonic	672
chr15:607	6.365699	9.396179	2.070641	0.02256	0.330455	up	chr15	60734614	60737990	-	hsa_circ_0000606	circBase	NM_0246	ICE2	sense overl	3376
chr14:551	6.364891	9.393325	2.069382	0.022598	0.330455	up	chr14	55148652	55169298	+	hsa_circ_0003301	circBase	NM_0155	SAMD4A	sense overl	20646
chr9:1256	6.364891	9.393325	2.069382	0.022598	0.330455	up	chr9	1.26E+08	1.26E+08	-	hsa_circ_0088377	circBase	NM_0188	RC3H2	exonic	966
chr17:530	6.364891	9.393325	2.069382	0.022598	0.330455	up	chr17	53068185	53078244	+	hsa_circ_0107135	circBase	NM_1785	STXBP4	exonic	527
chr10:930	6.364891	9.393325	2.069382	0.022598	0.330455	up	chr10	930386	936769	-	hsa_circ_0004995	circBase	NM_0151	LARP4B	sense overl	6383
chr12:100	6.351507	9.384504	2.064145	0.022753	0.330455	up	chr12	1.01E+08	1.01E+08	+	hsa_circ_0006258	circBase	NM_0179	SCYL2	exonic	508
chr14:235	6.351507	9.384504	2.064145	0.022753	0.330455	up	chr14	23547359	23548247	-	hsa_circ_0031277	circBase	NM_0149	ACIN1	exonic	335
chr3:4710	6.35094	9.385592	2.06452	0.022742	0.330455	up	chr3	47108559	47127804	-		novel	NM_0141	SETD2	exonic	832
chr1:2464	6.35094	9.385592	2.06452	0.022742	0.330455	up	chr1	2.46E+08	2.46E+08	-	hsa_circ_0017306	circBase	NM_0227	SMYD3	exonic	303
chr12:954	6.350085	9.382679	2.063224	0.022781	0.330455	up	chr12	95475152	95502337	-		novel	NM_0183	FGD6	sense overl	27185
chr11:698	6.329121	9.369786	2.055383	0.023016	0.330455	up	chr11	698868	700707	+	hsa_circ_0020682	circBase	NM_1749	TMEM80	exonic	207
chr2:2254	6.32363	9.365877	2.053108	0.023085	0.330455	up	chr2	2.25E+08	2.25E+08	-		novel	ENST000	CUL3	exonic	293
chr12:104	6.315902	9.358812	2.049253	0.023202	0.330455	up	chr12	1.05E+08	1.05E+08	+		novel	NM_0033	TXNRD1	sense overl	48760
chr7:7410	6.313985	9.359005	2.049104	0.023207	0.330455	up	chr7	74103457	74105443	+	hsa_circ_0005619	circBase	NM_0015	GTF2I	exonic	243
chr4:3930	6.313985	9.359005	2.049104	0.023207	0.330455	up	chr4	39301640	39304771	-	hsa_circ_0008596	circBase	NM_0029	RFC1	exonic	698
chr5:1398	6.313008	9.355937	2.047712	0.023249	0.330455	up	chr5	1.4E+08	1.4E+08	+	hsa_circ_0007862	circBase	NM_0177	ANKHD1	exonic	687
chr18:272	6.313008	9.355937	2.047712	0.023249	0.330455	up	chr18	2724896	2752550	+	hsa_circ_0108171	circBase	NM_0152	SMCHD1	exonic	1743
chr10:121	6.313008	9.355937	2.047712	0.023249	0.330455	up	chr10	12123470	12136270	+	hsa_circ_0004630	circBase	NM_0187	DHTKD1	exonic	1204
chr1:2333	6.313008	9.355937	2.047712	0.023249	0.330455	up	chr1	2.33E+08	2.33E+08	-		novel	NM_0148	PCNXL2	sense overl	10769
chr6:5627	6.313008	9.355937	2.047712	0.023249	0.330455	up	chr6	562783	582689	-		novel	NM_0183	EXOC2	sense overl	19906
chr1:2319	6.313008	9.355937	2.047712	0.023249	0.330455	up	chr1	2.32E+08	2.32E+08	+	hsa_circ_0007848	circBase	NM_0186	DISC1	exonic	347
chr14:761	6.307441	9.351976	2.045398	0.02332	0.330455	up	chr14	76198680	76201632	+	hsa_circ_0008187	circBase	NM_0150	TTLL5	exonic	239
chr5:1322	6.307441	9.351976	2.045398	0.02332	0.330455	up	chr5	1.32E+08	1.32E+08	-	hsa_circ_0073904	circBase	NM_0144	AFF4	exonic	927
chr3:1698	6.304779	9.350905	2.044635	0.023344	0.330455	up	chr3	1.7E+08	1.7E+08	-		novel	NM_0249	PHC3	sense overl	17182
chr1:3178	6.304779	9.350905	2.044635	0.023344	0.330455	up	chr1	31782890	31821821	+	hsa_circ_0113034	circBase	NM_0165	ZCCHC17	exonic	619
chr17:154	6.304779	9.350905	2.044635	0.023344	0.330455	up	chr17	15443746	15458678	-	hsa_circ_0008005	circBase	ENST000	TVP23C	exonic	579
chr7:1163	6.297662	9.345013	2.041327	0.023445	0.330455	up	chr7	1.16E+08	1.16E+08	+	hsa_circ_0082002	circBase	NM_0002	MET	exonic	1214
chr13:330	6.291591	9.342897	2.039724	0.023495	0.330455	up	chr13	33016524	33018263	-	hsa_circ_0100263	circBase	NM_0012	N4BP2L2	exonic	1739
chr17:269	6.285956	9.338915	2.037549	0.023562	0.330455	up	chr17	26904767	26905310	-		novel	ENST000	RP11-192	intronic	543
chr3:1965	6.276057	9.331915	2.033721	0.023681	0.330455	up	chr3	1.97E+08	1.97E+08	+	hsa_circ_0004950	circBase	NM_0025	PAK2	exonic	467
chr2:2424	6.276057	9.331915	2.033721	0.023681	0.330455	up	chr2	2.42E+08	2.42E+08	+		novel	T211709	G048935	intronic	873
chr7:9192	6.276057	9.331915	2.033721	0.023681	0.330455	up	chr7	91924202	92000921	+	hsa_circ_0081012	circBase	NM_0190	ANKIB1	exonic	1707
chr5:1706	6.276057	9.331915	2.033721	0.023681	0.330455	up	chr5	1.71E+08	1.71E+08	+	hsa_circ_0003718	circBase	NM_0228	RANBP17	sense overl	22418
chr17:616	6.259191	9.31755	2.02624	0.023915	0.330455	up	chr17	61655830	61666599	+	hsa_circ_0107400	circBase	NR_0735	DCAF7	exonic	957
chr6:1674	6.259191	9.31755	2.02624	0.023915	0.330455	up	chr6	1.67E+08	1.67E+08	+	hsa_circ_0131305	circBase	ENST000	RP11-517	exonic	715
chr7:9869	6.253413	9.313484	2.024	0.023986	0.330455	up	chr7	98692996	98719680	-		novel	NM_0011	SMURF1	intronic	26684
chr5:3212	6.253413	9.313484	2.024	0.023986	0.330455	up	chr5	32121600	32143986	-	hsa_circ_0128830	circBase	NM_0221	GOLPH3	sense overl	22386
chr5:3899	6.247614	9.309406	2.021751	0.024057	0.330455	up	chr5	38991050	39003724	-	hsa_circ_0006157	circBase	NM_1527	RICTOR	exonic	388
chr1:2253	6.237106	9.304302	2.018564	0.024158	0.330455	up	chr1	2.25E+08	2.25E+08	+		novel	NM_0013	DNAH14	exonic	688
chr2:1056	6.237106	9.304302	2.018564	0.024158	0.330455	up	chr2	1.06E+08	1.06E+08	+		novel	NM_1826	MRPS9	exonic	354
chr10:705	6.212481	9.285401	2.008352	0.024485	0.330455	up	chr10	70508892	70509442	+	hsa_circ_0018555	circBase	NM_0182	CCAR1	exonic	292
chr2:2414	6.207522	9.283419	2.007014	0.024528	0.330455	up	chr2	2.41E+08	2.41E+08	-	hsa_circ_0006808	circBase	NM_0165	ANKMY1	exonic	662
chr1:2177	6.206521	9.28125	2.006039	0.02456	0.330455	up	chr1	2.18E+08	2.18E+08	-	hsa_circ_0002843	circBase	NM_0180	GPATCH2	exonic	325
chr1:2658	6.206521	9.28125	2.006039	0.02456	0.330455	up	chr1	26584087	26586293	+	hsa_circ_0000033	circBase	NM_0012	CEP85	exonic	438
chr5:9509	6.203288	9.278108	2.004429	0.024612	0.330455	up	chr5	95091099	95099324	+	hsa_circ_0007444	circBase	NM_0148	RHOBTB3	exonic	479
chr1:1739	6.203288	9.278108	2.004429	0.024612	0.330455	up	chr1	1.74E+08	1.74E+08	-	hsa_circ_0004927	circBase	ENST000	RC3H1	exonic	381
chr15:659	6.203288	9.278108	2.004429	0.024612	0.330455	up	chr15	65994642	65995346	-	hsa_circ_0007072	circBase	NM_0058	DENND4A	exonic	343
chr2:2418	6.203288	9.278108	2.004429	0.024612	0.330455	up	chr2	24181170	24199945	+	hsa_circ_0005970	circBase	NM_1817	UBXN2A	exonic	301
chr4:9220	6.203288	9.278108	2.004429	0.024612	0.330455	up	chr4	92201831	92202070	+		novel	ENST000	CCSER1	intronic	239
chr7:1519	6.203288	9.278108	2.004429	0.024612	0.330455	up	chr7	1.52E+08	1.52E+08	-	hsa_circ_0006784	circBase	NM_1706	KMT2C	exonic	910
chr7:1585	6.200212	10.57661	2.190112	0.019305	0.330455	up	chr7	1.59E+08	1.59E+08	-	hsa_circ_0001776	circBase	NM_0207	ESYT2	exonic	495
chr9:1149	6.197284	9.27393	2.002096	0.024688	0.330455	up	chr9	1.15E+08	1.15E+08	-		novel	NR_0360	MIR3134	intronic	15428
chr17:143	6.197284	9.27393	2.002096	0.024688	0.330455	up	chr17	1437392	1461853	-	hsa_circ_0041252	circBase	NM_0062	PITPNA	exonic	748
chr20:389	6.197073	9.276145	2.00296	0.02466	0.330455	up	chr20	3893104	3899443	+	hsa_circ_0115205	circBase	NM_0249	PANK2	exonic	427
chr11:467	6.197073	9.276145	2.00296	0.02466	0.330455	up	chr11	46776437	46812133	-		novel	NM_0010	CKAP5	exonic	3219
chr12:308	6.183384	9.266418	1.997556	0.024836	0.330455	up	chr12	30881581	30904067	-	hsa_circ_0098354	circBase	NM_0239	CAPRIN2	exonic	1362
chr2:1010	6.172758	9.259058	1.993387	0.024973	0.330455	up	chr2	10101174	10126410	+	hsa_circ_0052577	circBase	NM_1981	GRHL1	exonic	991
chr2:2478	6.155897	9.247424	1.986061	0.025216	0.330455	up	chr2	24786772	24807429	+	hsa_circ_0000983	circBase	NM_0037	NCOA1	sense overl	20657
chr1:9496	6.155897	9.247424	1.986061	0.025216	0.330455	up	chr1	94964157	94972198	+	hsa_circ_0006524	circBase	NM_0028	ABCD3	exonic	459
chr12:110	6.155897	9.247424	1.986061	0.025216	0.330455	up	chr12	1.11E+08	1.11E+08	-	hsa_circ_0005785	circBase	NM_0162	ANAPC7	exonic	1031
chr11:108	6.155897	9.247424	1.986061	0.025216	0.330455	up	chr11	1.08E+08	1.08E+08	+		novel	NM_0000	ATM	exonic	1543
chr12:113	6.155897	9.247424	1.986061	0.025216	0.330455	up	chr12	1.14E+08	1.14E+08	+	hsa_circ_0006632	circBase	NM_0179	TPCN1	exonic	794
chr1:5117	6.155897	9.247424	1.986061	0.025216	0.330455	up	chr1	51171457	51246716	-		novel	NM_0070	FAF1	sense overl	75259
chr10:697	6.154763	9.24511	1.984838	0.025257	0.330455	up	chr10	69785302	69804320	-	hsa_circ_0007113	circBase	NM_0156	HERC4	exonic	682
chr11:959	6.154763	9.24511	1.984838	0.025257	0.330455	up	chr11	9597434	9603225	+		novel	NM_0033	WEE1	exonic	712
chr3:1255	6.145132	9.237552	1.980205	0.025412	0.330455	up	chr3	1.26E+08	1.26E+08	-		novel	T249577	G057398	exonic	504
chr1:2356	6.145132	9.237552	1.980205	0.025412	0.330455	up	chr1	2.36E+08	2.36E+08	-	hsa_circ_0017069	circBase	NM_1524	B3GALNT	exonic	650
chr13:111	6.145132	9.237552	1.980205	0.025412	0.330455	up	chr13	1.12E+08	1.12E+08	+	hsa_circ_0002680	circBase	NM_0038	ARHGEF7	exonic	698
chr3:9486	6.145132	9.237552	1.980205	0.025412	0.330455	up	chr3	9486731	9487424	+	hsa_circ_0064139	circBase	NM_0012	SETD5	exonic	337
chr17:560	6.145132	9.237552	1.980205	0.025412	0.330455	up	chr17	56081011	56082402	-	hsa_circ_0107191	circBase	NM_0069	SRSF1	sense overl	1391
chr11:117	6.145132	9.237552	1.980205	0.025412	0.330455	up	chr11	1.17E+08	1.17E+08	+		novel	NM_0149	CEP164	exonic	862
chr20:164	6.145132	9.237552	1.980205	0.025412	0.330455	up	chr20	16474935	16496309	-	hsa_circ_0114744	circBase	NM_0247	KIF16B	exonic	1071
chr2:5844	6.138883	9.233256	1.977487	0.025503	0.330455	up	chr2	58449076	58459247	-	hsa_circ_0001009	circBase	NM_0180	FANCL	exonic	278
chr12:104	6.138478	10.52082	2.12317	0.021062	0.330455	up	chr12	1.05E+08	1.05E+08	+	hsa_circ_0007044	circBase	NM_0033	TXNRD1	exonic	327
chr5:1122	6.127896	9.225699	1.972701	0.025665	0.330455	up	chr5	1.12E+08	1.12E+08	+	hsa_circ_0127631	circBase	NM_0012	SRP19	sense overl	1436
chr9:7452	6.124574	9.225681	1.972349	0.025677	0.330455	up	chr9	74522736	74523046	-		novel	NM_0010	ABHD17B	intronic	310
chr14:551	6.11351	9.218113	1.967546	0.025841	0.330455	up	chr14	55168779	55169298	+	hsa_circ_0004846	circBase	NM_0155	SAMD4A	exonic	519
chr1:4609	6.11351	9.218113	1.967546	0.025841	0.330455	up	chr1	46099160	46126049	-		novel	NM_0216	GPBP1L1	sense overl	26889
chr15:852	6.11351	9.218113	1.967546	0.025841	0.330455	up	chr15	85223943	85234875	-	hsa_circ_0000647	circBase	NM_0143	SEC11A	exonic	380
chr2:2283	6.11351	9.218113	1.967546	0.025841	0.330455	up	chr2	2.28E+08	2.28E+08	+	hsa_circ_0058514	circBase	NM_0045	AGFG1	exonic	527
chr16:694	6.11351	9.218113	1.967546	0.025841	0.330455	up	chr16	69404385	69406258	-	hsa_circ_0105829	circBase	NM_0056	TERF2	exonic	234
chr3:1941	6.11351	9.218113	1.967546	0.025841	0.330455	up	chr3	1.94E+08	1.94E+08	-	hsa_circ_0005494	circBase	NM_0245	ATP13A3	exonic	525
chr3:5294	6.101079	9.208036	1.961374	0.026053	0.330455	up	chr3	52946553	52947633	-	hsa_circ_0066143	circBase	NM_0163	SFMBT1	exonic	250

chrX:1196	6.101079	9.208036	1.961374	0.026053	0.330455	up	chrX	1.2E+08	1.2E+08	-	hsa_circ_0139663	circBase	NM_0035	CUL4B	exonic	603
chr7:9966	6.101079	9.208036	1.961374	0.026053	0.330455	up	chr7	99665318	99666874	+		novel	T329419	G077223	intronic	1556
chr17:973	6.101079	9.208036	1.961374	0.026053	0.330455	up	chr17	973208	1003975	-	hsa_circ_0041187	circBase	NM_0010	ABR	exonic	770
chr10:696	6.084533	9.195816	1.953718	0.026318	0.330455	up	chr10	69695909	69726559	-		novel	NM_0156	HERC4	exonic	848
chr10:461	6.084533	9.195816	1.953718	0.026318	0.330455	up	chr10	46158989	46159290	-	hsa_circ_0018281	circBase	NM_1748	ZFAND4	exonic	301
chr1:1466	6.084533	9.195816	1.953718	0.026318	0.330455	up	chr1	1.47E+08	1.47E+08	-		novel	NM_0011	FMO5	exonic	867
chr20:614	6.084533	9.195816	1.953718	0.026318	0.330455	up	chr20	61485367	61491660	-	hsa_circ_0004926	circBase	NM_0013	TCFL5	exonic	730
chr1:1459	6.084533	9.195816	1.953718	0.026318	0.330455	up	chr1	1.46E+08	1.46E+08	+	hsa_circ_0008497	circBase	ENST000	GPR89C	exonic	186
chr4:4194	6.084533	9.195816	1.953718	0.026318	0.330455	up	chr4	41940618	41951402	+	hsa_circ_0126388	circBase	NM_0181	TMEM33	exonic	569
chr10:861	6.072716	9.18967	1.949516	0.026465	0.330455	up	chr10	86177526	86185649	+	hsa_circ_0006956	circBase	NM_0189	CCSER2	exonic	254
chr2:6157	6.069841	9.188188	1.9485	0.026501	0.330455	up	chr2	61570949	61577828	-	hsa_circ_0120633	circBase	NM_0147	USP34	exonic	1249
chr10:575	6.069841	9.188188	1.9485	0.026501	0.330455	up	chr10	5751492	5773166	+	hsa_circ_0093751	circBase	uc001iik.3	FAM208B	sense overl	21674
chr8:2801	6.048226	9.173359	1.938756	0.026845	0.330455	up	chr8	28013458	28019595	+	hsa_circ_0001785	circBase	NM_0180	ELP3	exonic	467
chr1:9279	6.028652	9.158589	1.928669	0.027207	0.330455	up	chr1	92798947	92846430	+	hsa_circ_0000091	circBase	NM_0248	RPAP2	exonic	383
chr1:1195	6.024807	9.157624	1.927888	0.027235	0.330455	up	chr1	1.2E+08	1.2E+08	-		novel	NM_0158	WARS2	sense overl	14429
chr11:110	6.024807	9.157624	1.927888	0.027235	0.330455	up	chr11	1.1E+08	1.1E+08	+	hsa_circ_0005348	circBase	NM_0333	ZC3H12C	exonic	752
chr11:743	6.024807	9.157624	1.927888	0.027235	0.330455	up	chr11	74315736	74331134	+	hsa_circ_0006656	circBase	NM_0065	POLD3	exonic	617
chr20:339	6.024807	9.157624	1.927888	0.027235	0.330455	up	chr20	33954359	33971936	-	hsa_circ_0004994	circBase	NM_0182	UQCC1	exonic	335
chr2:4418	6.021277	9.152829	1.925461	0.027323	0.330455	up	chr2	44184523	44209573	-		novel	NM_1332	LRPPRC	exonic	1500
chr2:1287	6.021277	9.152829	1.925461	0.027323	0.330455	up	chr2	1.29E+08	1.29E+08	-	hsa_circ_0056390	circBase	NM_0245	SAP130	exonic	264
chr1:6236	6.021277	9.152829	1.925461	0.027323	0.330455	up	chr1	62365254	62374154	+		novel	NM_1768	INADL	exonic	361
chr7:2355	6.021277	9.152829	1.925461	0.027323	0.330455	up	chr7	23552512	23562051	-	hsa_circ_0133976	circBase	NM_0012	TRA2A	exonic	790
chr17:441	6.021277	9.152829	1.925461	0.027323	0.330455	up	chr17	44159806	44172067	-	hsa_circ_0006669	circBase	NM_0154	KANSL1	exonic	244
chr9:1406	6.021277	9.152829	1.925461	0.027323	0.330455	up	chr9	1.41E+08	1.41E+08	+	hsa_circ_0001904	circBase	NM_0247	EHMT1	exonic	331
chr14:718	6.021277	9.152829	1.925461	0.027323	0.330455	up	chr14	71859913	71862977	+		novel	ENST000	SIPA1L1	intronic	3064
chr12:578	5.997358	9.139028	1.917192	0.027625	0.330455	up	chr12	57891939	57892406	+	hsa_circ_0027261	circBase	NM_0049	MARS	exonic	321
chr13:103	5.983473	9.127589	1.910856	0.027859	0.330455	up	chr13	1.03E+08	1.03E+08	+	hsa_circ_0099790	circBase	NM_0032	TPP2	exonic	476
chr17:953	5.978331	9.126391	1.909871	0.027895	0.330455	up	chr17	953289	1003975	-	hsa_circ_0007919	circBase	NM_0010	ABR	exonic	1545
chr1:4098	5.978331	9.126391	1.909871	0.027895	0.330455	up	chr1	40980186	40982858	+	hsa_circ_0113348	circBase	NM_0227	EXO5	sense overl	2672
chr17:290	5.978331	9.126391	1.909871	0.027895	0.330455	up	chr17	29086508	29096983	+		novel	ENST000	SUZ12P	exonic	460
chr14:501	5.955112	9.108515	1.899792	0.028272	0.330455	up	chr14	50131343	50133131	-	hsa_circ_0101921	circBase	NM_0026	POLE2	exonic	190
chr11:349	5.955112	9.108515	1.899792	0.028272	0.330455	up	chr11	34978930	35013926	+	hsa_circ_0095769	circBase	NM_0034	PDHX	exonic	905
chr6:1118	5.955112	9.108515	1.899792	0.028272	0.330455	up	chr6	1.12E+08	1.12E+08	-		novel	NM_1472	TRAF3IP2	exonic	529
chr14:102	5.947764	10.34912	1.900855	0.028232	0.330455	up	chr14	1.03E+08	1.03E+08	+	hsa_circ_0002553	circBase	NM_1812	WDR20	exonic	1443
chr4:5627	5.930306	9.094459	1.891315	0.028593	0.330455	up	chr4	56277780	56284152	+	hsa_circ_0001414	circBase	NM_0184	TMEM165	exonic	585
chr1:1149	5.930306	9.094459	1.891315	0.028593	0.330455	up	chr1	1.15E+08	1.15E+08	-	hsa_circ_0110541	circBase	NM_0159	TRIM33	exonic	707
chr3:1962	5.930306	9.094459	1.891315	0.028593	0.330455	up	chr3	1.96E+08	1.96E+08	-	hsa_circ_0123217	circBase	NM_1526	RNF168	exonic	257
chr1:5912	5.928235	9.090824	1.889544	0.028661	0.330455	up	chr1	59126841	59150923	-	hsa_circ_0113709	circBase	NM_0010	MYSM1	exonic	1929
chr11:130	5.926886	9.090682	1.889375	0.028667	0.330455	up	chr11	1.31E+08	1.31E+08	-		novel	NM_0147	SNX19	sense overl	8801
chr13:419	5.926886	9.090682	1.889375	0.028667	0.330455	up	chr13	41902859	41936295	+		novel	NM_0245	NAA16	exonic	848
chr12:294	5.926886	9.090682	1.889375	0.028667	0.330455	up	chr12	29492783	29494151	-	hsa_circ_0098305	circBase	NM_0165	ERGIC2	sense overl	1368
chr1:2251	5.926886	9.090682	1.889375	0.028667	0.330455	up	chr1	2.25E+08	2.25E+08	+	hsa_circ_0112179	circBase	NM_0013	DNAH14	exonic	2865
chr1:1516	5.926886	9.090682	1.889375	0.028667	0.330455	up	chr1	1.52E+08	1.52E+08	+	hsa_circ_0000130	circBase	NM_0309	SNX27	exonic	232
chr21:405	5.926886	9.090682	1.889375	0.028667	0.330455	up	chr21	40596333	40601362	-	hsa_circ_0001194	circBase	NM_0189	BRWD1	exonic	382
chr2:1206	5.913755	9.083353	1.885096	0.028831	0.330455	up	chr2	1.21E+08	1.21E+08	+	hsa_circ_0117169	circBase	NM_0028	PTPN4	exonic	2019
chr10:990	5.900962	9.075003	1.880383	0.029013	0.330455	up	chr10	99023176	99025903	-		novel	uc009xvk.	ARHGAP	intronic	2727
chrM:1692	5.893597	9.070221	1.877676	0.029118	0.330455	up	chrM	1691	3790	+		novel	T370381	G087361	intronic	2099
chr10:701	5.885766	9.062786	1.873723	0.029272	0.330455	up	chr10	70196767	70229920	-	hsa_circ_0018524	circBase	NR_1022	DNA2	exonic	1572
chr2:2398	5.885766	9.062786	1.873723	0.029272	0.330455	up	chr2	23985078	24046439	-	hsa_circ_0119423	circBase	NR_12571	ATAD2B	exonic	1644
chr3:1714	5.885766	9.062786	1.873723	0.029272	0.330455	up	chr3	1.71E+08	1.71E+08	-		novel	NM_0026	PLD1	exonic	318
chr3:1957	5.885766	9.062786	1.873723	0.029272	0.330455	up	chr3	1.96E+08	1.96E+08	-	hsa_circ_0068610	circBase	NM_0032	TFRC	exonic	209
chr6:1604	5.885766	9.062786	1.873723	0.029272	0.330455	up	chr6	1.6E+08	1.6E+08	+	hsa_circ_0131235	circBase	ENST000	IGF2R	exonic	611
chr7:4460	5.885766	9.062786	1.873723	0.029272	0.330455	up	chr7	44609582	44610476	-		novel	uc010kyh.	DDX56	exonic	265
chr1:7842	5.885766	9.062786	1.873723	0.029272	0.330455	up	chr1	78425868	78433350	-	hsa_circ_0013034	circBase	NM_0039	FUBP1	exonic	1326
chr2:9661	5.885766	9.062786	1.873723	0.029272	0.330455	up	chr2	9661331	9666373	-		PMID:252	NM_0031	ADAM17	exonic	338
chr3:1865	5.885766	9.062786	1.873723	0.029272	0.330455	up	chr3	1.87E+08	1.87E+08	+		novel	NM_0019	EIF4A2	exonic	1004
chr12:275	5.885766	9.062786	1.873723	0.029272	0.330455	up	chr12	27521194	27524380	+	hsa_circ_0098256	circBase	NM_0201	ARNTL2	sense overl	3186
chr20:326	5.885766	9.062786	1.873723	0.029272	0.330455	up	chr20	32677321	32678408	-	hsa_circ_0008718	circBase	NM_0039	EIF2S2	sense overl	1087
chr10:103	5.885766	9.062786	1.873723	0.029272	0.330455	up	chr10	1.04E+08	1.04E+08	-	hsa_circ_0019609	circBase	NM_0122	MGEA5	exonic	1635
chr5:7312	5.880628	9.061797	1.872903	0.029304	0.330455	up	chr5	73128162	73136585	+	hsa_circ_0006969	circBase	NM_0010	ARHGEF2	exonic	403
chr1:7619	5.880628	9.061797	1.872903	0.029304	0.330455	up	chr1	76198328	76216231	+	hsa_circ_0114116	circBase	NM_0000	ACADM	exonic	827
chr5:1572	5.880628	9.061797	1.872903	0.029304	0.330455	up	chr5	1.57E+08	1.57E+08	-	hsa_circ_0074809	circBase	NM_0146	CLINT1	exonic	1028
chr14:352	5.880628	9.061797	1.872903	0.029304	0.330455	up	chr14	35254982	35264971	-		novel	NM_0134	BAZ1A	exonic	703
chr22:161	5.878297	9.057944	1.87097	0.02938	0.330455	up	chr22	16186118	16190791	-		novel	NR_12211	DUXAP8	sense overl	4673
chrX:1063	5.863811	9.049324	1.865981	0.029576	0.330455	up	chrX	1.06E+08	1.06E+08	-	hsa_circ_0091288	circBase	NM_0176	NUP62CL	exonic	252
chr17:780	5.863811	9.049324	1.865981	0.029576	0.330455	up	chr17	78022381	78024082	+	hsa_circ_0008677	circBase	NM_0179	CCDC40	exonic	483
chr10:783	5.850234	9.04188	1.861597	0.02975	0.330455	up	chr10	7839009	7844388	+		novel	NM_0051	ATP5C1	exonic	702
chr1:2333	5.850234	9.04188	1.861597	0.02975	0.330455	up	chr1	2.33E+08	2.33E+08	-	hsa_circ_0003314	circBase	NM_0148	PCNXL2	exonic	333
chr15:647	5.829177	9.028369	1.853868	0.030059	0.330455	up	chr15	64716229	64737307	+		novel	NM_0162	TRIP4	exonic	320
chr15:652	5.829177	9.028369	1.853868	0.030059	0.330455	up	chr15	65266939	65275931	-	hsa_circ_0003526	circBase	NM_0166	SPG21	exonic	476
chr16:157	5.829177	9.028369	1.853868	0.030059	0.330455	up	chr16	15766546	15771806	+		PMID:250	ENST000	NDE1	exonic	231
chr1:1803	5.829177	9.028369	1.853868	0.030059	0.330455	up	chr1	1.8E+08	1.8E+08	-	hsa_circ_0000164	circBase	NM_0323	ACBD6	exonic	279
chr1:4516	5.829177	9.028369	1.853868	0.030059	0.330455	up	chr1	45166850	45173643	-		novel	T009851	G002087	sense overl	6793
chr17:272	5.812918	9.015551	1.846663	0.03035	0.330455	up	chr17	27244302	27248826	-	hsa_circ_0042733	circBase	NM_0208	PHF12	exonic	419
chr2:7048	5.812918	9.015551	1.846663	0.03035	0.330455	up	chr2	70486491	70505006	+	hsa_circ_0120864	circBase	NM_0162	PCYOX1	sense overl	18515
chr2:5417	5.812918	9.015551	1.846663	0.03035	0.330455	up	chr2	54175557	54176420	-	hsa_circ_0120348	circBase	NM_0146	PSME4	exonic	258
chr7:3036	5.812918	9.015551	1.846663	0.03035	0.330455	up	chr7	30363257	30405911	+	hsa_circ_0134081	circBase	T320889	G075174	exonic	543
chr1:2037	5.812918	9.015551	1.846663	0.03035	0.330455	up	chr1	2.04E+08	2.04E+08	+		novel	NM_0148	ZC3H11A	exonic	1768
chr1:3564	5.805066	9.010549	1.843707	0.030471	0.330455	up	chr1	35643589	35653691	-	hsa_circ_0113147	circBase	ENST000	SFPQ	exonic	337
chr3:1950	5.797852	9.006735	1.841381	0.030566	0.330455	up	chr3	1.95E+08	1.95E+08	-	hsa_circ_0123172	circBase	NM_0122	ACAP2	exonic	543
chr8:1872	5.797852	9.006735	1.841381	0.030566	0.330455	up	chr8	18725183	18730243	-	hsa_circ_0136098	circBase	NM_0153	PSD3	exonic	1504
chr14:605	5.797656	9.007969	1.84199	0.030541	0.330455	up	chr14	60582436	60592541	+	hsa_circ_0000544	circBase	NM_0224	PCNXL4	exonic	1910
chr4:7974	5.779452	8.996132	1.835008	0.030828	0.330455	up	chr4	79747190	79780841	+	hsa_circ_0070191	circBase	NM_0175	BMP2K	exonic	809
chr1:2842	5.775823	8.994139	1.8338	0.030878	0.330455	up	chr1	28422439	28422819	-		novel	ENST000	SPCS2P4	sense overl	380
chr7:5079	5.775823	8.994139	1.8338	0.030878	0.330455	up	chr7	50793709	50823753	-	hsa_circ_0134432	circBase	ENST000	GRB10	intronic	30044
chr8:1192	5.775823	8.994139	1.8338	0.030878	0.330455	up	chr8	1.19E+08	1.19E+08	-		novel	ENST000	SAMD12	intronic	1556
chr14:233	5.775823	8.994139	1.8338	0.030878	0.330455	up	chr14	23364871	23374868	-		novel	NM_0181	RBM23	sense overl	9997
chr16:188	5.775823	8.994139	1.8338	0.030878	0.330455	up	chr16	18852886	18856973	-	hsa_circ_0006434	circBase	NM_0150	SMG1	exonic	700
chrX:1539	5.74921	8.977087	1.823678	0.0313	0.330455	up	chrX	1.54E+08	1.54E+08	+		novel	NR_11002	DKC1	exonic	865
chr14:660	5.736196	8.966704	1.817633	0.031555	0.330455	up	chr14	66082695	66096324	+	hsa_circ_0102436	circBase	NM_0044	FUT8	exonic	394
chr3:1360	5.736196	8.966704	1.817633	0.031555	0.330455	up	chr3	1.36E+08	1.36E+08	-	hsa_circ_0122063	circBase	NM_0058	STAG1	exonic	408
chr5:6604	5.736196	8.966704	1.817633	0.031555	0.330455	up	chr5	6604250	6605521	-	hsa_circ_0001466	circBase	NM_0177	NSUN2	exonic	356
chr1:1537	5.736196	8.966704	1.817633	0.031555	0.330455	up	chr1	1.54E+08	1.54E+08	+	hsa_circ_0006939	circBase	NM_0230	INTS3	exonic	574
chr12:112	5.736196	8.966704	1.817633	0.031555	0.330455	up	chr12	1.13E+08	1.13E+08	-	hsa_circ_0097400	circBase	NM_0011	HECTD4	exonic	522
chr20:303	5.728732	8.962838	1.815259	0.031655	0.330455	up	chr20	30380537	30382351	+	hsa_circ_0009098	circBase	NM_0121	TPX2	exonic	420
chr19:196	5.728732	8.962838	1.815259	0.031655	0.330455	up	chr19	19675741	19681642	-	hsa_circ_0050247	circBase	NM_0252	PBX4	exonic	732
chr11:773	5.72042	8.959066	1.812901	0.031756	0.330455	up	chr11	77386080	77396204	-		novel	NM_0165	RSF1	exonic	662
chr1:2228	5.72042	8.959066	1.812901	0.031756	0.330455	up	chr1	2.23E+08	2.23E+08	+		novel	NM_1985	MIA3	sense overl	356
chr15:431	5.72042	8.959066	1.812901	0.031756	0.330455	up	chr15	43120125	43170882	-	hsa_circ_0006297	circBase	NM_1735	TTBK2	exonic	604
chr4:7394	5.72042	8.959066	1.812901	0.031756	0.330455	up	chr4	73944358	73962989	-		novel	NM_0322	ANKRD17	exonic	2387
chr11:294	5.677846	8.932371	1.797172	0.032435	0.330455	up	chr11	29402788	29405659	+		PMID:250	T056445	G013057	sense overl	2871
chr17:473	5.664811	8.922871	1.79199	0.032662	0.330455	up	chr17	47389288	47396568	-		novel	NM_0148	ZNF652	sense overl	7280
chr18:294	5.662802	8.923107	1.792051	0.032659	0.330455	up	chr18	29487422	29497630	-	hsa_circ_0108236	circBase	NM_0149	TRAPPC8	exonic	1037
chr17:441	5.662802	8.923107	1.792051	0.032659	0.330455	up	chr17	44143902	44159908	-	hsa_circ_0044186	circBase	NM_0154	KANSL1	exonic	417
chr3:1195	5.662802	8.923107	1.792051	0.032659	0.330455	up	chr3	1.2E+08	1.2E+08	-	hsa_circ_0008797	circBase	NM_0020	GSK3B	exonic	420
chr6:1499	5.662802	8.923107	1.792051	0.032659	0.330455	up	chr6	1.5E+08	1.5E+08	-	hsa_circ_0078224	circBase	NM_0046	LATS1	exonic	873
chr10:346	5.662802	8.923107	1.792051	0.032659	0.330455	up	chr10	34636883	34673182	-	hsa_circ_0093588	circBase	NM_0196	PARD3	exonic	1337
chr1:6524	5.656134	8.917551	1.789035	0.032792	0.330455	up	chr1	65243305	65280523	+	hsa_circ_0012857	circBase	NM_0182	RAVER2	exonic	1574
chr11:129	5.656134	8.917551	1.789035	0.032792	0.330455	up	chr11	1.29E+08	1.29E+08	-		novel	ENST000	ARHGAP	intronic	2849
chr3:1212	5.656134	8.917551	1.789035	0.032792	0.330455	up	chr3	1.21E+08	1.21E+08	-	hsa_circ_0121608	circBase	NM_1994	POLQ	exonic	319
chr11:326	5.655164	8.916133	1.788282	0.032825	0.330455	up	chr11	32608557	32610681	+	hsa_circ_0095690	circBase	NM_0063	EIF3M	exonic	396
chr1:1554	5.655164	8.916133	1.788282	0.032825	0.330455	up	chr1	1.55E+08	1.55E+08	-	hsa_circ_0003247	circBase	NM_0184	ASH1L	exonic	844
chr21:406	5.655164	8.916133	1.788282	0.032825	0.330455	up	chr21	40641824	40646398	-	hsa_circ_0008466	circBase	NM_0189	BRWD1	exonic	385
chr21:436	5.655164	8.916133	1.788282	0.032825	0.330455	up	chr21	43691179	43706104	+		novel	NM_0049	ABCG1	exonic	687
chr10:769	5.655164	8.916133	1.788282	0.032825	0.330455	up	chr10	76978820	76980728	+	hsa_circ_0018911	circBase	NM_0033	VDAC2	exonic	434
chr11:127	5.646416	8.91078	1.785298	0.032957	0.330455	up	chr11	12785725	12785981	+	hsa_circ_0095328	circBase	NM_0219	TEAD1	exonic	256
chr14:755	5.64097	10.56893	1.883709	0.028885	0.330455	up	chr14	75513078	75516421	-	hsa_circ_0032649	circBase	NM_0143	MLH3	exonic	3343
chr5:6534	5.628142	10.06886	1.577542	0.043746	0.33431	up	chr5	65349233	65350779	+	hsa_circ_0072732	circBase	NM_0186	ERBB2IP	exonic	1546
chr21:171	5.608608	10.05237	1.567025	0.044386	0.33431	up	chr21	17163820	17205867	+		novel	NM_0133	USP25	exonic	1802

chr13:486	5.6028	8.886217	1.77584	0.03338	0.330455	up	chr13	48651288	48664554	-	hsa_circ_0004936	circBase	NM_014	MED4	sense overl	13266
chr2:1094	5.6028	8.886217	1.77584	0.03338	0.330455	up	chr2	1.09E+08	1.09E+08	+	hsa_circ_0116987	circBase	NM_1449	CCDC138	exonic	589
chr11:652	5.6028	8.886217	1.77584	0.03338	0.330455	up	chr11	65268321	65268664	+		novel	GSE61474	GSE61474	intronic	343
chr1:1747	5.6028	8.886217	1.77584	0.03338	0.330455	up	chr1	1747194	1756938	-	hsa_circ_0000008	circBase	NM_0020	GNB1	exonic	249
chr12:112	5.6028	8.886217	1.77584	0.03338	0.330455	up	chr12	1.13E+08	1.13E+08	-	hsa_circ_0097423	circBase	NM_0011	HECTD4	exonic	864
chr4:4077	5.6028	8.886217	1.77584	0.03338	0.330455	up	chr4	40776281	40800921	+	hsa_circ_0126352	circBase	ENST000	NSUN7	exonic	912
chr3:1835	5.6028	8.886217	1.77584	0.03338	0.330455	up	chr3	1.84E+08	1.84E+08	-	hsa_circ_0068253	circBase	NM_0186	PARL	exonic	703
chr14:355	5.6028	8.886217	1.77584	0.03338	0.330455	up	chr14	35568457	35579835	-	hsa_circ_0101741	circBase	NM_0179	PPP2R3C	exonic	520
chr19:582	5.592806	8.878253	1.773247	0.033497	0.330455	up	chr19	58287910	58291514	+	hsa_circ_0109985	circBase	uc002qqb.	ZNF587	intronic	3604
chr3:1279	5.588824	8.876273	1.772586	0.033527	0.330455	up	chr3	1.28E+08	1.28E+08	+	hsa_circ_0067209	circBase	NM_0219	EEFSEC	exonic	470
chr3:3355	5.583614	10.03135	1.553576	0.045219	0.33431	up	chr3	33557514	33580434	-	hsa_circ_0123548	circBase	NM_0150	CLASP2	exonic	706
chr1:2296	5.579684	8.87078	1.770761	0.03361	0.330455	up	chr1	2.3E+08	2.3E+08	-		novel	NM_0182	NUP133	exonic	306
chr15:592	5.569286	8.86371	1.768398	0.033717	0.330455	up	chr15	59204761	59205895	-	hsa_circ_0003713	circBase	NM_0247	SLTM	exonic	246
chr9:3352	5.569286	8.86371	1.768398	0.033717	0.330455	up	chr9	33528724	33572367	+		novel	NM_0012	ANKRD1	sense overl	43643
chr12:784	5.564213	10.01506	1.543123	0.045879	0.33431	up	chr12	78400198	78415642	+	hsa_circ_0002405	circBase	NM_0149	NAV3	exonic	1143
chr5:3730	5.552869	10.00561	1.537041	0.046267	0.334959	up	chr5	37301538	37303516	-	hsa_circ_0003227	circBase	NM_1534	NUP155	exonic	399
chr10:946	5.540207	8.848345	1.763693	0.033932	0.330455	up	chr10	94653105	94659401	+	hsa_circ_0019170	circBase	NM_0190	EXOC6	exonic	357
chr1:2356	5.540207	8.848345	1.763693	0.033932	0.330455	up	chr1	2.36E+08	2.36E+08	-	hsa_circ_0112522	circBase	NM_1524	B3GALN	exonic	480
chr22:161	5.532251	9.988376	1.526257	0.046965	0.336344	up	chr22	16189031	16190791	-		novel	NR_12211	DUXAP8	exonic	338
chr1:2251	5.509215	9.969145	1.514379	0.047747	0.336436	up	chr1	2.25E+08	2.25E+08	+	hsa_circ_0016600	circBase	NM_0013	DNAH14	exonic	863
chr22:284	5.508606	8.828103	1.757969	0.034195	0.330455	up	chr22	28490067	28504391	-	hsa_circ_0116372	circBase	NM_0011	TTC28	exonic	2491
chr16:681	5.505848	9.966395	1.51267	0.047861	0.336436	up	chr16	68155889	68160513	+	hsa_circ_0000711	circBase	NM_0045	NFATC3	exonic	1298
chr7:2466	5.498953	8.822426	1.756351	0.03427	0.330455	up	chr7	24663284	24690331	+	hsa_circ_0001685	circBase	NM_0164	MPP6	exonic	653
chr7:1047	5.498953	8.822426	1.756351	0.03427	0.330455	up	chr7	1.05E+08	1.05E+08	+	hsa_circ_0132875	circBase	NM_0186	KMT2E	exonic	1287
chrM:1397	5.498953	8.822426	1.756351	0.03427	0.330455	up	chrM	13978	14150	+		novel	uc004cox.	MTND5	sense overl	172
chrX:5362	5.498953	8.822426	1.756351	0.03427	0.330455	up	chrX	53622146	53634660	-	hsa_circ_0005518	circBase	NM_0314	HUWE1	exonic	1061
chr11:179	5.491857	8.819449	1.755567	0.034306	0.330455	up	chr11	17981016	18031686	-	hsa_circ_0095511	circBase	NM_0121	SERGEF	sense overl	50670
chr5:1386	5.48214	9.946711	1.500456	0.048682	0.336878	up	chr5	1.39E+08	1.39E+08	+	hsa_circ_0008922	circBase	NM_1991	MATR3	exonic	161
chr1:5295	5.477962	8.809293	1.752511	0.034448	0.330455	up	chr1	52959282	52992045	-	hsa_circ_0113601	circBase	NM_0152	ZCCHC11	exonic	1411
chr11:349	5.477962	8.809293	1.752511	0.034448	0.330455	up	chr11	34978930	35006275	+	hsa_circ_0003768	circBase	NM_0034	PDHX	exonic	840
chr14:972	5.477962	8.809293	1.752511	0.034448	0.330455	up	chr14	97299803	97327072	+	hsa_circ_0000566	circBase	NM_0033	VRK1	exonic	1073
chr2:1906	5.477962	8.809293	1.752511	0.034448	0.330455	up	chr2	1.91E+08	1.91E+08	+	hsa_circ_0001083	circBase	NM_0005	PMS1	exonic	602
chr1:2229	5.477962	8.809293	1.752511	0.034448	0.330455	up	chr1	2.23E+08	2.23E+08	+	hsa_circ_0000187	circBase	ENST000	RP11-452	exonic	159
chr6:1930	5.474776	8.809436	1.752695	0.03444	0.330455	up	chr6	1930336	1949920	-		novel	NM_0015	GMDS	sense overl	19584
chr1:1552	5.474776	8.809436	1.752695	0.03444	0.330455	up	chr1	1.55E+08	1.55E+08	-	hsa_circ_0014528	circBase	NM_0039	CLK2	exonic	262
chr14:556	5.474776	8.809436	1.752695	0.03444	0.330455	up	chr14	55647930	55650471	-	hsa_circ_0000539	circBase	NM_0147	DLGAP5	exonic	342
chr3:1437	5.474776	8.809436	1.752695	0.03444	0.330455	up	chr3	1.44E+08	1.44E+08	+	hsa_circ_0006731	circBase	ENST000	C3orf58	intronic	4295
chr6:1100	5.474776	8.809436	1.752695	0.03444	0.330455	up	chr6	1.1E+08	1.1E+08	+	hsa_circ_0130294	circBase	NM_0148	FIG4	exonic	848
chrX:1175	5.474776	8.809436	1.752695	0.03444	0.330455	up	chrX	1.18E+08	1.18E+08	+		novel	NM_0190	WDR44	exonic	343
chr12:508	5.434245	8.785552	1.745687	0.034768	0.330455	up	chr12	50824277	50835426	+	hsa_circ_0003248	circBase	NM_0528	LARP4	exonic	482
chr3:4772	5.413435	8.772375	1.741909	0.034946	0.330455	up	chr3	47727538	47734790	-	hsa_circ_0065284	circBase	NM_0030	SMARCC	exonic	220
chr1:5978	5.413435	8.772375	1.741909	0.034946	0.330455	up	chr1	59787207	59844509	+	hsa_circ_0005354	circBase	NM_0182	FGGY	exonic	568
chr11:874	5.406236	8.769432	1.741432	0.034968	0.330455	up	chr11	8740406	8740569	+		novel	NM_0054	ST5	antisense	163
chr6:1363	5.406236	8.769432	1.741432	0.034968	0.330455	up	chr6	13639794	13644961	-	hsa_circ_0001578	circBase	NM_0054	RANBP9	exonic	598
chr1:1863	5.406236	8.769432	1.741432	0.034968	0.330455	up	chr1	1.86E+08	1.86E+08	+		novel	NM_0178	C1orf27	exonic	612
chr14:507	5.406236	8.769432	1.741432	0.034968	0.330455	up	chr14	50732075	50769735	-	hsa_circ_0102014	circBase	NM_0248	L2HGDH	exonic	1056
chr15:300	5.406236	8.769432	1.741432	0.034968	0.330455	up	chr15	30053341	30065560	-	hsa_circ_0034293	circBase	NM_0032	TJP1	exonic	926
chr9:1150	5.406236	8.769432	1.741432	0.034968	0.330455	up	chr9	1.15E+08	1.15E+08	-	hsa_circ_0003458	circBase	NM_0051	PTBP3	exonic	844
chr14:377	5.406236	8.769432	1.741432	0.034968	0.330455	up	chr14	37751481	37777724	+		novel	ENST000	MIPOL1	intronic	26243
chr19:109	5.380463	8.752723	1.737447	0.035158	0.330455	up	chr19	10930655	10935897	+		novel	NM_0049	DNM2	exonic	387
chr11:468	5.380463	8.752723	1.737447	0.035158	0.330455	up	chr11	46804823	46812133	-	hsa_circ_0095890	circBase	NM_0147	CKAP5	exonic	599
chr2:2421	5.380463	8.752723	1.737447	0.035158	0.330455	up	chr2	2.42E+08	2.42E+08	-		novel	NM_0053	HDLBP	exonic	573
chr12:501	5.380463	8.752723	1.737447	0.035158	0.330455	up	chr12	50157768	50157978	+		novel	NM_0032	TMBIM6	sense overl	210
chr10:706	5.380463	8.752723	1.737447	0.035158	0.330455	up	chr10	70696691	70703013	+	hsa_circ_0007097	circBase	NM_0240	DDX50	exonic	340
chr10:128	5.380463	8.752723	1.737447	0.035158	0.330455	up	chr10	1.29E+08	1.29E+08	+	hsa_circ_0020394	circBase	NM_0012	DOCK1	exonic	2399
chr3:1192	5.340461	8.730111	1.732257	0.035406	0.330455	up	chr3	1.19E+08	1.19E+08	+	hsa_circ_0001329	circBase	NM_0165	TIMMDC	exonic	323
chr6:8489	5.334278	8.728267	1.732187	0.035409	0.330455	up	chr6	84894904	84896341	-	hsa_circ_0077223	circBase	NM_0148	CEP162	exonic	554
chr9:7184	5.334278	8.728267	1.732187	0.035409	0.330455	up	chr9	71849354	71867816	+		novel	NM_0048	TJP2	exonic	1736
chr6:1589	5.334278	8.728267	1.732187	0.035409	0.330455	up	chr6	1.59E+08	1.59E+08	+	hsa_circ_0131215	circBase	NM_0208	TMEM18	exonic	1101
chr8:1311	5.329768	8.725135	1.731324	0.03545	0.330455	up	chr8	1.31E+08	1.31E+08	-	hsa_circ_0008934	circBase	NM_0184	ASAP1	exonic	550
chrX:3396	5.322526	8.720504	1.730099	0.035509	0.330455	up	chrX	33964960	33965517	+		novel	T372937	G087967	intronic	557
chr1:2119	5.315986	8.716444	1.729032	0.035561	0.330455	up	chr1	2.12E+08	2.12E+08	-	hsa_circ_0002274	circBase	NM_0148	LPGAT1	exonic	616
chr20:333	5.275892	8.693822	1.723397	0.035833	0.330455	up	chr20	33342898	33364251	-		novel	NM_0140	NCOA6	sense overl	21353
chr2:1206	5.275892	8.693822	1.723397	0.035833	0.330455	up	chr2	1.21E+08	1.21E+08	+	hsa_circ_0056236	circBase	NM_0028	PTPN4	exonic	341
chr17:305	5.275892	8.693822	1.723397	0.035833	0.330455	up	chr17	30509775	30521126	+	hsa_circ_0106606	circBase	NM_0183	RHOT1	exonic	540
chr3:1719	5.275892	8.693822	1.723397	0.035833	0.330455	up	chr3	1.72E+08	1.72E+08	+	hsa_circ_0067990	circBase	NM_0227	FNDC3B	exonic	553
chr2:1280	5.258542	8.685872	1.722396	0.035882	0.330455	up	chr2	1.28E+08	1.28E+08	-		novel	ENST000	MAP3K2	exonic	1110
chr20:179	5.258542	8.685872	1.722396	0.035882	0.330455	up	chr20	17936008	17937681	-	hsa_circ_0004816	circBase	NM_0144	SNX5	exonic	216
chrX:1297	5.258542	8.685872	1.722396	0.035882	0.330455	up	chrX	1.3E+08	1.3E+08	-		novel	NM_0063	ENOX2	exonic	669
chr10:126	5.258542	8.685872	1.722396	0.035882	0.330455	up	chr10	1.27E+08	1.27E+08	-	hsa_circ_0005418	circBase	NM_0013	CTBP2	exonic	263
chr3:1295	5.258542	8.685872	1.722396	0.035882	0.330455	up	chr3	1.3E+08	1.3E+08	-	hsa_circ_0121862	circBase	ENST000	TMCC1	exonic	340
chr9:9623	5.258542	8.685872	1.722396	0.035882	0.330455	up	chr9	96238537	96261132	+		novel	NM_0146	FAM120A	sense overl	22595
chr15:562	5.258542	8.685872	1.722396	0.035882	0.330455	up	chr15	56207481	56209109	-	hsa_circ_0103939	circBase	ENST000	NEDD4	intronic	1628
chr6:1499	5.225501	8.666673	1.718187	0.036088	0.330455	up	chr6	1.5E+08	1.5E+08	-	hsa_circ_0131022	circBase	NM_0046	LATS1	sense overl	2486
chr3:5781	5.225501	8.666673	1.718187	0.036088	0.330455	up	chr3	57817109	57857425	+	hsa_circ_0124360	circBase	NM_0013	SLMAP	exonic	1051
chr1:2132	5.199466	8.653399	1.715828	0.036203	0.330455	up	chr1	2.13E+08	2.13E+08	+	hsa_circ_0004849	circBase	NM_0124	RPS6KC1	exonic	331
chr14:542	5.178611	8.64217	1.713689	0.036309	0.330455	up	chr14	54206963	54235681	-		novel	ENST000	AL162759	sense overl	28718
chr8:1434	5.178611	8.64217	1.713689	0.036309	0.330455	up	chr8	1.43E+08	1.43E+08	-	hsa_circ_0004423	circBase	NM_1450	TSNARE1	exonic	805
chr6:8955	5.178611	8.64217	1.713689	0.036309	0.330455	up	chr6	89554075	89563483	-	hsa_circ_0077292	circBase	NM_0038	RNGTT	exonic	373
chr4:1861	5.178611	8.64217	1.713689	0.036309	0.330455	up	chr4	1.86E+08	1.86E+08	+	hsa_circ_0003237	circBase	NM_0319	SNX25	exonic	733
chr7:4367	5.163142	8.632387	1.711021	0.03644	0.330455	up	chr7	43679047	43680248	-	hsa_circ_0001700	circBase	NR_0477	COA1	exonic	310
chr14:924	5.129189	8.614396	1.707414	0.036619	0.330455	up	chr14	92482039	92491764	-		novel	NM_0042	TRIP11	exonic	622
chr2:6321	5.128507	8.615206	1.708339	0.036573	0.330455	up	chr2	63217850	63223901	+	hsa_circ_0054918	circBase	NM_0152	EHBP1	exonic	495
chr3:5666	5.116068	8.608657	1.707644	0.036608	0.330455	up	chr3	56661064	56662642	-	hsa_circ_0001314	circBase	NM_0152	FAM208A	exonic	392
chr17:353	5.106286	10.5475	1.622417	0.041126	0.333189	up	chr17	35310185	35311207	+	hsa_circ_0004751	circBase	NM_0121	AATF	exonic	549
chr5:7780	5.093995	8.597076	1.7065	0.036665	0.330455	up	chr5	77805606	77806221	-	hsa_circ_0004539	circBase	NM_0057	LHFPL2	exonic	615
chr4:1487	5.093995	8.597076	1.7065	0.036665	0.330455	up	chr4	1.49E+08	1.49E+08	+	hsa_circ_0125474	circBase	NM_0246	ARHGAP	exonic	555
chr6:7798	5.093995	8.597076	1.7065	0.036665	0.330455	up	chr6	77981056	77983024	-	hsa_circ_0132345	circBase	T306892	G071718	exonic	435
chr7:3338	5.093995	8.597076	1.7065	0.036665	0.330455	up	chr7	33388679	33427756	+	hsa_circ_0079812	circBase	NM_0144	BBS9	exonic	666
chr2:1116	5.093995	8.597076	1.7065	0.036665	0.330455	up	chr2	1.12E+08	1.12E+08	+		novel	NM_0011	ACOXL	exonic	493
chr3:1013	5.067183	10.5132	1.586697	0.043197	0.33431	up	chr3	1.01E+08	1.01E+08	-	hsa_circ_0121367	circBase	NM_0144	ZBTB11	exonic	1490
chr13:505	5.04082	8.568189	1.702033	0.036888	0.330455	up	chr13	50564995	50618904	-		novel	NR_00261	DLEU2	sense overl	53909
chr13:733	5.04082	8.568189	1.702033	0.036888	0.330455	up	chr13	73357560	73428293	+	hsa_circ_0100821	circBase	NM_0063	PIBF1	exonic	1369
chr12:932	5.04082	8.568189	1.702033	0.036888	0.330455	up	chr12	93202792	93205210	-	hsa_circ_0099483	circBase	NM_0035	EEA1	exonic	296
chr11:134	5.04082	8.568189	1.702033	0.036888	0.330455	up	chr11	1.34E+08	1.34E+08	+		novel	NM_1383	GLB1L2	sense overl	63010
chr4:5734	5.04082	8.568189	1.702033	0.036888	0.330455	up	chr4	57349304	57360228	+		novel	NM_0069	SRP72	sense overl	10924
chr22:176	5.04082	8.568189	1.702033	0.036888	0.330455	up	chr22	17625913	17630635	-	hsa_circ_0062157	circBase	NM_0178	CECR5	exonic	411
chr10:506	5.040745	8.569212	1.703214	0.036829	0.330455	up	chr10	50682072	50701298	-		novel	NM_0001	ERCC6	exonic	913
chr6:7335	5.040745	8.569212	1.703214	0.036829	0.330455	up	chr6	73357659	73358632	+	hsa_circ_0132255	circBase	ENST000	KCNQ5	intronic	973
chr1:2437	5.009363	8.552489	1.700377	0.036971	0.330455	up	chr1	2.44E+08	2.44E+08	-	hsa_circ_0000199	circBase	NM_0054	AKT3	exonic	555
chr15:328	5.009363	8.552489	1.700377	0.036971	0.330455	up	chr15	32814648	32825569	-		novel	NR_0366	WHAMM	sense overl	10921
chr2:3260	5.009363	8.552489	1.700377	0.036971	0.330455	up	chr2	32608461	32608900	+		novel	NM_0162	BIRC6	intronic	439
chr2:6171	5.009363	8.552489	1.700377	0.036971	0.330455	up	chr2	61719169	61724142	-	hsa_circ_0054877	circBase	NM_0034	XPO1	exonic	1128
chr2:3750	5.009363	8.552489	1.700377	0.036971	0.330455	up	chr2	37505008	37544322	-		novel	ENST000	PRKD3	exonic	1951
chrX:1376	5.009363	8.552489	1.700377	0.036971	0.330455	up	chrX	13762533	13771560	+	hsa_circ_0139822	circBase	NM_0036	OFD1	exonic	717
chr3:3345	5.004107	8.550498	1.70091	0.036945	0.330455	up	chr3	33450727	33467233	-	hsa_circ_0001985	circBase	NM_0145	UBP1	exonic	706
chr1:2451	5.004107	8.550498	1.70091	0.036945	0.330455	up	chr1	2.45E+08	2.45E+08	+	hsa_circ_0008294	circBase	NM_0012	EFCAB2	exonic	197
chr12:123	5.004107	8.550498	1.70091	0.036945	0.330455	up	chr12	1.23E+08	1.23E+08	+		novel	NM_0147	KNTC1	sense overl	8633
chr14:310	4.908246	8.502331	1.694416	0.037272	0.330455	up	chr14	31058588	31071337	+		novel	NM_0177	G2E3	exonic	875
chr10:701	4.907155	8.500965	1.692592	0.037365	0.330455	up	chr10	70152894	70161494	-	hsa_circ_0093951	circBase	NM_0179	RUFY2	exonic	472
chr5:6856	4.907155	8.500965	1.692592	0.037365	0.330455	up	chr5	68565033	68574175	+		novel	NM_0017	CDK7	sense overl	9142
chr14:661	4.907155	8.500965	1.692592	0.037365	0.330455	up	chr14	66188492	66200099	+	hsa_circ_0032249	circBase	NM_0044	FUT8	exonic	575
chr12:315	4.907155	8.500965	1.692592	0.037365	0.330455	up	chr12	31545200	31545630	-	hsa_circ_0004487	circBase	NM_1449	DENND5	exonic	210
chr14:529	4.907155	8.500965	1.692592	0.037365	0.330455	up	chr14	52920855	52937398	-		novel	ENST000	TXNDC1	intronic	16543
chr11:108	4.907155	8.500965	1.692592	0.037365	0.330455	up	chr11	1.08E+08	1.08E+08	+	hsa_circ_0003641	circBase	NM_0000	ATM	exonic	526
chrX:1286	4.887797	8.491764	1.691803	0.037405	0.330455	up	chrX	1.29E+08	1.29E+08	-	hsa_circ_0139754	circBase	NM_0030	SMARCA	exonic	2181
chr8:4221	4.887797	8.491764	1.691803	0.037405	0.330455	up	chr8	42213033	42218883	+	hsa_circ_0136562	circBase	NM_0026	POLB	exonic	251
chr9:1733	4.805559	8.452462	1.68667	0.037667	0.330455	up	chr9	17330629	17342442	+	hsa_circ_0086422	circBase	NM_0177	CNTLN	exonic	545
chr21:350	4.805559	8.452462	1.68667	0.037667	0.330455	up	chr21	35091101	35094956	+	hsa_circ_0004248	circBase	NM_0030	ITSN1	exonic	217

chr10:323	4.805559	8.452462	1.68667	0.037667	0.330455	up	chr10	32310177	32311917	-		novel	NM_0045	KIF5B	sense overl	1740
chr1:2492	4.805559	8.452462	1.68667	0.037667	0.330455	up	chr1	2.49E+08	2.49E+08	+		novel	NM_1707	PGBD2	sense overl	12913
chr1:2316	4.761235	8.431302	1.685077	0.037749	0.330455	up	chr1	2.32E+08	2.32E+08	+	hsa_circ_0004834	circBase	NM_0059	TSNAX	exonic	246
chr15:6752	4.759828	8.430413	1.683771	0.037816	0.330455	up	chr15	67524151	67529158	-	hsa_circ_0000620	circBase	NM_0246	AAGAB	exonic	462
chr15:8564	4.759828	8.430413	1.683771	0.037816	0.330455	up	chr15	85641178	85661070	+	hsa_circ_0036624	circBase	NM_0026	PDE8A	exonic	882
chr15:4492	4.759828	8.430413	1.683771	0.037816	0.330455	up	chr15	44925702	44941209	-	hsa_circ_0103660	circBase	NM_0251	SPG11	exonic	279
chr7:1048	4.755037	8.428326	1.684501	0.037779	0.330455	up	chr7	1.05E+08	1.05E+08	+	hsa_circ_0081833	circBase	NM_1826	SRPK2	exonic	392
chr7:3269	4.755037	8.428326	1.684501	0.037779	0.330455	up	chr7	32695598	32758805	-		novel	ENST000	DPY19L1	exonic	561
chr12:108	4.726665	8.415174	1.683928	0.037808	0.330455	up	chr12	1.08E+08	1.08E+08	+	hsa_circ_0028042	circBase	NM_0070	PWP1	exonic	613
chr6:1448	4.695	8.400764	1.684483	0.03778	0.330455	up	chr6	1.45E+08	1.45E+08	+		novel	NM_0071	UTRN	exonic	1383
chr9:1015	4.695	8.400764	1.684483	0.03778	0.330455	up	chr9	1.02E+08	1.02E+08	-		novel	NM_1735	ANKS6	exonic	369
chrM:862	4.695	8.400764	1.684483	0.03778	0.330455	up	chrM	8625	8776	+		novel	uc011mfi.	OK/SW-cl	sense overl	151
chr22:413	4.695	8.400764	1.684483	0.03778	0.330455	up	chr22	41326381	41326821	+		novel	ENST000	XPNPEP3	intronic	440
chr3:1077	4.608816	8.361916	1.679271	0.038049	0.330455	up	chr3	1.08E+08	1.08E+08	-	hsa_circ_0066739	circBase	NM_0017	CD47	exonic	477
chr2:1591	4.608816	8.361916	1.679271	0.038049	0.330455	up	chr2	1.59E+08	1.59E+08	-	hsa_circ_0056768	circBase	NM_1388	CCDC148	exonic	963
chr1:2251	4.59577	8.356182	1.677326	0.03815	0.330455	up	chr1	2.25E+08	2.25E+08	+	hsa_circ_0016599	circBase	NM_0013	DNAH14	exonic	800
chr3:4126	4.59577	8.356182	1.677326	0.03815	0.330455	up	chr3	41265511	41280845	+	hsa_circ_0004030	circBase	NM_0010	CTNNB1	exonic	2406
chr4:3987	4.59577	8.356182	1.677326	0.03815	0.330455	up	chr4	39875908	39878773	-	hsa_circ_0008312	circBase	NM_0011	PDS5A	exonic	285
chr2:6171	4.59577	8.356182	1.677326	0.03815	0.330455	up	chr2	61719169	61722748	-	hsa_circ_0054876	circBase	NM_0034	XPO1	exonic	999
chr13:100	4.59577	8.356182	1.677326	0.03815	0.330455	up	chr13	1.01E+08	1.01E+08	+	hsa_circ_0000500	circBase	NM_0002	PCCA	exonic	428
chr5:1398	4.59577	8.356182	1.677326	0.03815	0.330455	up	chr5	1.4E+08	1.4E+08	+	hsa_circ_0128128	circBase	NM_0177	ANKHD1	exonic	547
chr9:1399	4.59577	8.356182	1.677326	0.03815	0.330455	up	chr9	1.4E+08	1.4E+08	+		novel	ENST000	MAN1B1	intronic	868
chr12:194	4.575195	8.347094	1.679078	0.038059	0.330455	up	chr12	19406873	19418784	+	hsa_circ_0025544	circBase	NM_0190	PLEKHA5	exonic	484
chr10:726	4.575195	8.347094	1.679078	0.038059	0.330455	up	chr10	72604229	72619256	+	hsa_circ_0004977	circBase	NM_0039	SGPL1	exonic	588
chr7:2365	4.575195	8.347094	1.679078	0.038059	0.330455	up	chr7	23650789	23651172	+	hsa_circ_0001684	circBase	NM_1387	CCDC126	exonic	383
chr2:3940	4.446096	8.29224	1.667267	0.038677	0.330455	up	chr2	39406327	39440613	-		novel	ENST000	CDKL4	exonic	637
chr13:2197	4.446096	8.29224	1.667267	0.038677	0.330455	up	chr13	21974511	21995303	-	hsa_circ_0100061	circBase	NM_1532	ZDHHC20	exonic	449
chr13:4659	4.446096	8.29224	1.667267	0.038677	0.330455	up	chr13	46594583	46619651	-	hsa_circ_0100610	circBase	ENST000	ZC3H13	exonic	457
chr3:1362	4.446096	8.29224	1.667267	0.038677	0.330455	up	chr3	1.36E+08	1.36E+08	-		novel	NM_0058	STAG1	exonic	554
chr7:1103	4.446096	8.29224	1.667267	0.038677	0.330455	up	chr7	11030329	11062606	+		novel	NM_0146	PHF14	exonic	417
chr1:1152	4.444516	8.291293	1.669561	0.038556	0.330455	up	chr1	1.15E+08	1.15E+08	-	hsa_circ_0013652	circBase	NM_0071	CSDE1	exonic	1461
chr17:530	4.444516	8.291293	1.669561	0.038556	0.330455	up	chr17	5308376	5320002	-	hsa_circ_0041702	circBase	NM_0025	NUP88	exonic	747
chr10:104	4.444516	8.291293	1.669561	0.038556	0.330455	up	chr10	1.04E+08	1.04E+08	+		novel	NM_0011	GBF1	intronic	17809
chr1:4098	4.410688	8.277863	1.662702	0.038919	0.330979	up	chr1	40980186	40981590	+	hsa_circ_0113347	circBase	NM_0227	EXO5	sense overl	1404
chr3:4798	4.359003	8.256412	1.66122	0.038998	0.330979	up	chr3	4798868	4800357	+		novel	ENST000	ITPR1	intronic	1489
chr12:570	4.338195	8.248042	1.659352	0.039098	0.330979	up	chr12	57059987	57064148	-	hsa_circ_0027089	circBase	NM_0066	PTGES3	exonic	153
chr18:542	4.300796	8.23318	1.65566	0.039295	0.330979	up	chr18	54283475	54293688	-	hsa_circ_0108671	circBase	NM_0047	TXNL1	exonic	464
chr17:549	4.300796	8.23318	1.65566	0.039295	0.330979	up	chr17	54921379	54925426	+	hsa_circ_0044709	circBase	NM_0036	DGKE	exonic	424
chr3:6745	4.300796	8.23318	1.65566	0.039295	0.330979	up	chr3	67451154	67459458	-	hsa_circ_0005688	circBase	NM_0038	SUCLG2	sense overl	8304
chr8:3322	4.300796	8.23318	1.65566	0.039295	0.330979	up	chr8	33229628	33247316	-		novel	NM_0326	FUT10	sense overl	17688
chr1:2007	4.262665	8.218952	1.648139	0.039702	0.330979	up	chr1	2.01E+08	2.01E+08	+	hsa_circ_0006265	circBase	NM_2034	CAMSAP	exonic	528
chr4:1467	4.262665	8.218952	1.648139	0.039702	0.330979	up	chr4	1.47E+08	1.47E+08	-	hsa_circ_0001448	circBase	NM_1788	ZNF827	exonic	636
chr17:264	4.262665	8.218952	1.648139	0.039702	0.330979	up	chr17	26490568	26499644	+	hsa_circ_0003638	circBase	NM_0162	NLK	exonic	398
chr1:4547	4.205693	8.196763	1.643088	0.039977	0.330979	up	chr1	45471465	45475355	-		novel	NM_0246	HECTD3	exonic	1176
chr8:8264	4.198343	8.194974	1.637776	0.040269	0.330979	up	chr8	82646660	82647223	+		novel	NM_1522	CHMP4C	intronic	563
chr6:1069	4.198343	8.194974	1.637776	0.040269	0.330979	up	chr6	1.07E+08	1.07E+08	+		novel	NM_0016	AIM1	exonic	431
chr4:5272	4.198343	8.194974	1.637776	0.040269	0.330979	up	chr4	52726708	52765544	+	hsa_circ_0126575	circBase	NM_0151	DCUN1D	sense overl	38836
chr6:1253	4.198343	8.194974	1.637776	0.040269	0.330979	up	chr6	1.25E+08	1.25E+08	+	hsa_circ_0008536	circBase	NR_10444	RNF217	exonic	399
chr17:583	4.198343	8.194974	1.637776	0.040269	0.330979	up	chr17	58342772	58372162	-	hsa_circ_0044949	circBase	NM_0325	USP32	exonic	698
chr7:3064	4.198343	8.194974	1.637776	0.040269	0.330979	up	chr7	30649200	30662078	+		novel	NM_0020	GARS	exonic	878
chr6:9046	4.198343	8.194974	1.637776	0.040269	0.330979	up	chr6	90461149	90472249	-	hsa_circ_0003814	circBase	NM_0146	MDN1	exonic	1083
chr4:1741	4.174935	8.186215	1.634716	0.040438	0.330979	up	chr4	1.74E+08	1.74E+08	+	hsa_circ_0001458	circBase	NM_0174	GALNT7	exonic	461
chr4:4010	4.141138	8.172551	1.63486	0.04043	0.330979	up	chr4	40103694	40115128	+	hsa_circ_0069541	circBase	NM_0181	N4BP2	exonic	1435
chr14:766	4.141138	8.172551	1.63486	0.04043	0.330979	up	chr14	76638185	76647192	+	hsa_circ_0003819	circBase	NM_0179	GPATCH	exonic	466
chr11:745	4.141138	8.172551	1.63486	0.04043	0.330979	up	chr11	74500670	74528759	+	hsa_circ_0006705	circBase	NM_0010	RNF169	exonic	340
chr2:6547	4.103366	8.159319	1.627138	0.04086	0.332407	up	chr2	65473657	65492309	+	hsa_circ_0008529	circBase	NM_0057	ACTR2	exonic	855
chr9:1935	4.052472	8.141653	1.617302	0.041416	0.333399	up	chr9	19352070	19358158	+		novel	NM_0179	DENND4	exonic	665
chr19:462	4.052472	8.141653	1.617302	0.041416	0.333399	up	chr19	46205880	46232232	-		novel	NM_0010	FBXO46	sense overl	26352
chr12:116	4.008233	8.125354	1.613041	0.041659	0.333399	up	chr12	1.17E+08	1.17E+08	-	hsa_circ_0000443	circBase	NM_0153	MED13L	sense overl	7173
chr1:2228	3.961554	8.109171	1.605466	0.042095	0.333399	up	chr1	2.23E+08	2.23E+08	+	hsa_circ_0003413	circBase	NM_1446	BROX	exonic	179
chr2:4868	3.949304	8.106942	1.596567	0.042613	0.333399	up	chr2	46689856	46690102	+		novel	ENST000	PPP1R21	intronic	246
chr9:7086	3.949304	8.106942	1.596567	0.042613	0.333399	up	chr9	70863721	70875912	+	hsa_circ_0087133	circBase	ENST000	CBWD3	exonic	358
chr20:593	3.949304	8.106942	1.596567	0.042613	0.333399	up	chr20	5932656	5948233	+	hsa_circ_0059397	circBase	NM_0324	MCM8	exonic	1032
chr20:388	3.949304	8.106942	1.596567	0.042613	0.333399	up	chr20	3888572	3893281	+	hsa_circ_0006873	circBase	NM_0249	PANK2	exonic	784
chr10:323	3.949304	8.106942	1.596567	0.042613	0.333399	up	chr10	32306070	32311964	-		novel	NM_0045	KIF5B	exonic	1036
chr1:7826	3.949304	8.106942	1.596567	0.042613	0.333399	up	chr1	78267015	78332076	+	hsa_circ_0114204	circBase	NM_1985	FAM73A	exonic	1368
chr10:124	3.949304	8.106942	1.596567	0.042613	0.333399	up	chr10	1.24E+08	1.24E+08	+		novel	NM_0216	PLEKHA1	sense overl	37621
chr4:7870	3.861243	8.07646	1.584521	0.043327	0.33431	up	chr4	7870348	7873807	-	hsa_circ_0069152	circBase	NM_1985	AFAP1	exonic	227
chr5:1792	3.835956	8.069134	1.575896	0.043845	0.33431	up	chr5	1.79E+08	1.79E+08	+	hsa_circ_0075318	circBase	NM_0039	SQSTM1	exonic	549
chr5:1123	3.809637	8.059711	1.574797	0.043912	0.33431	up	chr5	1.12E+08	1.12E+08	+	hsa_circ_0001520	circBase	NM_1526	DCP2	exonic	889
chr3:5756	3.806353	8.059868	1.570234	0.044189	0.33431	up	chr3	57569624	57570191	-	hsa_circ_0005129	circBase	NM_0016	ARF4	exonic	191
chr3:1295	3.806353	8.059868	1.570234	0.044189	0.33431	up	chr3	1.3E+08	1.3E+08	-	hsa_circ_0121864	circBase	ENST000	TMCC1	intronic	254
chr3:1719	3.806353	8.059868	1.570234	0.044189	0.33431	up	chr3	1.72E+08	1.72E+08	+	hsa_circ_0001362	circBase	NM_0227	FNDC3B	exonic	585
chr8:1190	3.806353	8.059868	1.570234	0.044189	0.33431	up	chr8	1.19E+08	1.19E+08	-		novel	ENST000	EXT1	intronic	89028
chr12:125	3.789839	8.054793	1.566415	0.044423	0.33431	up	chr12	1.25E+08	1.25E+08	-		novel	NM_0210	UBC	sense overl	1824
chr3:1578	3.756337	8.042772	1.564324	0.044552	0.33431	up	chr3	1.58E+08	1.58E+08	+	hsa_circ_0001355	circBase	NM_0166	RSRC1	exonic	322
chr6:1674	3.756337	8.042772	1.564324	0.044552	0.33431	up	chr6	1.67E+08	1.67E+08	+	hsa_circ_0131304	circBase	NM_0070	FGFR1OP	sense overl	17931
chr1:6326	3.648084	8.013077	1.541052	0.04601	0.33431	up	chr1	63269389	63300523	+	hsa_circ_0113857	circBase	NM_0328	ATG4C	exonic	1157
chr5:3213	3.648084	8.013077	1.541052	0.04601	0.33431	up	chr5	32135677	32143986	-	hsa_circ_0001470	circBase	NM_0221	GOLPH3	exonic	247
chr13:998	3.648084	8.013077	1.541052	0.04601	0.33431	up	chr13	99890680	99896878	+	hsa_circ_0030720	circBase	NR_02664	UBAC2	exonic	358
chr11:185	3.648084	8.013077	1.541052	0.04601	0.33431	up	chr11	18566169	18591924	-	hsa_circ_0095537	circBase	NM_0183	UEVLD	exonic	867
chr6:3804	3.648084	8.013077	1.541052	0.04601	0.33431	up	chr6	38044909	38050233	+		novel	ENST000	ZFAND3	intronic	5324
chr6:5660	3.648084	8.013077	1.541052	0.04601	0.33431	up	chr6	56600025	56601379	-		novel	NR_0469	RNU6-71	intronic	1354
chr20:475	3.648084	8.013077	1.541052	0.04601	0.33431	up	chr20	47570092	47580435	+	hsa_circ_0003998	circBase	NM_0064	ARFGEF2	exonic	304
chr4:4009	3.648084	8.013077	1.541052	0.04601	0.33431	up	chr4	40098846	40108644	+	hsa_circ_0069538	circBase	NM_0181	N4BP2	exonic	1612
chr8:1418	3.61448	8.003163	1.532635	0.046551	0.335138	up	chr8	1.42E+08	1.42E+08	-	hsa_circ_0005982	circBase	NM_0056	PTK2	exonic	899
chr16:683	3.516905	7.973043	1.506818	0.048252	0.336436	up	chr16	68300495	68331379	+	hsa_circ_0006437	circBase	ENST000	SLC7A6	exonic	1716
chr8:4886	3.516905	7.973043	1.506818	0.048252	0.336436	up	chr8	48868433	48869991	-	hsa_circ_0136683	circBase	NM_0069	PRKDC	exonic	245
chr1:3250	3.516905	7.973043	1.506818	0.048252	0.336436	up	chr1	32502510	32505174	+	hsa_circ_0002467	circBase	NM_0065	KHDRBS1	exonic	463
chr14:509	3.516905	7.973043	1.506818	0.048252	0.336436	up	chr14	50942845	50971575	-		novel	NM_0065	MAP4K5	exonic	361
chr9:3395	3.516905	7.973043	1.506818	0.048252	0.336436	up	chr9	33953282	33963789	-	hsa_circ_0001847	circBase	NM_0012	UBAP2	exonic	309
chr7:3306	3.509431	7.973034	1.505505	0.048341	0.336436	up	chr7	33060883	33063720	-	hsa_circ_0079800	circBase	NM_0164	NT5C3A	exonic	203
chr5:1428	3.509431	7.973034	1.505505	0.048341	0.336436	up	chr5	14286979	14316852	+	hsa_circ_0071894	circBase	NM_0071	TRIO	exonic	1384
chr2:3282	3.509431	7.973034	1.505505	0.048341	0.336436	up	chr2	32820100	32828150	+		novel	NM_0162	BIRC6	exonic	569
chr3:1724	3.509431	7.973034	1.505505	0.048341	0.336436	up	chr3	1.72E+08	1.73E+08	+		novel	NM_0180	ECT2	exonic	1152
chr11:773	3.509431	7.973034	1.505505	0.048341	0.336436	up	chr11	77328522	77328898	-		novel	ENST000	CLNS1A	intronic	376
chr1:5221	3.509431	7.973034	1.505505	0.048341	0.336436	up	chr1	52215816	52231653	+	hsa_circ_0008022	circBase	NM_0245	OSBPL9	exonic	446
chr7:1565	3.509431	7.973034	1.505505	0.048341	0.336436	up	chr7	1.57E+08	1.57E+08	-	hsa_circ_0083117	circBase	NM_0224	LMBR1	exonic	606
chr12:110	3.509431	7.973034	1.505505	0.048341	0.336436	up	chr12	1107489	1137738	+	hsa_circ_0005534	circBase	T071384	G016488	exonic	984
chr14:100	3.509431	7.973034	1.505505	0.048341	0.336436	up	chr14	1.01E+08	1.01E+08	+	hsa_circ_0101187	circBase	NM_0034	YY1	exonic	163
chr19:106	-8.5388	11.20926	4.795935	0.000816	0.330455	down	chr19	10689567	10692554	-		novel	NM_0054	AP1M2	exonic	621
chr21:347	-8.45709	11.13369	4.632497	0.000986	0.330455	down	chr21	34793786	34805178	+	hsa_circ_0002113	circBase	NM_0055	IFNGR2	exonic	673
chr12:121	-8.44381	11.11871	4.599095	0.001025	0.330455	down	chr12	1.21E+08	1.21E+08	-	hsa_circ_0003472	circBase	NM_1390	SPPL3	exonic	312
chr5:1274	-8.41775	11.09742	4.55753	0.001076	0.330455	down	chr5	1.27E+08	1.27E+08	+	hsa_circ_0073767	circBase	NM_0010	SLC12A2	exonic	1208
chr11:341	-8.3756	11.05827	4.48177	0.001176	0.330455	down	chr11	34110941	34113603	+	hsa_circ_0002280	circBase	NM_0058	CAPRIN1	exonic	474
chr8:9971	-8.3505	11.03272	4.431275	0.001247	0.330455	down	chr8	99718694	99719539	-	hsa_circ_0004592	circBase	NM_0062	STK3	exonic	333
chr10:428	-8.33567	11.02197	4.413951	0.001273	0.330455	down	chr10	428586	496304	-		novel	NM_0149	DIP2C	sense overl	67718
chr11:668	-8.29279	10.98269	4.344144	0.001382	0.330455	down	chr11	66829533	66834318	+		novel	NM_0013	RHOD	intronic	4785
chr17:531	-8.27956	10.96756	4.315636	0.001429	0.330455	down	chr17	53108528	53124515	+	hsa_circ_0107139	circBase	NM_1785	STXBP4	exonic	345
chr9:2081	-8.22752	10.92165	4.23386	0.001573	0.330455	down	chr9	20819794	20823114	+	hsa_circ_0003295	circBase	ENST000	FOCAD	exonic	465
chr1:5186	-8.19631	10.89491	4.183134	0.00167	0.330455	down	chr1	51868106	51874004	-	hsa_circ_0005567	circBase	NM_0019	EPS15	exonic	607
chrX:1020	-8.15806	10.8604	4.117794	0.001804	0.330455	down	chrX	1.02E+08	1.02E+08	+		novel	NR_0389	LINC0063	sense overl	50173
chr11:625	-8.15589	10.85788	4.112873	0.001814	0.330455	down	chr11	62549365	62550297	+	hsa_circ_0006855	circBase	NM_0064	TAF6L	exonic	429
chr9:1143	-8.13429	10.83684	4.073211	0.001901	0.330455	down	chr9	1.14E+08	1.14E+08	-	hsa_circ_0088030	circBase	NM_0122	PTGR1	exonic	551
chr20:458	-8.10833	10.81505	4.033649	0.001993	0.330455	down	chr20	45891031	45905539	-	hsa_circ_0007026	circBase	NM_0124	ZMYND8	exonic	623
chr10:430	-8.10833	10.81505	4.033649	0.001993	0.330455	down	chr10	43088827	43089247	-		novel	ENST000	ZNF33B	intronic	420
chr11:611	-8.09174	10.80004	4.006181	0.002058	0.330455	down	chr11	61133516	61135470	+	hsa_circ_0002058	circBase	NM_0164	TMEM13	exonic	248
chr2:6170	-8.07378	10.78348	3.97611	0.002133	0.330455	down	chr2	61709514	61717911	-	hsa_circ_0054853	circBase	NM_0034	XPO1	exonic	1085
chr15:594	-8.05798	10.76954	3.95108	0.002198	0.330455	down	chr15	59445788	59455502	-		novel	NM_0049	MYO1E	exonic	600

chr6:1614:	-8.04821	10.76039	3.934506	0.002241	0.330455	down	chr6	1.61E+08	1.61E+08	+	hsa_circ_0078617	circBase	NR_12042	MAP3K4	exonic	1555
chr14:1962	-8.03338	10.74771	3.911945	0.002302	0.330455	down	chr14	19652263	19656224	+		novel	NR_11052	DUXAP10	exonic	645
chr1:5275	-8.03338	10.74771	3.911945	0.002302	0.330455	down	chr1	52759124	52769597	+	hsa_circ_0012499	circBase	NM_0047	ZFYVE9	exonic	413
chr15:4198	-8.02592	10.74136	3.900672	0.002333	0.330455	down	chr15	41988272	41991357	+	hsa_circ_0000592	circBase	NM_0010	MGA	exonic	1124
chr2:2237	-8.00834	10.72554	3.872462	0.002413	0.330455	down	chr2	2.24E+08	2.24E+08	+	hsa_circ_0058443	circBase	NM_0044	ACSL3	exonic	584
chr7:1790	-8.00707	10.72403	3.869738	0.002421	0.330455	down	chr7	17908029	17946963	-		novel	NM_0151	SNX13	sense overl	38934
chr12:2845	-7.97486	10.69079	3.810998	0.002596	0.330455	down	chr12	28458581	28460682	+	hsa_circ_0007723	circBase	NM_0183	CCDC91	exonic	467
chr11:118	-7.97388	10.69501	3.819647	0.00257	0.330455	down	chr11	1.19E+08	1.19E+08	+	hsa_circ_0009166	circBase	NM_0217	VPS11	exonic	766
chr5:9509	-7.97256	10.69344	3.816833	0.002578	0.330455	down	chr5	95099211	95124562	+	hsa_circ_0003561	circBase	NM_0148	RHOBTB3	exonic	672
chr16:697	-7.96575	10.68255	3.796794	0.002641	0.330455	down	chr16	69718789	69728142	+		novel	NM_0065	NFAT5	exonic	2724
chr18:774	-7.96575	10.68255	3.796794	0.002641	0.330455	down	chr18	77455224	77464917	+	hsa_circ_0006209	circBase	NM_0047	CTDP1	exonic	458
chr6:5341	-7.95433	10.67711	3.788657	0.002667	0.330455	down	chr6	53415334	53415651	-		novel	ENST000	GCLC	intronic	317
chr18:566	-7.94336	10.66717	3.771508	0.002722	0.330455	down	chr18	56601664	56621031	+	hsa_circ_0003423	circBase	NM_0181	ZNF532	exonic	804
chr5:6528	-7.93586	10.66059	3.760232	0.002759	0.330455	down	chr5	65288537	65310553	+	hsa_circ_0005649	circBase	NM_0186	ERBB2IP	exonic	542
chrM:6547	-7.88234	10.6077	3.672372	0.003065	0.330455	down	chrM	6546	6983	+		novel	T370381	G087361	intronic	437
chr13:460	-7.88234	10.6077	3.672372	0.003065	0.330455	down	chr13	46090277	46099186	+	hsa_circ_0008937	circBase	NM_0314	COG3	exonic	421
chr20:495	-7.87753	10.60809	3.673929	0.003059	0.330455	down	chr20	49551566	49571822	-		novel	NM_0038	DPM1	sense overl	20256
chr7:8041	-7.86429	10.59195	3.647217	0.003159	0.330455	down	chr7	80418621	80440017	-	hsa_circ_0004365	circBase	NM_0063	SEMA3C	exonic	907
chr2:1868	-7.85803	10.59072	3.646063	0.003163	0.330455	down	chr2	1.87E+08	1.87E+08	-		novel	NR_11021	LINC0147	sense overl	52609
chr2:2253	-7.83826	10.57314	3.617932	0.003272	0.330455	down	chr2	2.25E+08	2.25E+08	-	hsa_circ_0119244	circBase	NM_0035	CUL3	sense overl	28910
chr1:9434	-7.82446	10.5562	3.590877	0.00338	0.330455	down	chr1	94341823	94343418	-	hsa_circ_0013218	circBase	NM_0145	DNTTIP2	exonic	1595
chr12:104	-7.81822	10.55534	3.590327	0.003382	0.330455	down	chr12	1.05E+08	1.05E+08	+		novel	ENST000	TXNRD1	intronic	9079
chr11:648	-7.81666	10.55344	3.587396	0.003394	0.330455	down	chr11	64846824	64847259	-		PMID:252	NM_0806	CDCA5	exonic	435
chr2:1560	-7.81434	10.54714	3.577306	0.003435	0.330455	down	chr2	15601324	15618413	-	hsa_circ_0008083	circBase	NM_0159	NBAS	exonic	1256
chr4:2659	-7.8081	10.54637	3.576885	0.003437	0.330455	down	chr4	2659529	2674099	+	hsa_circ_0006291	circBase	NM_0037	FAM193A	exonic	1020
chr3:1698	-7.80412	10.53797	3.563581	0.003493	0.330455	down	chr3	1.7E+08	1.7E+08	-	hsa_circ_0067896	circBase	NM_0249	PHC3	exonic	1681
chr5:1713	-7.80412	10.53797	3.563581	0.003493	0.330455	down	chr5	1.71E+08	1.71E+08	-	hsa_circ_0004098	circBase	NM_0336	FBXW11	exonic	289
chr2:1742	-7.80412	10.53797	3.563581	0.003493	0.330455	down	chr2	1.74E+08	1.74E+08	+		novel	NM_0319	CDCA7	exonic	1164
chr10:581	-7.80106	10.54117	3.569245	0.003469	0.330455	down	chr10	5815804	5842668	-	hsa_circ_0017586	circBase	NM_0014	GDI2	exonic	774
chr1:2437	-7.80106	10.54117	3.569245	0.003469	0.330455	down	chr1	2.44E+08	2.44E+08	-	hsa_circ_0112779	circBase	NM_0054	AKT3	exonic	252
chr10:582	-7.79551	10.53088	3.553069	0.003537	0.330455	down	chr10	5827104	5842668	-	hsa_circ_0005379	circBase	NM_0014	GDI2	exonic	674
chr6:5656	-7.76294	10.50864	3.52072	0.003678	0.330455	down	chr6	56564450	56569167	-	hsa_circ_0005891	circBase	NM_1833	DST	exonic	261
chr1:8555	-7.75634	10.50053	3.508354	0.003733	0.330455	down	chr1	8555122	8601377	-	hsa_circ_0009581	circBase	NM_0121	RERE	exonic	379
chr2:2141	-7.73226	10.47439	3.469219	0.003914	0.330455	down	chr2	2.14E+08	2.14E+08	+	hsa_circ_0119076	circBase	NM_0245	SPAG16	exonic	434
chr7:7365	-7.72144	10.46473	3.455237	0.003981	0.330455	down	chr7	73651691	73661093	-		novel	NM_1814	RFC2	exonic	508
chr1:2345	-7.72144	10.46473	3.455237	0.003981	0.330455	down	chr1	2.35E+08	2.35E+08	-		novel	NM_0056	TARBP1	exonic	3766
chr10:274	-7.72144	10.46473	3.455237	0.003981	0.330455	down	chr10	27431315	27434519	-	hsa_circ_0005633	circBase	NM_0142	YME1L1	exonic	262
chr1:2470	-7.72144	10.46473	3.455237	0.003981	0.330455	down	chr1	2.47E+08	2.47E+08	-		novel	NM_0154	AHCTF1	exonic	4570
chr2:3927	-7.71873	10.46918	3.462376	0.003946	0.330455	down	chr2	39278284	39321167	-		novel	NM_0056	SOS1	sense overl	42883
chr13:103	-7.67306	10.42849	3.401438	0.004248	0.330455	down	chr13	1.03E+08	1.03E+08	+		novel	NM_0176	BIVM	exonic	429
chr12:275	-7.67125	10.42624	3.397029	0.004271	0.330455	down	chr12	27521194	27530497	+	hsa_circ_0098257	circBase	NM_0201	ARNTL2	sense overl	9303
chr11:329	-7.66801	10.4179	3.380532	0.004357	0.330455	down	chr11	32948702	32979632	+	hsa_circ_0021572	circBase	NM_0010	QSER1	exonic	4760
chr2:2155	-7.65673	10.40785	3.361161	0.004461	0.330455	down	chr2	2.16E+08	2.16E+08	-	hsa_circ_0119087	circBase	NM_0004	BARD1	sense overl	68200
chr6:1499	-7.65673	10.40785	3.361161	0.004461	0.330455	down	chr6	1.5E+08	1.5E+08	-		novel	NM_0070	KATNA1	exonic	742
chr10:127	-7.64539	10.39779	3.341958	0.004566	0.330455	down	chr10	1.28E+08	1.28E+08	-	hsa_circ_0093060	circBase	uc001ljg.1	DHX32	exonic	1598
chr1:2333	-7.63393	10.3876	3.322516	0.004675	0.330455	down	chr1	2.33E+08	2.33E+08	+	hsa_circ_0000198	circBase	NM_0148	PCNXL2	exonic	844
chr3:1139	-7.63393	10.3876	3.322516	0.004675	0.330455	down	chr3	11399891	11468400	+	hsa_circ_0008210	circBase	NM_0063	ATG7	exonic	795
chr1:2201	-7.63393	10.3876	3.322516	0.004675	0.330455	down	chr1	2.2E+08	2.2E+08	-		novel	ENST000	EPRS	intronic	1716
chr1:1932	-7.63393	10.3876	3.322516	0.004675	0.330455	down	chr1	1.93E+08	1.93E+08	+	hsa_circ_0003820	circBase	NM_0245	CDC73	exonic	263
chr3:1505	-7.63393	10.3876	3.322516	0.004675	0.330455	down	chr3	15057658	15071959	+	hsa_circ_0064454	circBase	NM_0032	NR2C2	exonic	837
chr16:249	-7.63393	10.3876	3.322516	0.004675	0.330455	down	chr16	249060	270746	-	hsa_circ_0006663	circBase	NM_0180	LUC7L	exonic	531
chr10:120	-7.60838	10.37344	3.295815	0.00483	0.330455	down	chr10	12021055	12056183	-	hsa_circ_0017713	circBase	NM_0155	UPF2	exonic	808
chr6:1496	-7.60646	10.37102	3.291186	0.004857	0.330455	down	chr6	1.5E+08	1.5E+08	+	hsa_circ_0078201	circBase	NM_0150	TAB2	exonic	1501
chr9:1935	-7.56525	10.32759	3.208751	0.005371	0.330455	down	chr9	19350699	19371818	+		novel	NM_0179	DENND4C	exonic	1423
chr7:4743	-7.54095	10.30614	3.166612	0.005655	0.330455	down	chr7	47436396	47467974	-		novel	NM_0227	TNS3	exonic	700
chr20:625	-7.54095	10.30614	3.166612	0.005655	0.330455	down	chr20	62559687	62562375	+	hsa_circ_0007609	circBase	NM_0252	DNAJC5	exonic	504
chr20:135	-7.54095	10.30614	3.166612	0.005655	0.330455	down	chr20	13550153	13610799	-		novel	NM_0177	TASP1	exonic	642
chr12:253	-7.54095	10.30614	3.166612	0.005655	0.330455	down	chr12	25378547	25398329	-		novel	NM_0049	KRAS	exonic	461
chr22:388	-7.54095	10.30614	3.166612	0.005655	0.330455	down	chr22	38890025	38897285	-	hsa_circ_0116554	circBase	NM_0063	DDX17	exonic	1038
chr1:3341	-7.54095	10.30614	3.166612	0.005655	0.330455	down	chr1	33413822	33415375	-	hsa_circ_0000048	circBase	NM_1533	RNF19B	exonic	351
chr2:2320	-7.54095	10.30614	3.166612	0.005655	0.330455	down	chr2	2.32E+08	2.32E+08	+	hsa_circ_0005563	circBase	ENST000	ARMC9	exonic	545
chr4:5401	-7.54095	10.30614	3.166612	0.005655	0.330455	down	chr4	54011499	54023686	-	hsa_circ_0002527	circBase	NM_1525	SCFD2	sense overl	12187
chr10:126	-7.54095	10.30614	3.166612	0.005655	0.330455	down	chr10	1.26E+08	1.26E+08	-	hsa_circ_0003570	circBase	NM_0146	FAM53B	exonic	828
chr15:752	-7.53669	10.31131	3.176624	0.005586	0.330455	down	chr15	75215988	75219228	-	hsa_circ_0007840	circBase	NM_0042	COX5A	exonic	245
chr13:801	-7.5099	10.28727	3.129299	0.005919	0.330455	down	chr13	80144697	80164341	+	hsa_circ_0100954	circBase	NR_12577	LINC0106	sense overl	19644
chr11:702	-7.5099	10.28727	3.129299	0.005919	0.330455	down	chr11	70265851	70266616	+	hsa_circ_0096355	circBase	NM_0052	CTTN	exonic	222
chr12:510	-7.50782	10.28455	3.123983	0.005958	0.330455	down	chr12	51079615	51090958	+	hsa_circ_0098735	circBase	NM_1736	DIP2B	exonic	731
chr4:1066	-7.50782	10.28455	3.123983	0.005958	0.330455	down	chr4	1.07E+08	1.07E+08	-	hsa_circ_0070573	circBase	NM_0203	INTS12	exonic	348
chr8:1240	-7.48683	10.26832	3.092097	0.006196	0.330455	down	chr8	1.24E+08	1.24E+08	+	hsa_circ_0085438	circBase	NM_1456	TBC1D31	exonic	442
chr6:1010	-7.48471	10.26554	3.086684	0.006237	0.330455	down	chr6	1.01E+08	1.01E+08	-		novel	NM_0068	ASCC3	exonic	765
chr8:9953	-7.45907	10.24346	3.043625	0.006577	0.330455	down	chr8	99538969	99541880	-	hsa_circ_0007545	circBase	NM_0062	STK3	sense overl	2911
chr18:544	-7.45471	10.23134	3.021484	0.006758	0.330455	down	chr18	54483261	54547396	+	hsa_circ_0108684	circBase	NM_0152	WDR7	exonic	336
chr2:4443	-7.45471	10.23134	3.021484	0.006758	0.330455	down	chr2	44436348	44445676	+	hsa_circ_0009062	circBase	NM_0027	PPM1B	exonic	288
chr5:7470	-7.44163	10.21985	3.000082	0.006939	0.330455	down	chr5	74706835	74712858	-	hsa_circ_0129633	circBase	NM_0057	COL4A3B	exonic	251
chr15:563	-7.44163	10.21985	3.000082	0.006939	0.330455	down	chr15	56347039	56349835	+		novel	T117097	G027613	intronic	2796
chr8:1308	-7.44163	10.21985	3.000082	0.006939	0.330455	down	chr8	1.31E+08	1.31E+08	-		novel	NM_0166	FAM49B	exonic	640
chr7:1102	-7.44163	10.21985	3.000082	0.006939	0.330455	down	chr7	11021998	11030474	+	hsa_circ_0133015	circBase	NM_0146	PHF14	exonic	933
chr1:2202	-7.43297	10.22103	3.001654	0.006926	0.330455	down	chr1	2.2E+08	2.2E+08	+	hsa_circ_0112021	circBase	NM_0180	IARS2	exonic	400
chr9:1150	-7.43297	10.22103	3.001654	0.006926	0.330455	down	chr9	1.15E+08	1.15E+08	-	hsa_circ_0088074	circBase	NM_0051	PTBP3	exonic	598
chr3:1116	-7.40865	10.2013	2.964754	0.007249	0.330455	down	chr3	1.12E+08	1.12E+08	+	hsa_circ_0005488	circBase	NM_1457	PHLDB2	exonic	1023
chr2:4371	-7.40638	10.19824	2.959159	0.007299	0.330455	down	chr2	43712372	43755127	-	hsa_circ_0054313	circBase	NM_0220	THADA	exonic	572
chr20:356	-7.40638	10.19824	2.959159	0.007299	0.330455	down	chr20	35684548	35693876	-	hsa_circ_0001153	circBase	NM_0028	RBL1	exonic	517
chr3:1074	-7.40411	10.19517	2.953537	0.00735	0.330455	down	chr3	1.07E+08	1.07E+08	+	hsa_circ_0001324	circBase	NM_0202	BBX	exonic	414
chr18:130	-7.38162	10.17823	2.921859	0.007644	0.330455	down	chr18	13029661	13042333	+		novel	NM_0321	CEP192	exonic	1017
chr20:344	-7.37696	10.17191	2.914732	0.007712	0.330455	down	chr20	34430494	34459751	+	hsa_circ_0060150	circBase	NM_0164	PHF20	exonic	1199
chr9:3329	-7.36792	10.1666	2.908535	0.007771	0.330455	down	chr9	33293677	33295425	+	hsa_circ_0001844	circBase	NM_0025	NFX1	sense overl	1748
chr18:768	-7.35644	10.15801	2.900099	0.007853	0.330455	down	chr18	76870354	76886375	+	hsa_circ_0108901	circBase	NM_1985	ATP9B	exonic	374
chr3:1700	-7.35405	10.15478	2.897441	0.007879	0.330455	down	chr3	1.7E+08	1.7E+08	+	hsa_circ_0122688	circBase	NM_0054	SKIL	sense overl	21021
chr12:123	-7.35405	10.15478	2.897441	0.007879	0.330455	down	chr12	1.23E+08	1.23E+08	-		novel	uc001ucw.	HCAR1	intronic	13459
chr16:159	-7.34885	10.14039	2.886077	0.007991	0.330455	down	chr16	15973660	15978062	-	hsa_circ_0003349	circBase	NM_1446	FOPNL	exonic	283
chr2:1759	-7.34885	10.14039	2.886077	0.007991	0.330455	down	chr2	1.76E+08	1.76E+08	-	hsa_circ_0057153	circBase	NM_0018	ATF2	exonic	524
chr18:296	-7.34885	10.14039	2.886077	0.007991	0.330455	down	chr18	29691716	29693823	+	hsa_circ_0005729	circBase	NM_0162	RNF138	exonic	282
chr3:1458	-7.34008	10.14296	2.887273	0.007979	0.330455	down	chr3	1.46E+08	1.46E+08	-	hsa_circ_0067682	circBase	NM_0009	PLOD2	exonic	668
chr11:685	-7.33765	10.13965	2.884554	0.008006	0.330455	down	chr11	68560782	68562379	-	hsa_circ_0023267	circBase	NM_0018	CPT1A	exonic	196
chr3:5662	-7.33468	10.12813	2.875566	0.008096	0.330455	down	chr3	56626997	56628056	+	hsa_circ_0001313	circBase	NR_0244	CCDC66	exonic	468
chr8:3762	-7.33468	10.12813	2.875566	0.008096	0.330455	down	chr8	37623043	37623873	+	hsa_circ_0001788	circBase	NM_0071	PROSC	exonic	220
chr2:1606	-7.33468	10.12813	2.875566	0.008096	0.330455	down	chr2	1.61E+08	1.61E+08	+	hsa_circ_0003149	circBase	NM_0228	MARCH7	exonic	1269
chr21:405	-7.33468	10.12813	2.875566	0.008096	0.330455	down	chr21	40590077	40590586	-	hsa_circ_0061721	circBase	NM_0189	BRWD1	exonic	277
chr16:709	-7.33468	10.12813	2.875566	0.008096	0.330455	down	chr16	70929883	71022415	-		novel	NM_0012	HYDIN	exonic	5284
chr7:4746	-7.33468	10.12813	2.875566	0.008096	0.330455	down	chr7	47464703	47465665	-		novel	ENST000	TNS3	intronic	962
chr7:1387	-7.33468	10.12813	2.875566	0.008096	0.330455	down	chr7	1.39E+08	1.39E+08	-	hsa_circ_0082622	circBase	NM_0201	ZC3HAV1	exonic	1752
chr6:1093	-7.33468	10.12813	2.875566	0.008096	0.330455	down	chr6	10931672	10956475	+		novel	NM_0010	SYCP2L	exonic	530
chr8:1872	-7.33468	10.12813	2.875566	0.008096	0.330455	down	chr8	18725183	18793652	-		novel	NM_0153	PSD3	exonic	1613
chr16:680	-7.33468	10.12813	2.875566	0.008096	0.330455	down	chr16	68059317	68083453	+	hsa_circ_0039909	circBase	NM_0178	DUS2	exonic	270
chr12:211	-7.33468	10.12813	2.875566	0.008096	0.330455	down	chr12	21196267	21207570	+	hsa_circ_0025582	circBase	NM_0010	SLCO1B7	exonic	955
chr10:104	-7.33468	10.12813	2.875566	0.008096	0.330455	down	chr10	1.04E+08	1.04E+08	+	hsa_circ_0092479	circBase	NM_0041	GBF1	exonic	420
chr17:126	-7.32687	10.14064	2.884377	0.008008	0.330455	down	chr17	1264385	1265302	-	hsa_circ_0007643	circBase	NM_0067	YWHAE	exonic	314
chr2:3662	-7.32593	10.13094	2.876924	0.008082	0.330455	down	chr2	36623756	36691798	+	hsa_circ_0005579	circBase	NM_0164	CRIM1	exonic	660
chr16:179	-7.32593	10.13094	2.876924	0.008082	0.330455	down	chr16	1793335	1798721	+	hsa_circ_0037334	circBase	NM_0151	MAPK8IP	exonic	611
chr19:538	-7.32346	10.12757	2.874159	0.00811	0.330455	down	chr19	53885060	53885228	+		novel	uc010ydx.	ZNF765	intronic	168
chr16:894	-7.2947	10.10321	2.853134	0.008324	0.330455	down	chr16	89484691	89497734	-	hsa_circ_0000727	circBase	NM_0012	ANKRD11	exonic	156
chr11:857	-7.2947	10.10321	2.853134	0.008324	0.330455	down	chr11	85707868	85714494	-	hsa_circ_0023919	circBase	NM_0071	PICALM	exonic	451
chr12:707	-7.24944	10.05641	2.818225	0.008694	0.330455	down	chr12	70713077	70736087	+	hsa_circ_0027554	circBase	NM_0145	CNOT2	exonic	1119
chr10:116	-7.24938	10.05629	2.818152	0.008695	0.330455	down	chr10	1.17E+08	1.17E+08	+	hsa_circ_0020086	circBase	NM_0209	FAM160B	exonic	790
chr19:577	-7.23538	10.05316	2.815014	0.008729	0.330455	down	chr19	57776759	57776962	-		novel	T181957	G041860	intronic	203
chr8:9953	-7.23425	10.04339	2.809367	0.00879	0.330455	down	chr8	99538969	99560389	-	hsa_circ_0003593	circBase	NM_0062	STK3	exonic	369
chr9:2185	-7.23425	10.04339	2.809367	0.00879	0.330455	down	chr9	21859301	21862134	+		novel	uc031tde.1	MTAP	intronic	2833
chr5:3724	-7.21888	10.03025	2.800388	0.008889	0.330455	down	chr5	37243114	37247847	-	hsa_circ_0008755	circBase	NM_0230	C5orf42	exonic	724
chr3:1719	-7.21888	10.03025	2.800388	0.008889	0.330455	down	chr3	1.72E+08	1.72E+08	+	hsa_circ_0122726	circBase	NM_0227	FNDC3B	sense overl	14551

chr10:1587	-7.21888	10.03025	2.800388	0.008889	0.330455	down	chr10	15875628	15889942	-	hsa_circ_0006665	circBase	NM_0249	FAM188A	exonic	636
chr7:2466	-7.21888	10.03025	2.800388	0.008889	0.330455	down	chr7	24663281	24720139	+	hsa_circ_0133997	circBase	NM_0164	MPP6	sense overl	56858
chr8:9971	-7.21888	10.03025	2.800388	0.008889	0.330455	down	chr8	99718694	99761618	-	hsa_circ_0085045	circBase	NM_0062	STK3	exonic	448
chr2:2253	-7.21888	10.03025	2.800388	0.008889	0.330455	down	chr2	2.25E+08	2.25E+08	-	hsa_circ_0058480	circBase	NM_0035	CUL3	exonic	1464
chr15:658	-7.21888	10.03025	2.800388	0.008889	0.330455	down	chr15	65844013	65849241	+	hsa_circ_0035914	circBase	NM_0163	HACD3	exonic	282
chr10:697	-7.21888	10.03025	2.800388	0.008889	0.330455	down	chr10	69714351	69773943	-	hsa_circ_0018478	circBase	NM_0156	HERC4	exonic	1429
chr1:2369	-7.21888	10.03025	2.800388	0.008889	0.330455	down	chr1	2.37E+08	2.37E+08	+	hsa_circ_0017140	circBase	NM_0002	MTR	exonic	2371
chr10:618	-7.21888	10.03025	2.800388	0.008889	0.330455	down	chr10	61844359	61845011	-	hsa_circ_0093804	circBase	NM_0011	ANK3	exonic	326
chr1:2947	-7.20758	10.0313	2.799846	0.008895	0.330455	down	chr1	29474591	29475738	-	hsa_circ_0113012	circBase	NM_0056	SRSF4	sense overl	1147
chr22:249	-7.18638	10.01054	2.785879	0.009052	0.330455	down	chr22	24953720	24968029	+		novel	NM_0041	SNRPD3	sense overl	14309
chr3:2399	-7.16248	10.00438	2.779896	0.00912	0.330455	down	chr3	23996027	24009514	+	hsa_circ_0001276	circBase	NR_1105	NR1D2	exonic	1634
chr7:3267	-7.15888	9.980965	2.767125	0.009266	0.330455	down	chr7	32672154	32678977	-	hsa_circ_0006010	circBase	NR_0366	DPY19L1	exonic	270
chr6:5645	-7.14656	9.991338	2.771199	0.009219	0.330455	down	chr6	56456979	56475360	-		novel	NM_0155	DST	exonic	1498
chr12:129	-7.14452	9.978713	2.76442	0.009298	0.330455	down	chr12	1.29E+08	1.29E+08	-	hsa_circ_0006689	circBase	NM_1456	SLC15A4	exonic	712
chr10:698	-7.12631	9.953372	2.749279	0.009475	0.330455	down	chr10	69869430	69882097	+		novel	NR_0456	MYPN	intronic	12667
chr6:1461	-7.11619	11.60429	3.853878	0.002467	0.330455	down	chr6	1.46E+08	1.46E+08	-	hsa_circ_0130934	circBase	NM_1730	SHPRH	sense overl	30590
chr7:4754	-7.11191	9.951661	2.746799	0.009505	0.330455	down	chr7	47546504	47568745	-	hsa_circ_0080190	circBase	NM_0227	TNS3	exonic	150
chr6:4437	-7.11191	9.951661	2.746799	0.009505	0.330455	down	chr6	44371545	44376369	+	hsa_circ_0004346	circBase	NM_0012	CDC5L	exonic	553
chr7:1565	-7.10981	9.939503	2.740234	0.009583	0.330455	down	chr7	1.57E+08	1.57E+08	-	hsa_circ_0133778	circBase	NM_0224	LMBR1	exonic	659
chr18:105	-7.09305	9.925346	2.730969	0.009695	0.330455	down	chr18	10534462	10550251	+	hsa_circ_0004922	circBase	NM_0038	NAPG	sense overl	15789
chr14:103	-7.09305	9.925346	2.730969	0.009695	0.330455	down	chr14	1.03E+08	1.03E+08	+	hsa_circ_0101323	circBase	NM_0033	TRAF3	exonic	977
chr1:2008	-7.09305	9.925346	2.730969	0.009695	0.330455	down	chr1	2.01E+08	2.01E+08	+		novel	NM_2034	CAMSAP	sense overl	1444
chr2:4393	-7.09305	9.925346	2.730969	0.009695	0.330455	down	chr2	43939363	43968182	+	hsa_circ_0120097	circBase	NM_1720	PLEKHH2	exonic	920
chr17:585	-7.09305	9.925346	2.730969	0.009695	0.330455	down	chr17	58529240	58539422	-	hsa_circ_0107304	circBase	NM_0012	APPBP2	exonic	742
chr1:7809	-7.09305	9.925346	2.730969	0.009695	0.330455	down	chr1	78097534	78099090	-	hsa_circ_0012987	circBase	NM_0155	ZZZ3	exonic	1556
chr14:102	-7.09305	9.925346	2.730969	0.009695	0.330455	down	chr14	1.02E+08	1.02E+08	+		novel	NM_0013	DYNC1H1	exonic	7257
chr16:118	-7.09305	9.925346	2.730969	0.009695	0.330455	down	chr16	11857333	11859541	-	hsa_circ_0003769	circBase	NM_0141	ZC3H7A	exonic	480
chr19:110	-7.09305	9.925346	2.730969	0.009695	0.330455	down	chr19	11041194	11042549	+		novel	ENST000	C19orf52	intronic	1355
chr2:1103	-7.09305	9.925346	2.730969	0.009695	0.330455	down	chr2	1.1E+08	1.1E+08	-	hsa_circ_0008953	circBase	NM_1447	SEPT10	exonic	383
chr6:9054	-7.08177	9.928492	2.731333	0.00969	0.330455	down	chr6	90541982	90581107	+		novel	NM_0121	CASP8AP	sense overl	39125
chr10:861	-7.07854	9.924077	2.728657	0.009723	0.330455	down	chr10	86177526	86198463	+	hsa_circ_0003018	circBase	NM_0189	CCSER2	exonic	450
chr17:303	-7.07854	9.924077	2.728657	0.009723	0.330455	down	chr17	30302667	30315516	+		novel	NM_0153	SUZ12	sense overl	12849
chr12:122	-7.05105	9.90507	2.711607	0.009933	0.330455	down	chr12	1.22E+08	1.22E+08	+		novel	NM_0150	SETD1B	exonic	492
chr14:955	-7.04439	9.895939	2.703724	0.010031	0.330455	down	chr14	95595808	95599840	-		novel	NM_0306	DICER1	exonic	779
chr10:247	-7.04439	9.895939	2.703724	0.010031	0.330455	down	chr10	24783428	24802363	+	hsa_circ_0017974	circBase	NM_0195	KIAA1217	exonic	498
chr13:734	-7.04439	9.895939	2.703724	0.010031	0.330455	down	chr13	73467921	73506836	+		novel	NM_0063	PIBF1	sense overl	38915
chr17:119	-7.02999	9.896708	2.702634	0.010045	0.330455	down	chr17	11998891	12016677	+	hsa_circ_0042098	circBase	NM_0030	MAP2K4	exonic	420
chr18:129	-7.02202	11.59379	3.755199	0.002775	0.330455	down	chr18	12999419	13030607	+	hsa_circ_0107922	circBase	NM_0321	CEP192	exonic	1538
chr22:472	-7.01488	11.50919	3.660751	0.003108	0.330455	down	chr22	47287161	47308084	+	hsa_circ_0063853	circBase	NM_0143	TBC1D22	exonic	307
chr12:643	-7.01252	9.882646	2.689579	0.010211	0.330455	down	chr12	64377726	64390908	+		novel	NM_0207	SRGAP1	sense overl	13182
chr19:540	-6.99197	9.842676	2.655786	0.010654	0.330455	down	chr19	54025870	54059162	+		novel	NM_0185	ZNF331	exonic	199
chr1:5289	-6.97388	9.827677	2.641499	0.010847	0.330455	down	chr1	52891020	52937774	-		novel	NM_0152	ZCCHC11	exonic	2140
chr1:7625	-6.95546	9.812337	2.626551	0.011053	0.330455	down	chr1	76253181	76255041	+	hsa_circ_0012967	circBase	NM_0045	RABGGT	exonic	306
chr5:7080	-6.95546	9.812337	2.626551	0.011053	0.330455	down	chr5	70805757	70805922	+		novel	NM_0184	BDP1	sense overl	165
chr3:1556	-6.95546	9.812337	2.626551	0.011053	0.330455	down	chr3	1.56E+08	1.56E+08	+	hsa_circ_0122484	circBase	NM_0038	GMPS	exonic	859
chr2:2040	-6.95546	9.812337	2.626551	0.011053	0.330455	down	chr2	2.04E+08	2.04E+08	+		novel	NM_0011	NBEAL1	exonic	864
chr4:1487	-6.95546	9.812337	2.626551	0.011053	0.330455	down	chr4	1.49E+08	1.49E+08	+		novel	NM_0246	ARHGAP	exonic	905
chr7:6169	-6.95546	9.812337	2.626551	0.011053	0.330455	down	chr7	6169838	6180615	+		novel	NM_0321	USP42	sense overl	10777
chr1:1556	-6.95546	9.812337	2.626551	0.011053	0.330455	down	chr1	1.56E+08	1.56E+08	-		novel	NM_0182	YY1AP1	sense overl	3129
chr16:188	-6.95546	9.812337	2.626551	0.011053	0.330455	down	chr16	18846213	18856973	-	hsa_circ_0038258	circBase	NM_0150	SMG1	exonic	2334
chr11:129	-6.95546	9.812337	2.626551	0.011053	0.330455	down	chr11	1.3E+08	1.3E+08	+		novel	NM_0016	APLP2	exonic	1281
chr15:638	-6.95546	9.812337	2.626551	0.011053	0.330455	down	chr15	63821212	63855207	+	hsa_circ_0007349	circBase	NR_0463	USP3	exonic	823
chr11:107	-6.95546	9.812337	2.626551	0.011053	0.330455	down	chr11	1.08E+08	1.08E+08	-	hsa_circ_0094814	circBase	NM_0175	SLC35F2	exonic	829
chr6:7633	-6.95546	9.812337	2.626551	0.011053	0.330455	down	chr6	76331247	76344527	+	hsa_circ_0077078	circBase	NM_0155	SENP6	exonic	406
chr8:1311	-6.95546	9.812337	2.626551	0.011053	0.330455	down	chr8	1.31E+08	1.31E+08	-		novel	NM_0184	ASAP1	exonic	894
chr3:6915	-6.95546	9.812337	2.626551	0.011053	0.330455	down	chr3	69153649	69153962	+		novel	ENST000	ARL6IP5	intronic	313
chr19:474	-6.95546	9.812337	2.626551	0.011053	0.330455	down	chr19	47421744	47492932	+		novel	NM_0044	ARHGAP	sense overl	71188
chr16:197	-6.95546	9.812337	2.626551	0.011053	0.330455	down	chr16	19775169	19800244	+	hsa_circ_0038378	circBase	NM_1532	IQCK	exonic	216
chr13:100	-6.95062	9.828297	2.639056	0.01088	0.330455	down	chr13	1.01E+08	1.01E+08	+	hsa_circ_0099729	circBase	NM_0002	PCCA	exonic	792
chr19:898	-6.94786	9.823302	2.634594	0.010941	0.330455	down	chr19	8987044	8987334	-	hsa_circ_0049098	circBase	NM_0246	MUC16	exonic	193
chr4:1694	-6.93674	9.807963	2.620531	0.011137	0.330455	down	chr4	1.69E+08	1.69E+08	+		novel	NM_0160	PALLD	exonic	990
chr1:1509	-6.92161	9.809854	2.620144	0.011142	0.330455	down	chr1	1.51E+08	1.51E+08	+	hsa_circ_0006760	circBase	NM_0212	PRUNE	exonic	344
chr5:3698	-6.91327	11.49413	3.559078	0.003512	0.330455	down	chr5	36982266	36986403	+	hsa_circ_0128921	circBase	NM_0153	NIPBL	sense overl	4137
chr3:1249	-6.90284	9.782745	2.595274	0.011497	0.330455	down	chr3	1.25E+08	1.25E+08	-	hsa_circ_0067098	circBase	NM_0219	ZNF148	exonic	900
chr1:1556	-6.90284	9.782745	2.595274	0.011497	0.330455	down	chr1	1.56E+08	1.56E+08	-	hsa_circ_0003608	circBase	NM_0182	YY1AP1	exonic	390
chr6:8792	-6.89981	11.24534	3.288974	0.00487	0.330455	down	chr6	87920168	87928449	+	hsa_circ_0004383	circBase	ENST000	ZNF292	intronic	8281
chr6:1584	-6.89898	9.777361	2.590307	0.01157	0.330455	down	chr6	1.58E+08	1.59E+08	+		novel	NM_0038	SYNJ2	sense overl	16853
chr6:1701	-6.86391	9.739285	2.55259	0.012134	0.330455	down	chr6	1.7E+08	1.7E+08	-	hsa_circ_0078755	circBase	NM_0182	PHF10	exonic	1026
chr4:1704	-6.86019	9.746073	2.558412	0.012045	0.330455	down	chr4	1.7E+08	1.71E+08	-	hsa_circ_0125769	circBase	NM_0122	NEK1	exonic	806
chr1:2473	-6.84403	9.722954	2.534603	0.012414	0.330455	down	chr1	2.47E+08	2.47E+08	-	hsa_circ_0002922	circBase	NM_0012	ZNF124	sense overl	4248
chr4:1705	-6.83281	9.731456	2.540993	0.012314	0.330455	down	chr4	1.71E+08	1.71E+08	-	hsa_circ_0125772	circBase	NM_0122	NEK1	exonic	916
chr19:348	-6.82866	9.725725	2.535057	0.012407	0.330455	down	chr19	34881665	34882063	+		novel	ENST000	GPI	intronic	398
chr5:3735	-6.82496	9.733884	2.542193	0.012295	0.330455	down	chr5	37351291	37358253	-		novel	NM_1534	NUP155	exonic	331
chr10:111	-6.82485	9.733693	2.542	0.012298	0.330455	down	chr10	1118055	1126416	+	hsa_circ_0017438	circBase	NM_0140	WDR37	exonic	436
chr3:5784	-6.82397	9.706628	2.516547	0.012701	0.330455	down	chr3	57843459	57882659	+		novel	NM_0013	SLMAP	exonic	931
chr14:454	-6.82397	9.706628	2.516547	0.012701	0.330455	down	chr14	45464948	45523752	+	hsa_circ_0101839	circBase	NM_0150	FAM179B	exonic	2354
chr12:571	-6.82397	9.706628	2.516547	0.012701	0.330455	down	chr12	57133077	57139965	-		novel	NM_0009	PRIM1	exonic	610
chr11:118	-6.82397	9.706628	2.516547	0.012701	0.330455	down	chr11	1.18E+08	1.18E+08	-	hsa_circ_0024470	circBase	NM_0201	IFT46	exonic	373
chr22:412	-6.81953	11.32811	3.30796	0.004759	0.330455	down	chr22	41277773	41278181	+	hsa_circ_0008360	circBase	NM_0220	XPNPEP3	exonic	408
chr3:1116	-6.80817	9.696885	2.505203	0.012885	0.330455	down	chr3	1.12E+08	1.12E+08	+	hsa_circ_0001326	circBase	NM_1457	PHLDB2	exonic	666
chr12:104	-6.80352	9.689893	2.498006	0.013004	0.330455	down	chr12	1.05E+08	1.05E+08	+		novel	ENST000	CHST11	intronic	35244
chr1:3269	-6.80352	9.689893	2.498006	0.013004	0.330455	down	chr1	32691771	32692131	+	hsa_circ_0011385	circBase	NM_0037	EIF3I	exonic	278
chr20:356	-6.80352	9.689893	2.498006	0.013004	0.330455	down	chr20	35695126	35696589	-	hsa_circ_0060238	circBase	NM_0028	RBL1	exonic	556
chr4:1298	-6.80352	9.689893	2.498006	0.013004	0.330455	down	chr4	1.3E+08	1.3E+08	-		novel	NM_1446	SCLT1	exonic	390
chr4:1467	-6.80352	9.689893	2.498006	0.013004	0.330455	down	chr4	1.47E+08	1.47E+08	-	hsa_circ_0001447	circBase	NM_1788	ZNF827	exonic	402
chr11:634	-6.80352	9.689893	2.498006	0.013004	0.330455	down	chr11	63419407	63420047	-	hsa_circ_0022599	circBase	NM_0154	ATL3	exonic	156
chr2:1573	-6.80352	9.689893	2.498006	0.013004	0.330455	down	chr2	1.57E+08	1.57E+08	+		novel	NM_0004	GPD2	exonic	669
chr12:566	-6.80352	9.689893	2.498006	0.013004	0.330455	down	chr12	56679699	56692475	-		novel	ENST000	CS	exonic	242
chr7:7446	-6.80352	9.689893	2.498006	0.013004	0.330455	down	chr7	74466817	74471723	-	hsa_circ_0080641	circBase	ENST000	WBSCR1	exonic	348
chr2:7294	-6.80352	9.689893	2.498006	0.013004	0.330455	down	chr2	72945231	72958374	-	hsa_circ_0120939	circBase	NM_0151	EXOC6B	exonic	342
chr1:2285	-6.80352	9.689893	2.498006	0.013004	0.330455	down	chr1	2.29E+08	2.29E+08	+		novel	ENST000	OBSCN	intronic	377
chr17:601	-6.80352	9.689893	2.498006	0.013004	0.330455	down	chr17	60106901	60140662	-	hsa_circ_0107375	circBase	NM_0051	MED13	exonic	1217
chr13:966	-6.80352	9.689893	2.498006	0.013004	0.330455	down	chr13	96636057	96651561	-	hsa_circ_0030632	circBase	NM_0201	UGGT2	exonic	432
chr14:454	-6.80352	9.689893	2.498006	0.013004	0.330455	down	chr14	45473263	45497532	+	hsa_circ_0101843	circBase	NM_0150	FAM179B	exonic	1320
chr10:120	-6.80352	9.689893	2.498006	0.013004	0.330455	down	chr10	12039670	12056183	-	hsa_circ_0000213	circBase	NM_0155	UPF2	exonic	699
chr3:1933	-6.80352	9.689893	2.498006	0.013004	0.330455	down	chr3	1.93E+08	1.93E+08	+		novel	NM_0155	OPA1	exonic	1431
chr11:341	-6.80352	9.689893	2.498006	0.013004	0.330455	down	chr11	34111725	34113603	+	hsa_circ_0004851	circBase	NM_0058	CAPRIN1	exonic	412
chr14:377	-6.76355	9.686071	2.488746	0.013157	0.330455	down	chr14	37716307	37739730	+		novel	NM_1387	MIPOL1	exonic	691
chr6:8292	-6.75851	9.679021	2.481398	0.013281	0.330455	down	chr6	82920530	82922510	-	hsa_circ_0002041	circBase	NM_0155	IBTK	exonic	305
chr15:413	-6.74613	9.660495	2.467011	0.013526	0.330455	down	chr15	41365625	41372102	-		novel	NM_0175	INO80	exonic	468
chr10:273	-6.69818	9.620079	2.446481	0.013884	0.330455	down	chr10	27311486	27322306	-		PMID:250	NM_0149	ANKRD2	exonic	561
chr7:1566	-6.69335	9.613359	2.443478	0.013937	0.330455	down	chr7	1.57E+08	1.57E+08	-	hsa_circ_0005939	circBase	NM_0224	LMBR1	exonic	253
chr20:614	-6.67862	9.59264	2.434179	0.014104	0.330455	down	chr20	61488746	61490878	-	hsa_circ_0006458	circBase	NM_0013	TCFL5	exonic	404
chr4:1032	-6.67862	9.59264	2.434179	0.014104	0.330455	down	chr4	1.03E+08	1.03E+08	-	hsa_circ_0002782	circBase	NM_0221	SLC39A8	exonic	621
chr15:256	-6.67862	9.59264	2.434179	0.014104	0.330455	down	chr15	25650607	25657118	-	hsa_circ_0000586	circBase	NM_0004	UBE3A	exonic	291
chr1:5117	-6.65591	9.574736	2.424113	0.014286	0.330455	down	chr1	51171457	51210447	-	hsa_circ_0113517	circBase	NM_0070	FAF1	exonic	290
chr10:861	-6.65591	9.574736	2.424113	0.014286	0.330455	down	chr10	86191778	86237420	+	hsa_circ_0094298	circBase	NM_0189	CCSER2	sense overl	45642
chr15:994	-6.65591	9.574736	2.424113	0.014286	0.330455	down	chr15	99434553	99442850	+	hsa_circ_0037026	circBase	NM_0008	IGF1R	exonic	607
chr10:104	-6.65591	9.574736	2.424113	0.014286	0.330455	down	chr10	1.05E+08	1.05E+08	-	hsa_circ_0019779	circBase	NM_0122	NT5C2	exonic	398
chr15:657	-6.65537	9.602658	2.434423	0.014099	0.330455	down	chr15	65756098	65773953	-	hsa_circ_0104274	circBase	NM_0177	DPP8	exonic	954
chr2:2141	-6.63374	10.99832	2.87658	0.008085	0.330455	down	chr2	2.14E+08	2.14E+08	+	hsa_circ_0004664	circBase	NM_0245	SPAG16	exonic	553
chr11:134	-6.63275	9.585377	2.425102	0.014268	0.330455	down	chr11	1.34E+08	1.34E+08	-	hsa_circ_0024914	circBase	NM_0152	NCAPD3	exonic	836
chr5:1308	-6.63275	9.585377	2.425102	0.014268	0.330455	down	chr5	1.31E+08	1.31E+08	-	hsa_circ_0073835	circBase	NM_0163	RAPGEF6	exonic	298
chr11:130	-6.63275	9.585377	2.425102	0.014268	0.330455	down	chr11	1.31E+08	1.31E+08	-	hsa_circ_0024895	circBase	NM_0147	SNX19	exonic	408
chr8:5673	-6.63269	9.556333	2.413505	0.014481	0.330455	down	chr8	56730861	56737611	+		novel	NM_0248	TGS1	sense overl	6750
chr20:317	-6.63269	9.556333	2.413505	0.014481	0.330455	down	chr20	3175918	3180679	-		novel	NM_0239	DDRGK1	sense overl	4761
chr2:1734	-6.63269	9.556333	2.413505	0.014481	0.330455	down	chr2	1.73E+08	1.73E+08	+	hsa_circ_0118104	circBase	NM_0026	PDK1	exonic	573
chr3:1214	-6.63269	9.556333	2.413505	0.014481	0.330455	down	chr3	1.21E+08	1.21E+08	-	hsa_circ_0121617	circBase	NM_0044	GOLGB1	exonic	533
chr5:1586	-6.63269	9.556333	2.413505	0.014481	0.330455	down	chr5	1.59E+08	1.59E+08	+	hsa_circ_0128457	circBase	NM_1450	UBLCP1	exonic	236

chr2:1128	-6.63269	9.556333	2.413505	0.014481	0.330455	down	chr2	1.13E+08	1.13E+08	+		novel	NM_0328	TMEM87	exonic	1127
chr20:459	-6.63269	9.556333	2.413505	0.014481	0.330455	down	chr20	45910845	45976670	-		novel	NM_0124	ZMYND8	exonic	984
chr12:121	-6.63269	9.556333	2.413505	0.014481	0.330455	down	chr12	1.21E+08	1.21E+08	-	hsa_circ_0004479	circBase	NM_1390	SPPL3	exonic	401
chr1:9264	-6.63269	9.556333	2.413505	0.014481	0.330455	down	chr1	92643367	92648152	+	hsa_circ_0114470	circBase	NM_0152	KIAA110	exonic	2559
chr15:228	-6.63269	9.556333	2.413505	0.014481	0.330455	down	chr15	22866895	22870003	+		novel	NM_0529	TUBGCP	exonic	515
chr9:1118	-6.63269	9.556333	2.413505	0.014481	0.330455	down	chr9	1.12E+08	1.12E+08	-	hsa_circ_0087905	circBase	NM_0320	TMEM24	exonic	269
chr9:2204	-6.63269	9.556333	2.413505	0.014481	0.330455	down	chr9	22046749	22062025	+		novel	NR_00352	CDKN2B-	exonic	1179
chr15:639	-6.63269	9.556333	2.413505	0.014481	0.330455	down	chr15	63988322	64039282	-	hsa_circ_0104160	circBase	NM_0039	HERC1	exonic	2767
chr6:3694	-6.63269	9.556333	2.413505	0.014481	0.330455	down	chr6	36941836	36942675	-		novel	ENST000	MTCH1	intronic	839
chr1:1715	-6.63269	9.556333	2.413505	0.014481	0.330455	down	chr1	1.72E+08	1.72E+08	+	hsa_circ_0004412	circBase	NM_0151	PRRC2C	exonic	397
chr7:1024	-6.63269	9.556333	2.413505	0.014481	0.330455	down	chr7	1.02E+08	1.02E+08	-	hsa_circ_0132828	circBase	NM_1450	FBXL13	sense overl	41584
chr19:904	-6.63269	9.556333	2.413505	0.014481	0.330455	down	chr19	9048419	9048911	-		novel	NM_0246	MUC16	sense overl	492
chr8:5277	-6.61258	11.13851	2.967853	0.007221	0.330455	down	chr8	52773404	52773806	-	hsa_circ_0001801	circBase	NM_0529	PCMTD1	exonic	402
chr19:346	-6.60976	9.567884	2.414962	0.014454	0.330455	down	chr19	34687538	34712643	+	hsa_circ_0000926	circBase	NM_0155	LSM14A	exonic	1083
chr3:4708	-6.60976	9.567884	2.414962	0.014454	0.330455	down	chr3	47084050	47103836	-	hsa_circ_0065145	circBase	NM_0141	SETD2	exonic	1129
chr5:1703	-6.5864	9.550175	2.404626	0.014646	0.330455	down	chr5	1.7E+08	1.7E+08	+	hsa_circ_0128572	circBase	NM_0228	RANBP17	exonic	405
chr1:5425	-6.5864	9.550175	2.404626	0.014646	0.330455	down	chr1	54254805	54262817	-	hsa_circ_0006676	circBase	NM_0180	NDC1	exonic	477
chr2:1707	-6.5864	9.550175	2.404626	0.014646	0.330455	down	chr2	1.71E+08	1.71E+08	+	hsa_circ_0002603	circBase	NM_1720	UBR3	exonic	493
chr16:717	-6.57068	9.527735	2.393333	0.014859	0.330455	down	chr16	71712657	71715808	-	hsa_circ_0000715	circBase	NM_0150	PHLPP2	exonic	533
chrX:1436	-6.55934	9.512521	2.385513	0.015008	0.330455	down	chrX	1.44E+08	1.44E+08	-		novel	T381097	G090327	sense overl	15636
chr14:311	-6.5385	9.514086	2.383343	0.01505	0.330455	down	chr14	31139461	31144271	+	hsa_circ_0009140	circBase	NM_0161	SCFD1	exonic	387
chr15:812	-6.5385	9.514086	2.383343	0.01505	0.330455	down	chr15	81262802	81274523	-	hsa_circ_0005558	circBase	T120637	G028499	exonic	1143
chr4:1487	-6.53665	11.06871	2.855414	0.008301	0.330455	down	chr4	1.49E+08	1.49E+08	+	hsa_circ_0071106	circBase	NM_0246	ARHGAP	exonic	650
chr3:3178	-6.53184	9.505158	2.378698	0.01514	0.330455	down	chr3	3178943	3186394	+	hsa_circ_0123486	circBase	NM_1829	TRNT1	exonic	460
chr20:357	-6.51591	9.482799	2.365922	0.015389	0.330455	down	chr20	3577023	3605219	+		novel	NM_1393	ATRN	exonic	447
chr3:6160	-6.51591	9.482799	2.365922	0.015389	0.330455	down	chr3	61603333	61609229	+	hsa_circ_0007038	circBase	ENST000	PTPRG	intronic	5896
chr5:7407	-6.51394	9.495695	2.371812	0.015274	0.330455	down	chr5	74076187	74137504	-	hsa_circ_0129606	circBase	NM_0155	FAM169A	sense overl	61317
chr2:9981	-6.47007	10.84912	2.650356	0.010727	0.330455	down	chr2	99811213	99812219	+	hsa_circ_0008609	circBase	NR_0283	MRPL30	exonic	405
chr7:2468	-6.46538	9.429925	2.333561	0.016041	0.330455	down	chr7	24681334	24708279	+	hsa_circ_0079625	circBase	NM_0164	MPP6	exonic	997
chr8:4830	-6.46538	9.429925	2.333561	0.016041	0.330455	down	chr8	48308935	48353104	+	hsa_circ_0136657	circBase	NM_0010	SPIDR	exonic	572
chr19:457	-6.46538	9.429925	2.333561	0.016041	0.330455	down	chr19	45781180	45783992	+	hsa_circ_0004440	circBase	NM_0314	MARK4	exonic	490
chr11:734	-6.46353	9.458187	2.347124	0.015765	0.330455	down	chr11	73418464	73429935	-	hsa_circ_0004596	circBase	NM_0028	RAB6A	exonic	312
chr5:6528	-6.46353	9.458187	2.347124	0.015765	0.330455	down	chr5	65284462	65324171	+	hsa_circ_0129386	circBase	NM_0186	ERBB2IP	exonic	1263
chr4:8568	-6.46335	9.457895	2.346959	0.015768	0.330455	down	chr4	85686962	85687525	-	hsa_circ_0127161	circBase	NM_0149	WDFY3	exonic	339
chr2:7427	-6.43876	9.409496	2.319846	0.016327	0.330455	down	chr2	74273404	74307718	+		novel	NM_0012	TET3	exonic	2319
chr8:1240	-6.43876	9.409496	2.319846	0.016327	0.330455	down	chr8	1.24E+08	1.24E+08	+	hsa_circ_0085440	circBase	NM_1456	TBC1D31	exonic	1359
chr5:6487	-6.43876	9.409496	2.319846	0.016327	0.330455	down	chr5	64875250	64879046	+	hsa_circ_0072702	circBase	NM_0153	PPWD1	exonic	372
chr3:1214	-6.43876	9.409496	2.319846	0.016327	0.330455	down	chr3	1.21E+08	1.21E+08	-		novel	NM_0044	GOLGB1	exonic	7101
chr10:752	-6.43876	9.409496	2.319846	0.016327	0.330455	down	chr10	75296026	75335433	-		novel	NM_1525	USP54	exonic	1161
chr9:1344	-6.43876	9.409496	2.319846	0.016327	0.330455	down	chr9	1.34E+08	1.35E+08	-		novel	NM_0053	RAPGEF1	exonic	1844
chr20:343	-6.43876	9.409496	2.319846	0.016327	0.330455	down	chr20	34317233	34320057	-	hsa_circ_0008817	circBase	NM_0049	RBM39	exonic	315
chr4:4200	-6.43876	9.409496	2.319846	0.016327	0.330455	down	chr4	42003632	42041070	+	hsa_circ_0126390	circBase	NM_0063	SLC30A9	exonic	628
chr17:353	-6.43876	9.409496	2.319846	0.016327	0.330455	down	chr17	35343915	35348156	+	hsa_circ_0003532	circBase	NM_0121	AATF	exonic	566
chr6:8622	-6.43876	9.409496	2.319846	0.016327	0.330455	down	chr6	86227473	86253478	-		novel	NM_0204	SNX14	exonic	1133
chr10:698	-6.43876	9.409496	2.319846	0.016327	0.330455	down	chr10	69881194	69882097	+		PMID:250	NM_0325	MYPN	exonic	903
chr17:649	-6.43876	9.409496	2.319846	0.016327	0.330455	down	chr17	6493098	6538491	-		novel	NM_0148	KIAA075	exonic	2854
chr2:7158	-6.43876	9.409496	2.319846	0.016327	0.330455	down	chr2	71582848	71597135	+	hsa_circ_0120881	circBase	NM_0144	ZNF638	exonic	948
chr1:5897	-6.43764	9.439058	2.33442	0.016024	0.330455	down	chr1	58971731	59002413	-	hsa_circ_0000072	circBase	NM_1452	OMA1	exonic	865
chr1:2132	-6.43764	9.439058	2.33442	0.016024	0.330455	down	chr1	2.13E+08	2.13E+08	+	hsa_circ_0111917	circBase	NM_0124	RPS6KC1	exonic	367
chr2:9751	-6.43745	9.438758	2.334248	0.016027	0.330455	down	chr2	97519137	97520178	-	hsa_circ_0121236	circBase	NM_0164	ANKRD3	exonic	308
chr5:6505	-6.43026	9.429469	2.32875	0.016141	0.330455	down	chr5	65054393	65118690	+		novel	NM_0207	NLN	sense overl	64297
chr11:745	-6.41221	9.404885	2.314477	0.01644	0.330455	down	chr11	74500670	74521375	+	hsa_circ_0096435	circBase	NM_0010	RNF169	exonic	221
chr13:423	-6.41128	9.419669	2.321213	0.016298	0.330455	down	chr13	42385360	42442613	-	hsa_circ_0030110	circBase	NM_0150	VWA8	exonic	983
chrX:1994	-6.41128	9.419669	2.321213	0.016298	0.330455	down	chrX	19947902	19955650	-	hsa_circ_0140057	circBase	NM_1982	CXorf23	exonic	274
chr1:4234	-6.4053	9.395918	2.309806	0.016539	0.330455	down	chr1	42342168	42342355	-		novel	NM_0245	HIVEP3	intronic	187
chr3:4775	-6.39473	10.78247	2.550693	0.012164	0.330455	down	chr3	47752172	47777616	-		novel	NM_0030	SMARCC	exonic	435
chr7:1519	-6.35734	9.364232	2.290208	0.016961	0.330455	down	chr7	1.52E+08	1.52E+08	-	hsa_circ_0005823	circBase	NM_1706	KMT2C	exonic	192
chr2:5579	-6.35734	9.364232	2.290208	0.016961	0.330455	down	chr2	55791448	55806917	-	hsa_circ_0120446	circBase	NM_0204	PPP4R3B	exonic	640
chr1:3830	-6.35689	9.379757	2.296823	0.016817	0.330455	down	chr1	38305591	38323381	-		novel	NM_0059	MTF1	exonic	698
chr17:356	-6.34179	10.89113	2.591686	0.011549	0.330455	down	chr17	35640167	35646430	-	hsa_circ_0000759	circBase	NM_1988	ACACA	exonic	383
chr1:1624	-6.32924	9.359874	2.284857	0.017079	0.330455	down	chr1	1.62E+08	1.62E+08	+		novel	NM_1446	UHMK1	exonic	756
chr5:6847	-6.3292	9.34363	2.277778	0.017235	0.330455	down	chr5	68470703	68471364	+	hsa_circ_0001495	circBase	NM_0319	CCNB1	exonic	378
chr17:290	-6.32029	9.348498	2.27882	0.017212	0.330455	down	chr17	29070318	29096983	+		novel	ENST000	SUZ12P	exonic	529
chr1:1558	-6.29472	10.92647	2.583057	0.011676	0.330455	down	chr1	1.56E+08	1.56E+08	-	hsa_circ_0000139	circBase	NM_0322	GON4L	exonic	531
chr2:2320	-6.27911	9.311246	2.258834	0.017662	0.330455	down	chr2	2.32E+08	2.32E+08	+	hsa_circ_0006879	circBase	NM_0012	ARMC9	exonic	257
chr1:1761	-6.27911	9.311246	2.258834	0.017662	0.330455	down	chr1	1.76E+08	1.76E+08	-	hsa_circ_0008566	circBase	NM_0224	RFWD2	exonic	403
chr6:1706	-6.27183	9.318574	2.260919	0.017614	0.330455	down	chr6	1.71E+08	1.71E+08	+	hsa_circ_0001666	circBase	NM_0324	FAM120B	exonic	2038
chr2:7158	-6.27183	9.318574	2.260919	0.017614	0.330455	down	chr2	71582848	71595658	+	hsa_circ_0120880	circBase	NM_0144	ZNF638	exonic	825
chrX:1228	-6.26885	10.8274	2.496604	0.013027	0.330455	down	chrX	1.23E+08	1.23E+08	-		novel	NM_0010	THOC2	exonic	379
chr13:206	-6.26242	9.306903	2.255115	0.017747	0.330455	down	chr13	20635192	20641530	+	hsa_circ_0100001	circBase	NM_0034	ZMYM2	exonic	714
chr7:7720	-6.24924	9.289602	2.246533	0.017944	0.330455	down	chr7	77200394	77230123	+	hsa_circ_0080835	circBase	NM_0028	PTPN12	exonic	596
chr4:1517	-6.24924	9.289602	2.246533	0.017944	0.330455	down	chr4	1.52E+08	1.52E+08	-	hsa_circ_0008618	circBase	NM_0067	LRBA	exonic	409
chr14:457	-6.23142	10.63476	2.339254	0.015925	0.330455	down	chr14	45700343	45706924	-	hsa_circ_0101888	circBase	NM_0183	MIS18BP	exonic	451
chr20:732	-6.22692	10.78993	2.441534	0.013972	0.330455	down	chr20	732453	733428	-		novel	T212270	G049020	antisense	975
chr12:508	-6.21855	10.61918	2.317616	0.016373	0.330455	down	chr12	50821544	50835426	+	hsa_circ_0026228	circBase	NM_0528	LARP4	exonic	786
chr5:3899	-6.21444	9.246543	2.223554	0.018486	0.330455	down	chr5	38991050	39021238	-	hsa_circ_0006308	circBase	NM_1527	RICTOR	exonic	486
chr15:590	-6.21444	9.246543	2.223554	0.018486	0.330455	down	chr15	59036111	59036948	-		novel	ENST000	ADAM10	intronic	837
chr4:1517	-6.21444	9.246543	2.223554	0.018486	0.330455	down	chr4	1.52E+08	1.52E+08	-		novel	NM_0067	LRBA	exonic	2404
chr2:2019	-6.21444	9.246543	2.223554	0.018486	0.330455	down	chr2	2.02E+08	2.02E+08	+	hsa_circ_0002860	circBase	NM_0038	CFLAR	exonic	930
chr1:2159	-6.21444	9.246543	2.223554	0.018486	0.330455	down	chr1	21599191	21599404	-	hsa_circ_0008563	circBase	NM_0013	ECE1	exonic	213
chr1:2005	-6.21444	9.246543	2.223554	0.018486	0.330455	down	chr1	2.01E+08	2.01E+08	-		novel	NM_0148	KIF14	exonic	1358
chr19:556	-6.21444	9.246543	2.223554	0.018486	0.330455	down	chr19	55610151	55614936	-	hsa_circ_0000958	circBase	NM_0176	PPP1R12	exonic	380
chr1:5186	-6.21444	9.246543	2.223554	0.018486	0.330455	down	chr1	51860052	51887530	-	hsa_circ_0012423	circBase	NM_0019	EPS15	exonic	1079
chr5:1261	-6.21444	9.246543	2.223554	0.018486	0.330455	down	chr5	1.26E+08	1.26E+08	+	hsa_circ_0007726	circBase	NM_0055	LMNB1	exonic	580
chr14:735	-6.21444	9.246543	2.223554	0.018486	0.330455	down	chr14	73550201	73554869	+	hsa_circ_0102553	circBase	NM_0212	RBM25	exonic	219
chr16:746	-6.21444	9.246543	2.223554	0.018486	0.330455	down	chr16	74694829	74695349	-	hsa_circ_0004172	circBase	NM_0181	RFWD3	exonic	520
chr9:7995	-6.21444	9.246543	2.223554	0.018486	0.330455	down	chr9	79952170	79959197	+		novel	NM_0151	VPS13A	exonic	1060
chr14:103	-6.21444	9.246543	2.223554	0.018486	0.330455	down	chr14	1.04E+08	1.04E+08	+		novel	NM_0023	MARK3	exonic	1498
chr10:951	-6.21309	10.77877	2.42577	0.014256	0.330455	down	chr10	95140975	95148911	-	hsa_circ_0005898	circBase	NM_0134	MYOF	exonic	420
chr2:1530	-6.21203	9.276043	2.236683	0.018174	0.330455	down	chr2	1.53E+08	1.53E+08	-	hsa_circ_0117652	circBase	NM_0058	STAM2	exonic	664
chr3:1252	-6.19685	9.256504	2.226573	0.018414	0.330455	down	chr3	1.25E+08	1.25E+08	-	hsa_circ_0067115	circBase	NM_0037	SNX4	exonic	334
chr18:294	-6.18779	9.245676	2.220169	0.018567	0.330455	down	chr18	29418102	29420186	-		novel	NM_0149	TRAPPC8	sense overl	2084
chr18:193	-6.18741	10.75102	2.384027	0.015037	0.330455	down	chr18	19345732	19399607	+	hsa_circ_0000836	circBase	NM_0207	MIB1	exonic	1600
chr16:705	-6.18117	9.254293	2.223735	0.018481	0.330455	down	chr16	70524232	70531958	-		novel	NM_0153	COG4	exonic	515
chr18:337	-6.17796	10.58841	2.277966	0.017231	0.330455	down	chr18	33775219	33783152	+	hsa_circ_0003552	circBase	NM_0179	MOCOS	exonic	876
chr14:888	-6.17086	9.241957	2.216447	0.018657	0.330455	down	chr14	88883054	88899556	+	hsa_circ_0102765	circBase	NM_0184	SPATA7	exonic	922
chr7:4745	-6.16734	10.73719	2.365572	0.015396	0.330455	down	chr7	47451324	47467974	-	hsa_circ_0080170	circBase	NM_0227	TNS3	exonic	399
chr11:111	-6.16734	10.73719	2.365572	0.015396	0.330455	down	chr11	1.12E+08	1.12E+08	+	hsa_circ_0094993	circBase	NM_0019	DLAT	exonic	617
chr16:564	-6.1508	10.72286	2.345332	0.015801	0.330455	down	chr16	56488685	56504703	+		novel	NM_0182	OGFOD1	sense overl	16018
chr5:1488	-6.14964	9.232206	2.209247	0.018832	0.330455	down	chr5	1.49E+08	1.49E+08	-		novel	NM_0018	CSNK1A1	sense overl	27655
chr3:1961	-6.14688	10.71831	2.338655	0.015937	0.330455	down	chr3	1.96E+08	1.96E+08	-	hsa_circ_0005051	circBase	NM_0155	UBXN7	exonic	179
chr4:7705	-6.14116	10.55004	2.227399	0.018394	0.330455	down	chr4	77055327	77065626	-	hsa_circ_0070039	circBase	NM_0174	NUP54	exonic	643
chr16:683	-6.12366	10.69883	2.311861	0.016495	0.330455	down	chr16	68321648	68325564	+	hsa_circ_0039955	circBase	NM_0039	SLC7A6	exonic	499
chr5:1389	-6.12355	9.200327	2.190234	0.019302	0.330455	down	chr5	1.39E+08	1.39E+08	+	hsa_circ_0007452	circBase	NM_0033	UBE2D2	exonic	278
chr7:8062	-6.12355	9.200327	2.190234	0.019302	0.330455	down	chr7	8062112	8110761	+	hsa_circ_0134783	circBase	NM_1384	GLCCI1	exonic	568
chr22:413	-6.12097	10.6957	2.307308	0.016592	0.330455	down	chr22	41325290	41326947	+		novel	ENST000	XPNPEP3	intronic	1657
chr13:438	-6.11708	9.209347	2.194192	0.019203	0.330455	down	chr13	43872481	43900661	-	hsa_circ_0100536	circBase	NM_0179	ENOX1	exonic	410
chr3:1013	-6.11355	9.18856	2.183099	0.019481	0.330455	down	chr3	1.01E+08	1.01E+08	-	hsa_circ_0121363	circBase	NM_0144	ZBTB11	exonic	1881
chr14:736	-6.08441	9.186979	2.179304	0.019578	0.330455	down	chr14	73664738	73678650	+	hsa_circ_0032509	circBase	NM_0000	PSEN1	exonic	360
chr6:1448	-6.05624	9.153459	2.158097	0.020125	0.330455	down	chr6	1.45E+08	1.45E+08	+		novel	NM_0071	UTRN	exonic	551
chr6:1011	-6.05066	9.163816	2.16286	0.020001	0.330455	down	chr6	1.01E+08	1.01E+08	-	hsa_circ_0008321	circBase	NM_0068	ASCC3	exonic	414
chr6:1750	-6.02272	9.114862	2.137121	0.020682	0.330455	down	chr6	17507399	17543374	+		novel	NM_0063	CAP2	exonic	909
chr9:3554	-6.01945	10.60695	2.188019	0.019357	0.330455	down	chr9	35546426	35548532	+	hsa_circ_0002702	circBase	ENST000	RUSC2	exonic	2106
chr2:1915	-6.01613	9.14027	2.148302	0.020383	0.330455	down	chr2	1.92E+08	1.92E+08	+	hsa_circ_0002024	circBase	NM_0059	NAB1	exonic	1024
chr4:1488	-6.00272	10.59319	2.170366	0.019806	0.330455	down	chr4	1.49E+08	1.49E+08	+	hsa_circ_0001449	circBase	NM_0246	ARHGAP	exonic	222
chr1:2109	-5.98634	9.089776	2.122087	0.021092	0.330455	down	chr1	21091869	21106404	-	hsa_circ_0111822	circBase	NM_0162	HP1BP3	exonic	794
chr10:134	-5.98634	9.089776	2.122087	0.021092	0.330455	down	chr10	1.34E+08	1.35E+08	+	hsa_circ_0020537	circBase	NM_0055	INPP5A	exonic	399
chr14:207	-5.98634	9.089776	2.122087	0.021092	0.330455	down	chr14	20793697	20797532	-	hsa_circ_0004766	circBase	NM_0211	CCNB1IP	exonic	393
chr2:1125	-5.98553	9.104963	2.129177	0.020898	0.330455	down	chr2	1.13E+08	1.13E+08	-		novel	NM_0226	ANAPC1	exonic	2491

chr1:4772	-5.98076	9.116329	2.134089	0.020764	0.330455	down	chr1	47725960	47737913	-	hsa_circ_0113481	circBase	NM_0030	STIL	exonic	860
chr7:8043	-5.98076	9.116329	2.134089	0.020764	0.330455	down	chr7	8043537	8110761	+	hsa_circ_0134778	circBase	NM_1384	GLCCI1	exonic	720
chr3:1941	-5.98076	9.116329	2.134089	0.020764	0.330455	down	chr3	1.94E+08	1.94E+08	-		novel	ENST000	ATP13A3	intronic	610
chr11:1290	-5.98038	9.11584	2.133828	0.020771	0.330455	down	chr11	12901254	12923660	+	hsa_circ_0003015	circBase	NM_0219	TEAD1	exonic	543
chr10:702	-5.9735	9.091568	2.121844	0.021099	0.330455	down	chr10	70218860	70229920	-	hsa_circ_0009172	circBase	NR_1022	DNA2	exonic	645
chr5:1679	-5.97295	10.40101	2.035077	0.023639	0.330455	down	chr5	1.68E+08	1.68E+08	-	hsa_circ_0128563	circBase	NM_0245	PANK3	exonic	681
chr4:9549	-5.96381	10.55314	2.118374	0.021195	0.330455	down	chr4	95494501	95507595	+	hsa_circ_0070467	circBase	NM_0064	PDLIM5	exonic	672
chr9:1141	-5.95605	10.5504	2.115704	0.021269	0.330455	down	chr9	1.14E+08	1.14E+08	-		novel	uc010muc	KIAA0368	exonic	790
chr1:2333	-5.94854	9.063663	2.106855	0.021516	0.330455	down	chr1	2.33E+08	2.33E+08	-	hsa_circ_0112418	circBase	NM_0148	PCNXL2	exonic	915
chr12:100	-5.94854	9.063663	2.106855	0.021516	0.330455	down	chr12	1E+08	1E+08	-	hsa_circ_0027845	circBase	NM_0150	UHRF1BP	exonic	2532
chr6:1070	-5.94854	9.063663	2.106855	0.021516	0.330455	down	chr6	1.07E+08	1.07E+08	-	hsa_circ_0008514	circBase	NM_0327	RTN4IP1	exonic	463
chr13:964	-5.94854	9.063663	2.106855	0.021516	0.330455	down	chr13	96412293	96413052	+		novel	NM_0062	DNAJC3	exonic	302
chr17:738	-5.94854	9.063663	2.106855	0.021516	0.330455	down	chr17	73835919	73837082	-	hsa_circ_0045728	circBase	NM_1992	UNC13D	exonic	486
chr5:8146	-5.94854	9.063663	2.106855	0.021516	0.330455	down	chr5	81460217	81474406	+	hsa_circ_0129844	circBase	NM_0314	ATG10	exonic	237
chr3:1252	-5.94854	9.063663	2.106855	0.021516	0.330455	down	chr3	1.25E+08	1.25E+08	-	hsa_circ_0006245	circBase	NM_0227	OSBPL11	exonic	502
chr22:460	-5.94854	9.063663	2.106855	0.021516	0.330455	down	chr22	46085591	46136418	+	hsa_circ_0008199	circBase	NM_0132	ATXN10	exonic	1057
chr1:5224	-5.94854	9.063663	2.106855	0.021516	0.330455	down	chr1	52246835	52253461	+	hsa_circ_0113540	circBase	ENST000	OSBPL9	exonic	955
chr6:1283	-5.94854	9.063663	2.106855	0.021516	0.330455	down	chr6	1.28E+08	1.28E+08	-	hsa_circ_0005771	circBase	NM_0028	PTPRK	exonic	311
chr6:3733	-5.94854	9.063663	2.106855	0.021516	0.330455	down	chr6	37336259	37344809	+	hsa_circ_0131692	circBase	NM_0039	RNF8	exonic	996
chr3:6998	-5.94854	9.063663	2.106855	0.021516	0.330455	down	chr3	69986972	69990482	+		novel	NM_0002	MITF	exonic	408
chr1:2193	-5.94854	9.063663	2.106855	0.021516	0.330455	down	chr1	2.19E+08	2.19E+08	+	hsa_circ_0004417	circBase	NM_1387	LYPLAL1	sense overl	48227
chr7:5604	-5.94854	9.063663	2.106855	0.021516	0.330455	down	chr7	56049902	56062681	+	hsa_circ_0080251	circBase	NM_0014	GBAS	exonic	339
chr8:3771	-5.94854	9.063663	2.106855	0.021516	0.330455	down	chr8	37716622	37716808	-		novel	NM_0251	RAB11FII	sense overl	186
chr11:731	-5.94854	9.063663	2.106855	0.021516	0.330455	down	chr11	73109185	73109537	+		novel			intergenic	352
chr14:714	-5.94854	9.063663	2.106855	0.021516	0.330455	down	chr14	71413631	71429048	+	hsa_circ_0032383	circBase	NM_0149	PCNX	exonic	315
chr4:1893	-5.94451	9.091977	2.119617	0.02116	0.330455	down	chr4	1.89E+08	1.89E+08	+		PMID:250	NR_0338	LINC0106	exonic	146
chr2:9666	-5.93039	9.07659	2.11148	0.021386	0.330455	down	chr2	9666239	9699330	-		novel	NM_0031	ADAM17	sense overl	33091
chr15:575	-5.88478	10.56398	2.074932	0.022434	0.330455	down	chr15	57523349	57526305	+	hsa_circ_0006380	circBase	NM_0032	TCF12	exonic	456
chr5:6486	-5.88149	10.32035	1.913179	0.027773	0.330455	down	chr5	64863339	64868113	+	hsa_circ_0072697	circBase	NM_0153	PPWD1	exonic	773
chr1:2244	-5.86913	9.041988	2.090707	0.021975	0.330455	down	chr1	2.24E+08	2.24E+08	-	hsa_circ_0016575	circBase	NM_0025	NVL	exonic	757
chr1:2445	-5.84679	9.017534	2.077319	0.022364	0.330455	down	chr1	2.45E+08	2.45E+08	-	hsa_circ_0017265	circBase	NM_0011	ADSS	exonic	257
chr3:1184	-5.83656	10.28806	1.863471	0.029676	0.330455	down	chr3	11849306	11871338	-	hsa_circ_0121562	circBase	NR_10431	TAMM41	exonic	1617
chr1:2356	-5.83655	10.45408	2.000284	0.024747	0.330455	down	chr1	2.36E+08	2.36E+08	-	hsa_circ_0002343	circBase	NM_1524	B3GALNT	exonic	539
chr22:319	-5.83617	10.44903	1.993858	0.024958	0.330455	down	chr22	31962342	31990249	+		novel	NM_0147	SFI1	sense overl	27907
chr12:108	-5.83238	9.002589	2.068732	0.022617	0.330455	down	chr12	10865809	10868382	-	hsa_circ_0025460	circBase	NM_0036	YBX3	exonic	213
chr15:935	-5.82991	9.016319	2.075616	0.022414	0.330455	down	chr15	93540186	93558139	+	hsa_circ_0036998	circBase	NM_0012	CHD2	exonic	1311
chr4:1219	-5.82991	9.016319	2.075616	0.022414	0.330455	down	chr4	1219147	1235307	-	hsa_circ_0001386	circBase	NM_0010	CTBP1	exonic	702
chr17:385	-5.8254	10.44455	1.989029	0.025117	0.330455	down	chr17	38562635	38563194	-	hsa_circ_0003457	circBase	NM_0010	TOP2A	exonic	327
chr14:353	-5.81888	10.93411	2.316773	0.016391	0.330455	down	chr14	35331249	35331528	+	hsa_circ_0006137	circBase	NM_0134	BAZ1A	exonic	279
chr5:5616	-5.80975	10.50545	2.0053	0.024584	0.330455	down	chr5	56160560	56161804	+	hsa_circ_0001485	circBase	NM_0059	MAP3K1	exonic	467
chr2:1366	-5.80308	10.2609	1.822023	0.031369	0.330455	down	chr2	1.37E+08	1.37E+08	-		novel	NM_0013	DARS	sense overl	3509
chr3:1361	-5.79308	10.40446	1.931642	0.0271	0.330455	down	chr3	1.36E+08	1.36E+08	-	hsa_circ_0067480	circBase	NM_0058	STAG1	exonic	637
chr1:3309	-5.79308	10.40446	1.931642	0.0271	0.330455	down	chr1	33093108	33100393	-	hsa_circ_0011424	circBase	NM_1785	ZBTB8OS	exonic	320
chr1:4652	-5.79097	10.48257	1.977071	0.025517	0.330455	down	chr1	46521464	46546422	-	hsa_circ_0012300	circBase	NM_0036	PIK3R3	exonic	835
chr1:4776	-5.78958	8.990177	2.059767	0.022884	0.330455	down	chr1	47767232	47776034	-	hsa_circ_0007322	circBase	NM_0030	STIL	exonic	496
chr12:508	-5.76508	8.964479	2.044819	0.023338	0.330455	down	chr12	50868605	50869067	+		novel	ENST000	LARP4	intronic	462
chr14:644	-5.749	8.948425	2.035253	0.023633	0.330455	down	chr14	64464936	64468799	+		novel	NM_0151	SYNE2	exonic	543
chr5:1427	-5.7481	8.963546	2.043485	0.023379	0.330455	down	chr5	1.43E+08	1.43E+08	-	hsa_circ_0001543	circBase	NM_0001	NR3C1	exonic	1197
chr3:1941	-5.7481	8.963546	2.043485	0.023379	0.330455	down	chr3	1.94E+08	1.94E+08	-	hsa_circ_0007863	circBase	NM_0245	ATP13A3	exonic	1068
chr3:5669	-5.73521	10.36403	1.872156	0.029333	0.330455	down	chr3	56694758	56707753	-	hsa_circ_0001315	circBase	NM_0011	FAM208A	exonic	1037
chr7:1790	-5.71523	8.914712	2.01605	0.024238	0.330455	down	chr7	17908029	17915413	-	hsa_circ_0005519	circBase	NM_0151	SNX13	exonic	397
chr1:4151	-5.71402	10.34203	1.839734	0.030633	0.330455	down	chr1	41512078	41541123	-	hsa_circ_0011938	circBase	NM_0122	SCMH1	exonic	753
chr1:2417	-5.70538	8.936405	2.026875	0.023895	0.330455	down	chr1	2.42E+08	2.42E+08	-		novel	ENST000	OPN3	sense overl	7215
chr12:123	-5.67745	10.39786	1.868297	0.029485	0.330455	down	chr12	1.24E+08	1.24E+08	-	hsa_circ_0029182	circBase	NM_0227	MPHOSPH	exonic	2771
chr8:1956	-5.67091	10.30774	1.788877	0.032799	0.330455	down	chr8	19562271	19610737	-		novel	ENST000	CSGALNA	intronic	48466
chr6:1090	-5.67012	10.90643	2.199743	0.019065	0.330455	down	chr6	10906252	10964076	+		novel	NM_0010	SYCP2L	exonic	1835
chr1:2251	-5.66962	8.885845	2.004224	0.024619	0.330455	down	chr1	2.25E+08	2.25E+08	+	hsa_circ_0112170	circBase	NM_0013	DNAH14	exonic	1391
chr14:382	-5.66135	8.908735	2.011288	0.024391	0.330455	down	chr14	38256672	38266152	+	hsa_circ_0101775	circBase	NM_0013	TTC6	exonic	763
chr12:652	-5.66024	8.892053	2.005861	0.024566	0.330455	down	chr12	65258447	65260683	+	hsa_circ_0027440	circBase	NM_0152	TBC1D30	exonic	253
chr10:167	-5.63277	10.3473	1.794115	0.032568	0.330455	down	chr10	16794537	16824083	-	hsa_circ_0006577	circBase	NM_0124	RSU1	exonic	489
chr12:650	-5.62481	10.86623	2.144009	0.020498	0.330455	down	chr12	65078566	65092490	+		novel	NM_1781	RASSF3	sense overl	13924
chr1:2369	-5.62444	10.27029	1.733073	0.035366	0.330455	down	chr1	2.37E+08	2.37E+08	+		novel	NM_0002	MTR	exonic	1704
chr6:2823	-5.61607	8.880515	2.001883	0.024695	0.330455	down	chr6	28239631	28240564	+	hsa_circ_0131616	circBase	NM_1527	ZSCAN26	exonic	199
chr3:1139	-5.6137	8.855597	2.19769	0.019116	0.330455	down	chr3	11399891	11483059	+		novel	NM_0063	ATG7	sense overl	83168
chr5:4360	-5.6137	8.855597	2.19769	0.019116	0.330455	down	chr5	43609244	43619221	+		novel	NM_0123	NNT	exonic	740
chr1:5897	-5.6137	8.855597	2.19769	0.019116	0.330455	down	chr1	58971731	59000003	-	hsa_circ_0006281	circBase	NM_1452	OMA1	exonic	636
chr5:7313	-5.6137	8.855597	2.19769	0.019116	0.330455	down	chr5	73136304	73136585	+	hsa_circ_0005777	circBase	NM_0010	ARHGEF2	exonic	281
chr13:418	-5.6137	8.855597	2.19769	0.019116	0.330455	down	chr13	41890981	41905505	+	hsa_circ_0030066	circBase	NM_0185	NAA16	exonic	853
chrM:3568	-5.6137	8.855597	2.19769	0.019116	0.330455	down	chrM	3567	3790	+		novel	T370381	G087361	intronic	223
chr12:474	-5.6137	8.855597	2.19769	0.019116	0.330455	down	chr12	47497895	47501947	+	hsa_circ_0004650	circBase	NM_0012	PCED1B	sense overl	4052
chr13:214	-5.6137	8.855597	2.19769	0.019116	0.330455	down	chr13	21436855	21442840	-	hsa_circ_0029684	circBase	NM_0224	XPO4	exonic	248
chr7:1524	-5.6137	8.855597	2.19769	0.019116	0.330455	down	chr7	1.52E+08	1.53E+08	+	hsa_circ_0083062	circBase	NM_0204	ACTR3B	exonic	851
chr11:464	-5.6137	8.855597	2.19769	0.019116	0.330455	down	chr11	46455023	46529920	-	hsa_circ_0095872	circBase	NM_0177	AMBRA1	exonic	817
chr20:319	-5.6137	8.855597	2.19769	0.019116	0.330455	down	chr20	31954664	31967497	-	hsa_circ_0114926	circBase	NM_0160	CDK5RAI	exonic	666
chr5:1341	-5.6137	8.855597	2.19769	0.019116	0.330455	down	chr5	1.34E+08	1.34E+08	+	hsa_circ_0074009	circBase	NM_0148	DDX46	exonic	1341
chr11:280	-5.6137	8.855597	2.19769	0.019116	0.330455	down	chr11	28098553	28113060	-	hsa_circ_0007496	circBase	NM_0312	KIF18A	exonic	942
chr9:1100	-5.6137	8.855597	2.19769	0.019116	0.330455	down	chr9	1.1E+08	1.1E+08	+	hsa_circ_0087863	circBase	NM_0028	RAD23B	exonic	619
chr2:2900	-5.6137	8.855597	2.19769	0.019116	0.330455	down	chr2	29006772	29011675	+	hsa_circ_0007439	circBase	NM_0027	PPP1CB	exonic	224
chr7:1341	-5.6137	8.855597	2.19769	0.019116	0.330455	down	chr7	1.34E+08	1.34E+08	+		novel	NM_0202	AKR1B10	sense overl	50536
chr3:1827	-5.6137	8.855597	2.19769	0.019116	0.330455	down	chr3	1.83E+08	1.83E+08	-	hsa_circ_0122928	circBase	NM_0201	MCCC1	exonic	672
chr15:510	-5.6137	8.855597	2.19769	0.019116	0.330455	down	chr15	51012136	51041943	-	hsa_circ_0103790	circBase	NM_0328	SPPL2A	exonic	1422
chr19:4424	-5.60807	10.32609	1.763642	0.033934	0.330455	down	chr19	44248923	44254899	-	hsa_circ_0008781	circBase	NM_0191	SMG9	exonic	707
chr1:2239	-5.59379	10.07323	1.618787	0.041331	0.333399	down	chr1	2.24E+08	2.24E+08	-		novel	NM_0054	TP53BP2	exonic	329
chr2:1486	-5.59229	10.83714	2.104508	0.021582	0.330455	down	chr2	1.49E+08	1.49E+08	+	hsa_circ_0001073	circBase	NM_0016	ACVR2A	exonic	473
chr9:1148	-5.58706	10.23388	1.680288	0.037996	0.330455	down	chr9	1.15E+08	1.15E+08	-	hsa_circ_0137723	circBase	NM_0224	SUSD1	exonic	582
chr1:2310	-5.56934	8.851721	1.99345	0.024971	0.330455	down	chr1	2.31E+08	2.31E+08	-	hsa_circ_0004531	circBase	NM_0245	TTC13	exonic	401
chr2:3210	-5.56861	8.850998	1.993277	0.024977	0.330455	down	chr2	32108454	32157204	-	hsa_circ_0004215	circBase	NM_0159	MEMO1	exonic	514
chr1:1020	-5.56209	10.0536	1.604864	0.04213	0.333399	down	chr1	10209241	10221344	+	hsa_circ_0009701	circBase	NM_0060	UBE4B	exonic	607
chr16:302	-5.54341	8.813884	1.983145	0.025313	0.330455	down	chr16	30287333	30305508	-		novel	NR_0024	SMG1P5	exonic	2427
chr1:1214	-5.54155	10.94684	2.146907	0.02042	0.330455	down	chr1	1.21E+08	1.21E+08	+		novel	T018636	G004213	sense overl	1019
chr6:1356	-5.52105	8.822331	1.98636	0.025206	0.330455	down	chr6	1.36E+08	1.36E+08	-	hsa_circ_0005214	circBase	NM_0176	AHI1	exonic	320
chr5:1104	-5.52105	8.822331	1.98636	0.025206	0.330455	down	chr5	1.1E+08	1.1E+08	-		novel	NM_1392	WDR36	exonic	716
chr5:1123	-5.52105	8.822331	1.98636	0.025206	0.330455	down	chr5	1.12E+08	1.12E+08	+	hsa_circ_0073608	circBase	NM_1526	DCP2	exonic	753
chr9:8103	-5.51001	10.17114	1.595373	0.042684	0.333399	down	chr9	81035195	81038128	+	hsa_circ_0139039	circBase	T360816	G085208	sense overl	2933
chr17:412	-5.50984	8.811641	1.983506	0.025301	0.330455	down	chr17	41219624	41226538	-	hsa_circ_0043954	circBase	NM_0072	BRCA1	exonic	590
chr2:2069	-5.50728	10.24105	1.639878	0.040153	0.330979	down	chr2	2.07E+08	2.07E+08	+	hsa_circ_0002431	circBase	NM_0050	NDUFS1	exonic	331
chr2:4760	-5.49291	8.78396	1.974687	0.025598	0.330455	down	chr2	47600601	47602438	+	hsa_circ_0120175	circBase	NM_0023	EPCAM	exonic	415
chr8:1243	-5.49288	10.14838	1.577558	0.043745	0.33431	down	chr8	1.24E+08	1.24E+08	-	hsa_circ_0085465	circBase	NM_0141	ATAD2	exonic	333
chr1:9464	-5.48981	9.99632	1.565949	0.044452	0.33431	down	chr1	94643167	94649844	-	hsa_circ_0013224	circBase	NM_0048	ARHGAP	exonic	796
chr2:1780	-5.47108	8.792316	1.978844	0.025458	0.330455	down	chr2	1.78E+08	1.78E+08	-	hsa_circ_0057168	circBase	NM_0061	NFE2L2	exonic	549
chr3:1968	-5.46318	10.20866	1.594619	0.042728	0.333399	down	chr3	1.97E+08	1.97E+08	-	hsa_circ_0008500	circBase	NM_0040	DLG1	exonic	381
chr11:701	-5.46301	8.771884	1.972224	0.025681	0.330455	down	chr11	70178065	70202360	+		novel	NM_0036	PPFIA1	exonic	1505
chr1:5563	-5.46301	8.771884	1.972224	0.025681	0.330455	down	chr1	55637228	55643802	-		novel	NM_0153	USP24	sense overl	6574
chr18:196	-5.45538	9.957909	1.542184	0.045938	0.33431	down	chr18	196636	204692	+	hsa_circ_0005653	circBase	NM_0051	USP14	exonic	701
chr11:108	-5.43875	9.943946	1.533099	0.046521	0.335138	down	chr11	1.08E+08	1.08E+08	+		novel	NM_0000	ATM	exonic	2307
chr7:6840	-5.43875	9.943946	1.533099	0.046521	0.335138	down	chr7	6840578	6841154	-	hsa_circ_0007135	circBase	NM_1980	CCZ1B	exonic	287
chr5:7404	-5.38967	8.737531	1.966965	0.025861	0.330455	down	chr5	74041502	74046492	-	hsa_circ_0129604	circBase	NM_0323	GFM2	exonic	419
chr12:508	-5.37686	10.05195	1.506838	0.048251	0.336436	down	chr12	50824277	50834332	+	hsa_circ_0098715	circBase	NM_0528	LARP4	exonic	428
chr19:346	-5.35307	10.04368	1.500062	0.048709	0.336878	down	chr19	34685382	34706566	+	hsa_circ_0008030	circBase	NM_0155	LSM14A	exonic	660
chr9:1141	-5.35241	8.707607	1.958957	0.026136	0.330455	down	chr9	1.14E+08	1.14E+08	-	hsa_circ_0137701	circBase	NM_0010	KIAA0368	exonic	1611
chr5:3852	-5.3521	10.0512	1.504152	0.048432	0.336475	down	chr5	38523520	38530768	-	hsa_circ_0072309	circBase	NM_0023	LIFR	exonic	580
chr1:6229	-5.35141	10.03363	1.494341	0.049099	0.337946	down	chr1	62293093	62350080	+		PMID:252	NM_1768	INADL	exonic	1313
chr12:117	-5.33352	8.705135	1.960551	0.026081	0.330455	down	chr12	1.17E+08	1.17E+08	+	hsa_circ_0006150	circBase	NM_0121	FBXW8	exonic	404
chr5:1398	-5.31562	8.684568	2.102769	0.021631	0.330455	down	chr5	1.4E+08	1.4E+08	+		novel	uc003lfq.2	ANKHD1	exonic	1379
chr1:1699	-5.30986	8.698251	1.960804	0.026072	0.330455	down	chr1	1.7E+08	1.7E+08	-	hsa_circ_0111121	circBase	NM_0149	KIFAP3	sense overl	53891
chrX:6726	-5.29197	11.02518	2.048223	0.023234	0.330455	down	chrX	67266823	67273652	-	hsa_circ_0007445	circBase	NM_0025	OPHN1	sense overl	6817
chr5:6165	-5.26519	8.664243	1.95276	0.026352	0.330455	down	chr5	61659004	61662308	+	hsa_circ_0129299	circBase	NM_0045	KIF2A	exonic	527
chr1:2335	-5.26519	8.664243	1.95276	0.026352	0.330455	down	chr1	23356961	23377013	+	hsa_circ_0009061	circBase	NM_0150	KDM1A	exonic	300
chr3:1366	-5.25431	8.650693	2.085158	0.022135	0.330455	down	chr3	1.37E+08	1.37E+08	+	hsa_circ_0067492	circBase	NM_1447	IL20RB	exonic	737

chr19:588	-5.25184	8.66545	1.956073	0.026236	0.330455	down	chr19	58805463	58806947	+	hsa_circ_0005959	circBase	ENST000	AC010642	exonic	1484
chr3:1597	-5.25184	8.66545	1.956073	0.026236	0.330455	down	chr3	15976031	15988362	+		novel	uc003caq.	GALNT15	sense overl	12331
chr6:7654	-5.25014	10.42429	1.672955	0.038378	0.330455	down	chr6	76545617	76551095	+	hsa_circ_0132333	circBase	NM_0049	MYO6	exonic	319
chr1:2240	-5.23176	8.640154	1.946204	0.026581	0.330455	down	chr1	22404921	22417994	+	hsa_circ_0007604	circBase	GSE61474	GSE61474	intronic	13073
chr11:622	-5.23176	8.640154	1.946204	0.026581	0.330455	down	chr11	62289033	62289417	-		novel	ENST000	AHNAK	intronic	384
chr9:3658	-5.23176	8.640154	1.946204	0.026581	0.330455	down	chr9	36581640	36589649	+	hsa_circ_0005460	circBase	NM_0147	MELK	exonic	299
chr6:1632	-5.21006	10.6533	1.766371	0.033809	0.330455	down	chr6	16326624	16328701	-	hsa_circ_0007132	circBase	NM_0003	ATXN1	exonic	2077
chr12:508	-5.1914	8.631868	1.951576	0.026393	0.330455	down	chr12	50824277	50847455	+	hsa_circ_0098716	circBase	NM_0528	LARP4	exonic	695
chr10:119	-5.18833	8.61488	2.065067	0.022726	0.330455	down	chr10	11990367	12056183	-		novel	NM_0155	UPF2	exonic	2029
chr7:1629	-5.18833	8.61488	2.065067	0.022726	0.330455	down	chr7	16298014	16317851	-	hsa_circ_0079480	circBase	NM_0011	ISPD	exonic	284
chr4:1288	-5.18833	8.61488	2.065067	0.022726	0.330455	down	chr4	1.29E+08	1.29E+08	-	hsa_circ_0070923	circBase	NM_1527	MFSD8	exonic	800
chr1:6232	-5.18833	8.61488	2.065067	0.022726	0.330455	down	chr1	62321701	62350080	+	hsa_circ_0012779	circBase	NM_1768	INADL	exonic	1019
chr15:594	-5.18833	8.61488	2.065067	0.022726	0.330455	down	chr15	59464095	59510286	-		novel	NM_0049	MYO1E	exonic	1570
chr1:1682	-5.18833	8.61488	2.065067	0.022726	0.330455	down	chr1	1.68E+08	1.68E+08	+		novel	NM_0051	TBX19	exonic	462
chr9:9874	-5.18833	8.61488	2.065067	0.022726	0.330455	down	chr9	98740342	98766983	+	hsa_circ_0008720	circBase	NM_0202	ERCC6L2	exonic	337
chr3:6754	-5.18833	8.61488	2.065067	0.022726	0.330455	down	chr3	67546221	67579610	-	hsa_circ_0005657	circBase	NM_0038	SUCLG2	exonic	836
chr5:3244	-5.18833	8.61488	2.065067	0.022726	0.330455	down	chr5	32441399	32443035	-		novel	ENST000	ZFR	intronic	1636
chr22:291	-5.18833	8.61488	2.065067	0.022726	0.330455	down	chr22	29120964	29130715	-	hsa_circ_0001215	circBase	NM_0071	CHEK2	exonic	598
chrX:2485	-5.18833	8.61488	2.065067	0.022726	0.330455	down	chrX	24859768	24906239	+		novel	NM_0169	POLA1	exonic	428
chr15:566	-5.18833	8.61488	2.065067	0.022726	0.330455	down	chr15	56665638	56687032	+	hsa_circ_0103943	circBase	NM_1985	TEX9	exonic	709
chr22:221	-5.18833	8.61488	2.065067	0.022726	0.330455	down	chr22	22160138	22162135	-	hsa_circ_0008870	circBase	NM_0027	MAPK1	exonic	373
chr20:290	-5.18833	8.61488	2.065067	0.022726	0.330455	down	chr20	2903852	2945848	+	hsa_circ_0001126	circBase	NM_0028	PTPRA	exonic	543
chr4:4009	-5.18833	8.61488	2.065067	0.022726	0.330455	down	chr4	40098846	40155887	+		novel	NM_0181	N4BP2	sense overl	57041
chr19:496	-5.18833	8.61488	2.065067	0.022726	0.330455	down	chr19	49610179	49610372	+		novel	ENST000	SNRNP70	intronic	193
chrX:7064	-5.18833	8.61488	2.065067	0.022726	0.330455	down	chrX	70641158	70644088	+	hsa_circ_0091024	circBase	NM_0046	TAF1	exonic	369
chr1:2109	-5.18833	8.61488	2.065067	0.022726	0.330455	down	chr1	21094076	21107033	-	hsa_circ_0111825	circBase	NM_0162	HP1BP3	exonic	835
chrX:7726	-5.18833	8.61488	2.065067	0.022726	0.330455	down	chrX	77264598	77271378	+	hsa_circ_0140627	circBase	ENST000	ATP7A	exonic	919
chrX:6773	-5.18833	8.61488	2.065067	0.022726	0.330455	down	chrX	67731690	67756578	+		novel	NM_1738	YIPF6	sense overl	24888
chr12:2714	-5.18833	8.61488	2.065067	0.022726	0.330455	down	chr12	27148169	27156323	-	hsa_circ_0098248	circBase	NM_0165	TM7SF3	exonic	599
chr10:126	-5.18833	8.61488	2.065067	0.022726	0.330455	down	chr10	1.27E+08	1.27E+08	-	hsa_circ_0002133	circBase	NM_0013	CTBP2	sense overl	35847
chr16:197	-5.18833	8.61488	2.065067	0.022726	0.330455	down	chr16	19721830	19722762	-		novel	NM_0010	KNOP1	exonic	147
chr5:1382	-5.18833	8.61488	2.065067	0.022726	0.330455	down	chr5	1.38E+08	1.38E+08	+	hsa_circ_0128100	circBase	NM_0019	CTNNA1	exonic	327
chr16:586	-5.18833	8.61488	2.065067	0.022726	0.330455	down	chr16	58633139	58633415	-	hsa_circ_0105679	circBase	NM_0162	CNOT1	exonic	276
chr12:425	-5.18833	8.61488	2.065067	0.022726	0.330455	down	chr12	42591912	42604482	-		novel	NM_0011	YAF2	intronic	12570
chr14:103	-5.18833	8.61488	2.065067	0.022726	0.330455	down	chr14	1.04E+08	1.04E+08	+	hsa_circ_0008282	circBase	NM_0023	MARK3	sense overl	6317
chr5:1398	-5.18833	8.61488	2.065067	0.022726	0.330455	down	chr5	1.4E+08	1.4E+08	+	hsa_circ_0001541	circBase	NM_0177	ANKHD1	exonic	625
chr9:1250	-5.13926	8.595614	1.94372	0.026669	0.330455	down	chr9	1.25E+08	1.25E+08	+	hsa_circ_0003250	circBase	NM_1387	MRRF	exonic	367
chr2:3158	-5.12832	8.597466	1.948934	0.026485	0.330455	down	chr2	31587023	31621565	-		novel	NM_0003	XDH	exonic	2325
chr15:589	-5.0725	8.560555	1.941285	0.026755	0.330455	down	chr15	58919898	58974513	-		novel	NM_0011	ADAM10	exonic	1154
chrX:1231	-5.0005	8.523426	1.937599	0.026887	0.330455	down	chrX	1.23E+08	1.23E+08	+		novel	NM_0066	STAG2	exonic	838
chr14:455	-5.0005	8.523426	1.937599	0.026887	0.330455	down	chr14	45587230	45599993	-	hsa_circ_0101874	circBase	NM_0020	FKBP3	sense overl	12763
chr1:5332	-4.99322	8.526029	1.945867	0.026593	0.330455	down	chr1	53322669	53329829	+	hsa_circ_0002361	circBase	NM_0010	ZYG11A	exonic	1070
chr1:1135	-4.92058	8.488901	1.944024	0.026658	0.330455	down	chr1	11356008	11357828	+		novel	ENST000	UBIAD1	sense overl	1820
chr20:182	-4.91579	10.40173	1.484031	0.049811	0.340107	down	chr20	18278628	18287037	+	hsa_circ_0006099	circBase	NM_0012	ZNF133	exonic	472
chr2:1827	-4.85796	8.45392	1.931655	0.0271	0.330455	down	chr2	1.83E+08	1.83E+08	+	hsa_circ_0002844	circBase	NR_10984	SSFA2	exonic	583
chr18:920	-4.84582	8.447277	1.929258	0.027186	0.330455	down	chr18	9208654	9221997	+	hsa_circ_0003652	circBase	NM_0152	ANKRD12	exonic	639
chr6:1462	-4.84582	8.447277	1.929258	0.027186	0.330455	down	chr6	1.46E+08	1.46E+08	-	hsa_circ_0001649	circBase	NM_1730	SHPRH	exonic	440
chr4:4166	-4.84406	8.450763	1.941651	0.026742	0.330455	down	chr4	41663436	41694401	+	hsa_circ_0126380	circBase	NM_0011	LIMCH1	exonic	1267
chr15:910	-4.84406	8.450763	1.941651	0.026742	0.330455	down	chr15	91025191	91025513	+		novel	NM_0038	IQGAP1	exonic	226
chrX:4438	-4.77827	8.414943	1.976923	0.025522	0.330455	down	chrX	44383247	44386611	-	hsa_circ_0007290	circBase	NM_1737	FUNDC1	sense overl	3364
chr7:9192	-4.77827	8.414943	1.976923	0.025522	0.330455	down	chr7	91924202	91936970	+	hsa_circ_0135061	circBase	NM_0190	ANKIB1	exonic	576
chr7:1286	-4.77372	8.413912	1.932099	0.027084	0.330455	down	chr7	1.29E+08	1.29E+08	-	hsa_circ_0001741	circBase	NM_0124	TNPO3	exonic	432
chr14:325	-4.73219	11.10164	1.754201	0.03437	0.330455	down	chr14	32559707	32563592	+	hsa_circ_0031583	circBase	NM_0011	ARHGAP	exonic	3885
chr7:9898	-4.6709	8.366691	1.926441	0.027288	0.330455	down	chr7	98985661	98987635	+	hsa_circ_0004299	circBase	NM_0057	ARPC1B	exonic	331
chr1:2040	-4.58579	8.329686	1.944192	0.026653	0.330455	down	chr1	2.04E+08	2.04E+08	+	hsa_circ_0003600	circBase	NM_0056	SOX13	exonic	776
chr11:128	-4.58579	8.329686	1.944192	0.026653	0.330455	down	chr11	1.29E+08	1.29E+08	-	hsa_circ_0000371	circBase	NM_0011	ARHGAP	exonic	177
chr10:976	-4.58407	8.330608	1.94458	0.026639	0.330455	down	chr10	97681772	97687089	+	hsa_circ_0094631	circBase	NM_0011	C10orf131	exonic	300
chr12:104	-4.58407	8.330608	1.94458	0.026639	0.330455	down	chr12	1.05E+08	1.05E+08	+		novel	ENST000	TXNRD1	intronic	9827
chr22:508	-4.58407	8.330608	1.94458	0.026639	0.330455	down	chr22	50810448	50832564	+	hsa_circ_0001258	circBase	NM_0146	PPP6R2	exonic	374
chr19:112	-4.58407	8.330608	1.94458	0.026639	0.330455	down	chr19	11230767	11238761	+	hsa_circ_0003892	circBase	NM_0005	LDLR	exonic	544
chr17:269	-4.58407	8.330608	1.94458	0.026639	0.330455	down	chr17	26918715	26920084	-	hsa_circ_0006686	circBase	NM_0064	SPAG5	exonic	1260
chr6:1898	-4.58407	8.330608	1.94458	0.026639	0.330455	down	chr6	1898176	1898362	-		novel	ENST000	GMDS	intronic	186
chr8:1460	-4.58407	8.330608	1.94458	0.026639	0.330455	down	chr8	1.46E+08	1.46E+08	-		novel	T354426	G083553	intronic	1100
chr9:1343	-4.58407	8.330608	1.94458	0.026639	0.330455	down	chr9	1.34E+08	1.34E+08	+		novel	NM_0133	PRRC2B	exonic	4667
chr2:1389	-4.58407	8.330608	1.94458	0.026639	0.330455	down	chr2	1.39E+08	1.39E+08	-		novel	GSE61474	GSE61474	sense overl	3302
chr7:9035	-4.58407	8.330608	1.94458	0.026639	0.330455	down	chr7	90350137	90356126	+		novel	ENST000	CDK14	intronic	5989
chr2:1734	-4.58407	8.330608	1.94458	0.026639	0.330455	down	chr2	1.73E+08	1.73E+08	+		novel	NR_10372	PDK1	exonic	1169
chr9:3791	-4.58407	8.330608	1.94458	0.026639	0.330455	down	chr9	37910995	37912138	-		novel	ENST000	RP11-613	intronic	1143
chr6:2985	-4.58407	8.330608	1.94458	0.026639	0.330455	down	chr6	29856889	29911700	+		novel	uc011dmb	HLA-G	intronic	54811
chr14:558	-4.58407	8.330608	1.94458	0.026639	0.330455	down	chr14	55817098	55821940	+	hsa_circ_0102165	circBase	GSE61474	GSE61474	exonic	414
chr11:622	-4.58407	8.330608	1.94458	0.026639	0.330455	down	chr11	62290554	62290776	-	hsa_circ_0096049	circBase	ENST000	AHNAK	intronic	222
chr20:554	-4.58407	8.330608	1.94458	0.026639	0.330455	down	chr20	5547378	5550885	-		novel	NM_0195	GPCPD1	exonic	211
chr11:475	-4.58407	8.330608	1.94458	0.026639	0.330455	down	chr11	47508301	47510576	-	hsa_circ_0007254	circBase	NM_0065	CELF1	exonic	320
chr20:233	-4.58407	8.330608	1.94458	0.026639	0.330455	down	chr20	23370582	23383709	-	hsa_circ_0003853	circBase	NM_0220	NAPB	exonic	463
chr6:1357	-4.58407	8.330608	1.94458	0.026639	0.330455	down	chr6	13579682	13584457	+	hsa_circ_0007218	circBase	NM_0122	SIRT5	exonic	309
chr10:101	-4.58407	8.330608	1.94458	0.026639	0.330455	down	chr10	1.02E+08	1.02E+08	+		novel	ENST000	ABCC2	exonic	994
chr7:9160	-4.58407	8.330608	1.94458	0.026639	0.330455	down	chr7	91603024	91645581	+		novel	NM_0057	AKAP9	exonic	3703
chr1:2018	-4.58407	8.330608	1.94458	0.026639	0.330455	down	chr1	2.02E+08	2.02E+08	+	hsa_circ_0003008	circBase	NM_0180	IPO9	exonic	531
chr1:1596	-4.58407	8.330608	1.94458	0.026639	0.330455	down	chr1	15964801	15978390	+	hsa_circ_0000020	circBase	NM_0323	DDI2	exonic	551
chr6:8632	-4.58407	8.330608	1.94458	0.026639	0.330455	down	chr6	86328535	86329135	-	hsa_circ_0006131	circBase	NM_0063	SYNCRIP	exonic	272
chr9:9771	-4.58407	8.330608	1.94458	0.026639	0.330455	down	chr9	97717458	97741709	+	hsa_circ_0139391	circBase	NM_0328	C9orf3	exonic	316
chr2:7365	-4.58407	8.330608	1.94458	0.026639	0.330455	down	chr2	73659325	73718625	+	hsa_circ_0055236	circBase	ENST000	ALMS1	exonic	8198
chr11:204	-4.58407	8.330608	1.94458	0.026639	0.330455	down	chr11	20424464	20448411	+	hsa_circ_0021502	circBase	NM_0057	PRMT3	exonic	288
chrX:6956	-4.58407	8.330608	1.94458	0.026639	0.330455	down	chrX	69561648	69596060	+	hsa_circ_0090951	circBase	NM_0123	KIF4A	exonic	901
chr20:495	-4.56092	8.318746	1.936802	0.026915	0.330455	down	chr20	49558567	49571822	-	hsa_circ_0115434	circBase	NM_0038	DPM1	exonic	333
chr16:534	-4.56092	8.318746	1.936802	0.026915	0.330455	down	chr16	53476569	53504834	+		novel	NM_0056	RBL2	exonic	2332
chr4:3805	-4.48793	8.286876	1.940929	0.026768	0.330455	down	chr4	38055819	38104778	+	hsa_circ_0007820	circBase	NM_0151	TBC1D1	exonic	647
chr1:1130	-4.46781	8.281099	1.952125	0.026374	0.330455	down	chr1	1.13E+08	1.13E+08	-	hsa_circ_0110460	circBase	NM_0177	ST7L	exonic	945
chr9:1282	-4.38283	8.242706	1.934961	0.026981	0.330455	down	chr9	1.28E+08	1.28E+08	-		novel	NM_0241	MAPKAP	exonic	566
chr11:945	-4.38283	8.242706	1.934961	0.026981	0.330455	down	chr11	94528176	94533477	+	hsa_circ_0000350	circBase	NM_1308	AMOTL1	exonic	1072
chr2:2088	-4.37816	8.240952	1.933263	0.027042	0.330455	down	chr2	2.09E+08	2.09E+08	-	hsa_circ_0001095	circBase	NM_0010	PLEKHM	exonic	936
chr22:161	-4.37344	8.239191	1.931505	0.027105	0.330455	down	chr22	16186810	16190791	-		novel	NR_12211	DUXAP8	exonic	619
chr12:118	-4.35406	8.23208	1.923893	0.02738	0.330455	down	chr12	1.19E+08	1.19E+08	-	hsa_circ_0028668	circBase	NM_0162	TAOK3	exonic	451
chr15:328	-4.23518	8.19176	1.909306	0.027916	0.330455	down	chr15	32819013	32825569	-		novel	ENST000	RP11-632	exonic	666
chr21:163	-4.22862	8.189812	1.905002	0.028077	0.330455	down	chr21	16337033	16415895	+		novel	ENST000	AF127577	sense overl	78862
chrX:1309	-4.22862	8.189812	1.905002	0.028077	0.330455	down	chrX	1.31E+08	1.31E+08	-		novel	NR_02697	FIRRE	sense overl	7636
chr5:1455	-4.22862	8.189812	1.905002	0.028077	0.330455	down	chr5	1.46E+08	1.46E+08	-	hsa_circ_0074414	circBase	NM_0201	LARS	exonic	469
chr12:123	-4.22862	8.189812	1.905002	0.028077	0.330455	down	chr12	1.24E+08	1.24E+08	-	hsa_circ_0029192	circBase	NM_0227	MPHOSPH	exonic	1983
chr3:9761	-4.14588	8.149915	1.910625	0.027867	0.330455	down	chr3	97611703	97634580	+		novel	NM_1536	CRYBG3	exonic	801
chr3:1338	-4.14588	8.149915	1.910625	0.027867	0.330455	down	chr3	1.34E+08	1.34E+08	-		novel	NM_0029	RYK	exonic	403
chr4:1487	-4.14588	8.149915	1.910625	0.027867	0.330455	down	chr4	1.49E+08	1.49E+08	+	hsa_circ_0125473	circBase	NM_0246	ARHGAP	sense overl	27257
chr2:2912	-4.14588	8.149915	1.910625	0.027867	0.330455	down	chr2	29129325	29137074	+	hsa_circ_0002025	circBase	NM_0151	WDR43	exonic	383
chr6:1169	-4.11193	8.1398	1.894077	0.028488	0.330455	down	chr6	1.17E+08	1.17E+08	+		novel	NM_0159	RWDD1	exonic	471
chrM:846	-3.82035	8.041123	1.854427	0.030037	0.330455	down	chrM	8468	8625	+		novel	uc011mfi.	OK/SW-cl	sense overl	157
chr1:5295	-3.82035	8.041123	1.854427	0.030037	0.330455	down	chr1	52954580	52981726	-		novel	NM_0152	ZCCHC11	exonic	797
chr22:320	-3.82035	8.041123	1.854427	0.030037	0.330455	down	chr22	32097641	32134897	-		novel	NM_1735	PRR14L	exonic	6158
chr5:1386	-3.82035	8.041123	1.854427	0.030037	0.330455	down	chr5	1.39E+08	1.39E+08	+	hsa_circ_0003816	circBase	NM_0188	MATR3	exonic	1089
chr20:135	-3.82035	8.041123	1.854427	0.030037	0.330455	down	chr20	13509079	13539761	-	hsa_circ_0059468	circBase	NM_0177	TASP1	exonic	306
chr6:1501	-3.68056	7.995391	1.818069	0.031536	0.330455	down	chr6	1.5E+08	1.5E+08	-	hsa_circ_0007771	circBase	NM_0328	LRP11	exonic	735
chr2:4577	-3.50252	7.943231	1.775836	0.03338	0.330455	down	chr2	45773870	45780869	-	hsa_circ_0005542	circBase	NM_0180	SRBD1	exonic	465
chr1:1677	-3.50252	7.943231	1.775836	0.03338	0.330455	down	chr1	1.68E+08	1.68E+08	+	hsa_circ_0000153	circBase	NM_0039	MPZL1	exonic	617
chr1:1599	-3.50252	7.943231	1.775836	0.03338	0.330455	down	chr1	1599203	1665562	-		novel	NM_0017	CDK11B	sense overl	66359
chr16:704	-3.50252	7.943231	1.775836	0.03338	0.330455	down	chr16	70497071	70497677	+	hsa_circ_0002599	circBase	NM_1450	FUK	exonic	256
chr16:705	-3.45093	7.935084	1.743397	0.034876	0.330455	down	chr16	70560489	70569323	+	hsa_circ_0105868	circBase	NM_0124	SF3B3	exonic	895
chr14:967	-3.45093	7.935084	1.743397	0.034876	0.330455	down	chr14	96794683	96813678	-	hsa_circ_0103014	circBase	NM_0180	ATG2B	exonic	2001
chr17:549	-3.45093	7.935084	1.743397	0.034876	0.330455	down	chr17	54985628	54986238	-	hsa_circ_0004311	circBase	NM_0050	TRIM25	sense overl	410
chr2:4065	-3.45093	7.935084	1.743397	0.034876	0.330455	down	chr2	40655612	40657441	-	hsa_circ_0005232	circBase	ENST000	SLC8A1	exonic	1829
chr17:805	-3.45093	7.935084	1.743397	0.034876	0.330455	down	chr17	80521229	80529746	+	hsa_circ_0000817	circBase	NM_0045	FOXK2	exonic	490
chr1:2244	-3.45093	7.935084	1.743397	0.034876	0.330455	down	chr1	2.24E+08	2.25E+08	-		novel	NM_0025	NVL	exonic	2158

chr15:100	-3.2812	7.886301	1.70766	0.036607	0.330455	down	chr15	1.01E+08	1.01E+08	-	hsa_circ_0103069	circBase	NM_1390	ADAMTS	exonic	398
chr13:965	-3.2812	7.886301	1.70766	0.036607	0.330455	down	chr13	96519592	96547558	-		novel	NM_0201	UGGT2	exonic	924
chr2:5588	-3.2812	7.886301	1.70766	0.036607	0.330455	down	chr2	55889090	55900214	-	hsa_circ_0120467	circBase	NM_0331	PNPT1	exonic	568
chr12:111	-3.2812	7.886301	1.70766	0.036607	0.330455	down	chr12	1.12E+08	1.12E+08	-	hsa_circ_0028269	circBase	NM_0029	ATXN2	exonic	1307
chr9:8040	-3.2812	7.886301	1.70766	0.036607	0.330455	down	chr9	80409378	80430686	-	hsa_circ_0087264	circBase	NM_0020	GNAQ	exonic	414
chr19:362	-3.01641	7.826891	1.607771	0.041962	0.333399	down	chr19	36220059	36221363	+	hsa_circ_0050636	circBase	uc021usu.	KMT2B	exonic	418
chr3:2742	-3.01641	7.826891	1.607771	0.041962	0.333399	down	chr3	27424643	27493989	-	hsa_circ_0123427	circBase	NM_0036	SLC4A7	exonic	3530
chr3:1724	-3.01641	7.826891	1.607771	0.041962	0.333399	down	chr3	1.72E+08	1.73E+08	+		novel	NM_0180	ECT2	sense overl	24643
chr22:468	-3.01641	7.826891	1.607771	0.041962	0.333399	down	chr22	46859603	46860242	-	hsa_circ_0116806	circBase	NM_0142	CELSR1	exonic	639
chr15:915	-3.01641	7.826891	1.607771	0.041962	0.333399	down	chr15	91517365	91523619	-		novel	NM_0039	PRC1	exonic	639
chr1:1809	-3.01641	7.826891	1.607771	0.041962	0.333399	down	chr1	1.81E+08	1.81E+08	-	hsa_circ_0007905	circBase	NM_0058	STX6	exonic	391
chr9:3574	-2.94746	7.820487	1.549544	0.045472	0.33431	down	chr9	35740235	35740499	+		novel	NM_0209	GBA2	antisense	264
chr9:1141	-2.94746	7.820487	1.549544	0.045472	0.33431	down	chr9	1.14E+08	1.14E+08	-	hsa_circ_0088023	circBase	NM_0010	KIAA036	exonic	578
chr3:6541	-2.94746	7.820487	1.549544	0.045472	0.33431	down	chr3	65415194	65428524	-	hsa_circ_0003540	circBase	NM_0047	MAGI1	exonic	1089
chr3:5000	-2.94746	7.820487	1.549544	0.045472	0.33431	down	chr3	50004902	50012825	+	hsa_circ_0065769	circBase	NM_0057	RBM6	exonic	1439
chr19:187	-2.94746	7.820487	1.549544	0.045472	0.33431	down	chr19	1877830	1878066	-		novel	NM_0011	ABHD17A	intronic	236
chr5:8751	-2.94746	7.820487	1.549544	0.045472	0.33431	down	chr5	87516379	87541609	-	hsa_circ_0005476	circBase	NR_1102	TMEM161	sense overl	25230
chr19:130	-2.94746	7.820487	1.549544	0.045472	0.33431	down	chr19	13039155	13039661	-	hsa_circ_0000896	circBase	NM_0044	FARSA	exonic	338
chr19:402	-2.94746	7.820487	1.549544	0.045472	0.33431	down	chr19	4024033	4024656	+		novel	ENST000	PIAS4	intronic	623
chr10:495	-2.94746	7.820487	1.549544	0.045472	0.33431	down	chr10	49516137	49534008	+		novel	ENST000	MAPK8	exonic	263
chr20:344	-2.94746	7.820487	1.549544	0.045472	0.33431	down	chr20	34446223	34459751	+	hsa_circ_0003322	circBase	NM_0164	PHF20	exonic	942
chr12:194	-2.94746	7.820487	1.549544	0.045472	0.33431	down	chr12	19406873	19451985	+		novel	NM_0190	PLEKHA5	sense overl	45112
chr8:1311	-2.94746	7.820487	1.549544	0.045472	0.33431	down	chr8	1.31E+08	1.31E+08	-	hsa_circ_0085596	circBase	NM_0184	ASAP1	exonic	471
chr9:8091	-2.94746	7.820487	1.549544	0.045472	0.33431	down	chr9	80916869	80923499	+	hsa_circ_0139038	circBase	NM_0211	PSAT1	exonic	619
chr17:456	-2.94746	7.820487	1.549544	0.045472	0.33431	down	chr17	45636523	45643473	+		novel	ENST000	NPEPPS	intronic	6950

Supplementary Table 5

Accessior	Gene	Descriptic	Mw(kDa)	Length	Protein gr	Peptides c	Unique pe	Sequence	Intensity circ-	LFQ intensit	MS/MS co	SAF circ-1
P04264	KRT1	Keratin, ty	66.039	644		18	1	27.2	2221300000	798190000	18	0.02795
P62987	UBA52	Ubiquitin-6	14.728	128	P62979;P0	6	6	32.8	110220000	30543000	18	0.140625
P35579	MYH9	Myosin-9 (	226.532	1960	P35580;P3	12	12	7.1	32814000	6739300	9	0.004592
P63261	ACTG1	Actin, cyto	41.793	375	P60709;P6	6	6	15.2	229980000	67837000	7	0.018667
P15924	DSP	Desmoplak	331.774	2871		9	9	3.1	23787000	7500500	9	0.003135
P19338	NCL	Nucleolin (	76.614	710		6	6	6.6	72717000	20146000	6	0.008451
P10809	HSPD1	60 kDa he	61.055	573		5	5	8.9	23921000	6602800	5	0.008726
P52272	HNRNPM	Heterogen	77.516	730		6	6	9.5	37695000	11702000	6	0.008219
P06733	ENO1	Alpha-enol	47.169	434		4	4	9.7	15753000	5152800	4	0.009217
P25705	ATP5F1A	ATP synth	59.751	553		5	5	7.6	17451000	5370700	5	0.009042
P05141	SLC25A5	ADP/ATP t	32.852	298	P12236;P1	3	3	11.1	12257000	2692200	3	0.010067
P05783	KRT18	Keratin, ty	48.058	430		4	2	8.4	4742700	1472300	2	0.004651
P07437	TUBB	Tubulin be	49.671	444	Q9BVA1;C	4	4	11	7459900	1852300	4	0.009009
P08238	HSP90AB1	Heat shoc	83.264	724	Q58FF7;P	4	4	3.9	10539000	3069800	4	0.005525
P05089	ARG1	Arginase-1	34.735	322		2	2	3.7	10811000	4173300	2	0.006211
P07195	LDHB	L-lactate d	36.638	334	Q6ZMR3;F	2	2	6.9	11574000	3437400	2	0.005988
P07355	ANXA2	Annexin A	38.604	339	A6NMY6	2	2	5.6	6791500	2109200	2	0.0059
P13639	EEF2	Elongation	95.338	858		3	3	3	9267200	2700800	3	0.003497
P17844	DDX5	Probable A	69.148	614	Q92841	3	3	5	5932400	1508100	3	0.004886
P22087	FBL	rRNA 2'-O-	33.784	321		2	2	4	9106800	2330600	3	0.009346
P22455	FGFR4	Fibroblast	87.954	802		3	3	2.6	16957000	5264000	3	0.003741
P22626	HNRNPA2	Heterogen	37.43	353		4	4	11	8137100	2030600	3	0.008499
P26599	PTBP1	Polypyrimi	59.633	557		3	3	4.8	7422600	2290100	3	0.005386
Q86U44	METTL3	N6-adenos	67.681	580		3	3	6	10436000	3151200	3	0.004418
P62805	H4C1	Histone H4	11.367	103		1	1	11.7	11034000	3441600	3	0.029126
Q02413	DSG1	Desmoglei	113.748	1049		3	3	2.7	10479000	2874000	3	0.00286
Q08554	DSC1	Desmocoll	99.987	894		3	3	4.6	11371000	3215900	2	0.002237
Q15233	NONO	Non-POU	54.232	471		3	3	8.1	17642000	4987500	2	0.004246
Q6NXT2	H3-5	Histone H3	15.214	135	Q71DI3;Q	3	3	16.3	25837000	7265800	2	0.014815
P00533	EGFR	Epidermal	134.277	1210		1	1	0.7	1479800	459390	1	0.000826
P02788	LTF	Lactotrans	78.182	710		1	1	1.4	1801100	559120	1	0.001408
P04075	ALDOA	Fructose-b	39.42	364		2	2	6.3	4710100	840790	2	0.005495
P04406	GAPDH	Glyceralde	36.053	335		3	3	6.9	8215800	2550400	2	0.00597
P06576	ATP5F1B	ATP synth	56.56	529		1	1	2.3	4179100	807840	2	0.003781
P07910	HNRNPC	Heterogen	33.67	306		2	2	6.5	6194000	1682500	2	0.006536
P08670	VIM	Vimentin C	53.652	466	P41219	2	2	4.5	2904000	392540	1	0.002146
P09874	PARP1	Poly [ADP-	113.084	1014		2	2	2.1	3222500	911940	2	0.001972
P11021	HSPA5	Endoplasm	72.333	654		2	2	4.1	3056100	948700	2	0.003058
Q9Y5A9	YTHDF2	YTH doma	70.898	579		2	2	4.3	18196000	3147100	2	0.003096
P11586	MTHFD1	C-1-tetrahy	101.531	935		2	2	2.6	4139400	1284300	2	0.002139
P12268	IMPDH2	Inosine-5'-	55.805	514		2	2	3.5	4987800	1582000	1	0.001946
P14618	PKM	Pyruvate k	57.937	531		1	1	2.4	7654800	2376300	1	0.001883
P19105	MYL12A	Myosin reg	19.794	171	O14950;P2	1	1	6.4	3213100	0	1	0.005848
P23246	SFPQ	Splicing fa	76.149	707		2	2	3.8	3261800	1012600	2	0.002829
P23396	RPS3	40S riboso	26.688	243		2	2	9.1	3630300	876670	2	0.00823
P31689	DNAJA1	DnaJ homo	44.868	397		1	1	3.3	3415800	1060400	2	0.005038
P31944	CASP14	Caspase-1	27.68	242		2	2	8.7	2514200	780480	1	0.004132
P33993	MCM7	DNA replic	81.308	719		2	2	3.1	2799400	877400	2	0.002782
P34931	HSPA1L	Heat shoc	70.375	641	P0DMV9;F	2	2	3.6	4153600	1398000	2	0.00312
P40227	CCT6A	T-complex	58.024	531		2	2	4	2922100	892570	2	0.003766
P42704	LRPPRC	Leucine-ric	157.905	1394		2	2	0.6	6198300	1101700	1	0.000717
P43243	MATR3	Matrin-3 O	94.623	847		2	2	2.4	8928100	2632500	2	0.002361
P55291	CDH15	Cadherin-1	88.916	814		1	1	2.3	135370000	42022000	1	0.001229
P60174	TPI1	Triosephos	26.669	249		1	1	4.8	2687100	834160	1	0.004016
P61313	RPL15	60S riboso	24.146	204		1	1	4.4	4081900	1267200	1	0.004902
P62258	YWHAE	14-3-3 pro	29.174	255	P63104;P2	2	2	8.2	3478600	743690	2	0.007843
P62701	RPS4X	40S riboso	29.598	263	Q8TD47;P	2	2	7.6	13395000	4158300	2	0.007605
Q00610	CLTC	Clathrin he	191.615	1675		1	1	0.7	3234200	1004000	1	0.000597
Q00839	HNRNPU	Heterogen	90.584	825		2	2	2.8	3774900	817440	2	0.002424
Q14204	DYNC1H1	Cytoplasm	532.408	4646		2	2	0.5	3024400	938880	2	0.00043
Q5QJ74	TBCEL	Tubulin-sp	48.195	424		1	1	1.7	0	0	1	0.002358
Q5VSP4	LCN1P1	Putative lip	17.918	162	P31025	1	1	6.8	14455000	4487300	1	0.006173
Q86VP6	CAND1	Cullin-asso	136.376	1230		1	1	0.7	3419800	1061600	1	0.000813
Q96AE4	FUBP1	Far upstre	67.56	644		2	2	3.6	5369900	1542800	2	0.003106
Q99880	H2BC13	Histone H2	13.952	126	Q99879;Q	1	1	11.1	2419800	5622000	1	0.007937
Q9BQE3	TUBA1C	Tubulin alp	49.895	449	Q71U36;P	2	2	4.7	5243300	1450300	2	0.004454
O15523	DDX3Y	ATP-deper	73.154	660	O00571	1	1	1.5	1940100	602270	1	0.001515
O43143	DHX15	ATP-deper	90.933	795		1	1	1.1	1020900	316920	1	0.001258
O43175	PHGDH	D-3-phosp	56.651	533		1	1	2.1	3785600	1175200	1	0.001876
O75323	NIPSNAP2	Protein Nip	33.743	286		1	1	3.1	4905900	1522900	1	0.003497
O75390	CS	Citrate syn	51.712	466		1	1	2.4	3105300	963990	1	0.002146
O75533	SF3B1	Splicing fa	145.83	1304		1	1	0.7	1264800	392630	1	0.000767
P00505	GOT2	Aspartate a	47.518	430		1	1	2.3	1008900	313200	1	0.002326
P00558	PGK1	Phosphogl	44.615	417		1	1	2.4	4209000	1306600	1	0.002398
P02545	LMNA	Prelamin-A	74.139	664		1	1	1.5	1564400	485650	1	0.001506
P04040	CAT	Catalase C	59.756	527		1	1	1.7	1794700	557130	1	0.001898
P08758	ANXA5	Annexin A	35.937	320		1	1	2.8	1124000	348910	1	0.003125
P08865	RPSA	40S riboso	32.854	295	A0A8I5KQ	1	1	4.4	1589500	493430	1	0.00339
P11177	PDHB	Pyruvate d	39.233	359		1	1	3.3	0	0	1	0.002786
P13010	XRCC5	X-ray repa	82.705	732		1	1	1.1	0	0	1	0.001366

P14625	HSP90B1	Endoplasm	92.469	803		1	1	1.7	2378100	738240	1	0.001245
P14868	DARS1	Aspartate-	57.136	501		1	1	2	1665100	516890	1	0.001996
P14923	JUP	Junction pl	81.745	745		1	1	1.2	7000100	2173000	1	0.001342
P17987	TCP1	T-complex	60.344	556		1	1	2.3	2251100	698800	1	0.001799
P22681	CBL	E3 ubiquiti	123.516	906		1	1	4.4	2495100	774570	1	0.004032
P22234	PAICS	Bifunctiona	47.079	425		1	1	2.6	1205900	374350	1	0.002353
P25311	AZGP1	Zinc-alpha	34.259	298		1	1	3.4	2276400	706670	1	0.003356
P27708	CAD	CAD prote	242.984	2225		1	1	0.4	0	0	1	0.000449
P29401	TKT	Transketol	67.878	623		1	1	1.3	2587900	803360	1	0.001605
P31153	MAT2A	S-adenosy	43.661	395		1	1	3.8	1360800	422430	1	0.002532
P32119	PRDX2	Peroxiredo	21.892	198	Q06830	1	1	5.6	4446100	1380200	1	0.005051
P33991	MCM4	DNA replic	96.558	863		1	1	1.7	2594000	805250	1	0.001159
P34897	SHMT2	Serine hyd	55.993	504		1	1	1.8	2740900	850870	1	0.001984
P42285	MTREX	Exosome F	117.805	1042		1	1	0.8	0	0	1	0.00096
P45880	VDAC2	Voltage-de	31.567	294		1	1	2.7	2486300	771840	1	0.003401
P47897	QARS1	Glutamine-	87.799	775		1	1	1.4	1196400	371400	1	0.00129
P48741	HSPA7	Putative he	40.244	367	P17066	1	1	3	1769400	549280	1	0.002725
P49411	TUFM	Elongation	49.875	455		1	1	2.6	3189000	989970	1	0.002198
P50990	CCT8	T-complex	59.621	548		1	1	2.7	2536600	787430	1	0.001825
P55060	CSE1L	Exportin-2	110.417	971		1	1	1.1	1473100	457310	1	0.00103
P55072	VCP	Transitiona	89.322	806		1	1	1.2	1817100	564070	1	0.001241
P55265	ADAR	Double-str	136.066	1226		1	1	0.9	2279500	707620	1	0.000816
P60660	MYL6	Myosin ligh	16.93	151		1	1	8.6	2165500	672240	1	0.006623
P60842	EIF4A1	Eukaryotic	46.154	406	Q14240	1	1	2.5	3265700	1013800	1	0.002463
P61247	RPS3A	40S riboso	29.945	264		1	1	3.4	2701400	838600	1	0.003788
P62241	RPS8	40S riboso	24.205	208		1	1	6.2	4665600	1448300	1	0.004808
P62263	RPS14	40S riboso	16.273	151		1	1	7.3	5021500	1558800	1	0.006623
P62269	RPS18	40S riboso	17.719	152		1	1	7.2	1511000	469050	1	0.006579
P62280	RPS11	40S riboso	18.431	158		1	1	8.2	1952700	606180	1	0.006329
P62424	RPL7A	60S riboso	29.996	266		1	1	3.8	1059600	328940	1	0.003759
P62753	RPS6	40S riboso	28.681	249		1	1	3.2	1330600	413040	1	0.004016
P62826	RAN	GTP-bindi	24.423	216		1	1	5.1	2498000	775440	0	0
P78527	PRKDC	DNA-depe	469.089	4128		1	1	0.2	1305100	405140	1	0.000242
P84098	RPL19	60S riboso	23.466	196		1	1	5.1	335620	104190	0	0
Q00535	CDK5	Cyclin-dep	33.304	292	P06493;P2	1	1	2.7	2865900	889660	1	0.003425
Q01082	SPTBN1	Spectrin be	274.609	2364		1	1	0.4	1068600	331730	1	0.000423
Q02878	RPL6	60S riboso	32.728	288		1	1	3.1	3496200	1085300	1	0.003472
Q07020	RPL18	60S riboso	21.634	188		1	1	5.3	4296200	1333700	1	0.005319
Q07666	KHDRBS1	KH domair	48.227	443		1	1	3.2	1641100	509450	1	0.002257
Q08188	TGM3	Protein-glu	76.632	693		1	1	2.5	3295600	1023100	0	0
Q08J23	NSUN2	RNA cytos	86.471	767		1	1	1.3	1516000	470620	1	0.001304
Q13347	EIF3I	Eukaryotic	36.502	325		1	1	2.8	1669000	518100	1	0.003077
Q13547	HDAC1	Histone de	55.103	482	Q92769	1	1	2.5	1615700	501580	1	0.002075
Q14103	HNRNPD	Heterogen	38.434	355		1	1	3.9	3050100	946830	1	0.002817
Q15046	KARS1	Lysine--tRI	68.048	597		1	1	2.5	3342600	1037700	1	0.001675
Q53GG5	PDLIM3	PDZ and L	39.232	364		1	1	4.1	229800	71337	0	0
Q5VTE0	EEF1A1P5	Putative el	50.185	462	P68104;Q(	1	1	1.9	12454000	3866000	0	0
Q5VZ66	JAKMIP3	Janus kina	98.529	844		1	1	1.2	1715600	532570	1	0.001185
Q6EEV6	SUMO4	Small ubiq	10.653	95	P61956;P5	1	1	12.6	0	0	1	0.010526
Q8IYF1	ELOA2	Elongin-A2	83.921	753		1	1	1.1	0	0	1	0.001328
Q92945	KHSRP	Far upstre	73.115	711		1	1	1.5	2301700	714530	1	0.001406
Q96CN9	GCC1	GRIP and	87.811	775		1	1	1.4	3462700	1074900	1	0.00129
Q96PK6	RBM14	RNA-bindi	69.492	669		1	1	1.6	2118500	657630	1	0.001495
Q99623	PHB2	Prohibitin-2	33.296	299		1	1	4	1949300	605140	1	0.003344
Q9BTA0	FAM167B	Protein FA	18.414	163		1	1	3.7	2954800	917270	1	0.006135
Q9H1E1	RNASE7	Ribonuclea	17.419	156		1	1	7.7	2220500	689310	1	0.00641
Q9NZM1	MYOF	Myoferlin C	234.709	2061		1	1	0.6	2948700	915370	1	0.000485
Q9NZT1	CALML5	Calmodulir	15.893	146		1	1	5.5	2970600	922170	0	0
Q9UMS4	PRPF19	Pre-mRNA	55.181	504		1	1	2.4	2816500	874320	1	0.001984
Q9Y230	RUVBL2	RuvB-like 2	51.157	463		1	1	2.4	1189000	369090	1	0.00216
Q9Y265	RUVBL1	RuvB-like	50.228	456		1	1	2.4	2067700	641870	1	0.002193
Q9Y5T5	USP16	Ubiquitin c	93.57	823		1	1	1.8	1336700	414970	1	0.001215
O60271	SPAG9	C-Jun-ami	146.205	1321		1	1	0.7	1402200	435290	0	0
O94776	MTA2	Metastasis	75.023	668		1	1	1	0	0	0	0
P63010	AP2B1	AP-2 comp	104.553	937		1	1	1.1	1703300	528760	0	0
P85299	PRR5	Proline-ricl	42.753	388		1	1	3.4	307610	95490	0	0
Q7Z406	MYH14	Myosin-14	227.871	1995		1	1	0.5	614060	190620	0	0
Q8IUR7	ARMC8	Armadillo r	75.509	673		1	1	1.5	0	0	0	0
Q8NAC3	IL17RC	Interleukin-	86.24	791		1	1	1.6	743990	230960	0	0
Q8ND30	PPFIBP2	Liprin-beta	98.544	876		1	1	1.4	2264100	702830	0	0
Q8NI36	WDR36	WD repeat	105.322	951		1	1	0.6	0	0	0	0
Q8TD07	RAET1E	Retinoic ac	30.122	263		1	1	3.4	3955100	1227800	0	0
Q9UDT6	CLIP2	CAP-Gly d	115.837	1046		1	1	1	264380000	82072000	0	0

Accessior	Gene	Descriptio	Mw(kDa)	Length	Protein gr	Peptides a	Unique pe	Sequence	Intensity a	LFQ intens	MS/MS co	SAF anti-2
P04264	KRT1	Keratin, typ	66.039	644		18	1	27.2	8.56E+08	7.47E+08	9	0.013975
P62987	UBA52	Ubiquitin-6	14.728	128	P62979;P0	2	2	21.1	1828300	4141700	1	0.007813
P35579	MYH9	Myosin-9 O	226.532	1960	P35580;P3	10	10	5.9	15220000	18667000	5	0.002551
P63261	ACTG1	Actin, cytop	41.793	375	P60709;P6	6	6	16.5	1.12E+08	1.12E+08	5	0.013333
P15924	DSP	Desmoplak	331.774	2871		7	7	2.4	8257800	8141500	1	0.000348
P19338	NCL	Nucleolin O	76.614	710		4	4	5.6	6551400	8978800	1	0.001408
P10809	HSPD1	60 kDa hea	61.055	573		3	3	5.8	1922000	2745000	1	0.001745
P52272	HNRNPM	Heterogen	77.516	730		1	1	1.9	65785	65785	0	0
P06733	ENO1	Alpha-enol	47.169	434		4	4	7.8	8977400	8714700	1	0.002304
P25705	ATP5F1A	ATP syntha	59.751	553		3	3	5.8	3328000	3374600	0	0
P05141	SLC25A5	ADP/ATP t	32.852	298	P12236;P1	3	3	11.1	2061000	3173700	1	0.003356
P05783	KRT18	Keratin, typ	48.058	430		2	2	4.7	2317800	2317800	2	0.004651
P07437	TUBB	Tubulin bet	49.671	444	Q9BVA1;Q	2	2	5.6	1061900	1525300	0	0
P08238	HSP90AB1	Heat shock	83.264	724	Q58FF7;P0	2	2	2.9	1044000	1245800	0	0
P05089	ARG1	Arginase-1	34.735	322		2	2	6.8	1294000	476880	1	0.003106
P07195	LDHB	L-lactate de	36.638	334	Q6ZMR3;P	2	2	6.9	4313800	4469400	1	0.002994
P07355	ANXA2	Annexin A2	38.604	339	A6NMY6	2	2	5.6	4279400	4278500	1	0.00295
P13639	EEF2	Elongation	95.338	858		3	3	3	1719900	1896000	0	0
P17844	DDX5	Probable A	69.148	614	Q92841	1	1	2	213020	546460	0	0
P22087	FBL	rRNA 2'-O-	33.784	321		2	2	4	1659900	2156300	0	0
P22626	HNRNPA2	Heterogen	37.43	353		2	2	6.5	739360	1234800	0	0
P26599	PTBP1	Polypyrimic	59.633	557		2	2	3.4	738320	752380	0	0
Q86U44	METTL3	N6-adenos	67.681	580		3	3	6	1165600	1254200	0	0
P62805	H4C1	Histone H4	11.367	103		1	1	11.7	3650600	3634100	0	0
Q02413	DSG1	Desmoglei	113.748	1049		3	3	2.7	2890700	3269800	0	0
Q08554	DSC1	Desmocolli	99.987	894		3	3	4.6	4643300	4957300	1	0.001119
Q15233	NONO	Non-POU c	54.232	471		2	2	5.5	2097200	2586300	1	0.002123
Q6NXT2	H3-5	Histone H3	15.214	135	Q71DI3;Q1	1	1	5.2	9153800	9908700	1	0.007407
P00533	EGFR	Epidermal	134.277	1210		1	1	0.7	953340	953340	1	0.000826
P02788	LTF	Lactotransf	78.182	710		1	1	1.4	1532100	1532100	1	0.001408
P04075	ALDOA	Fructose-b	39.42	364		1	1	4.1	600930	1222300	0	0
P04406	GAPDH	Glyceralde	36.053	335		1	1	2.4	0	0	0	0
P07910	HNRNPC	Heterogen	33.67	306		1	1	3.6	219540	459880	0	0
P08670	VIM	Vimentin O	53.652	466	P41219	1	1	2.4	1438200	1947100	1	0.002146
P09874	PARP1	Poly [ADP-	113.084	1014		1	1	1.1	93348	181760	0	0
Q9Y5A9	YTHDF2	YTH doma	70.898	579		1	1	2.5	938590	1649900	0	0
P11586	MTHFD1	C-1-tetrahy	101.531	935		2	2	2.6	861550	862270	0	0
P12268	IMPDH2	Inosine-5'-r	55.805	514		2	2	3.5	1712300	1678600	1	0.001946
P14618	PKM	Pyruvate k	57.937	531		1	1	2.4	3568100	3568100	1	0.001883
P19105	MYL12A	Myosin reg	19.794	171	O14950;P2	1	1	6.4	2249200	2249200	1	0.005848
P23396	RPS3	40S riboso	26.688	243		1	1	3.7	495170	745450	0	0
P31944	CASP14	Caspase-1	27.68	242		1	1	4.1	2277600	2277600	1	0.004132
P33993	MCM7	DNA replic	81.308	719		2	2	3.1	523380	514980	0	0
P34931	HSPA1L	Heat shock	70.375	641	P0DMV9;P	2	2	3.6	684850	576280	0	0
P40227	CCT6A	T-complex	58.024	531		2	2	4	1006600	1021100	0	0
P42704	LRPPRC	Leucine-ric	157.905	1394		1	1	0.6	1521200	2343700	1	0.000717
P43243	MATR3	Matrin-3 O	94.623	847		1	1	1.1	170980	310080	0	0
P55291	CDH15	Cadherin-1	88.916	814		1	1	2.3	40274000	40274000	1	0.001229
P60174	TPI1	Triosephos	26.669	249		1	1	4.8	1119800	1119800	1	0.004016
P61313	RPL15	60S riboso	24.146	204		1	1	4.4	2111800	2111800	1	0.004902
P62258	YWHAE	14-3-3 prot	29.174	255	P63104;P2	1	1	4.3	769400	1105600	0	0
P62701	RPS4X	40S riboso	29.598	263	Q8TD47;P	1	1	3.4	1408300	1408300	0	0
Q00610	CLTC	Clathrin he	191.615	1675		1	1	0.4	0	0	1	0.000597
Q00839	HNRNPU	Heterogen	90.584	825		1	1	1	688270	1042700	0	0
Q14204	DYNC1H1	Cytoplasm	532.408	4646		1	1	0.2	1357900	1357900	0	0
Q5QJ74	TBCEL	Tubulin-sp	48.195	424		1	1	1.7	0	0	1	0.002358
Q5VSP4	LCN1P1	Putative lip	17.918	162	P31025	1	1	6.8	52460000	52460000	1	0.006173
Q86VP6	CAND1	Cullin-asso	136.376	1230		1	1	0.7	3131500	3131500	1	0.000813
Q96AE4	FUBP1	Far upstre	67.56	644		1	1	2	410740	534970	0	0
Q99880	H2BC13	Histone H2	13.952	126	Q99879;Q	2	2	23	8172100	3301300	1	0.007937
Q9BQE3	TUBA1C	Tubulin alp	49.895	449	Q71U36;P	1	1	2	112240	289640	0	0
O15523	DDX3Y	ATP-deper	73.154	660	O00571	1	1	1.5	432300	432300	0	0
O43143	DHX15	ATP-deper	90.933	795		1	1	1.1	237470	237470	0	0
O43175	PHGDH	D-3-phospl	56.651	533		1	1	2.1	488160	488160	0	0
O75323	NIPSNAP2	Protein Nip	33.743	286		1	1	3.1	3468600	3468600	0	0
O75390	CS	Citrate syn	51.712	466		1	1	2.4	358300	358300	0	0
O75533	SF3B1	Splicing fa	145.83	1304		1	1	0.7	741070	741070	0	0
P00505	GOT2	Aspartate a	47.518	430		1	1	2.3	387630	387630	0	0
P00558	PGK1	Phosphogl	44.615	417		1	1	2.4	94520	94520	0	0
P02545	LMNA	Prelamin-A	74.139	664		1	1	1.5	995510	995510	0	0
P04040	CAT	Catalase O	59.756	527		1	1	1.7	633670	633670	0	0
P08758	ANXA5	Annexin A5	35.937	320		1	1	2.8	233090	233090	0	0
P08865	RPSA	40S riboso	32.854	295	A0A8I5KQ	1	1	4.4	457390	457390	0	0
P14868	DARS1	Aspartate--	57.136	501		1	1	2	511620	511620	0	0
P14923	JUP	Junction pl	81.745	745		1	1	1.2	2883500	2883500	0	0
P25311	AZGP1	Zinc-alpha-	34.259	298		1	1	3.4	91450	91450	0	0
P29401	TKT	Transketol	67.878	623		1	1	1.3	1396100	1396100	0	0
P31153	MAT2A	S-adenosy	43.661	395		1	1	3.8	253000	253000	0	0
P34897	SHMT2	Serine hyd	55.993	504		1	1	1.8	618060	618060	0	0
P45880	VDAC2	Voltage-de	31.567	294		1	1	2.7	943740	943740	0	0
P47897	QARS1	Glutamine-	87.799	775		1	1	1.4	630970	630970	0	0
P48741	HSPA7	Putative he	40.244	367	P17066	1	1	3	1284500	1284500	0	0

P49411	TUFM	Elongation	49.875	455		1	1	2.6	218860	218860	0	0
P50990	CCT8	T-complex	59.621	548		1	1	2.7	348220	348220	0	0
P55060	CSE1L	Exportin-2	110.417	971		1	1	1.1	728560	728560	0	0
P55072	VCP	Transitiona	89.322	806		1	1	1.2	519770	519770	0	0
P55265	ADAR	Double-stra	136.066	1226		1	1	0.9	400960	400960	0	0
P60660	MYL6	Myosin ligh	16.93	151		1	1	8.6	180340	180340	0	0
P61247	RPS3A	40S riboso	29.945	264		1	1	3.4	884370	884370	0	0
P61978	HNRNPK	Heterogen	50.976	463		1	1	2.6	1126500	1126500	1	0.00216
P62263	RPS14	40S riboso	16.273	151		1	1	7.3	1808900	1808900	0	0
P62269	RPS18	40S riboso	17.719	152		1	1	7.2	837340	837340	0	0
P62280	RPS11	40S riboso	18.431	158		1	1	8.2	527120	527120	0	0
P62424	RPL7A	60S riboso	29.996	266		1	1	3.8	273080	273080	0	0
P62753	RPS6	40S riboso	28.681	249		1	1	3.2	528810	528810	0	0
P62826	RAN	GTP-bindir	24.423	216		1	1	5.1	1032800	1032800	1	0.00463
P84098	RPL19	60S riboso	23.466	196		1	1	5.1	1053600	1053600	1	0.005102
Q01082	SPTBN1	Spectrin be	274.609	2364		1	1	0.4	616420	616420	0	0
Q07020	RPL18	60S riboso	21.634	188		1	1	5.3	1325200	1325200	0	0
Q08188	TGM3	Protein-glu	76.632	693		1	1	2.5	1366500	1366500	1	0.001443
Q13347	EIF3I	Eukaryotic	36.502	325		1	1	2.8	421590	421590	0	0
Q53GG5	PDLIM3	PDZ and L	39.232	364		1	1	4.1	13851000	13851000	1	0.002747
Q5VTE0	EEF1A1P5	Putative el	50.185	462	P68104;Q(	1	1	1.9	4030300	4030300	1	0.002165
Q5VZ66	JAKMIP3	Janus kina	98.529	844		1	1	1.2	140950	140950	0	0
Q92945	KHSRP	Far upstrea	73.115	711		1	1	1.5	321920	321920	0	0
Q96CN9	GCC1	GRIP and	87.811	775		1	1	1.4	2121700	2121700	0	0
Q99623	PHB2	Prohibitin-2	33.296	299		1	1	4	915250	915250	0	0
Q9BTA0	FAM167B	Protein FA	18.414	163		1	1	3.7	1148600	1148600	0	0
Q9NZM1	MYOF	Myoferlin C	234.709	2061		1	1	0.6	2810600	2810600	0	0
Q9NZT1	CALML5	Calmodulir	15.893	146		1	1	5.5	2549500	2549500	1	0.006849
Q9UMS4	PRPF19	Pre-mRNA	55.181	504		1	1	2.4	291470	291470	0	0
Q9Y230	RUVBL2	RuvB-like 2	51.157	463		1	1	2.4	203750	203750	0	0
Q9Y265	RUVBL1	RuvB-like 1	50.228	456		1	1	2.4	476580	476580	0	0
O94776	MTA2	Metastasis	75.023	668		1	1	1	0	0	0	0
P63010	AP2B1	AP-2 comp	104.553	937		1	1	1.1	878580	0	0	0
Q7Z406	MYH14	Myosin-14	227.871	1995		1	1	0.5	423380	0	0	0
Q8IUR7	ARMC8	Armadillo r	75.509	673		1	1	1.5	0	0	0	0
Q8NAC3	IL17RC	Interleukin-	86.24	791		1	1	1.6	564710	0	0	0
Q8NI36	WDR36	WD repeat	105.322	951		1	1	0.6	0	0	0	0
Q8TD07	RAET1E	Retinoic ac	30.122	263		1	1	3.4	1216600	0	0	0
Q9UKK3	PARP4	Protein mo	192.595	1724		1	1	2.3	1492600	1492600	0	0

CBL
CBLC
SYTL4
PRKN
CBLB
SOCS5
UHRF1
ITCH
SMURF1
RBBP6
NEDD4L
NEDD4
SMURF2
RAPSN
ZEB2
WWP1
WWP2
SIAH2
SYVN1
NEURL1
ZMYND8
UHRF2
XIAP
