# Supplementary figures and images for: The N6-methyladenosine-mediated cLMNB1 degrades FGFR4 to overcome osimertinib resistance in non-small cell lung cancer
Source: Cell Death Dis. 2025 Nov 10;16(1):818. doi: 10.1038/s41419-025-08124-8 (PMC12603240; doi:10.1038/s41419-025-08124-8)

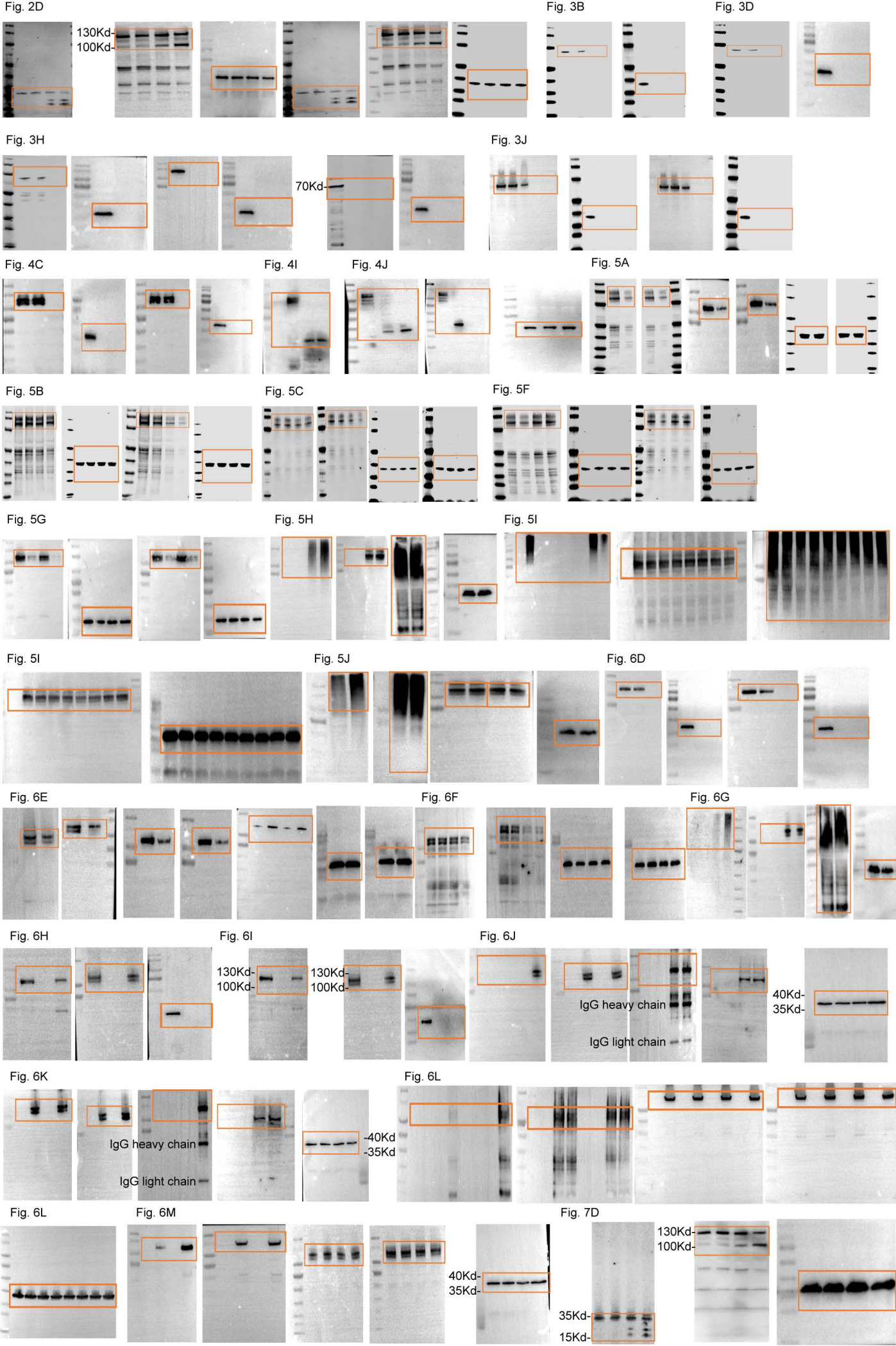

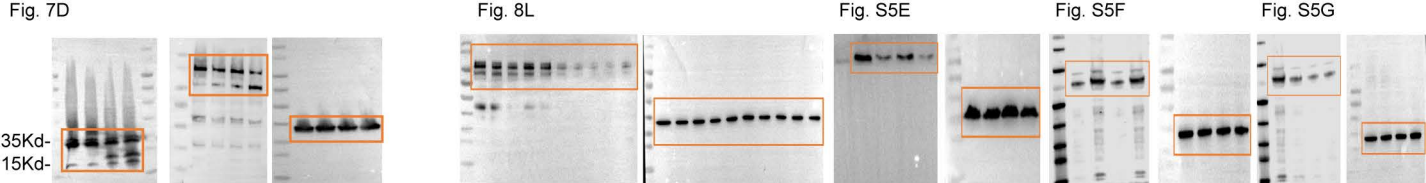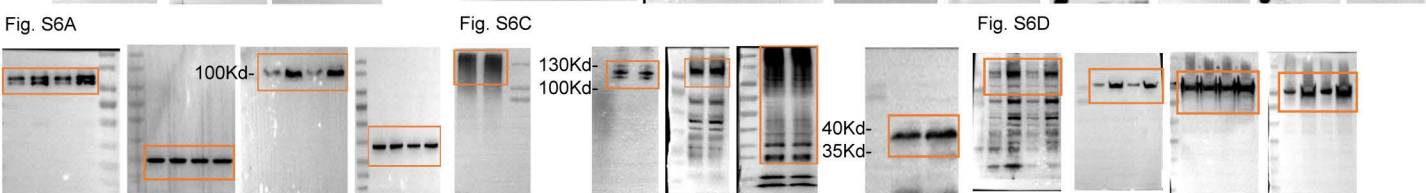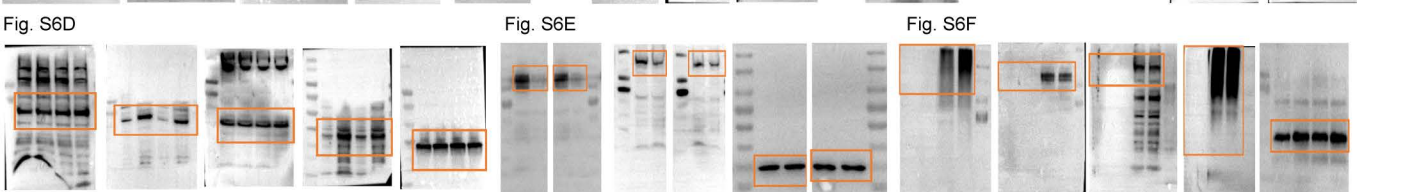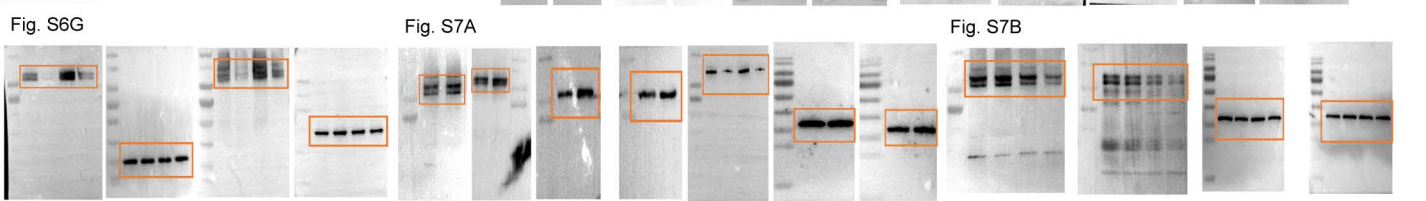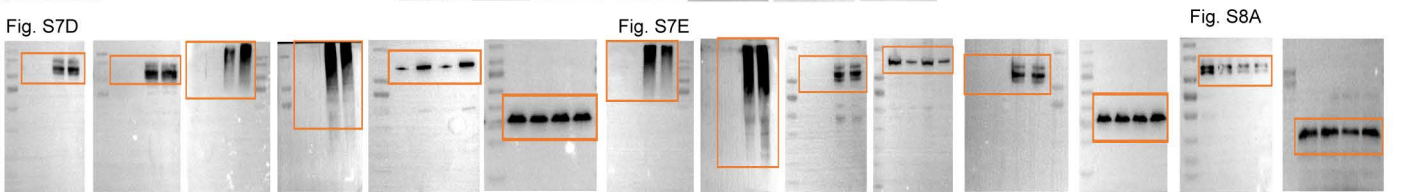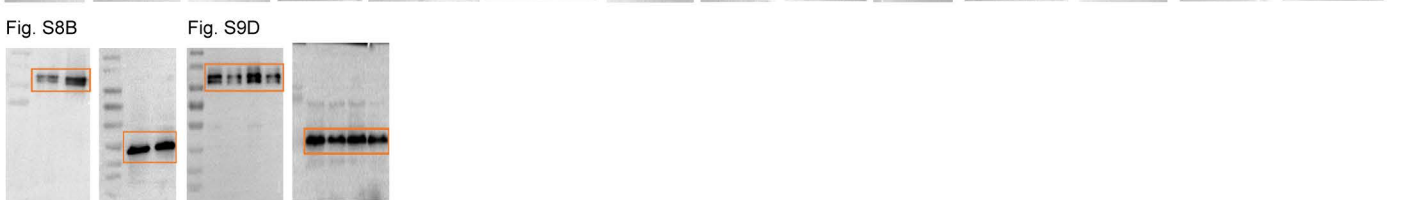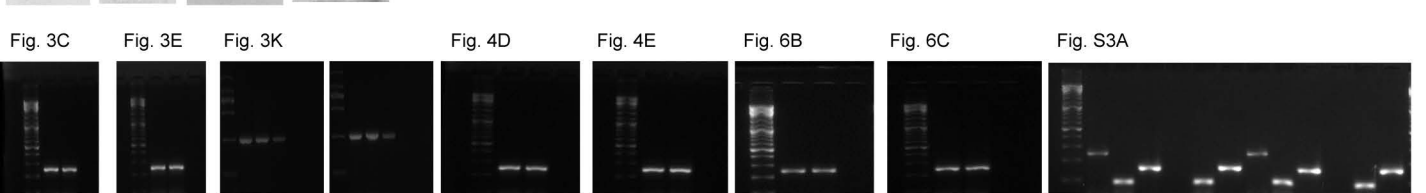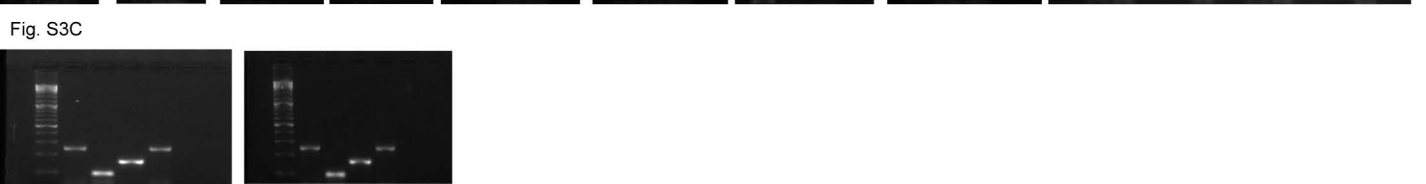

Supplement: Supplementary file 2 — Uncropped blots and gels [file 41419_2025_8124_MOESM2_ESM.pdf]
